# Adaptive Rhodium Catalysis with a Lewis‑Acidic Secondary Sphere for Divergent Hydrogenation of Propargylic Alcohols

**DOI:** 10.1002/anie.202515903

**Published:** 2025-10-01

**Authors:** Jiajun Wu, Vishal Chugh, Lachlan Sharp‐Bucknall, Ibrahim Abdellah, Alexandre Vasseur, Christophe Werlé

**Affiliations:** ^1^ Max Planck Institute for Chemical Energy Conversion Stiftstr. 34–36 45470 Mülheim an der Ruhr Germany; ^2^ Université de Lorraine CNRS, L2CM Metz F‐57000 France; ^3^ Université de Lorraine CNRS, L2CM Nancy F‐54000 France

**Keywords:** Adaptive catalysis, Divergent hydrogenation, Propargylic alcohols, Rhodium, Secondary‐sphere interactions

## Abstract

Achieving chemoselective transformations of multifunctional molecules under mild and sustainable conditions remains a central challenge in catalysis. Here, we show that a rhodium complex equipped with a Lewis acidic secondary coordination sphere can mediate condition‐dependent, divergent hydrogenation of propargylic alcohols. By tuning the reaction medium, the system selectively yields one of three products—retained alkynes, allylic ethers, or (*E*)‐alkenes—from a single substrate–catalyst pair under hydrogen. Mechanistic studies implicate π‐allyl and rhodium hydride intermediates, with solvent polarity and nucleophilicity steering the reaction pathway. Control experiments confirm the critical roles of both molecular hydrogen and the boron Lewis acid in enabling divergent selectivity. These findings demonstrate how rational secondary‐sphere design can enable adaptive catalysis and provide a platform for programmable bond transformations in multifunctional substrates.

## Graphical abstract

Adaptive rhodium catalysis with a Lewis‐acidic secondary sphere enables divergent hydrogenation of propargylic alcohols into alkynes, allylic ethers, or alkenes. Catalyst design and condition‐dependent pathways provide practical guidance for chemoselectivity control.

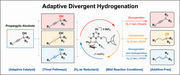

Adaptive catalysts—systems that modulate their reactivity in response to subtle environmental cues—represent a strategic frontier in selective synthesis.^[^ ^1^, ^2^, ^3^, ^4^, ^5^, ^6^, ^7^, ^8^ ^]^ In contrast, conventional catalysts, though often effective at favoring thermodynamic products, may lack the kinetic discrimination needed to distinguish between similar reactive sites. This limitation becomes particularly evident with multifunctional substrates, where competing pathways require careful control over chemo‐, regio‐, and stereoselectivity.

Molecular hydrogen (H_2_) is a highly atom‐economical and environmentally benign reductant,^[^ ^9^, ^10^ ^]^ and continues to attract interest for sustainable catalysis.^[^ ^11^, ^12^, ^13^ ^]^ However, with multifunctional substrates, especially under forcing conditions (e.g., elevated pressures or temperatures), conventional hydrogenation catalysts often fail to maintain chemoselectivity due to competing pathways.

Propargylic alcohols illustrate this challenge. These readily available building blocks feature orthogonal hydroxy and alkyne groups, supporting a diverse set of transformations^[^ ^14^ ^]^—including substitution,^[^ ^15^, ^16^, ^17^, ^18^, ^19^, ^20^, ^21^, ^22^, ^23^, ^24^, ^25^, ^26^, ^27^, ^28^, ^29^, ^30^, ^31^, ^32^, ^33^, ^34^, ^35^, ^36^ ^]^ rearrangement,^[^ ^37^, ^38^, ^39^, ^40^, ^41^, ^42^, ^43^ ^]^ and reduction (Scheme 1a1).^[^ ^44^, ^45^, ^46^, ^47^, ^48^, ^49^, ^50^ ^]^ Although protocols have been developed to achieve specific reduction outcomes from propargylic alcohols (Scheme 1a2), each transformation generally relies on a distinct catalytic system tailored to a single pathway—such as deoxygenation or reduction of alkynes to *cis*‐ or *trans*‐alkenes. Notably, these methods often require stoichiometric reductants (e.g., silanes or hydrides), and few offer effective chemoselectivity under hydrogen gas, highlighting a key limitation in sustainable reductive strategies. A single approach capable of selectively accessing multiple products from this functional motif—such as alkynes, allylic ethers, or alkenes—would streamline synthetic planning and facilitate access to scaffolds found in biologically active compounds (Scheme 1b).^[^ ^51^, ^52^, ^53^, ^54^, ^55^ ^]^

**Scheme 1:**
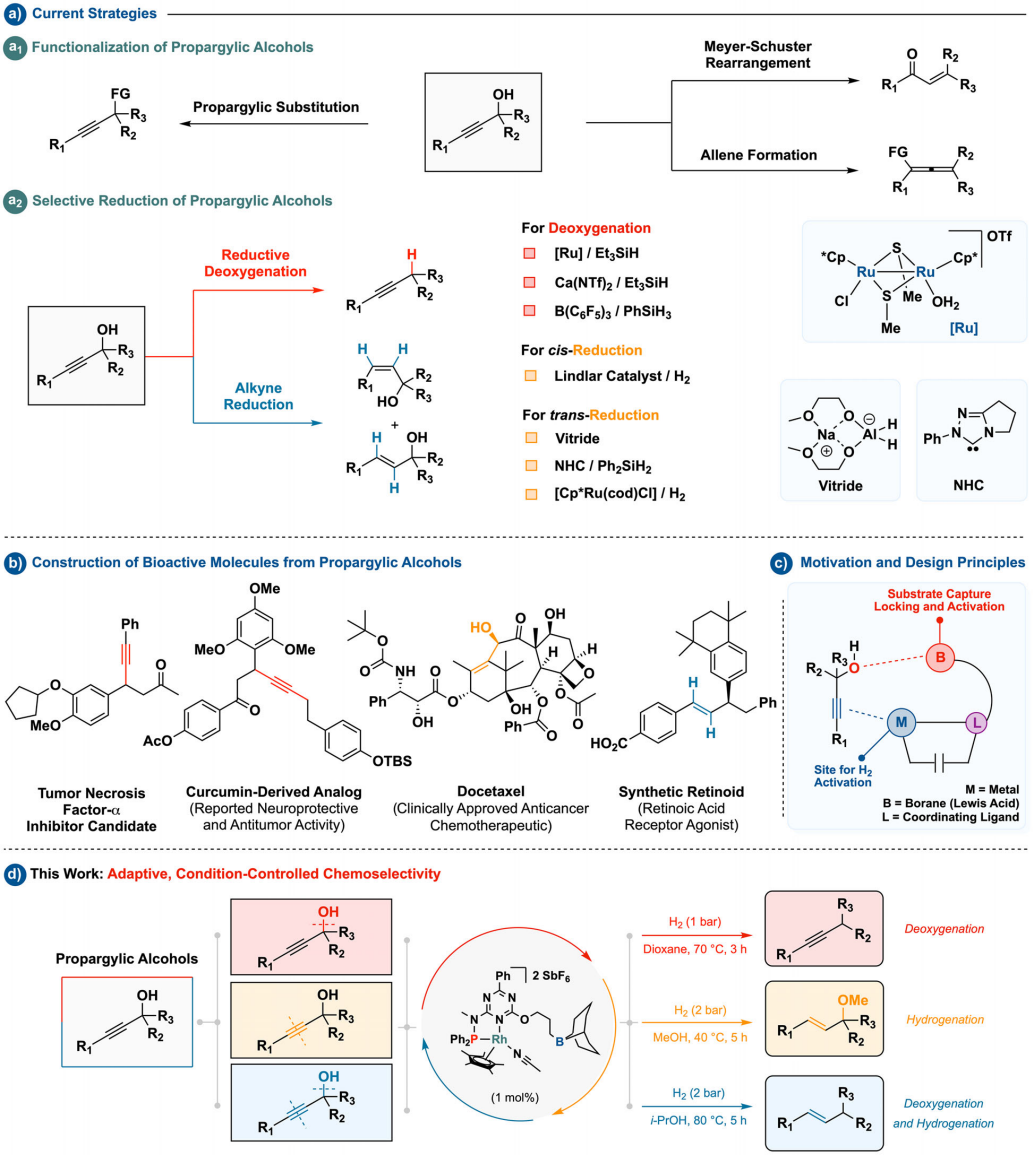
Conceptual overview and design framework for controlled hydrogenation of propargylic alcohols.

Inspired by this opportunity, we considered whether a catalyst combining a secondary coordination sphere for hydroxyl activation and a metal site for hydrogenation and/or alkyne binding could enable divergent reactivity (Scheme 1c). Such a system might engage the alcohol through Lewis acid–base interactions while promoting hydride formation or π‐activation at the metal center. In line with this design, we turned to a rhodium complex previously developed in our group for chemoselective nitroarene hydrogenation,^[^ ^4^ ^]^ which features a modular ligand incorporating a triazine π‐acceptor, a nitrogen‐tethered phosphine donor, and a proximal borane Lewis acid.^[^ ^56^, ^57^, ^58^, ^59^, ^60^, ^61^, ^62^, ^63^, ^64^, ^65^, ^66^, ^67^, ^68^, ^69^, ^70^, ^71^ ^]^ This architecture creates a polarized coordination environment capable of supporting both *inner*‐ and *outer*‐sphere processes. We reasoned that its reactivity could be shifted from heteroatom reduction to the more challenging context of multifunctional propargylic substrates.

Here, we report that this boron‐assisted rhodium complex enables condition‐dependent hydrogenation of propargylic alcohols to yield three distinct products under mild conditions (Scheme 1d). By modulating the reaction medium, the catalyst can be directed to favor the formation of retained alkynes, allylic ethers, or alkenes. These findings expand the scope of adaptive catalysis beyond heteroatom reduction, demonstrating that secondary‐sphere interactions and hydrogen activation can be orchestrated to direct divergent reactivity from a single scaffold—a hallmark of adaptive catalysis.

With the mechanistic hypothesis in hand, we first assessed whether rhodium complex **C1** could mediate condition‐dependent transformations of propargylic alcohols. We selected *1,3‐diphenylprop‐2‐yn‐1‐ol* (**1a**) as a benchmark substrate. Under initial conditions (1 mol% **C1** in dioxane, 80 °C, 2 bar H_2_, 5 h), hydrodeoxygenation to alkyne **2a** was the predominant outcome, forming in 65% yield alongside minor amounts of allylic alcohol **3aa** and (*E*)‐alkene **4a** (Table 1, entry 1). This outcome indicated that **C1** can engage and discriminate between both the hydroxyl and alkyne functionalities under a single catalytic platform, enabling multiple distinct reductive pathways. Systematic variation of reaction parameters revealed strong condition‐dependence in product distribution. Lowering the hydrogen pressure to 1 bar and reducing the temperature to 70 °C increased the yield of alkyne **2a** to 76%, indicating that milder conditions favor hydrodeoxygenation (Table 1, entry 7). In contrast, the choice of solvent had a marked effect on selectivity. When the reaction was conducted in isopropanol, alkene **4a** was obtained as the major product in 92% yield (Table 1, entry 4), whereas methanol under milder conditions (40 °C) favored the formation of allylic ether **3a** in 90% yield (Table 1, entry 8). These outcomes suggest that both the polarity and nucleophilicity of the medium influence the course of the reaction. Control experiments confirmed the homogeneous nature of the catalysis.^[^ ^72^, ^73^ ^]^ Neither mercury nor thiourea poisoning significantly affected the reactivity (Table 1, entries 9–11). In contrast, omission of H_2_ completely suppressed product formation, confirming the requirement of molecular hydrogen as the terminal reductant.

**Table 1:** Optimization of reaction conditions for divergent hydrogenation of propargylic alcohol **1a**.

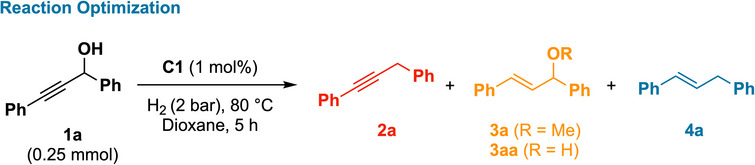			

Entry	Deviation from above	Conv. (%)^a)^	Yield (%)^a)^ **2a**:**3a**/**3aa**:**4a**
1	None	>99	65:0/7:9
2	THF	>99	60:0/6:12
3	MeOH	>99	0:74/0:26
4	*i*‐PrOH *(Condition C)*	>99	0:0/0:92
5	*t*‐Amyl alcohol	>99	0:0/0:89
6	Dioxane, 60 °C, 1 bar H_2_	92	67:0/4:9
7	Dioxane, 70 °C, 1 bar H_2_, 3 h *(Condition A)*	>99	76:0/3:7
8	MeOH, 40 °C *(Condition B)*	>99	0:90/0:2
9	*Condition A*, Hg (400 mol%)	95	70:0/0:7
10	*Condition B*, Hg (400 mol%)	>99	0:95/0:0
11	*Condition C*, Hg (400 mol%)	>99	0:0/0:94

^a)^ Conversions (%) and yields (%) are determined by ^1^H NMR using mesitylene (0.25 mmol) as the internal standard.

With three condition sets identified—*Condition A* (hydrodeoxygenation to alkynes), *Condition B* (allylic ether formation), and *Condition C* (alkene formation)—we next evaluated the generality of the rhodium complex across a series of propargylic alcohols (Scheme 2).

**Scheme 2:**
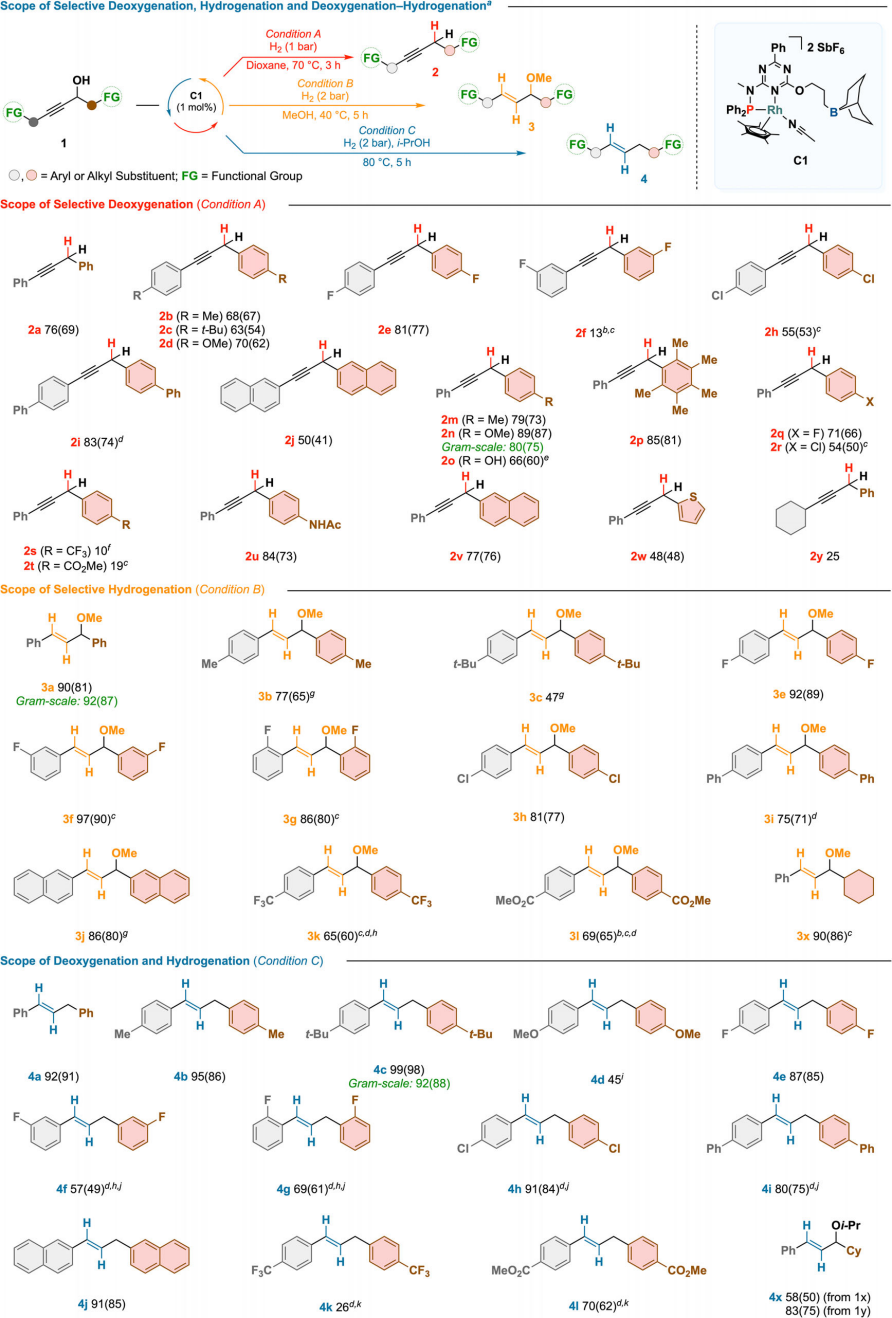
Scope of substrates under optimized conditions. ^a)^ *Condition A*: **1** (0.5 mmol), **C1** (1 mol%), dioxane (2 mL), H_2_ (1 bar), 70 °C, 3 h; *Condition B*: **1** (0.5 mmol), **C1** (1 mol%), MeOH (2 mL), H_2_ (2 bar), 40 °C, 5 h; *Condition C*: **1** (0.5 mmol), **C1** (1 mol%), *i‐*PrOH (2 mL), H_2_ (2 bar), 80 °C, 5 h; yields were determined by ^1^H NMR using mesitylene (0.5 mmol) as an internal standard; isolated yields are given in parentheses. ^b)^ **C1** (2 mol%). ^c)^80 °C. ^d)^Substrate in 0.25 mmol scale. ^e)^ *i*‐PrOH, 2 bar, 60 °C, 5 h. ^f)^16 h. ^g)^3 h. ^h)^ **C1** (3 mol%). ^i)^MeOH, 40 °C. ^j)^100 °C. ^k)^ **C1** (5 mol%), 120 °C.

Under *Condition A*, substrates bearing electron‐donating substituents (Me, *t*‐Bu, OMe, and OH) on the aryl ring afforded alkynes **2b–2d** and **2m–2p** in good yields. Halogenated substrates (*p*‐F, *p*‐Cl) were similarly well tolerated, furnishing **2e**, **2h**, **2q**, and **2r**. In contrast, electron‐deficient motifs such as CF_3_, CO_2_Me, and *m*‐F led to diminished reactivity, with only trace to moderate yields of **2f** and **2s–2t**. Notably, amide‐substituted alcohol **1u** gave alkyne **2u** in 84% yield, suggesting good chemoselectivity toward reductive deoxygenation in the presence of polar functional groups.^[^ ^74^ ^]^ Heterocyclic, biphenyl, and naphthyl substrates (**2i**, **2j**, **2v**, **2w**) were also compatible. However, purely aliphatic substrate **1y** showed low conversion, yielding only 25% of **2y**, consistent with a preference for π‐rich aryl systems under these conditions.

Applying *Conditions B* and *C* to the same substrate pool enabled divergent access to allylic ethers (**3b–3j**) and alkenes (**4b–4j**), maintaining broad compatibility. Aryl substrates with electron‐donating or halogen substituents underwent smooth conversion under both conditions, with representative yields up to 99%. Biphenyl and naphthyl derivatives also performed well, affording **3i–3j** and **4i–4j** in high yields. In contrast, substrates bearing strongly electron‐withdrawing groups (e.g., CF_3_, CO_2_Me) required increased temperature and catalyst loading to achieve moderate yields of **3k–3l** and **4k–4l**. For the cyclohexyl‐substituted substrate **1x**, *Condition B* afforded ether **3x** (90%), while *Condition C* yielded alkene **4x** (83%) with retained isopropyl ether functionality—an exception to the typical full deoxygenation observed under these conditions. For unsymmetrical substrates bearing electronically distinct aryl groups, reactions under *Conditions B* and *C* generally afforded near 1:1 mixtures of regioisomeric ethers and alkenes. These outcomes likely reflect similar accessibility of both aryl termini during the transformation (see the Supporting Information, Scheme S1, for full product distributions and yields).

To evaluate the operational practicality of the system, representative substrates were subjected to *gram‐scale* reactions. Substrates **1a** (4.75 mmol), **1c** (3.5 mmol), and **1n** (4.5 mmol) were subjected to the reaction conditions appropriate for each target product, affording alkyne **2n** (75%, 0.75 g), ether **3a** (87%, 0.93 g), and alkene **4c** (88%, 0.94 g) without loss of efficiency or selectivity. These results demonstrate that the method is readily scalable without compromising efficiency or selectivity, supporting its potential application in synthetic campaigns.

To elucidate the origins of selectivity and the role of catalyst design in governing divergent reactivity, we compared rhodium complex **C1** to a series of structurally related analogues under representative conditions (Scheme 3). The boron‐free complex **C2**, previously shown to catalyze the semihydrogenation of internal alkynes,^[^ ^8^ ^]^ displayed diminished activity and selectivity, affording only 30% of alkyne **2a** under *Condition A* and 70% of alkene **4a** under *Condition C*. Supplementing **C2** with a boron‐based additive (**C3**) partially restored activity, suggesting that boron‐mediated secondary‐sphere interactions contribute critically to both reactivity and selectivity, particularly during deoxygenation.^[^ ^75^ ^]^ In contrast, benchmark hydrogenation catalysts (**C4**–**C9**) failed to yield the target products under any of the tested conditions, instead producing mixtures of side products, highlighting the distinct reactivity of **C1**.

**Scheme 3:**
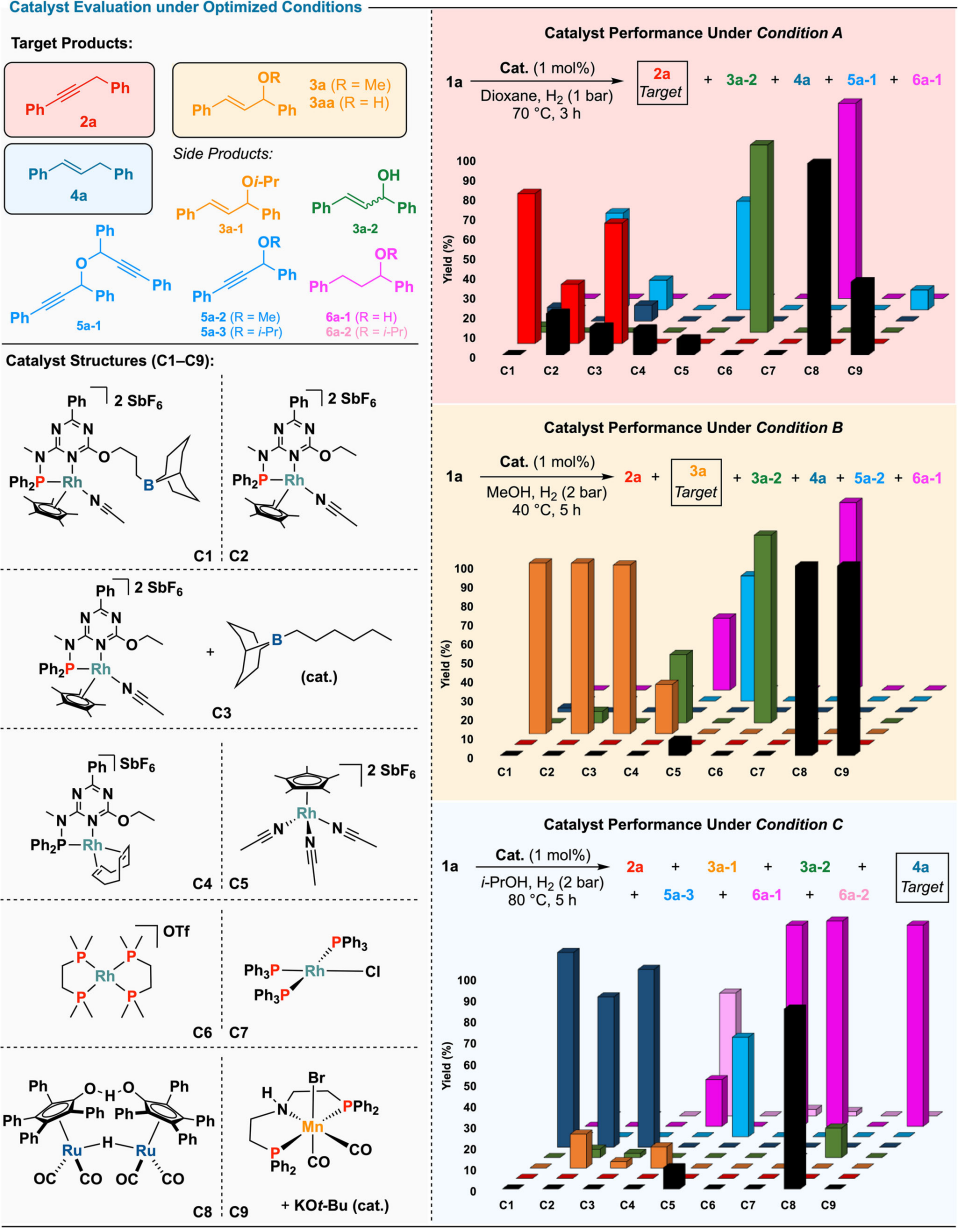
Comparative catalyst screening under standard conditions. Black bars indicate the remaining unreacted starting material **1a**.

Further mechanistic studies with **C1** revealed a condition‐responsive, stepwise network (Scheme 4). In the absence of hydrogen, propargylic alcohol **1a** underwent etherification to yield a mixture of propargylic ethers (**5a‐1**, **5a‐2**, **5a‐3**), with the product distribution dependent on solvent (Scheme 4a). Under hydrogenative conditions, **5a‐1** was selectively converted to alkyne **2a** (Scheme 4b), supporting the role of ether intermediates in the hydrodeoxygenation pathway.^[^ ^76^ ^]^

**Scheme 4:**
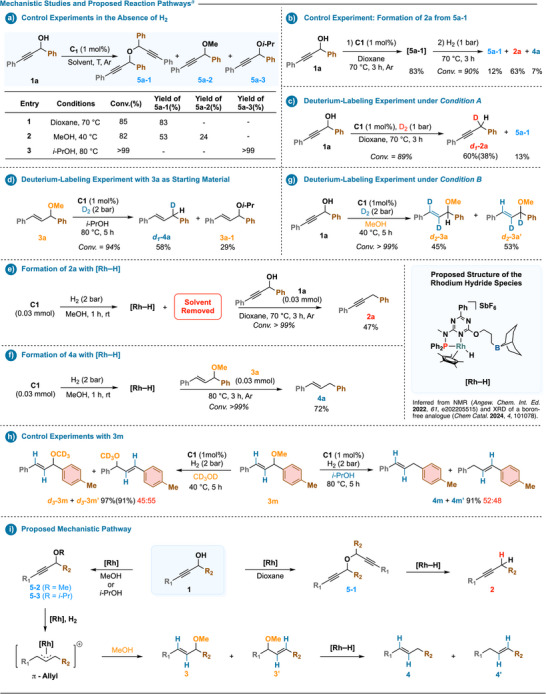
Empirical mechanistic studies and tentative catalytic cycle. ^a)^Yields were determined by ^1^H NMR using an internal standard, as detailed in the Supporting Information; isolated yields are provided in parentheses.

To probe the origin of hydrogen incorporation during reduction, deuterium‐labeling experiments were conducted under *Conditions A* and *C*. Exposure of **1a** to D_2_ (1 bar) under *Condition A* led to ***d*_1_‐2a** in 60% yield (Scheme 4c), while treatment of **3a** under *Condition C* afforded ***d*_1_‐4a** in 58% yield (Scheme 4d). In both cases, deuterium incorporation occurred at the propargylic or allylic position, consistent with reduction mediated by a metal hydride species. To directly evaluate the involvement of rhodium hydride intermediates, a stoichiometric [**Rh–H**] complex was generated in situ from **C1** via a reported protocol.^[^ ^8^ ^]^ This species converted **1a** and **3a** into **2a** (47%) and **4a** (72%), respectively (Scheme 4e,f), supporting its role as a common reductive intermediate in both transformations.^[^ ^77^ ^]^

To gain further insight into the ether formation pathway under *Condition B*, additional deuterium‐labeling experiments were carried out. Reaction of **1a** with D_2_ (2 bar) gave a near 1:1 mixture of *trans*‐***d*_2_‐3a** and ***d*_2_‐3a’** (Scheme 4g), while transformation of **3m** in *d*_4_‐methanol afforded the corresponding labeled ethers ***d*_3_‐3m** and ***d*_3_‐3m’** in 97% combined yield (Scheme 4h, left). These results indicate that methanol acts as a nucleophile, displacing the methoxy group in a substitution process. Upon further hydrogenation, **3m** was converted to alkenes **4m** and **4m’** in 91% yield (Scheme 4h, right). The formation of regioisomeric and isotope‐labeled products from both ether and olefin precursors supports a common intermediate accessible via C─O bond activation, consistent with the involvement of a π‐allyl‐type species,^[^ ^78^, ^79^, ^80^, ^81^, ^82^, ^83^, ^84^, ^85^, ^86^, ^87^, ^88^, ^89^, ^90^, ^91^, ^92^, ^93^ ^]^ likely formed through rhodium‐catalyzed activation of propargylic ethers.

Taken together, these findings support a unified mechanistic model for the condition‐dependent transformation of propargylic alcohols (Scheme 4i). Under nonreductive conditions, initial etherification leads to propargylic ethers (**5**), which serve as common intermediates. In aprotic solvents such as dioxane, these ethers undergo deoxygenation via rhodium hydride species to afford alkynes (**2**). In contrast, protic solvents like methanol or isopropanol promote C─O bond activation, enabling substitution to form allylic ethers (**3/3’**) at lower temperatures, or further reduction to alkenes (**4/4’**) at elevated temperatures via sequential hydrogenation. This condition‐responsive divergence highlights how subtle changes in the reaction environment can steer product selectivity through a shared intermediate pathway.

In summary, we have developed a condition‐responsive method for the divergent hydrogenation of propargylic alcohols using a boron‐assisted rhodium complex. By tuning the reaction medium, a single substrate–catalyst combination can selectively yield alkynes, allylic ethers, or alkenes with high selectivity. Mechanistic studies support the involvement of rhodium hydride and allylic‐type intermediates, with molecular hydrogen playing a central role in both reductive pathways.

These findings illustrate how secondary‐sphere design can enable adaptive reactivity through modulation of reaction conditions. More broadly, the ability to steer divergent product outcomes from a common intermediate platform suggests a powerful approach to programmable catalysis. This strategy may ultimately be extended to enable chemo‐, regio‐, or even enantioselective control in other transformation classes—including C─C bond formation or multistep cascade processes—using a single, tunable catalytic architecture. Such adaptive systems challenge the traditional notion of “one catalyst, one transformation” and open new directions for reactivity control in complex molecular settings.

## Author Contributions

J.W. performed the experiments, carried out analytical characterizations, and prepared the initial manuscript draft. J.W., V.C., and L.S.B. jointly evaluated the substrate scope. I.A. and A.V. contributed technical support in experiment execution and data analysis. C.W. conceived and supervised the project and led the manuscript revision with input from all authors. All authors reviewed and approved the final version of the manuscript.

## Conflict of Interests

The authors declare no conflict of interest.

## Supporting information



Supporting Information

## Data Availability

The data that support the findings of this study (e.g., general considerations, experimental methods, synthetic details, copies of NMR spectra) are available in the Supporting Information of this article.
