# Supplementary material for: Adaptive Rhodium Catalysis with a Lewis‑Acidic Secondary Sphere for Divergent Hydrogenation of Propargylic Alcohols
Source: Angew Chem Int Ed Engl. 2025 Oct 1;64(49):e202515903. doi: 10.1002/anie.202515903 (PMC12668305; doi:10.1002/anie.202515903)
Supplement: Supplementary file 1 — Supporting Information [file ANIE-64-e202515903-s001.pdf]

Supporting Information

Adaptive Rhodium Catalysis with a Lewis-Acidic Secondary Sphere for Divergent Hydrogenation of Propargylic Alcohols

Jiajun Wu,[1] Vishal Chugh,[1] Lachlan Sharp-Bucknall,[1] Ibrahim Abdellah,[2] Alexandre Vasseur,[3] and Christophe Werlé[1,3,]*

[1] Max Planck Institute for Chemical Energy Conversion, Stiftstr. 34 – 36, 45470 Mülheim an der Ruhr, Germany.

[2] Université de Lorraine, CNRS, L2CM, F-57000 Metz, France

[3] Université de Lorraine, CNRS, L2CM, F-54000 Nancy, France

* Email: christophe.werle@cec.mpg.de; christophe.werle@univ-lorraine.fr

1. Methods and Procedures **S4**
2. Preparation of Propargylic Alcohols **S5**
2.1 General Procedure for the Preparation of Propargylic Alcohols **1a–1k**; **1m-y** S5
2.2 Preparation of Propargylic Alcohols **1l** S17
3. Optimization of Reaction Parameters **S18**
3.1 Optimization of Reaction Conditions for **2a** S18
3.2 Optimization of Reaction Conditions for **3a** S19
3.3 Optimization of Reaction Conditions for **4a** S19
4. Asymmetric Substrate Scope Under *Conditions B* and *C* **S20**
5. Preparation of Alkynes 2 .... **S21**
5.1 General Procedure for the Preparation of Alkynes **2** S21
5.2 Compounds monitored by crude ^{1}H NMR S29
6. Preparation of Allyl Ether Derivatives 3 .... **S33**
6.1 General Procedure for the Preparation of Allyl Ether Derivatives **3** S33
6.2 Compounds monitored by crude ^{1}H NMR S42
7. Preparation of *trans*-Alkenes 4 .... **S43**
7.1 General Procedure for the Preparation of *trans*-Alkenes **4** S43
7.2 Compounds monitored by crude ^{1}H NMR S54
8. *Gram-scale* Procedure for the Synthesis of 2n, 3a, and 4c **S56**
8.1 Synthesis of **2n** S56
8.2 Synthesis of **3a** S56
8.3 Synthesis of **4c** S57
9. Additional Tests with Cyano-, Ketone-, and Nitro-Substituted Propargylic Alcohols **S58**
10. Catalyst Screening under Optimized Conditions **S59**
11. Mechanistic Studies **S77**
11.1 Control Experiments in the Absence of H_2 .... S77
11.2 Control Experiments with Boron-Based Additive Alone S78
11.3 Control Experiment: Formation of **2a** from **5a-1** using **C1** S79
11.4 Control Experiment: Formation of **2a** from **5a-1** using **C2** S80
11.5 Conversion of Isolated Propargylic Ether **5a-3** to Allylic Ether **3a** under *Condition B* S81
11.6 Time-Course Study for **1a** → **5a-1** → **2a** under *Condition A* S82
11.7 Deuterium-Labeling Experiment under *Condition A* S83
11.8 Deuterium-Labeling Experiment Starting from **3a** S84
11.9 Formation of **2a** with **[Rh–H]** S85
11.10 Formation of **4a** with **[Rh–H]** S85
11.11 Deuterium-Labeling Experiment under *Condition B* S86
11.12 Control Experiments with **3m** S87

11.12.1 Under *Condition B* with CD_3OD S87

11.12.2 Under *Condition C* S88

12. NMR spectra S90

13. References S178

1. Methods and Procedures

All reactions were prepared within an MBraun glovebox under an argon atmosphere. Solvents employed in air- and moisture-sensitive experiments were purified using a two-column solvent purification system (MBraun SPS-7) and transferred directly into the glovebox for storage over molecular sieves (3 or 4 Å). Technical grade solvents were used for workup and purification procedures.

All reagents, sourced from abcr, Alfa Aesar, or Sigma-Aldrich at the highest commercial quality, were used without further purification. Conversions and yields were determined spectroscopically *via* ^{1}H NMR with the indicated internal standard. Products were isolated by silica gel column chromatography (60 Å, 230–400 mesh particle size) or preparative TLC on SIL G-200 UV_{254} (20 × 20 cm, 2 mm) glass plates.

Routine solution-state NMR spectra were recorded on a Bruker Avance NEO 400 MHz spectrometer equipped with a standard 5 mm BBFO probehead. ^{1}H and ^{13}C chemical shifts (δ) are expressed in parts per million (ppm) and referenced to residual solvent signals: $CDCl_3$ at $\delta(^{1}H) = 7.26$ ppm and $\delta(^{13}C) = 77.16$ ppm; CD_3OD at $\delta(^{1}H) = 3.31$ ppm and $\delta(^{13}C) = 49.00$ ppm. Coupling constants (*J*) are reported in Hertz (Hz). Abbreviations used to denote multiplicities are: s = singlet, d = doublet, dd = doublet of doublets, t = triplet, q = quartet, tt = triplet of triplets, m = multiplet, and bs = broad singlet.

High-resolution mass spectra (HR-MS) were recorded using a Bruker ESQ3000 spectrometer. Gas chromatography-mass spectrometry (GC-MS) was conducted on a Shimadzu QP2020 instrument.

Disclaimer:

CAUTION: *When opening a Fisher-Porter tube, wear gloves and work behind a protective shield inside a fume hood. The headspace may contain hydrogen gas, which can be released upon opening. Ensure the area is free of ignition sources, and vent the gas slowly to avoid pressure-related hazards.*

---

2. Preparation of Propargylic Alcohols

2.1 General Procedure for the Preparation of Propargylic Alcohols 1a–1k; 1m-y

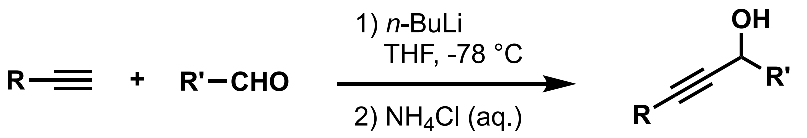

1

n-BuLi (1.6 M in hexanes, 3.5 mL, 5.6 mmol) was added dropwise to a stirred solution of the terminal alkyne (5.0 mmol) in anhydrous THF (10 mL) at –78 °C under an argon atmosphere. The resulting mixture was stirred at –78 °C for 1 h. A solution of the corresponding aldehyde (5.0 mmol) in THF (5 mL) was then added dropwise. The reaction mixture was allowed to warm to room temperature and stirred for an additional 2 h. The reaction was quenched with saturated aqueous NH_4Cl, and the mixture was extracted with EtOAc (3 × 20 mL). The combined organic layers were dried over anhydrous $MgSO_4$, filtered, and concentrated under reduced pressure. The crude residue was purified by flash column chromatography to afford the corresponding propargylic alcohols **1a–1k** and **1m–1y**.

1,3-Diphenylprop-2-yn-1-ol (1a)[1]

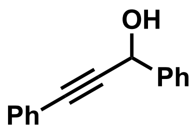

Prepared according to the general procedure using phenylacetylene (510 mg, 5.00 mmol) and benzaldehyde (530 mg, 5.00 mmol). The crude product was purified by flash column chromatography on silica gel (pentane/EtOAc = 5:1) to afford **1a** as a colorless oil (1.01 g, 97%).

Formula: $C_{15}H_{12}O$

Molecular weight: 208.26

Isolated yield: 97% (1.01 g)

Rf: 0.4 (Pentane/EtOAc = 5:1)

^{1}H NMR (400 MHz, $CDCl_3$, 298 K) δ 7.56 (d, *J* = 7.2 Hz, 2H), 7.42 (dd, *J* = 7.2, 2.1 Hz, 2H), 7.38 – 7.22 (m, 6H), 5.62 (s, 1H), 2.72 (s, 1H)

^{13}C NMR (101 MHz, $CDCl_3$, 298 K) δ 140.7, 131.8, 128.7, 128.7, 128.5, 128.4, 126.8, 122.5, 88.9, 86.7, 65.1

1,3-Di-*p*-tolylprop-2-yn-1-ol (1b)[2]

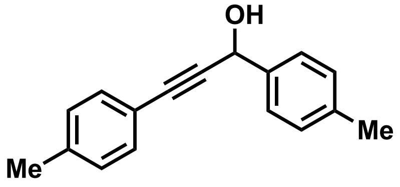

Prepared according to the general procedure using 4-methylphenylacetylene (581 mg, 5.00 mmol) and 4-methylbenzaldehyde (600 mg, 5.00 mmol). The crude product was purified by flash column chromatography on silica gel (pentane/EtOAc = 5:1) to afford **1b** as a white solid (1.17 g, 99%).

Formula: $C_{17}H_{16}O$

Molecular weight: 236.31

Isolated yield: 99% (1.17 g)

Rf: 0.4 (Pentane/EtOAc = 5:1)

^{1}H NMR (400 MHz, CDCl$_3$, 298 K) δ 7.51 (d, *J* = 8.0 Hz, 2H), 7.37 (d, *J* = 8.0 Hz, 2H), 7.21 (d, *J* = 7.9 Hz, 2H), 7.12 (d, *J* = 7.9 Hz, 2H), 5.65 (d, *J* = 6.2 Hz, 1H), 2.37 (s, 3H), 2.35 (s, 3H), 2.23 (d, J = 6.2 Hz, 1H)

^{13}C NMR (101 MHz, CDCl$_3$, 298 K) δ 138.9, 138.4, 138.1, 131.8, 129.5, 129.2, 126.9, 119.5, 88.3, 86.8, 65.2, 21.6, 21.3

1,3-Bis(4-(*tert*-butyl)phenyl)prop-2-yn-1-ol (1c)

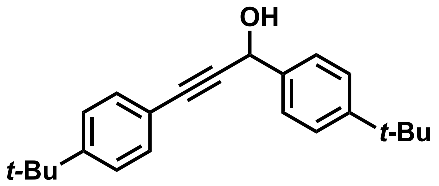

Prepared according to the general procedure using 4-(*tert*-butyl)phenylacetylene (791 mg, 5.00 mmol) and 4-(*tert*-butyl)benzaldehyde (811 mg, 5.00 mmol). The crude product was purified by flash column chromatography on silica gel (pentane/EtOAc = 10:1) to afford **1c** as a white solid (1.40 g, 87%).

Formula: $C_{23}H_{28}O$

Molecular weight: 320.48

Isolated yield: 87% (1.4 g)

Rf: 0.4 (Pentane/EtOAc = 10:1)

^{1}H NMR (400 MHz, CDCl$_3$, 298 K) δ 7.56 (d, *J* = 8.5 Hz, 2H), 7.47 – 7.39 (m, 4H), 7.34 (d, *J* = 8.5 Hz, 2H), 5.67 (d, *J* = 4.3 Hz, 1H), 2.20 (d, *J* = 4.3 Hz, 1H), 1.33 (s, 9H), 1.31 (s, 9H).

^{13}C NMR (101 MHz, CDCl$_3$, 298 K) δ 152.0, 151.6, 138.0, 131.6, 126.7, 125.8, 125.4, 119.6, 88.3, 86.8, 65.1, 34.9, 34.8, 31.5, 31.3

HRMS (ESI$^+$): Calcd. for $C_{23}H_{28}ONa^+$ $[M + Na]^+$: 343.20323; Found 343.20291

1,3-Bis(4-methoxyphenyl)prop-2-yn-1-ol (1d)[3]

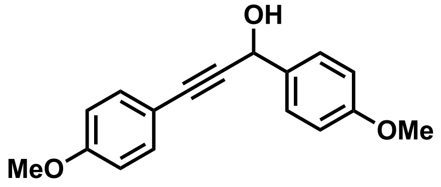

Prepared according to the general procedure using 4-methoxyphenylacetylene (661 mg, 5.00 mmol) and 4-methoxybenzaldehyde (680 mg, 5.00 mmol). The crude product was purified by flash column chromatography on silica gel (pentane/EtOAc = 3:1) to afford **1d** as a yellow oil (1.20 g, 89%).

Formula: $C_{17}H_{16}O_3$

Molecular weight: 268.31

Isolated yield: 89% (1.20 g)

Rf: 0.3 (Pentane/EtOAc = 3:1)

^{1}H NMR (400 MHz, CDCl$_3$, 298 K) δ 7.54 (d, *J* = 8.6 Hz, 2H), 7.41 (d, *J* = 8.7 Hz, 2H), 6.92 (d, *J* = 8.6 Hz, 2H), 6.84 (d, *J* = 8.7 Hz, 2H), 5.63 (d, *J* = 5.7 Hz, 1H), 3.82 (s, 3H), 3.81 (s, 3H), 2.26 (d, *J* = 5.7 Hz, 1H)

^{13}C NMR (101 MHz, CDCl$_3$, 298 K) δ 159.9, 159.8, 133.3, 133.3, 128.3, 114.7, 114.1, 114.1, 87.7, 86.6, 64.9, 55.5, 55.4

1,3-Bis(4-fluorophenyl)prop-2-yn-1-ol (1e)[3]

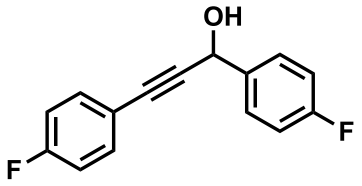

Prepared according to the general procedure using 4-fluorophenylacetylene (600 mg, 5.00 mmol) and 4-fluorobenzaldehyde (620 mg, 5.00 mmol). The crude product was purified by flash column chromatography on silica gel (pentane/EtOAc = 5:1) to afford **1e** as a yellow oil (1.16 g, 95%).

Formula: $C_{15}H_{10}OF_2$

Molecular weight: 244.24

Isolated yield: 95% (1.16 g)

Rf: 0.4 (Pentane/EtOAc = 5:1)

^{1}H NMR (400 MHz, CDCl$_3$, 298 K) δ 7.62 – 7.52 (m, 2H), 7.49 – 7.39 (m, 2H), 7.13 – 6.96 (m, 4H), 5.65 (s, 1H), 2.64 (s, 1H).

^{13}C NMR (101 MHz, CDCl$_3$, 298 K) δ 162.8 (d, *J* = 250.1 Hz), 162.8 (d, *J* = 247.3 Hz), 136.5 (d, *J* = 3.1 Hz), 133.8 (d, *J* = 8.5 Hz), 128.7 (d, *J* = 8.3 Hz), 118.4 (d, *J* = 3.5 Hz), 115.8 (d, *J* = 22.1 Hz), 115.6 (d, *J* = 21.5 Hz), 88.3, 85.9, 64.5

^{19}F NMR (376 MHz, CDCl$_3$, 298 K) δ -110.1, -113.5

1,3-Bis(3-fluorophenyl)prop-2-yn-1-ol (1f)

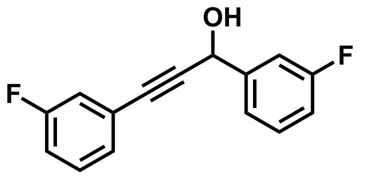

Prepared according to the general procedure using 3-fluorophenylacetylene (601 mg, 5.00 mmol) and 3-fluorobenzaldehyde (621 mg, 5.00 mmol). The crude product was purified by flash column chromatography on silica gel (pentane/EtOAc = 5:1) to afford **1f** as a colorless oil (1.05 g, 86%).

Formula: $C_{15}H_{10}OF_2$

Molecular weight: 244.24

Isolated yield: 86% (1.05 g)

Rf: 0.3 (Pentane/EtOAc = 5:1)

^{1}H NMR (400 MHz, CDCl$_3$, 298 K) δ 7.38 – 7.23 (m, 5H), 7.15 (d, *J* = 9.5 Hz, 1H), 7.10 – 6.98 (m, 2H), 5.67 (d, *J* = 6.2 Hz, 1H), 2.32 (d, *J* = 6.2 Hz, 1H)

^{13}C NMR (101 MHz, CDCl$_3$, 298 K) δ 163.0 (d, *J* = 247.6 Hz), 162.4 (d, *J* = 247.8 Hz), 142.9 (d, *J* = 6.9 Hz), 130.4 (d, *J* = 8.1 Hz), 130.1 (d, *J* = 8.6 Hz), 127.8 (d, *J* = 3.1 Hz), 124.0 (d, *J* = 9.5 Hz), 122.4 (d, *J* = 3.0 Hz), 118.7 (d, *J* = 22.9 Hz), 116.3 (d, *J* = 21.2 Hz), 115.6 (d, *J* = 21.2 Hz), 113.8 (d, *J* = 22.6 Hz), 89.1, 85.8 (d, *J* = 3.3 Hz), 64.5 (d, *J* = 2.0 Hz)

^{19}F NMR (376 MHz, CDCl$_3$, 298 K) δ -112.3, -112.7

HRMS (GC-EI): Calcd. for $C_{15}H_{10}OF_2^+$ $[M]^+$: 244.06842; Found 244.06957

1,3-Bis(2-fluorophenyl)prop-2-yn-1-ol (1g)

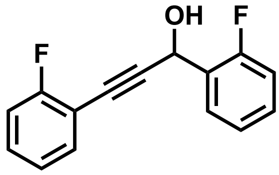

Prepared according to the general procedure using 2-fluorophenylacetylene (601 mg, 5.00 mmol) and 2-fluorobenzaldehyde (621 mg, 5.00 mmol). The crude product was purified by flash column chromatography on silica gel (pentane/EtOAc = 5:1) to afford **1g** as a colorless oil (1.03 g, 84%).

Formula: $C_{15}H_{10}OF_2$

Molecular weight: 244.24

Isolated yield: 84% (1.03 g)

Rf: 0.3 (Pentane/EtOAc = 5:1)

^{1}H NMR (400 MHz, CDCl$_3$, 298 K) δ 7.77 (t, *J* = 7.6 Hz, 1H), 7.46 (t, *J* = 7.4 Hz, 1H), 7.38 – 7.28 (m, 2H), 7.20 (t, *J* = 7.5 Hz, 1H), 7.14 – 7.03 (m, 3H), 6.00 (d, *J* = 6.0 Hz, 1H), 2.60 (d, *J* = 6.0 Hz, 1H)

^{13}C NMR (101 MHz, CDCl$_3$, 298 K) δ 163.0 (d, *J* = 265.1 Hz), 160.5 (d, *J* = 261.6 Hz), 133.8 (d, *J* = 0.9 Hz), 130.6 (d, *J* = 8.0 Hz), 130.5 (d, *J* = 8.3 Hz), 128.7 (d, *J* = 3.4 Hz), 127.6 (d, *J* = 13.1 Hz),

124.6 (d, J = 3.6 Hz), 124.1 (d, J = 3.8 Hz), 115.8 (d, J = 21.3 Hz), 115.7, (d, J =20.9 Hz), 111.0 (d, J = 15.6 Hz), 92.8 (d, J = 3.4 Hz), 80.2, 59.7 (d, J = 5.1 Hz)

^{19}F NMR (376 MHz, CDCl$_3$, 298 K) δ -109.7, -119.1

HRMS (GC-EI): Calcd. for $C_{15}H_{10}OF_2^+$ $[M]^+$: 244.06842; Found 244.06957

1,3-Bis(4-chlorophenyl)prop-2-yn-1-ol (1h)[2]

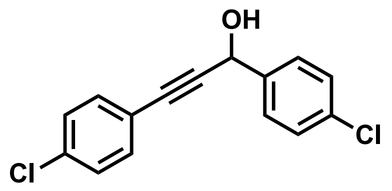

Prepared according to the general procedure using 4-chlorophenylacetylene (682 mg, 5.00 mmol) and 4-chlorobenzaldehyde (703 mg, 5.00 mmol). The crude product was purified by flash column chromatography on silica gel (pentane/EtOAc = 10:1) to afford **1h** as a white solid (0.85 g, 61%).

Formula: $C_{15}H_{10}OCl_2$

Molecular weight: 277.14

Isolated yield: 61% (0.85 g)

Rf: 0.4 (Pentane/EtOAc = 10:1)

^{1}H NMR (400 MHz, CDCl$_3$, 298 K) δ 7.54 (d, J = 8.4 Hz, 2H), 7.42 – 7.35 (m, 4H), 7.30 (d, J = 8.4 Hz, 2H), 5.66 (s, 1H)

^{13}C NMR (101 MHz, CDCl$_3$, 298 K) δ 139.0, 135.1, 134.5, 133.1, 129.0, 128.9, 128.2, 120.7, 89.3, 86.0, 64.5

1,3-Di([1,1'-biphenyl]-4-yl)prop-2-yn-1-ol (1i)

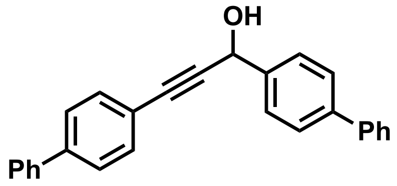

Prepared according to the general procedure using 4-ethynylbiphenyl (891 mg, 5.00 mmol) and biphenyl-4-carboxaldehyde (911 mg, 5.00 mmol). The crude product was purified by flash column chromatography on silica gel (pentane/EtOAc = 6:1) to afford **1i** as a white solid (1.50 g, 83%).

Formula: $C_{27}H_{20}O$

Molecular weight: 360.46

Isolated yield: 83% (1.5 g)

Rf: 0.4 (Pentane/EtOAc = 6:1)

^{1}H NMR (400 MHz, CDCl$_3$, 298 K) δ 7.72 (d, J = 8.2 Hz, 2H), 7.69 – 7.53 (m, 10H), 7.49 – 7.42 (m, 4H), 7.37 (t, J = 7.3 Hz, 2H), 5.77 (s, 1H)

^{13}C NMR (101 MHz, CDCl$_3$, 298 K) δ 141.6, 141.6, 140.8, 140.4, 139.8, 132.4, 129.0, 129.0, 127.9, 127.6, 127.4, 127.3, 127.2, 127.2, 121.4, 89.4, 86.8, 65.1

HRMS (ESI): Calcd. for $C_{27}H_{20}ONa^+$ $[M+Na]^+$: 383.14064, found 383.14032

1,3-Di(naphthalen-2-yl)prop-2-yn-1-ol (1j)

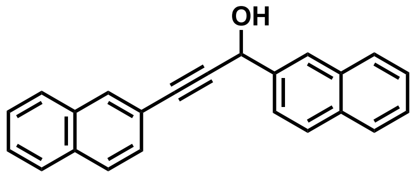

Prepared according to the general procedure using 2-ethynylnaphthalene (761 mg, 5.00 mmol) and 2-naphthaldehyde (781 mg, 5.00 mmol). The crude product was purified by flash column chromatography on silica gel (pentane/EtOAc = 5:1) to afford **1j** as a white solid (1.39 g, 90%).

Formula: $C_{23}H_{16}O$

Molecular weight: 308.38

Isolated yield: 90% (1.39 g)

Rf: 0.3 (Pentane/EtOAc = 5:1)

^{1}H NMR (400 MHz, CDCl$_3$, 298 K) δ 8.10 (s, 1H), 8.03 (s, 1H), 7.95 – 7.75 (m, 7H), 7.60 – 7.45 (m, 5H), 5.92 (d, *J* = 6.1 Hz, 1H), 2.44 (d, *J* = 6.1 Hz, 1H)

^{13}C NMR (101 MHz, CDCl$_3$, 298 K) δ 138.1, 133.4, 133.3, 133.0, 132.9, 131.9, 128.7, 128.5, 128.4, 128.1, 127.9, 127.8, 127.8, 126.9, 126.7, 126.4, 126.4, 125.6, 124.8, 119.8, 89.2, 87.3, 65.4

HRMS (GC-EI): Calcd. for $C_{23}H_{16}O^+$ $[M]^+$: 308.11970; Found 308.11975

1,3-Bis(4-(trifluoromethyl)phenyl)prop-2-yn-1-ol (1k)

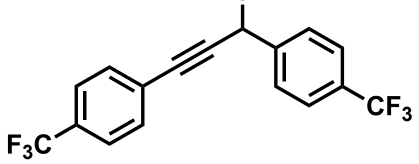

Prepared according to the general procedure using 1-ethynyl-4-(trifluoromethyl)benzene (851 mg, 5.00 mmol) and 4-(trifluoromethyl)benzaldehyde (871 mg, 5.00 mmol). The crude product was purified by flash column chromatography on silica gel (pentane/EtOAc = 5:1) to afford **1k** as a colorless oil (1.51 g, 88%).

Formula: $C_{17}H_{10}OF_6$

Molecular weight: 344.26

Isolated yield: 88% (1.51 g)

Rf: 0.3 (Pentane/EtOAc = 5:1)

^{1}H NMR (400 MHz, CDCl$_3$, 298 K) δ 7.72 (d, *J* = 8.3 Hz, 2H), 7.67 (d, *J* = 8.3 Hz, 2H), 7.62 – 7.53 (m, 4H), 5.77 (d, *J* = 5.2 Hz, 1H), 2.69 (d, J = 5.2 Hz, 1H)

^{13}C NMR (101 MHz, CDCl$_3$, 298 K) δ 144.0, 144.0, 132.2, 130.9 (q, *J* = 32.6 Hz), 130.8 (q, *J* = 32.9 Hz), 127.1, 125.9 (q, *J* = 3.8 Hz), 125.5 (q, *J* = 3.8 Hz), 124.1 (q, *J* = 270.5 Hz), 123.9 (q, *J* = 273.3 Hz), 90.4, 85.9, 64.5

^{19}F NMR (376 MHz, CDCl$_3$, 298 K) δ -62.6, -62.9.

HRMS (GC-EI): Calcd. for $C_{17}H_{10}OF_6^+$ $[M]^+$: 344.06304; Found 344.06355

3-Phenyl-1-(*p*-tolyl)prop-2-yn-1-ol (1m)[4]

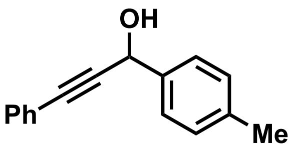

Prepared according to the general procedure using phenylacetylene (510 mg, 5.00 mmol) and 4-methylbenzaldehyde (600 mg, 5.00 mmol). The crude product was purified by flash column chromatography on silica gel (pentane/EtOAc = 5:1) to afford **1m** as a colorless oil (850 mg, 77%).

Formula: $C_{16}H_{14}O$

Molecular weight: 222.29

Isolated yield: 77% (0.85 g)

Rf: 0.4 (Pentane/EtOAc = 5:1)

^{1}H NMR (400 MHz, CDCl$_3$, 298 K) δ 7.56 – 7.46 (m, 4H), 7.37 – 7.29 (m, 3H), 7.23 (d, *J* = 7.9 Hz, 2H), 5.67 (d, *J* = 5.9 Hz, 1H), 2.45 (d, *J* = 5.9 Hz, 1H), 2.39 (s, 3H)

^{13}C NMR (101 MHz, CDCl$_3$, 298 K) δ 138.4, 137.9, 131.9, 129.4, 128.7, 128.4, 126.8, 122.6, 89.0, 86.6, 65.1, 21.3

1-(4-Methoxyphenyl)-3-phenylprop-2-yn-1-ol (1n)[4]

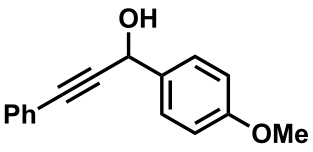

Prepared according to the general procedure using phenylacetylene (510 mg, 5.00 mmol) and 4-methoxybenzaldehyde (680 mg, 5.00 mmol). The crude product was purified by flash column chromatography on silica gel (pentane/EtOAc = 5:1) to afford **1n** as a colorless oil (1.13 g, 95%).

Formula: $C_{16}H_{14}O_2$

Molecular weight: 238.29

Isolated yield: 95% (1.13 g)

Rf: 0.3 (Pentane/EtOAc = 5:1)

^{1}H NMR (400 MHz, CDCl$_3$, 298 K) δ 7.55 (d, *J* = 8.7 Hz, 2H), 7.51 – 7.43 (m, 2H), 7.36 – 7.29 (m, 3H), 6.93 (d, *J* = 8.7 Hz, 2H), 5.65 (d, *J* = 6.1 Hz, 1H), 3.82 (s, 3H), 2.33 (d, *J* = 6.1 Hz, 1H)

^{13}C NMR (101 MHz, CDCl$_3$, 298 K) δ 159.8, 133.1, 131.9, 128.7, 128.4, 128.3, 122.6, 114.1, 89.0, 86.6, 64.9, 55.5

4-(1-Hydroxy-3-phenylprop-2-yn-1-yl)phenol (1o)[5]

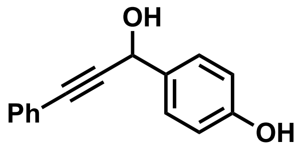

Prepared according to a modified general procedure using phenylacetylene (510 mg, 5.00 mmol), *n*-BuLi (1.6 M in hexane, 3.5 mL, 5.60 mmol), and 4-hydroxybenzaldehyde (305 mg, 2.50 mmol). The crude product was purified by flash column chromatography on silica gel (pentane/EtOAc = 10:1) to afford **1o** as a white solid (477 mg, 85%).

Formula: $C_{15}H_{12}O_2$

Molecular weight: 224.26

Isolated yield: 85% (477 mg)

Rf: 0.4 (Pentane/EtOAc = 10:1)

^{1}H NMR (400 MHz, CD_3OD, 298 K) δ 7.47 – 7.38 (m, 4H), 7.37 – 7.28 (m, 3H), 6.80 (d, *J* = 8.6 Hz, 2H), 5.53 (s, 1H)

^{13}C NMR (101 MHz, CD_3OD, 298 K) δ 158.6, 133.7, 132.6, 129.5, 129.5, 129.4, 124.3, 116.1, 90.8, 86.3, 65.1

1-(2,3,4,5,6-Pentamethylphenyl)-3-phenylprop-2-yn-1-ol (1p)

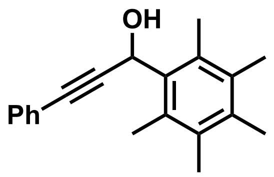

Prepared according to the general procedure using phenylacetylene (510 mg, 5.00 mmol) and pentamethylbenzaldehyde (881 mg, 5.00 mmol). The crude product was purified by flash column chromatography on silica gel (pentane/EtOAc = 5:1) to afford **1p** as a colorless oil (1.32 g, 95% yield).

Formula: $C_{20}H_{22}O$

Molecular weight: 278.39

Isolated yield: 95% (1.32 g)

Rf: 0.4 (Pentane/EtOAc = 5:1)

^{1}H NMR (400 MHz, $CDCl_3$, 298 K) δ 7.47 – 7.37 (m, 2H), 7.34 – 7.26 (m, 3H), 6.28 (d, *J* = 3.9 Hz, 1H), 2.53 (s, 6H), 2.26 (s, 3H), 2.25 (s, 6H), 2.13 (d, *J* = 3.9 Hz, 1H)

^{13}C NMR (101 MHz, $CDCl_3$, 298 K) δ 135.6, 134.6, 133.7, 132.4, 131.8, 128.4, 128.3, 123.1, 89.7, 85.8, 61.7, 17.4, 17.3, 16.9

HRMS (ESI$^+$): Calcd. for $C_{20}H_{22}ONa^+$ $[M + Na]^+$: 301.15629; Found 301.15598

1-(4-Fluorophenyl)-3-phenylprop-2-yn-1-ol (1q)[1]

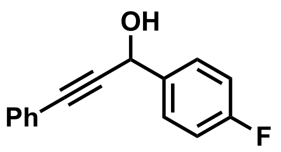

Prepared according to the general procedure using phenylacetylene (510 mg, 5.00 mmol) and 4-fluorobenzaldehyde (620 mg, 5.00 mmol). The crude product was purified by flash column chromatography on silica gel (pentane/EtOAc = 5:1) to afford **1q** as a colorless oil (1.13 g, >99%).

Formula:$C_{15}H_{11}FO$

Molecular weight: 226.25

Isolated yield: >99% (1.13g)

Rf: 0.4 (Pentane/EtOAc = 5:1)

^{1}H NMR (400 MHz, $CDCl_3$, 298 K) δ 7.59 (m, 2H), 7.48 (m, 2H), 7.40 – 7.29 (m, 3H), 7.08 (t, J = 8.7 Hz, 2H), 5.67 (s, 1H), 2.78 (s, 1H)

^{13}C NMR (101 MHz, $CDCl_3$, 298 K) δ 162.8 (d, J = 246.8 Hz), 136.6 (d, J = 3.2 Hz), 131.8, 128.8, 128.7 (d, J = 8.3 Hz), 128.5, 122.3, 115.6 (d, J = 21.6 Hz), 88.6, 86.9, 64.4

^{19}F NMR (376 MHz, $CDCl_3$, 298 K) δ -113.7

1-(4-Chlorophenyl)-3-phenylprop-2-yn-1-ol (1r)[1]

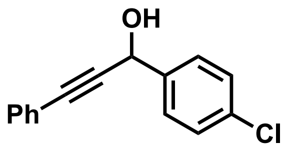

Prepared according to the general procedure using phenylacetylene (510 mg, 5.00 mmol) and 4-chlorobenzaldehyde (703 mg, 5.00 mmol). The crude product was purified by flash column chromatography on silica gel (pentane/EtOAc = 5:1) to afford **1r** as a yellow oil (1.14 g, 94%).

Formula:$C_{15}H_{11}OCl$

Molecular weight: 242.70

Isolated yield: 94% (1.14 g)

Rf: 0.4 (Pentane/EtOAc = 5:1)

^{1}H NMR (400 MHz, $CDCl_3$, 298 K) δ 7.55 (d, J = 8.4 Hz, 2H), 7.51 – 7.43 (m, 2H), 7.39 – 7.28 (m, 5H), 5.67 (d, J = 6.0 Hz, 1H), 2.42 (d, J = 6.0 Hz, 1H)

^{13}C NMR (101 MHz, $CDCl_3$, 298 K) δ 139.2, 134.4, 131.9, 128.9, 128.5, 128.3, 127.4, 122.3, 88.3, 87.1, 64.5

3-Phenyl-1-(4-(trifluoromethyl)phenyl)prop-2-yn-1-ol (1s)[6]

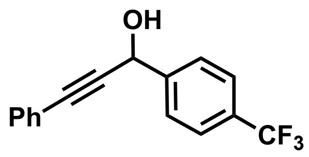

Prepared according to the general procedure using phenylacetylene (510 mg, 5.00 mmol) and 4-(trifluoromethyl)benzaldehyde (871 mg, 5.00 mmol). The crude product was purified by flash column chromatography on silica gel (pentane/EtOAc = 5:1) to afford **1s** as a yellow oil (1.28 g, 93%).

Formula: $C_{16}H_{11}OF_3$

Molecular weight: 276.26

Isolated yield: 93% (1.28 g)

Rf: 0.3 (Pentane/EtOAc = 5:1)

^{1}H NMR (400 MHz, $CDCl_3$, 298 K) δ 7.74 (d, J = 8.2 Hz, 2H), 7.66 (d, J = 8.2 Hz, 2H), 7.49 – 7.45 (m, 2H), 7.37 – 7.29 (m, 3H), 5.76 (s, 1H), 2.60 (s, 1H)

^{13}C NMR (101 MHz, $CDCl_3$, 298 K) δ 144.4, 131.9, 130.6 (q, J = 32.6 Hz), 129.0, 128.5, 127.1, 125.7 (q, J = 3.7 Hz), 124.2 (q, J = 270.5 Hz), 122.1, 88.1, 87.3, 64.4

^{19}F NMR (376 MHz, $CDCl_3$, 298 K) δ -62.5

Methyl 4-(1-hydroxy-3-phenylprop-2-yn-1-yl)benzoate (1t)[7]

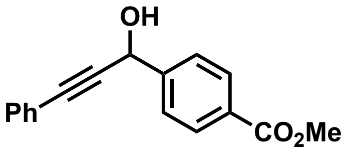

Prepared according to the general procedure using phenylacetylene (510 mg, 5.00 mmol) and methyl 4-formylbenzoate (820 mg, 5.00 mmol). The crude product was purified by flash column chromatography on silica gel (pentane/EtOAc = 4:1) to afford **1t** as a white solid (1.01 g, 76%). **Caution**: This compound should be stored under argon to prevent degradation.

Formula: $C_{17}H_{14}O_3$

Molecular weight: 266.30

Isolated yield: 76% (1.01 g)

Rf: 0.4 (Pentane/EtOAc = 4:1)

^{1}H NMR (400 MHz, $CDCl_3$, 298 K) δ 7.99 (d, J = 8.2 Hz, 2H), 7.62 (d, J = 8.2 Hz, 2H), 7.45 – 7.35 (m, 2H), 7.35 – 7.20 (m, 3H), 5.69 (d, J = 6.1 Hz, 1H), 3.86 (s, 3H), 3.30 (d, J = 6.1 Hz, 1H)

^{13}C NMR (101 MHz, $CDCl_3$, 298 K) δ 167.0, 145.7, 131.8, 130.0, 129.9, 128.8, 128.4, 126.7, 122.2, 88.3, 87.0, 64.5, 52.3

N-(4-(1-Hydroxy-3-phenylprop-2-yn-1-yl)phenyl)acetamide (1u)[5]

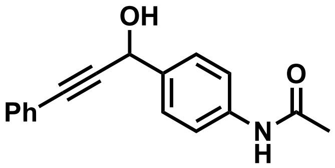

Prepared according to a modified general procedure using phenylacetylene (612 mg, 6.00 mmol), *n*-BuLi (1.6 M in hexane, 3.75 mL), and N-(4-formylphenyl)acetamide (489.5 mg, 3.00 mmol). The crude product was purified by flash column chromatography on silica gel (pentane/EtOAc = 1:4) to afford **1u** as a white solid (0.72 g, 90%).

Formula: $C_{17}H_{15}NO_2$

Molecular weight: 265.31

Isolated yield: 90% (0.72 g)

Rf: 0.3 (Pentane/EtOAc = 1:4)

^{1}H NMR (400 MHz, $CDCl_3$, 298 K) δ 7.64 – 7.50 (m, 4H), 7.50 – 7.43 (m, 2H), 7.38 – 7.29 (m, 3H), 7.23 (s, 1H), 5.66 (d, J = 6.0 Hz, 1H), 2.30 (d, J = 6.0 Hz, 1H), 2.19 (s, 3H)

^{13}C NMR (101 MHz, $CDCl_3$, 298 K) δ 169.7, 138.0, 136.7, 131.7, 128.6, 128.4, 127.5, 122.4, 120.4, 89.0, 86.5, 64.5, 24.3

1-(Naphthalen-2-yl)-3-phenylprop-2-yn-1-ol (1v)[4]

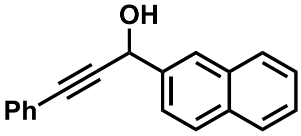

Prepared according to the general procedure using phenylacetylene (510 mg, 5.00 mmol) and 2-naphthaldehyde (781 mg, 5.00 mmol). The crude product was purified by flash column chromatography on silica gel (pentane/EtOAc = 3:1) to afford **1v** as a white solid (1.29 g, >99%).

Formula: $C_{19}H_{14}O$

Molecular weight: 258.32

Isolated yield: >99% (1.29 g)

Rf: 0.3 (Pentane/EtOAc = 3:1)

^{1}H NMR (400 MHz, $CDCl_3$, 298 K) δ 8.06 (s, 1H), 7.92 – 7.82 (m, 3H), 7.74 (dd, J = 8.5, 1.7 Hz, 1H), 7.54 – 7.45 (m, 4H), 7.42 – 7.29 (m, 3H), 5.87 (d, J = 6.0 Hz, 1H), 2.40 (d, J = 6.0 Hz, 1H)

^{13}C NMR (101 MHz, $CDCl_3$, 298 K) δ 138.1, 133.4, 133.3, 131.9, 128.8, 128.8, 128.5, 128.4, 127.8, 126.5, 126.5, 125.7, 124.8, 122.5, 88.8, 87.1, 65.4

3-Phenyl-1-(thiophen-2-yl)prop-2-yn-1-ol (1w)[4]

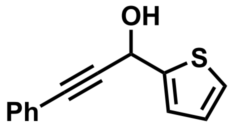

Prepared according to the general procedure using phenylacetylene (510 mg, 5.00 mmol) and thiophene-2-carbaldehyde (561 mg, 5.00 mmol). The crude product was purified by flash column chromatography on silica gel (pentane/EtOAc = 5:1) to afford **1w** as an orange oil (0.90 g, 84%).

Formula: $C_{13}H_{10}OS$

Molecular weight: 214.28

Isolated yield: 84% (0.9 g)

Rf: 0.3 (Pentane/EtOAc = 5:1)

^{1}H NMR (400 MHz, $CDCl_3$, 298 K) δ 7.49 – 7.43 (m, 2H), 7.33 – 7.25 (m, 4H), 7.23 – 7.18 (m, 1H), 6.96 (dd, J = 5.1, 3.5 Hz, 1H), 5.85 (d, J = 5.4 Hz, 1H), 2.79 (d, J = 5.9 Hz, 1H)

^{13}C NMR (101 MHz, $CDCl_3$, 298 K) δ 144.7, 131.9, 128.9, 128.4, 126.9, 126.2, 125.8, 122.2, 88.2, 86.1, 60.8

1-Cyclohexyl-3-phenylprop-2-yn-1-ol (1x)[4]

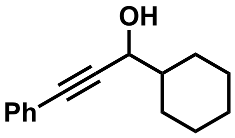

Prepared according to the general procedure using phenylacetylene (510 mg, 5.00 mmol) and cyclohexanecarbaldehyde (261 mg, 5.00 mmol). The crude product was purified by flash column chromatography on silica gel (pentane/EtOAc) to afford **1x** as a colorless oil (0.93 g, 87%).

Formula: $C_{15}H_{18}O$

Molecular weight: 214.31

Isolated yield: 87% (0.93 g)

^{1}H NMR (400 MHz, $CDCl_3$, 298 K) δ 7.49 – 7.39 (m, 2H), 7.36 – 7.27 (m, 3H), 4.38 (d, J = 6.0 Hz, 1H), 2.06 – 1.88 (m, 3H), 1.83 – 1.76 (m, 2H), 1.71 – 1.60 (m, 2H), 1.30 – 1.11 (m, 5H)

^{13}C NMR (101 MHz, $CDCl_3$, 298 K) δ 131.8, 128.4, 128.4, 122.9, 89.4, 85.8, 67.8, 44.4, 28.8, 28.3, 26.5, 26.0, 26.0

3-Cyclohexyl-1-phenylprop-2-yn-1-ol (1y)[8]

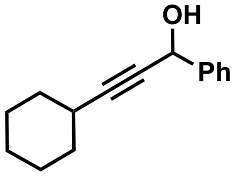

Prepared according to the general procedure using ethynylcyclohexane (540 mg, 5.00 mmol) and benzaldehyde (530 mg, 5.00 mmol). The crude product was purified by flash column chromatography on silica gel (pentane/EtOAc = 10:1) to afford **1y** as a colorless oil (0.99 g, 92%).

Formula: $C_{15}H_{18}O$

Molecular weight: 214.31

Isolated yield: 92% (0.99 g)

Rf: 0.3 (Pentane/EtOAc = 10:1)

^{1}H NMR (400 MHz, CDCl$_3$, 298 K) δ 7.61 – 7.52 (m, 2H), 7.41 – 7.31 (m, 3H), 5.47 (d, *J* = 5.5 Hz, 1H), 2.52 – 2.40 (m, 1H), 2.12 (d, *J* = 5.5 Hz, 1H), 1.87 – 1.80 (m, 2H), 1.76 – 1.67 (m, 2H), 1.55 – 1.42 (m, 3H), 1.35 – 1.27 (m, 3H)

^{13}C NMR (101 MHz, CDCl$_3$, 298 K) δ 141.4, 128.7, 128.3, 126.8, 91.9, 79.9, 65.0, 32.7, 29.3, 26.0, 25.0

2.2 Preparation of Propargylic Alcohols 1l

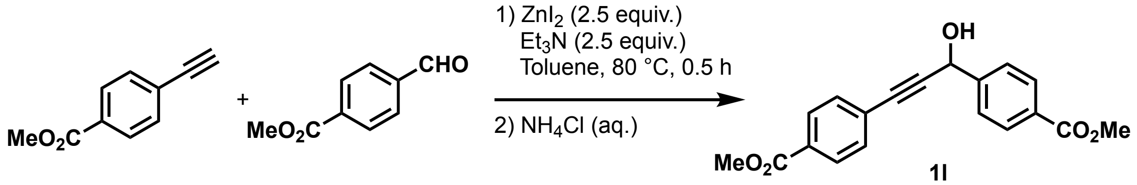

In an oven-dried Schlenk tube equipped with a magnetic stir bar, methyl 4-ethynylbenzoate (160.7 mg, 1.00 mmol), methyl 4-formylbenzoate (164.1 mg, 1.00 mmol), and zinc iodide (798 mg, 2.50 mmol) were added sequentially. The tube was sealed and purged with argon. Triethylamine (253 mg, 2.50 mmol) and toluene (5 mL) were then added *via* syringe. The reaction mixture was stirred at 80 °C for 30 min. After cooling to room temperature, the reaction was quenched with aqueous NH_4Cl and extracted with EtOAc (3 × 10 mL). The combined organic layers were dried over anhydrous $MgSO_4$, filtered, and concentrated under reduced pressure. The crude residue was purified by flash column chromatography on silica gel (pentane/EtOAc = 3:2) to afford **1l** as a white solid (312 mg, 96%).

Dimethyl 4,4'-(3-hydroxyprop-1-yne-1,3-diyl)dibenzoate (1l)

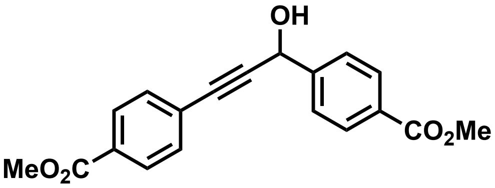

Formula: $C_{19}H_{16}O_5$

Molecular weight: 324.33

Isolated yield: 96% (312 mg)

Rf: 0.4 (Pentane/EtOAc = 3:2)

^{1}H NMR (400 MHz, $CDCl_3$, 298 K) δ 8.07 (d, *J* = 8.3 Hz, 2H), 7.98 (d, *J* = 8.3 Hz, 2H), 7.67 (d, *J* = 8.3 Hz, 2H), 7.50 (d, *J* = 8.3 Hz, 2H), 5.76 (d, *J* = 4.3 Hz, 1H), 3.92 (s, 3H), 3.91 (s, 3H), 2.67 (d, *J* = 4.3 Hz, 1H)

^{13}C NMR (101 MHz, $CDCl_3$, 298 K) δ 166.9, 166.6, 145.1, 131.8, 130.3, 130.2, 129.6, 126.9, 126.7, 91.1, 86.3, 64.7, 52.5, 52.4

HRMS (ESI$^+$): Calcd. for $C_{19}H_{16}O_5Na^+$ $[M + Na]^+$: 347.08899; Found 347.08875

3. Optimization of Reaction Parameters

3.1 Optimization of Reaction Conditions for 2a

Table S1 – Optimization of reaction conditions for compound **1a** in dioxane.

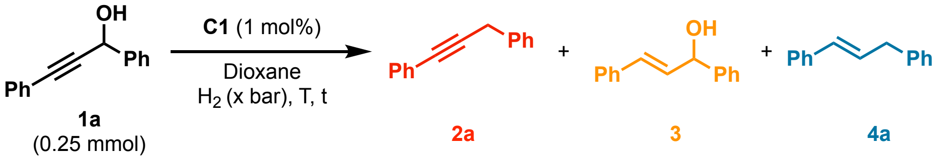

Entry	H_2 (bar)	T (°C) / t (h)	Yield of 2a (%)[a]	Yield of 3 (%)[a]	Yield of 4a (%)[a]
1	2	80 / 5	60	07	09
2	2	60 / 5	62	02	10
3	2	40 / 5	30	00	03
4	1	60 / 5	67	00	09
5	2	60 / 3	57	00	07
6	2	70 / 3	62	03	08
7	1	70 / 3	76	03	07

[a]Yields determined by ^{1}H NMR using mesitylene (0.25 mmol) as an internal standard.

3.2 Optimization of Reaction Conditions for 3a

Table S2 – Optimization of reaction conditions for compound **3a** in methanol.

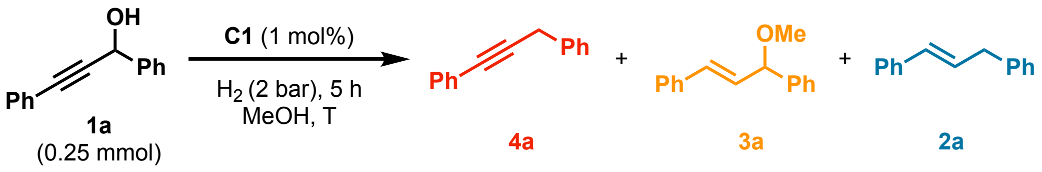

Entry	T (°C)	Yield of 2a (%)[a]	Yield of 3a (%)[a]	Yield of 4a (%)[a]
1	80	00	74	26
2	60	00	83	16
3	40	00	90	02

[a]Yields determined by ^1H NMR using mesitylene (0.25 mmol) as an internal standard.

3.3 Optimization of Reaction Conditions for 4a

Table S3 – Solvent screening for compound **2a**.

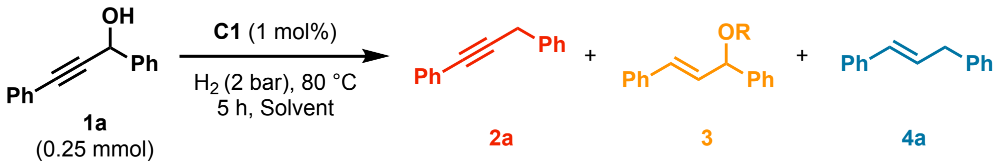

Entry	Solvent	R	Yield of 2a (%)[a]	Yield of 3 (%)[a]	Yield of 4a (%)[a]
1	Toluene	-	00	00	00
2	MeCN	-	00	00	00
3	*t*-AmylOH	-	00	00	89
4	MeOH	Me	00	74	26
5	*i*-PrOH	-	00	00	92
6	THF	H	60	06	12
7	Dioxane	H	65	07	09
8	2-MeTHF	H	46	04	16

[a]Yields determined by ^1H NMR using mesitylene (0.25 mmol) as an internal standard.

4. Asymmetric Substrate Scope Under *Conditions B* and *C*

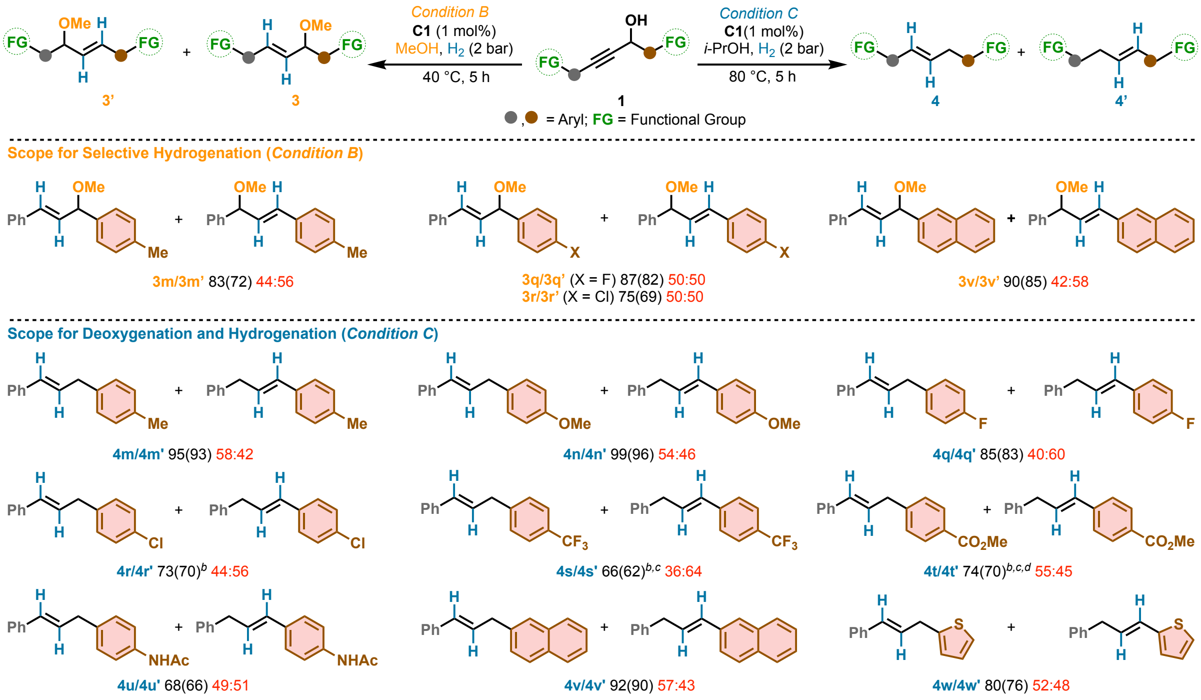

Scheme S1 – Scope of asymmetric substrates under *Condition B* and *Condition C*. [a]*Condition B*: **1** (0.5 mmol, 1 equiv), **C1** (1 mol%), MeOH (2 mL), H_2 (2 bar), 40 °C, 5 h. *Condition C*: **1** (0.5 mmol, 1 equiv), **C1** (1 mol%), *i*-PrOH (2 mL), H_2 (2 bar), 80 °C, 5 h. Yields determined by ^{1}H NMR using mesitylene (0.5 mmol) as an internal standard; isolated yields in parentheses; regioisomeric ratios in red. [b]Reaction performed at 100 °C. [c]**C1** used at 3 mol%. [d]Reaction performed on 0.25 mmol scale.

5. Preparation of Alkynes 2

5.1 General Procedure for the Preparation of Alkynes 2

In an oven-dried Fisher–Porter tube under an argon atmosphere, propargylic alcohol **1** (0.50 mmol), complex **C1** (6.7 mg, 1 mol%), and dioxane (2 mL) were added. The mixture was flash-frozen using liquid nitrogen, evacuated, and subsequently pressurized with H_2 (1 bar). The sealed tube was stirred at 70 °C for 3 h. After cooling to room temperature, the pressure was carefully released. The solvent was removed under reduced pressure. Conversion and yield were determined by ^{1}H NMR spectroscopy using mesitylene (0.50 mmol) as an internal standard. Product **2** was purified by flash column chromatography on silica gel using pentane/EtOAc as the eluent.

Prop-1-yne-1,3-diyldibenzene (2a)[9]

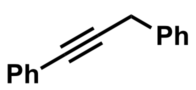

Prepared according to the general procedure using **1a** (104.1 mg, 0.50 mmol). The crude product was purified by flash column chromatography on silica gel (pentane/EtOAc = 50:1) to afford **2a** as a colorless oil (66 mg, 69% yield).

Formula: $C_{15}H_{12}$

Molecular weight: 192.26

Isolated yield: 69% (66 mg), colorless oil

Rf: 0.4 (Pentane/EtOAc = 50:1)

^{1}H NMR (400 MHz, CDCl$_3$, 298 K) δ 7.51 – 7.40 (m, 4H), 7.39 – 7.21 (m, 6H), 3.85 (s, 2H)

^{13}C NMR (101 MHz, CDCl$_3$, 298 K) δ 136.9, 131.8, 128.7, 128.4, 128.1, 128.0, 126.8, 123.8, 87.7, 82.8, 25.9

4,4'-(Prop-1-yne-1,3-diyl)bis(methylbenzene) (2b)[10]

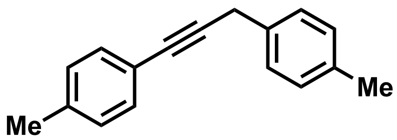

Prepared according to the general procedure using **1b** (118.2 mg, 0.50 mmol). The crude product was purified by flash column chromatography on silica gel (pentane/EtOAc = 50:1) to afford **2b** as a yellow oil (74 mg, 67% yield).

Formula: $C_{17}H_{16}$

Molecular weight: 220.32

Isolated yield: 67% (74 mg), yellow oil

Rf: 0.5 (Pentane/EtOAc = 50:1)

^{1}H NMR (400 MHz, CDCl$_3$, 298 K) δ 7.37 (d, J = 8.0 Hz, 2H), 7.33 (d, J = 8.0 Hz, 2H), 7.17 (d, J = 7.9 Hz, 2H), 7.13 (d, J = 7.9 Hz, 2H), 3.81 (s, 2H), 2.37 (s, 3H), 2.37 (s, 3H)

^{13}**C NMR (101 MHz, CDCl$_3$, 298 K)** δ 137.9, 136.3, 134.0, 131.6, 129.3, 129.1, 128.0, 120.8, 87.2, 82.6, 25.5, 21.6, 21.2

4,4'-(Prop-1-yne-1,3-diyl)bis(*tert*-butylbenzene) (2c)

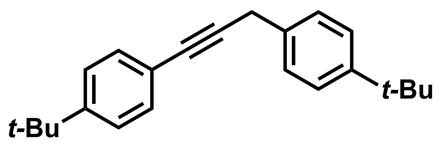

Prepared according to the general procedure using **1c** (160.2 mg, 0.50 mmol). The crude product was purified by flash column chromatography on silica gel (pentane/EtOAc = 50:1) to afford **2c** as a yellow oil (82 mg, 54% yield).

Formula: $C_{23}H_{28}$

Molecular weight: 304.48

Isolated yield: 54% (82 mg), yellow oil

Rf: 0.5 (Pentane/EtOAc = 50:1)

^{1}**H NMR (400 MHz, CDCl$_3$, 298 K)** δ 7.44 – 7.32 (m, 8H), 3.83 (s, 2H), 1.36 (s, 9H), 1.34 (s, 9H)

^{13}**C NMR (101 MHz, CDCl$_3$, 298 K)** δ 151.0, 149.6, 134.0, 131.5, 127.8, 125.6, 125.3, 120.9, 87.2, 82.6, 34.8, 34.6, 31.5, 31.3, 25.4

HRMS (GC-EI): Calcd. for $C_{23}H_{28}^+$ $[M]^+$: 304.21855; Found 304.21885

4,4'-(Prop-1-yne-1,3-diyl)bis(methoxybenzene) (2d)[11]

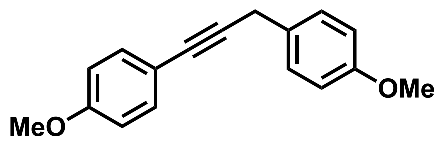

Prepared according to the general procedure using **1d** (134.2 mg, 0.50 mmol). The crude product was purified by flash column chromatography on silica gel (pentane/EtOAc = 25:1) to afford **2d** as a yellow oil (78 mg, 62% yield).

Formula: $C_{17}H_{16}O_2$

Molecular weight: 252.31

Isolated yield: 62% (78 mg), yellow oil

Rf: 0.4 (Pentane/EtOAc = 25:1)

^{1}**H NMR (400 MHz, CDCl$_3$, 298 K)** δ 7.40 (d, *J* = 8.6 Hz, 2H), 7.34 (d, *J* = 8.4 Hz, 2H), 6.89 (d, *J* = 8.4 Hz, 2H), 6.84 (d, *J* = 8.6 Hz, 2H), 3.81 (s, 6H), 3.77 (s, 2H)

^{13}**C NMR (101 MHz, CDCl$_3$, 298 K)** δ 159.3, 158.5, 133.1, 129.1, 129.0, 116.0, 114.0, 114.0, 86.5, 82.3, 55.4, 55.4, 25.0

4,4'-(Prop-1-yne-1,3-diyl)bis(fluorobenzene) (2e)

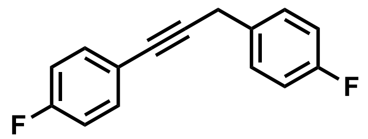

Prepared according to the general procedure using **1e** (122.2 mg, 0.50 mmol). The crude product was purified by flash column chromatography on silica gel (pentane/EtOAc = 50:1) to afford **2e** as a yellow oil (88 mg, 77% yield).

Formula: $C_{15}H_{10}F_2$

Molecular weight: 228.24

Isolated yield: 77% (88 mg), yellow oil

Rf: 0.4 (Pentane/EtOAc = 50:1)

^{1}H NMR (400 MHz, $CDCl_3$, 298 K) δ 7.52 – 7.31 (m, 4H), 7.09 – 6.97 (m, 4H), 3.79 (s, 2H)

^{13}C NMR (101 MHz, $CDCl_3$, 298 K) δ 162.4 (d, J = 248.8 Hz), 161.9 (d, J = 244.6 Hz), 133.6 (d, J = 8.3 Hz), 132.4 (d, J = 3.2 Hz), 129.5 (d, J = 8.0 Hz), 119.7 (d, J = 3.5 Hz), 115.6 (d, J = 22.0 Hz), 115.5 (d, J = 21.5 Hz), 87.1, 81.9, 25.1

^{19}F NMR (376 MHz, $CDCl_3$, 298 K) δ -111.6, -116.4

HRMS (GC-EI): Calcd. for $C_{15}H_{10}F_2^+$ $[M]^+$: 228.07451; Found 228.07445

4,4'-(Prop-1-yne-1,3-diyl)bis(chlorobenzene) (2h)

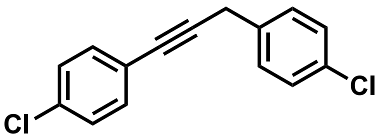

Prepared according to a modified general procedure using **1h** (138.6 mg, 0.50 mmol) at 80 °C. The crude product was purified by flash column chromatography on silica gel (pentane/EtOAc = 100:3) to afford **2h** as a yellow oil (69 mg, 53% yield).

Formula: $C_{15}H_{10}Cl_2$

Molecular weight: 261.15

Isolated yield: 53% (69 mg), yellow oil

Rf: 0.4 (Pentane/EtOAc = 100:3)

^{1}H NMR (400 MHz, $CDCl_3$, 298 K) δ 7.37 – 7.24 (m, 8H), 3.77 (s, 2H)

^{13}C NMR (101 MHz, $CDCl_3$, 298 K) δ 135.1, 134.1, 133.0, 132.7, 129.4, 128.8, 128.7, 122.0, 88.1, 82.0, 25.3

HRMS (GC-EI): Calcd. for $C_{15}H_{10}Cl_2^+$ $[M]^+$: 260.01541; Found 260.01558

4,4''-(Prop-1-yne-1,3-diyl)di-1,1'-biphenyl (2i)

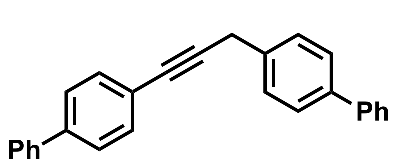

Prepared according to a modified general procedure using **1i** (90.1 mg, 0.25 mmol). The crude product was purified by flash column chromatography on silica gel (pentane/EtOAc = 25:1) to afford **2i** as a yellow solid (64 mg, 74% yield).

Formula: $C_{27}H_{20}$

Molecular weight: 344.46

Isolated yield: 74% (64 mg), yellow solid

Rf: 0.4 (Pentane/EtOAc = 25:1)

^{1}H NMR (400 MHz, CDCl$_3$, 298 K) δ 7.51 – 7.43 (m, 10H), 7.39 (d, *J* = 8.2 Hz, 2H), 7.35 – 7.30 (m, 4H), 7.26 – 7.21 (m, 2H), 3.78 (s, 2H)

^{13}C NMR (101 MHz, CDCl$_3$, 298 K) δ 141.0, 140.7, 140.5, 139.8, 135.9, 132.2, 129.0, 128.9, 128.5, 127.7, 127.4, 127.3, 127.2, 127.1, 127.1, 122.7, 88.3, 82.8, 25.6

HRMS (GC-EI): Calcd. for $C_{27}H_{20}^+$ $[M]^+$: 344.15595; Found 344.15654

2,2'-(Prop-1-yne-1,3-diyl)dinaphthalene (2j)

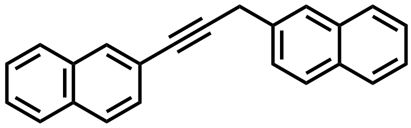

Prepared according to the general procedure using **1j** (154.2 mg, 0.50 mmol). The crude product was purified by flash column chromatography on silica gel (pentane/DCM = 4:1) to afford **2j** as an orange solid (60 mg, 41% yield).

Formula: $C_{23}H_{16}$

Molecular weight: 292.38

Isolated yield: 41% (60 mg), orange solid

Rf: 0.3 (Pentane/DCM = 4:1)

^{1}H NMR (400 MHz, CDCl$_3$, 298 K) δ 8.05 (s, 1H), 7.95 (s, 1H), 7.90 – 7.80 (m, 6H), 7.61 – 7.48 (m, 6H), 4.08 (s, 2H)

^{13}C NMR (101 MHz, CDCl$_3$, 298 K) δ 134.3, 133.7, 133.2, 132.8, 132.5, 131.5, 128.8, 128.4, 128.0, 127.9, 127.8, 127.8, 126.7, 126.6, 126.6, 126.4, 126.3, 125.7, 121.1, 88.0, 83.4, 26.2

HRMS (GC-EI): Calcd. for $C_{23}H_{16}^+$ $[M]^+$: 292.12465; Found 292.12490

1-Methyl-4-(3-phenylprop-2-yn-1-yl)benzene (2m)[9]

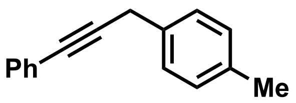

Prepared according to the general procedure using **1m** (111.1 mg, 0.50 mmol). The crude product was purified by flash column chromatography on silica gel (pentane/EtOAc = 50:1) to afford **2m** as a colorless oil (75 mg, 73% yield).

Formula: $C_{16}H_{14}$

Molecular weight: 206.29

Isolated yield: 73% (75 mg), colorless oil

Rf: 0.5 (Pentane/EtOAc = 50:1)

^{1}H NMR (400 MHz, $CDCl_3$, 298 K) δ 7.51 – 7.41 (m, 2H), 7.37 – 7.28 (m, 5H), 7.17 (d, J = 7.9 Hz, 2H), 3.81 (s, 2H), 2.36 (s, 3H)

^{13}C NMR (101 MHz, $CDCl_3$, 298 K) δ 136.3, 133.8, 131.8, 129.4, 128.4, 128.0, 127.9, 123.9, 88.0, 82.6, 25.5, 21.2

1-Methoxy-4-(3-phenylprop-2-yn-1-yl)benzene (2n)[9]

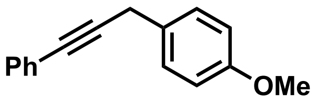

Prepared according to the general procedure using **1n** (119.2 mg, 0.50 mmol). The crude product was purified by flash column chromatography on silica gel (pentane/EtOAc = 50:1) to afford **2n** as a yellow oil (97 mg, 87% yield).

Formula: $C_{16}H_{14}O$

Molecular weight: 222.29

Isolated yield: 87% (97 mg), yellow oil

Rf: 0.4 (Pentane/EtOAc = 50:1)

^{1}H NMR (400 MHz, $CDCl_3$, 298 K) δ 7.46 – 7.36 (m, 2H), 7.31 – 7.20 (m, 5H), 6.84 (d, J = 8.7 Hz, 2H), 3.74 (s, 3H), 3.72 (s, 2H)

^{13}C NMR (101 MHz, $CDCl_3$, 298 K) δ 158.5, 131.7, 129.0, 128.9, 128.3, 127.9, 123.8, 114.1, 88.1, 82.5, 55.4, 25.0

4-(3-Phenylprop-2-yn-1-yl)phenol (2o)

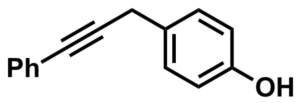

Prepared according to a modified general procedure using **1o** (112.1 mg, 0.50 mmol) and *i*-PrOH (2 mL) under 2 bar of H_2 at 60 °C for 5 h. The crude product was purified by flash column chromatography on silica gel (pentane/EtOAc = 5:1) to afford **2o** as a brown oil (62 mg, 60% yield).

Formula: $C_{15}H_{12}O$

Molecular weight: 208.26

Isolated yield: 60% (62 mg), brown oil

Rf: 0.4 (Pentane/EtOAc = 5:1)

^{1}H NMR (400 MHz, CDCl$_3$, 298 K) δ 7.47 (dd, *J* = 6.3, 2.8 Hz, 2H), 7.39 – 7.24 (m, 5H), 6.83 (d, *J* = 8.4 Hz, 2H), 5.23 (s, 1H), 3.78 (s, 2H)

^{13}C NMR (101 MHz, CDCl$_3$, 298 K) δ 154.3, 131.7, 129.3, 129.1, 128.4, 127.9, 123.8, 115.5, 88.1, 82.6, 25.0

HRMS (ESI$^+$): Calcd. for $C_{15}H_{11}O^-$ $[M-H]^-$: 207.08154; Found 207.08161

1,2,3,4,5-Pentamethyl-6-(3-phenylprop-2-yn-1-yl)benzene (2p)

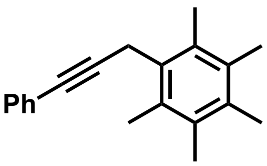

Prepared according to the general procedure using **1p** (139.2 mg, 0.50 mmol). The crude product was purified by flash column chromatography on silica gel (pentane/EtOAc = 50:1) to afford **2p** as a colorless oil (106 mg, 81% yield).

Formula: $C_{20}H_{22}$

Molecular weight: 262.396

Isolated yield: 81% (106 mg), colorless oil

Rf: 0.4 (Pentane/EtOAc = 50:1)

^{1}H NMR (400 MHz, CDCl$_3$, 298 K) δ 7.40 – 7.31 (m, 2H), 7.26 – 7.19 (m, 3H), 3.75 (s, 2H), 2.38 (s, 6H), 2.29 – 2.21 (m, 9H)

^{13}C NMR (101 MHz, CDCl$_3$, 298 K) δ 133.7, 132.8, 132.1, 131.7, 131.6, 128.2, 127.5, 124.2, 88.2, 80.5, 21.4, 17.1, 16.9, 16.8

HRMS (GC-EI): Calcd. for $C_{20}H_{22}^+$ $[M]^+$: 262.17160; Found 262.17196

1-Fluoro-4-(3-phenylprop-2-yn-1-yl)benzene (2q)[12]

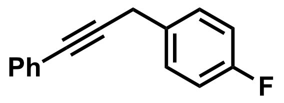

Prepared according to the general procedure using **1q** (113.1 mg, 0.50 mmol). The crude product was purified by flash column chromatography on silica gel (pentane/EtOAc = 50:1) to afford **2q** as a yellow oil (69 mg, 66% yield).

Formula: $C_{15}H_{11}F$

Molecular weight: 210.25

Isolated yield: 66% (69 mg), yellow oil

Rf: 0.5 (Pentane/EtOAc = 50:1)

^{1}H NMR (400 MHz, $CDCl_3$, 298 K) δ 7.51 – 7.42 (m, 2H), 7.42 – 7.35 (m, 2H), 7.35 – 7.28 (m, 3H), 7.04 (t, *J* = 8.7 Hz, 2H), 3.81 (s, 2H)

^{13}C NMR (101 MHz, $CDCl_3$, 298 K) δ 161.9 (d, *J* = 244.6 Hz), 132.5 (d, *J* = 3.2 Hz), 131.8, 129.5 (d, *J* = 8.0 Hz), 128.4, 128.1, 123.6, 115.5 (d, *J* = 21.4 Hz), 87.4, 83.0, 25.1

^{19}F NMR (376 MHz, $CDCl_3$, 298 K) δ -116.6

1-Chloro-4-(3-phenylprop-2-yn-1-yl)benzene (2r)[12]

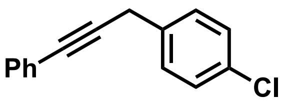

Prepared according to a modified general procedure using **1r** (121.4 mg, 0.50 mmol) at 80 °C. The crude product was purified by flash column chromatography on silica gel (pentane/EtOAc = 50:1) to afford **2r** as a yellow oil (57 mg, 50% yield).

Formula: $C_{15}H_{11}Cl$

Molecular weight: 226.70

Isolated yield: 50% (57 mg), yellow oil

Rf: 0.5 (Pentane/EtOAc = 50:1)

^{1}H NMR (400 MHz, $CDCl_3$, 298 K) δ 7.49 – 7.39 (m, 2H), 7.35 – 7.26 (m, 7H), 3.77 (s, 2H)

^{13}C NMR (101 MHz, $CDCl_3$, 298 K) δ 135.4, 132.6, 131.8, 129.4, 128.8, 128.4, 128.1, 123.5, 87.0, 83.1, 25.3

N-(4-(3-phenylprop-2-yn-1-yl)phenyl)acetamide (2u)[13]

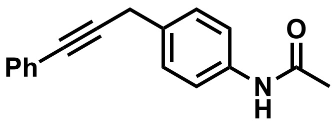

Prepared according to the general procedure using **1u** (132.7 mg, 0.50 mmol). The crude product was purified by flash column chromatography on silica gel (pentane/EtOAc = 1:3) to afford **2u** as a colorless oil (91 mg, 73% yield).

Formula: $C_{17}H_{15}NO$

Molecular weight: 249.31

Isolated yield: 73% (91 mg), colorless oil

Rf: 0.4 (Pentane/EtOAc = 1:3)

^{1}H NMR (400 MHz, CDCl$_3$, 298 K) δ 7.65 (s, 1H), 7.49 – 7.41 (m, 4H), 7.34 (d, *J* = 8.3 Hz, 2H), 7.32 – 7.27 (m, 3H), 3.78 (s, 2H), 2.16 (s, 3H)

^{13}C NMR (101 MHz, CDCl$_3$, 298 K) δ 168.8, 136.6, 132.8, 131.7, 128.6, 128.4, 128.0, 123.7, 120.4, 87.6, 82.8, 25.3, 24.6

2-(3-Phenylprop-2-yn-1-yl)naphthalene (2v)[9]

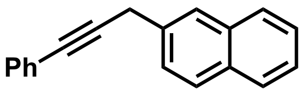

Prepared according to the general procedure using **1v** (129.2 mg, 0.50 mmol). The crude product was purified by flash column chromatography on silica gel (pentane/EtOAc = 50:1) to afford **2v** as a white solid (92 mg, 76% yield).

Formula: $C_{19}H_{14}$

Molecular weight: 242.32

Isolated yield: 76% (92 mg), white solid

Rf: 0.4 (Pentane/EtOAc = 50:1)

^{1}H NMR (400 MHz, CDCl$_3$, 298 K) δ 7.91 (s, 1H), 7.89 – 7.79 (m, 3H), 7.63 – 7.43 (m, 5H), 7.40 – 7.27 (m, 3H), 4.02 (s, 2H)

^{13}C NMR (101 MHz, CDCl$_3$, 298 K) δ 134.3, 133.7, 132.5, 131.8, 128.4, 128.3, 128.0, 127.8, 126.6, 126.4, 126.3, 125.7, 123.8, 87.6, 83.0, 26.1

2-(3-Phenylprop-2-yn-1-yl)thiophene (2w)[14]

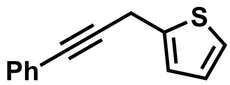

Prepared according to the general procedure using **1w** (107.1 mg, 0.50 mmol). The crude product was purified by flash column chromatography on silica gel (pentane/EtOAc = 50:1) to afford **2w** as a yellow oil (48 mg, 48% yield).

Formula: $C_{13}H_{10}S$

Molecular weight: 198.28

Isolated yield: 48% (48 mg), yellow oil

Rf: 0.4 (Pentane/EtOAc = 50:1)

^{1}H NMR (400 MHz, CDCl$_3$, 298 K) δ 7.52 – 7.45 (m, 2H), 7.37 – 7.30 (m, 3H), 7.21 (dd, *J* = 5.1, 1.1 Hz, 1H), 7.10 – 7.03 (m, 1H), 6.98 (dd, *J* = 5.1, 3.5 Hz, 1H), 4.02 (s, 2H)

^{13}C NMR (101 MHz, CDCl$_3$, 298 K) δ 139.7, 131.8, 128.4, 128.1, 127.0, 125.2, 124.2, 123.5, 86.8, 82.5, 20.8

5.2 Compounds monitored by crude ¹H NMR

The following compounds were monitored by crude ¹H NMR due to low conversion or loss during purification, attributed to volatility and/or instability under the purification conditions. Yields were estimated from the crude reaction mixtures using mesitylene as an internal standard.

3,3'-(Prop-1-yne-1,3-diyl)bis(fluorobenzene) (2f)

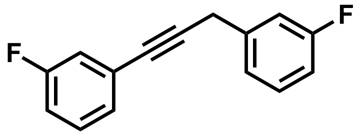

Prepared according to a modified general procedure using **1f** (122.1 mg, 0.50 mmol) with 2 mol% of **C1** at 80 °C. The reaction mixture was analyzed by ¹H NMR spectroscopy, giving **2f** in 13% NMR yield (no purification performed).

Formula: $C_{15}H_{10}F_2$
Molecular weight: 228.24
NMR yield: 13%

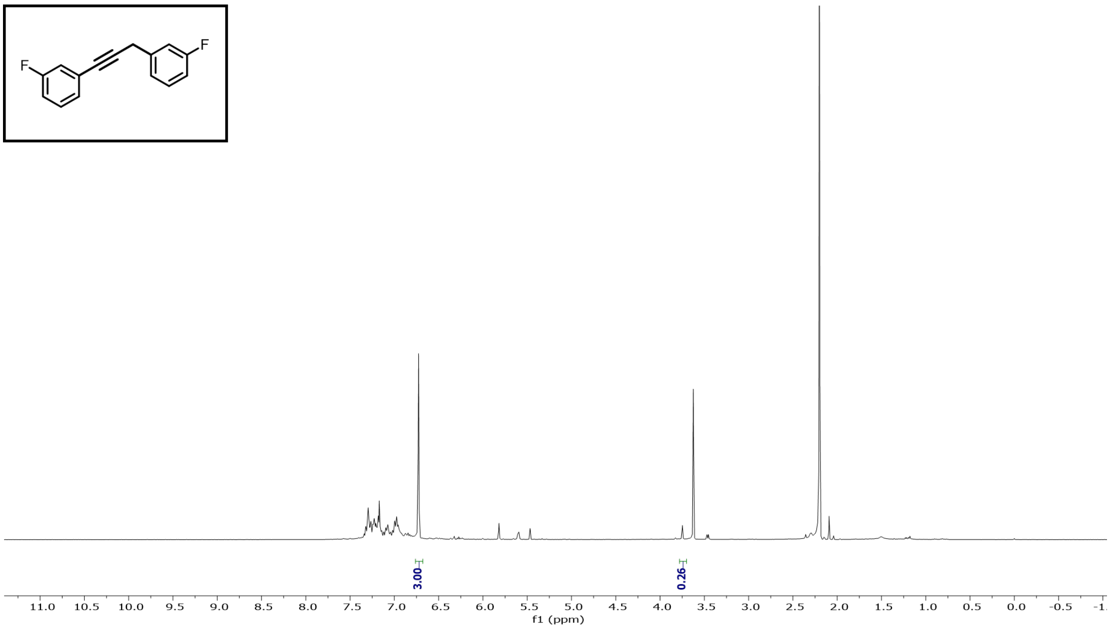

Figure S1 – ¹H NMR (400 MHz, CDCl₃, 298 K) spectrum of the crude reaction mixture showing **2f**.

1-(3-Phenylprop-2-yn-1-yl)-4-(trifluoromethyl)benzene (2s)

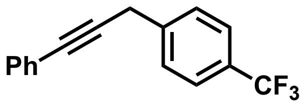

Prepared according to a modified general procedure using **1s** (138.1 mg, 0.50 mmol). The reaction was carried out for 16 h. The reaction mixture was analyzed by ^{1}H NMR spectroscopy, giving **2s** in 10% NMR yield (no purification performed).

Formula: $C_{16}H_{11}O_3$

Molecular weight: 260.26

NMR yield: 10%

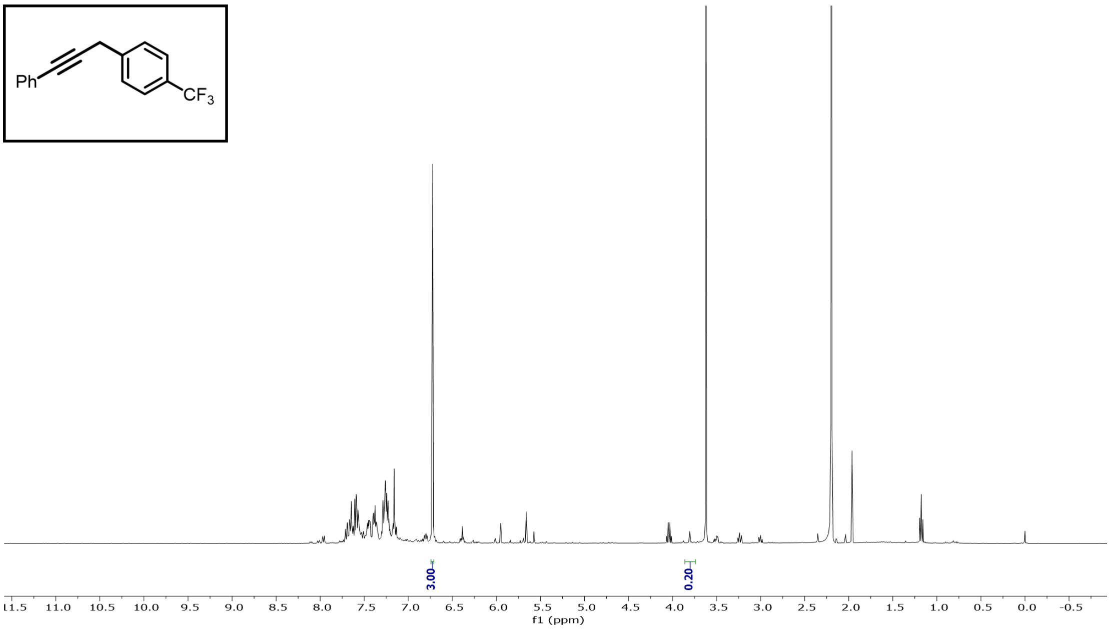

Figure S2 – ^{1}H NMR (400 MHz, $CDCl_3$, 298 K) spectrum of the crude reaction mixture showing **2s**.

Methyl 4-(3-phenylprop-2-yn-1-yl)benzoate (2t)

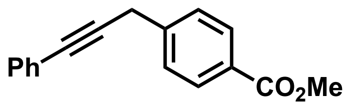

Prepared according to a modified general procedure using **1t** (133.2 mg, 0.50 mmol) at 80 °C. The reaction mixture was analyzed by ^{1}H NMR spectroscopy, giving **2t** in 19% NMR yield (no purification performed).

Formula: $C_{17}H_{14}O_2$

Molecular weight: 250.30

NMR yield: 19%

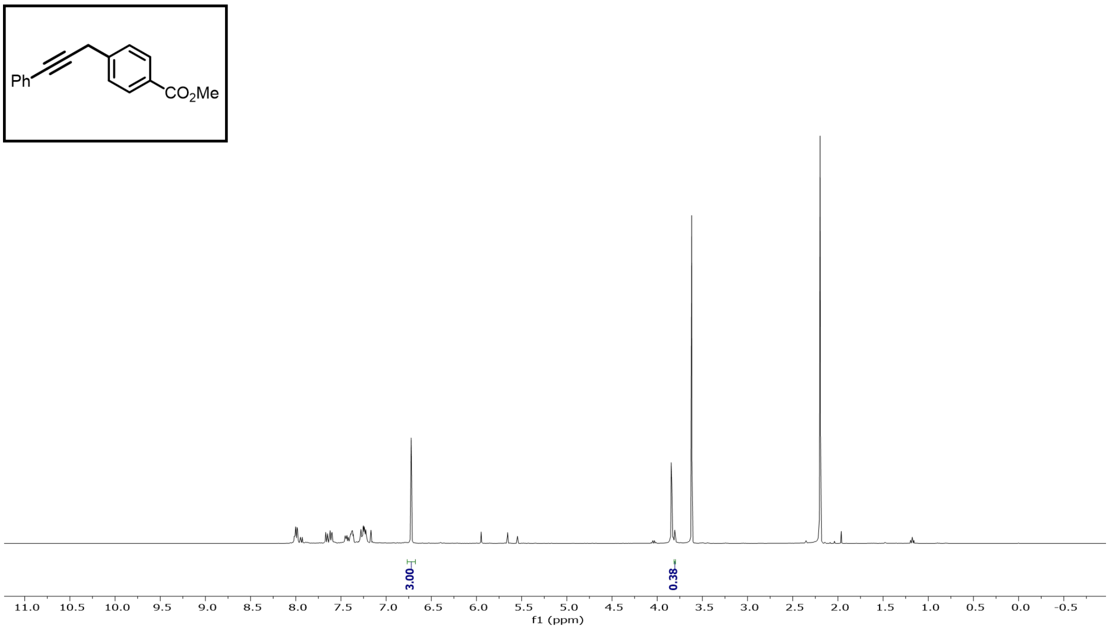

Figure S3 – ^{1}H NMR (400 MHz, $CDCl_3$, 298 K) spectrum of the crude reaction mixture showing **2t**.

(3-Cyclohexylprop-2-yn-1-yl)benzene (2y)

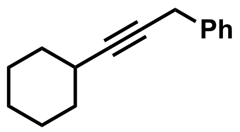

Prepared according to the general procedure using **1y** (107.2 mg, 0.50 mmol). The reaction mixture was analyzed by ^{1}H NMR spectroscopy, giving **2y** in 25% NMR yield (no purification performed).

Formula: $C_{15}H_{18}$

Molecular weight: 198.31

NMR yield: 25%

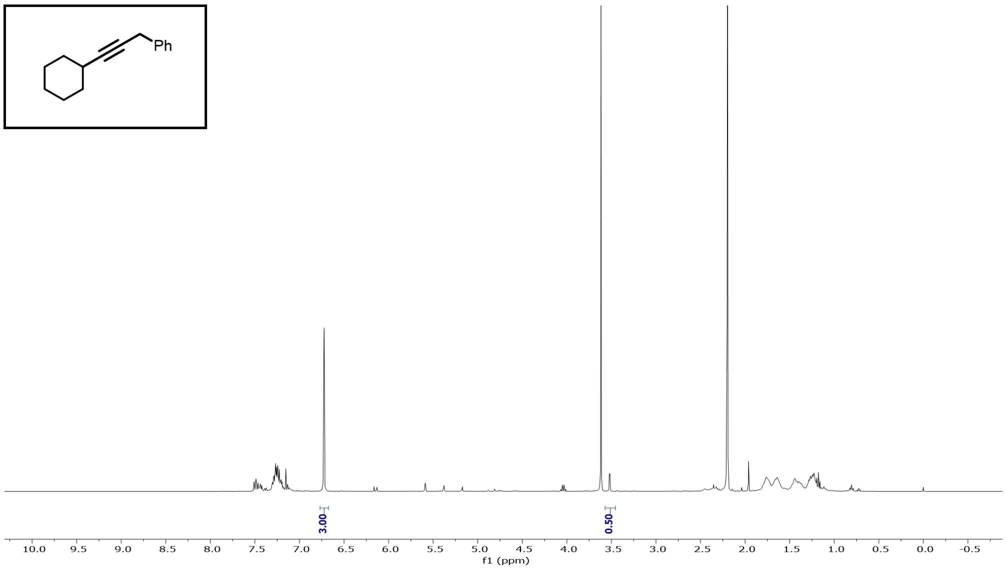

Figure S4 – ^{1}H NMR (400 MHz, $CDCl_3$, 298 K) spectrum of the crude reaction mixture showing **2y**.

6. Preparation of Allyl Ether Derivatives 3

6.1 General Procedure for the Preparation of Allyl Ether Derivatives 3

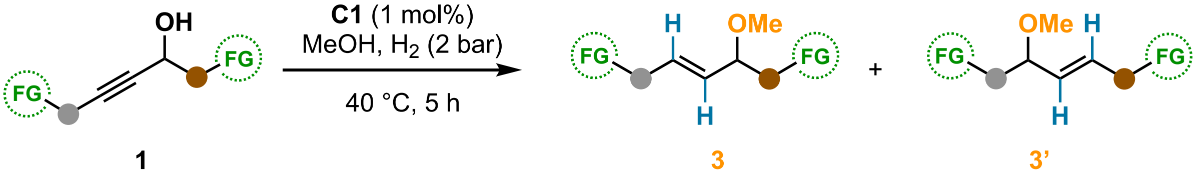

In an oven-dried Fisher–Porter tube under an argon atmosphere, the corresponding propargylic alcohol **1** (0.50 mmol), catalyst **C1** (6.7 mg, 1 mol%), and methanol (2 mL) were added. The mixture was flash-frozen using liquid nitrogen, evacuated, and subsequently pressurized with H_2 (2 bar). The sealed tube was stirred at 40 °C for 5 h. After completion, the reaction was cooled to room temperature, and the pressure was carefully released. The solvent was removed under reduced pressure. Conversion and crude yield were determined by ^{1}H NMR spectroscopy using mesitylene (0.50 mmol) as an internal standard. Pure compound **3** or a mixture of geometric isomers **3/3'** was isolated by flash column chromatography on silica gel using a gradient of EtOAc/pentane as the eluent.

(*E*)-(3-Methoxyprop-1-ene-1,3-diyl)dibenzene (3a)[15]

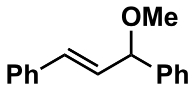

Prepared according to the general procedure using **1a** (104.2 mg, 0.50 mmol). The crude product was purified by flash column chromatography on silica gel (pentane/EtOAc/Et_3N = 100:2:1) to afford **3a** as a colorless oil (91 mg, 81% yield).

Formula: $C_{16}H_{16}O$

Molecular weight: 224.30

Isolated yield: 81% (91 mg), colorless oil

Rf: 0.3 (Pentane/EtOAc/Et_3N = 100:2:1)

^{1}H NMR (400 MHz, $CDCl_3$, 298 K) δ 7.38 – 7.30 (m, 6H), 7.28 – 7.22 (m, 3H), 7.21 – 7.15 (m, 1H), 6.60 (d, J = 15.9 Hz, 1H), 6.25 (dd, J = 15.9, 7.0 Hz, 1H), 4.76 (d, J = 7.0 Hz, 1H), 3.34 (s, 3H)

^{13}C NMR (101 MHz, $CDCl_3$, 298 K) δ 141.1, 136.7, 131.6, 130.2, 128.6, 128.6, 127.8, 127.0, 126.7, 84.4, 56.6

(*E*)-4,4'-(3-methoxyprop-1-ene-1,3-diyl)bis(methylbenzene) (3b)[16]

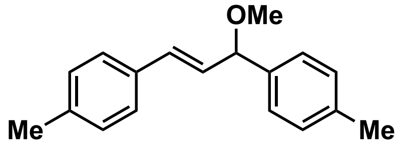

Prepared according to a modified general procedure using **1b** (104.2 mg, 0.50 mmol). The reaction mixture was stirred for 3 h. The crude product was purified by flash column chromatography on silica gel (pentane/EtOAc/Et_3N = 100:2:1) to afford **3b** as a colorless oil (82 mg, 65% yield).

Formula: $C_{18}H_{20}O$

Molecular weight: 252.36

Isolated yield: 65% (82 mg), colorless oil

Rf: 0.3 (Pentane/EtOAc/Et_3N = 100:2:1)

^{1}H NMR (400 MHz, $CDCl_3$, 298 K) δ 7.27 (d, *J* = 6.6 Hz, 4H), 7.16 (d, *J* = 7.9 Hz, 2H), 7.09 (d, *J* = 7.9 Hz, 2H), 6.57 (d, *J* = 15.9 Hz, 1H), 6.23 (dd, *J* = 15.9, 7.0 Hz, 1H), 4.75 (d, *J* = 7.0 Hz, 1H), 3.35 (s, 3H), 2.34 (s, 3H), 2.31 (s, 3H)

^{13}C NMR (101 MHz, $CDCl_3$, 298 K) δ 138.3, 137.6, 137.4, 134.0, 131.3, 129.4, 129.3, 129.3, 126.9, 126.6, 84.3, 56.4, 21.3, 21.3

(*E*)-4,4'-(3-Methoxyprop-1-ene-1,3-diyl)bis(fluorobenzene) (3e)

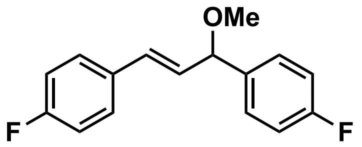

Prepared according to the general procedure using **1e** (122.2 mg, 0.50 mmol). The crude product was purified by flash column chromatography on silica gel (pentane/EtOAc/Et_3N = 100:2:1) to afford **3e** as a colorless oil (116 mg, 89% yield).

Formula: $C_{16}H_{14}OF_2$

Molecular weight: 260.28

Isolated yield: 89% (116 mg), colorless oil

Rf: 0.3 (Pentane/EtOAc/Et_3N = 100:2:1)

^{1}H NMR (400 MHz, $CDCl_3$, 298 K) δ 7.41 – 7.29 (m, 4H), 7.10 – 6.92 (m, 4H), 6.57 (d, *J* = 15.9 Hz, 1H), 6.17 (dd, *J* = 15.9, 6.9 Hz, 1H), 4.77 (d, *J* = 6.9 Hz, 1H), 3.36 (s, 3H)

^{13}C NMR (101 MHz, $CDCl_3$, 298 K) δ 162.6 (d, *J* = 248.2 Hz), 162.5 (d, *J* = 246.8 Hz), 136.9 (d, *J* = 3.2 Hz), 132.7 (d, *J* = 3.3 Hz), 130.6, 129.8 (d, *J* = 2.2 Hz), 128.6 (d, *J* = 8.0 Hz), 128.3 (d, *J* = 8.0 Hz), 115.6 (d, *J* = 21.7 Hz), 115.5 (d, *J* = 21.5 Hz), 83.6, 56.6.

^{19}F NMR (376 MHz, $CDCl_3$, 298 K) δ -114.0, -114.7

HRMS (GC-EI): Calcd. for $C_{16}H_{14}OF_2^+$ $[M]^+$: 260.10072; Found 260.10105

(*E*)-3,3'-(3-methoxyprop-1-ene-1,3-diyl)bis(fluorobenzene) (3f)

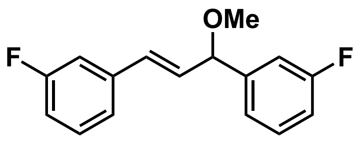

Prepared according to a modified general procedure using **1f** (122.2 mg, 0.50 mmol) at 80 °C. The crude product was purified by flash column chromatography on silica gel (pentane/EtOAc/Et_3N = 100:2:1) to afford **3f** as a colorless oil (117 mg, 90% yield).

Formula: $C_{16}H_{14}OF_2$

Molecular weight: 260.28

Isolated yield: 90% (117 mg), colorless oil

Rf: 0.3 (Pentane/EtOAc/Et_3N = 100:2:1)

^{1}H NMR (400 MHz, $CDCl_3$, 298 K) δ 7.34 – 7.19 (m, 2H), 7.16 – 7.03 (m, 4H), 6.99 – 6.88 (m, 2H), 6.59 (d, *J* = 15.9 Hz, 1H), 6.22 (dd, *J* = 15.9, 6.9 Hz, 1H), 4.77 (d, *J* = 6.9 Hz, 1H), 3.36 (s, 3H)

^{13}C NMR (101 MHz, $CDCl_3$, 298 K) δ 163.2 (d, *J* = 247.4 Hz), 163.1 (d, *J* = 246.2 Hz), 143.6 (d, *J* = 6.7 Hz), 138.8 (d, *J* = 7.7 Hz), 131.1, 130.7 (d, *J* = 2.5 Hz), 130.2 (d, *J* = 6.7 Hz), 130.1 (d, *J* = 7.0 Hz), 122.6 (d, *J* = 2.8 Hz), 122.5 (d, *J* = 2.9 Hz), 114.8 (d, *J* = 21.2 Hz), 114.7 (d, *J* = 21.1 Hz), 113.7 (d, *J* = 22.0 Hz), 113.2 (d, *J* = 21.8 Hz), 83.5 (d, *J* = 1.7 Hz), 56.7

^{19}F NMR (376 MHz, $CDCl_3$, 298 K) δ -112.6, -113.3

HRMS (GC-EI): Calcd. for $C_{16}H_{14}OF_2^+$ $[M]^+$: 260.10072; Found 260.10105

(*E*)-2,2'-(3-methoxyprop-1-ene-1,3-diyl)bis(fluorobenzene) (3g)

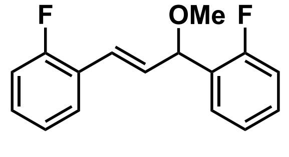

Prepared according to a modified general procedure using **1g** (122.2 mg, 0.50 mmol) at 80 °C. The crude product was purified by flash column chromatography on silica gel (pentane/EtOAc/Et_3N = 100:2:1) to afford **3g** as a colorless oil (104 mg, 80% yield).

Formula: $C_{16}H_{14}OF_2$

Molecular weight: 260.28

Isolated yield: 80% (104 mg), colorless oil

^{1}H NMR (400 MHz, $CDCl_3$, 298 K) δ 7.51 – 7.38 (m, 2H), 7.27 – 7.14 (m, 3H), 7.07 – 6.96 (m, 3H), 6.83 (d, *J* = 16.1 Hz, 1H), 6.36 (dd, *J* = 16.1, 6.9 Hz, 1H), 5.17 (d, *J* = 6.9 Hz, 1H), 3.39 (s, 3H)

^{13}C NMR (101 MHz, $CDCl_3$, 298 K) δ 160.5 (d, *J* = 250.6 Hz), 160.4 (d, *J* = 247.7 Hz), 131.4 (d, *J* = 4.6 Hz), 129.4 (d, *J* = 8.3 Hz), 129.2 (d, *J* = 8.4 Hz), 128.1 (d, J = 13.4 Hz), 128.1 (d, *J* = 4.1 Hz), 127.7 (d, *J* = 3.7 Hz), 124.6 (d, *J* = 3.5 Hz), 124.4 (d, *J* = 12.1 Hz), 124.2 (d, *J* = 3.6 Hz), 124.1 (d, *J* = 3.9 Hz), 115.8 (d, *J* = 22.1 Hz), 115.6 (d, *J* = 21.8 Hz), 77.7 (d, *J* = 2.6 Hz), 56.8

^{19}F NMR (376 MHz, $CDCl_3$, 298 K) δ -117.7, -119.1

HRMS (GC-EI): Calcd. for $C_{16}H_{14}OF_2^+$ $[M]^+$: 260.10072; Found 260.10066

(*E*)-4,4'-(3-methoxyprop-1-ene-1,3-diyl)bis(chlorobenzene) (3h)[16]

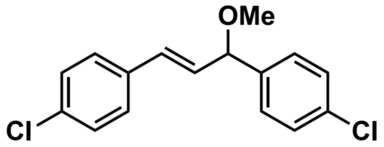

Prepared according to the general procedure using **1h** (138.6 mg, 0.50 mmol). The crude product was purified by flash column chromatography on silica gel (pentane/EtOAc/Et_3N = 100:2:1) to afford **3h** as a white solid (113 mg, 77% yield).

Formula: $C_{16}H_{14}OCl_2$

Molecular weight: 293.19

Isolated yield: 77% (113 mg), white solid

Rf: 0.3 (Pentane/EtOAc/Et_3N = 100:2:1)

^{1}H NMR (400 MHz, $CDCl_3$, 298 K) δ 7.34 – 7.18 (m, 8H), 6.52 (d, *J* = 15.9 Hz, 1H), 6.15 (dd, *J* = 15.9, 6.9 Hz, 1H), 4.72 (d, *J* = 6.9 Hz, 1H), 3.32 (s, 3H)

^{13}C NMR (101 MHz, $CDCl_3$, 298 K) δ 139.4, 135.0, 133.7, 133.6, 130.6, 130.5, 128.9, 128.9, 128.3, 127.9, 83.5, 56.6

(*E*)-4,4''-(3-Methoxyprop-1-ene-1,3-diyl)di-1,1'-biphenyl (3i)

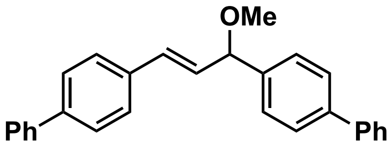

Prepared according to a modified general procedure using **1i** (90.1 mg, 0.25 mmol). The crude product was purified by flash column chromatography on silica gel (pentane/EtOAc/Et_3N = 100:3:1) to afford **3i** as a white solid (67 mg, 71% yield).

Formula: $C_{28}H_{24}O$

Molecular weight: 376.50

Isolated yield: 71% (67 mg), white solid

Rf: 0.3 (Pentane/EtOAc/Et_3N = 100:3:1)

^{1}H NMR (400 MHz, $CDCl_3$, 298 K) δ 7.66 – 7.58 (m, 8H), 7.54 – 7.44 (m, 8H), 7.41 – 7.35 (m, 2H), 6.75 (d, *J* = 15.9 Hz, 1H), 6.41 (dd, *J* = 15.9, 7.0 Hz, 1H), 4.91 (d, *J* = 7.0 Hz, 1H), 3.47 (s, 3H)

^{13}C NMR (101 MHz, $CDCl_3$, 298 K) δ 141.0, 140.8, 140.7, 140.6, 140.2, 135.7, 132.4, 131.2, 130.2, 128.9, 128.9, 127.5, 127.4, 127.4, 127.2, 127.2, 127.0, 84.2, 56.6

HRMS (GC-EI): Calcd. for $C_{28}H_{24}O^+$ $[M]^+$: 376.18217; Found 376.18281

(*E*)-2,2'-(3-methoxyprop-1-ene-1,3-diyl)dinaphthalene (3j)

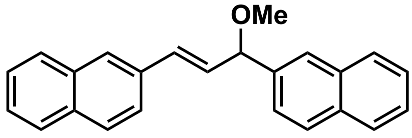

Prepared according to a modified general procedure using **1j** (154.2 mg, 0.50 mmol), with the reaction stirred for 3 h. The crude product was purified by flash column chromatography on silica gel (pentane/DCM/Et_3N = 80:20:1) to afford **3j** as a white solid (130 mg, 80% yield).

Formula: $C_{24}H_{20}O$

Molecular weight: 324.42

Isolated yield: 80% (130 mg), white solid

Rf: 0.3 (Pentane/DCM/Et_3N = 80:20:1)

^{1}H NMR (400 MHz, $CDCl_3$, 298 K) δ 7.94 – 7.86 (m, 4H), 7.85 – 7.75 (m, 4H), 7.65 – 7.57 (m, 2H), 7.55 – 7.43 (m, 4H), 6.87 (d, *J* = 15.9 Hz, 1H), 6.53 (dd, *J* = 15.9, 6.8 Hz, 1H), 5.06 (d, *J* = 6.8 Hz, 1H), 3.49 (s, 3H)

^{13}C NMR (101 MHz, $CDCl_3$, 298 K) δ 138.5, 134.2, 133.6, 133.5, 133.2, 133.2, 131.8, 130.6, 128.6, 128.3, 128.1, 128.1, 127.9, 127.8, 126.8, 126.4, 126.3, 126.1, 126.0, 125.9, 125.0, 123.7, 84.6, 56.7

GC-MS (EI): theor. m/z 324.150865, found 324.150950

(*E*)-4,4'-(3-Methoxyprop-1-ene-1,3-diyl)bis((trifluoromethyl)benzene) (3k)

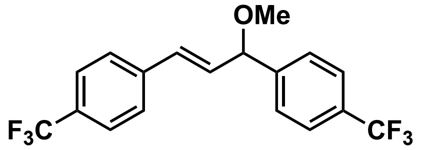

Prepared according to a modified general procedure using **1k** (86.1 mg, 0.25 mmol) with 3 mol% of **C1** at 80 °C. The crude product was purified by flash column chromatography on silica gel (pentane/EtOAc/Et_3N = 100:3:1) to afford **3k** as a white solid (54 mg, 60% yield).

Formula: $C_{18}H_{14}OF_2$

Molecular weight: 360.30

Isolated yield: 60% (54 mg), white solid

Rf: 0.3 (Pentane/EtOAc/Et_3N = 100:3:1)

^{1}H NMR (400 MHz, $CDCl_3$, 298 K) δ 7.64 (d, *J* = 8.1 Hz, 2H), 7.60 – 7.40 (m, 6H), 6.69 (d, *J* = 15.9 Hz, 1H), 6.32 (dd, *J* = 15.9, 6.7 Hz, 1H), 4.88 (d, *J* = 6.7 Hz, 1H), 3.40 (s, 3H)

^{13}C NMR (101 MHz, $CDCl_3$, 298 K) δ 144.7, 139.8, 132.1, 130.7, 130.2 (q, *J* = 32.5 Hz), 129.9 (q, *J* = 32.6 Hz), 127.2, 126.9, 125.8 (q, *J* = 3.9 Hz), 125.7 (q, *J* = 4.0 Hz), 124.2 (q, *J* = 273.1 Hz), 124.2 (q, *J* = 273.0 Hz), 83.5, 56.9

^{19}F NMR (376 MHz, $CDCl_3$, 298 K) δ -62.5, -62.6

HRMS (GC-EI): Calcd. for $C_{18}H_{14}OF_2^+$ $[M]^+$: 360.09434; Found 360.09472

Dimethyl 4,4'-(3-methoxyprop-1-ene-1,3-diyl)(E)-dibenzoate (3l)

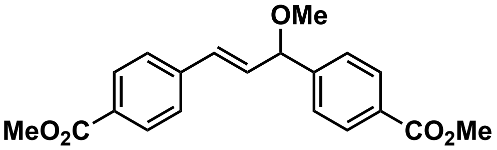

Prepared according to a modified general procedure using **1l** (81.1 mg, 0.25 mmol) with 2 mol% of complex **C1** at 80 °C. The crude product was purified by flash column chromatography on silica gel (pentane/EtOAc/Et_3N = 5:1:0.1) to afford **3l** as a yellow solid (55 mg, 65% yield).

Formula: $C_{20}H_{20}O_5$

Molecular weight: 340.37

Isolated yield: 65% (55 mg), yellow solid

Rf: 0.3 (Pentane/EtOAc/Et_3N = 5:1:0.1)

^{1}H NMR (400 MHz, $CDCl_3$, 298 K) δ 8.04 (d, *J* = 8.2 Hz, 2H), 7.96 (d, *J* = 8.2 Hz, 2H), 7.51 – 7.37 (m, 4H), 6.67 (d, *J* = 15.9 Hz, 1H), 6.34 (dd, *J* = 15.9, 6.8 Hz, 1H), 4.86 (d, *J* = 6.8 Hz, 1H), 3.91 (s, 3H), 3.89 (s, 3H), 3.39 (s, 3H)

^{13}C NMR (101 MHz, $CDCl_3$, 298 K) δ 167.0, 166.9, 145.8, 140.9, 132.2, 131.0, 130.1, 130.0, 129.8, 129.4, 126.9, 126.6, 83.8, 56.8, 52.3, 52.2

HR-MS (ESI): Calcd. for $C_{20}H_{20}O_5Na^+$ $[M+Na]^+$: 363.12029, found 363.11997

(*E*)-1-(1-Methoxy-3-phenylallyl)-4-methylbenzene (3m)

(*E*)-1-(3-Methoxy-3-phenylprop-1-en-1-yl)-4-methylbenzene (3m’)

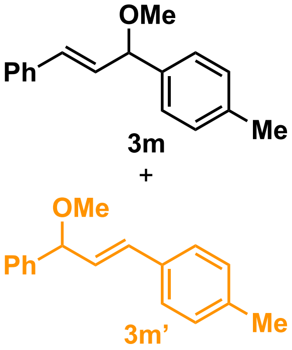

Prepared according to the general procedure using **1m** (111.2 mg, 0.50 mmol). The crude product was purified by flash column chromatography on silica gel (pentane/EtOAc/Et_3N = 100:2:1) to afford a mixture of **3m** and **3m’** (44:56 ratio) as a colorless oil (86 mg, 72% yield).

Ratio: 3m:3m’ = 44:56

Formula: $C_{17}H_{18}O$

Molecular weight: 238.33

Isolated yield: 72% (86 mg), colorless oil

Rf: 0.3 (Pentane/EtOAc/Et_3N = 100:2:1)

^{1}H NMR (400 MHz, $CDCl_3$, 298 K) δ 7.38 – 7.28 (m, 3H), 7.26 – 7.21 (m, 3H), 7.20 – 7.10 (m, 2H), 7.06 (d, *J* = 7.9 Hz, 1H), 6.64 – 6.46 (m, 1H), 6.32 – 6.09 (m, 1H), 4.84 – 4.64 (m, 1H), 3.33 (s, 0.56 ×3H), 3.32 (s, 0.44 ×3H), 2.30 (s, 0.56 ×3H), 2.27 (s, 0.44 ×3H)

^{13}C NMR (101 MHz, CDCl$_3$, 298 K) δ 141.3, 138.1, 137.7, 137.6, 136.8, 133.9, 131.6, 131.3, 130.4, 129.4, 129.2, 128.6, 127.8, 127.8, 127.0, 126.7, 126.6, 84.5, 84.3, 56.5, 56.5, 21.3, 21.3

HRMS (ESI$^+$): Calcd. for $C_{17}H_{18}ONa^+$ $[M+Na]^+$: 261.12499; Found 261.12495

(*E*)-1-Fluoro-4-(1-methoxy-3-phenylallyl)benzene (3q)

(*E*)-1-Fluoro-4-(3-methoxy-3-phenylprop-1-en-1-yl)benzene (3q')

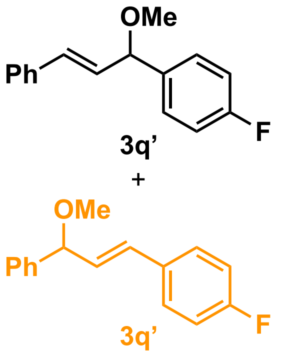

Prepared according to the general procedure using **1q** (113.1 mg, 0.50 mmol). The crude product was purified by flash column chromatography on silica gel (pentane/EtOAc/Et_3N = 100:3:1) to afford a mixture of **3q** and **3q'** (50:50 ratio) as a colorless oil (99 mg, 82% yield).

Ratio: 3q:3q' = 50:50

Formula: $C_{16}H_{15}OF$

Molecular weight: 242.29

Isolated yield: 82% (99 mg), colorless oil

Rf: 0.3 (Pentane/EtOAc/Et_3N = 100:3:1)

^{1}H NMR (400 MHz, CDCl$_3$, 298 K) δ 7.38 – 7.18 (m, 7H), 7.06 – 6.91 (m, 2H), 6.65 – 6.47 (m, 1H), 6.30 – 6.09 (m, 1H), 4.84 – 4.66 (m, 1H), 3.35 (s, 3H)

^{13}C NMR (101 MHz, CDCl$_3$, 298 K) δ 162.5 (d, *J* = 247.9 Hz), 162.4 (d, *J* = 246.6 Hz), 141.0, 137.0 (d, *J* = 3.1 Hz), 136.5, 132.9 (d, *J* = 3.3 Hz), 131.8, 130.3, 130.1, 130.1, 130.0, 128.7, 128.6 (d, *J* = 8.1 Hz), 128.2 (d, *J* = 8.0 Hz), 128.0, 127.9, 127.0, 126.7. 115.5 (d, *J* = 21.7 Hz), 115.5 (d, *J* = 21.5 Hz), 84.3, 83.7, 56.6, 56.5

^{19}F NMR (376 MHz, CDCl$_3$, 298 K) δ -114.2, -114.8.

HRMS (ESI$^+$): Calcd. for $C_{16}H_{15}OF^+$ $[M]^+$: 242.11014; Found 242.10974

(*E*)-1-Chloro-4-(1-methoxy-3-phenylallyl)benzene (3r)

(*E*)-1-Chloro-4-(3-methoxy-3-phenylprop-1-en-1-yl)benzene (3r')

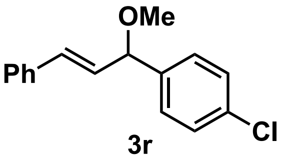

3r

+

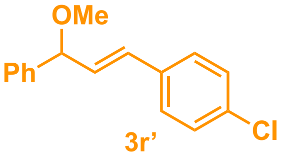

3r'

Prepared according to the general procedure using **1r** (121.4 mg, 0.50 mmol). The crude product was purified by flash column chromatography on silica gel (pentane/EtOAc/Et_3N = 100:3:1) to afford a mixture of **3r** and **3r'** (50:50 ratio) as a colorless oil (89 mg, 69% yield).

Ratio: 3r:3r' = 50:50

Formula: $C_{16}H_{15}OCl$

Molecular weight: 258.75

Isolated yield: 69% (89 mg), colorless oil

Rf: 0.4 (Pentane/EtOAc/Et_3N = 100:3:1)

^{1}H NMR (400 MHz, $CDCl_3$, 298 K) δ 7.32 – 7.27 (m, 3H), 7.25 – 7.17 (m, 6H), 6.61 – 6.42 (m, 1H), 6.23 – 6.08 (m, 1H), 4.79 – 4.61 (m, 1H), 3.29 (s, 3H)

^{13}C NMR (101 MHz, $CDCl_3$, 298 K) δ 140.8, 139.7, 136.4, 135.2, 133.5, 133.4, 132.0, 131.0, 130.1, 129.7, 128.8, 128.7, 128.7, 128.5, 128.3, 128.0, 127.9, 127.9, 127.0, 126.7, 84.2, 83.7, 56.6, 56.6

HRMS (ESI$^+$): Calcd. for $C_{16}H_{15}OCl^+$ $[M]^+$: 258.08059; Found 258.08023

(*E*)-2-(1-Methoxy-3-phenylallyl)naphthalene (3v)

(*E*)-2-(3-Methoxy-3-phenylprop-1-en-1-yl)naphthalene (3v')

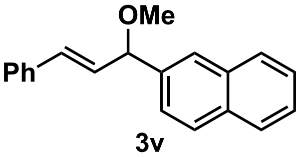

3v

+

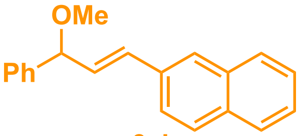

3v'

Prepared according to the general procedure using **1v** (129.2 mg, 0.50 mmol). The crude product was purified by flash column chromatography on silica gel (pentane/EtOAc/Et_3N = 100:3:1) to afford a mixture of **3v** and **3v'** (42:58 ratio) as a colorless oil (116 mg, 85% yield).

Ratio: 3v:3v' = 42:58

Formula: $C_{20}H_{18}O$

Molecular weight: 274.36

Isolated yield: 85% (116 mg), colorless oil

Rf: 0.4 (Pentane/EtOAc/Et_3N = 100:3:1)

^{1}H NMR (400 MHz, $CDCl_3$, 298 K) δ 7.86 – 7.68 (m, 4H), 7.58 – 7.33 (m, 6H), 7.31 – 7.17 (m, 2H), 6.76 (d, *J* = 15.9 Hz, 0.58 ×1H), 6.65 (d, *J* = 15.9 Hz, 0.42 ×1H), 6.44 – 6.29 (m, 1H), 4.94 (d, *J* = 6.8 Hz, 0.42 ×1H), 4.83 (d, *J* = 7.0 Hz, 0.58 ×1H), 3.40 (s, 0.42 ×3H), 3.38 (s, 0.58 ×3H)

^{13}C NMR (101 MHz, $CDCl_3$, 298 K) δ 141.1, 138.5, 136.7, 134.2, 133.7, 133.5, 133.2, 133.2, 131.7, 131.7, 130.7, 130.2, 128.7, 128.7, 128.5, 128.3, 128.1, 127.9, 127.9, 127.8, 127.8, 127.0, 126.8, 126.7, 126.4, 126.3, 126.1, 126.0, 125.9, 125.0, 123.8, 84.5, 84.5, 56.7, 56.6

HRMS (GC-EI): Calcd. for $C_{20}H_{18}O^+$ $[M]^+$: 274.13522; Found 274.13520, 274.13547

(*E*)-(3-Cyclohexyl-3-methoxyprop-1-en-1-yl)benzene (3x)

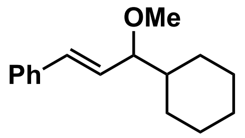

Prepared according to a modified general procedure using **1x** (122.1 mg, 0.50 mmol) at 80 °C. The crude product was purified by flash column chromatography on silica gel (pentane/EtOAc = 50:1) to afford **3x** as a colorless oil (99 mg, 86% yield).

Formula: $C_{16}H_{22}O$

Molecular weight: 230.35

Isolated yield: 86% (99 mg), colorless oil

Rf: 0.3 (Pentane/EtOAc = 50:1)

^{1}H NMR (400 MHz, $CDCl_3$, 298 K) δ 7.36 (d, *J* = 7.5 Hz, 2H), 7.28 (t, *J* = 7.5 Hz, 2H), 7.22 – 7.17 (m, 1H), 6.45 (d, *J* = 16.0 Hz, 1H), 6.02 (dd, *J* = 16.0, 8.2 Hz, 1H), 3.36 (t, *J* = 7.4 Hz, 1H), 3.26 (s, 3H), 1.93 – 1.84 (m, 1H), 1.73 – 1.59 (m, 4H), 1.54 – 1.45 (m, 1H), 1.24 – 1.09 (m, 3H), 1.03 – 0.91 (m, 2H)

^{13}C NMR (101 MHz, $CDCl_3$, 298 K) δ 136.8, 133.0, 129.3, 128.7, 127.7, 126.5, 87.5, 56.6, 43.0, 29.4, 29.0, 26.7, 26.3, 26.2

HRMS (GC-EI): Calcd. for $C_{16}H_{22}O^+$ $[M]^+$: 230.16652; Found 230.16662

6.2 Compounds monitored by crude ^{1}H NMR

The following compounds were monitored by crude ^{1}H NMR due to low conversion or loss during purification, attributed to volatility and/or instability under the purification conditions. Yields were estimated from the crude reaction mixtures using mesitylene as an internal standard.

(*E*)-4,4'-(3-methoxyprop-1-ene-1,3-diyl)bis(tert-butylbenzene) (3c)

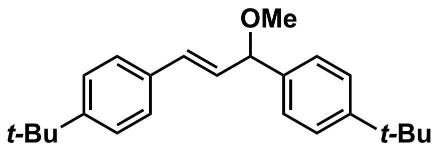

Prepared according to a modified general procedure using **1c** (160.2 mg, 0.50 mmol), with the reaction stirred for 3 h. The reaction mixture was analyzed by ^{1}H NMR spectroscopy, giving **3c** in 47% NMR yield (no purification performed).

Formula: $C_{24}H_{32}O$

Molecular weight: 336.24

NMR yield: 47%

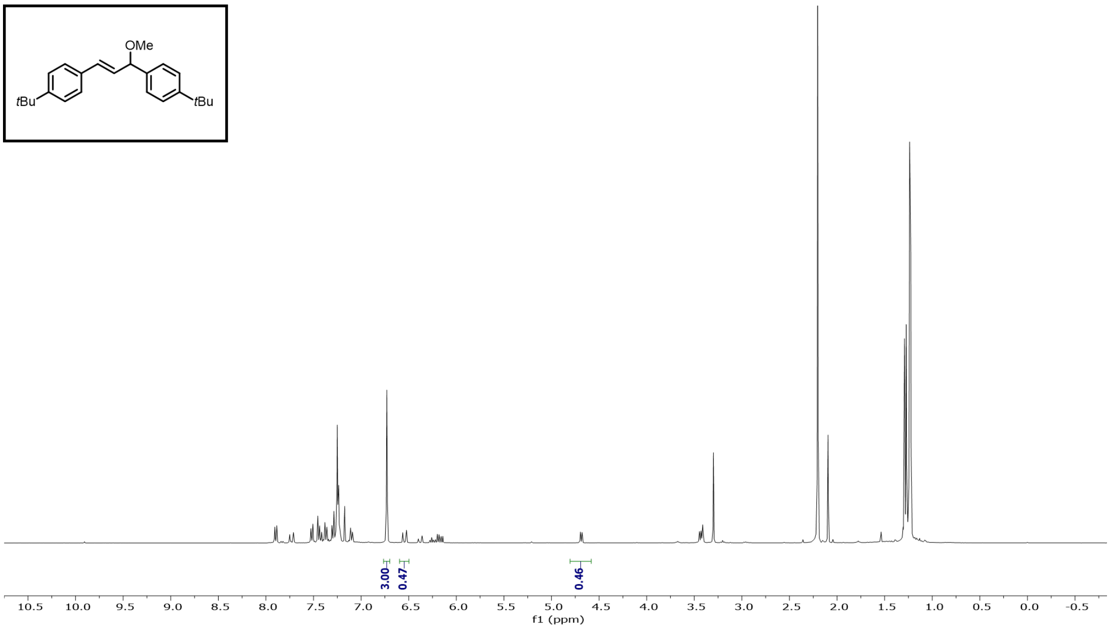

Figure S5 – ^{1}H NMR (400 MHz, $CDCl_3$, 298 K) spectrum of the crude reaction mixture showing **3c**.

7. Preparation of *trans*-Alkenes 4

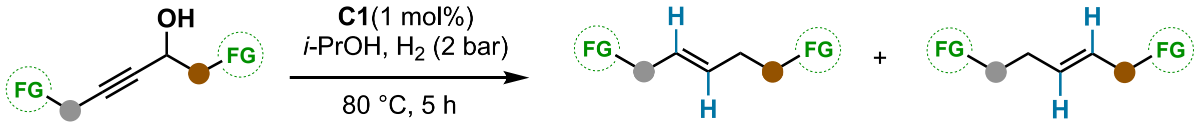

1 **4** **4’**

7.1 General Procedure for the Preparation of *trans*-Alkenes 4

In an oven-dried Fisher–Porter tube under an argon atmosphere, the propargylic alcohol **1** (0.50 mmol), complex **C1** (6.7 mg, 1 mol%), and *i*-PrOH (2 mL) were added. The mixture was flash-frozen using liquid nitrogen, evacuated, and then pressurized with H_2 (2 bar). The sealed tube was stirred at 80 °C for 5 h. After completion, the reaction was cooled to room temperature, and the pressure was carefully released. The solvent was removed under reduced pressure. Conversion and yield were determined by crude ^{1}H NMR spectroscopy using mesitylene (0.50 mmol) as an internal standard. The pure product **4** or a mixture of isomers **4/4’** was isolated by flash column chromatography on silica gel using EtOAc/pentane as the eluent.

(*E*)-Prop-1-ene-1,3-diyldibenzene (4a)[17]

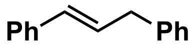

Prepared according to the general procedure using **1a** (104.1 mg, 0.50 mmol). The crude product was purified by flash column chromatography on silica gel (pentane/EtOAc = 50:1) to afford **4a** as a colorless oil (88 mg, 91% yield).

Formula: $C_{15}H_{14}$

Molecular weight: 194.28

Isolated yield: 91% (88 mg), as a colorless oil

Rf: 0.4 (Pentane/EtOAc = 50:1)

^{1}H NMR (400 MHz, CDCl$_3$, 298 K) δ 7.37 – 7.16 (m, 10H), 6.45 (d, *J* = 15.8 Hz, 1H), 6.35 (dt, *J* = 15.8, 6.6 Hz, 1H), 3.54 (d, *J* = 6.6 Hz, 2H)

^{13}C NMR (101 MHz, CDCl$_3$, 298 K) δ 140.3, 137.6, 131.2, 129.4, 128.8, 128.6, 128.6, 127.2, 126.3, 126.3, 39.5

(*E*)-4,4'-(Prop-1-ene-1,3-diyl)bis(methylbenzene) (4b)[17]

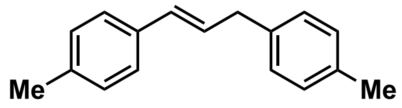

Prepared according to the general procedure using **1b** (118.2 mg, 0.50 mmol). The crude product was purified by flash column chromatography on silica gel (pentane/EtOAc = 50:1) to afford **4b** as a white solid (96 mg, 86% yield).

Formula: $C_{17}H_{18}$

Molecular weight: 222.33

Isolated yield: 86% (96 mg) as a white solid

Rf: 0.4 (Pentane/EtOAc = 50:1)

^{1}H NMR (400 MHz, CDCl$_3$, 298 K) δ 7.29 (d, *J* = 8.1 Hz, 2H), 7.20 – 7.11 (m, 6H), 6.46 (d, *J* = 15.7 Hz, 1H), 6.33 (dt, *J* = 15.7, 6.7 Hz, 1H), 3.54 (d, *J* = 6.7 Hz, 2H), 2.37 (s, 3H), 2.36 (s, 3H)

^{13}C NMR (101 MHz, CDCl$_3$, 298 K) δ 137.4, 136.9, 135.7, 134.9, 130.8, 129.3, 129.3, 128.7, 128.6, 126.1, 39.1, 21.3, 21.2

(*E*)-4,4'-(Prop-1-ene-1,3-diyl)bis(tert-butylbenzene) (4c)[18]

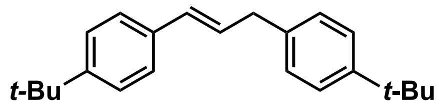

Prepared according to the general procedure using **1c** (160.2 mg, 0.50 mmol). The crude product was purified by flash column chromatography on silica gel (pentane/EtOAc = 100:3) to afford **4c** as a white solid (150 mg, 98% yield).

Formula: $C_{23}H_{30}$

Molecular weight: 306.49

Isolated yield: 98% (150 mg) as a white solid

Rf: 0.4 (Pentane/EtOAc = 100:3)

^{1}H NMR (400 MHz, CDCl$_3$, 298 K) δ 7.52 – 7.44 (m, 6H), 7.33 (d, *J* = 8.2 Hz, 2H), 6.61 (d, *J* = 15.7 Hz, 1H), 6.47 (dt, *J* = 15.7, 6.9 Hz, 1H), 3.66 (d, *J* = 6.9 Hz, 2H), 1.47 (s, 9H), 1.46 (s, 9H)

^{13}C NMR (101 MHz, CDCl$_3$, 298 K) δ 150.2, 149.1, 137.4, 134.9, 130.8, 128.7, 128.4, 126.0, 125.5, 125.5, 39.0, 34.6, 34.5, 31.6, 31.5

(*E*)-4,4'-(Prop-1-ene-1,3-diyl)bis(fluorobenzene) (4e)[17]

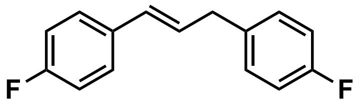

Prepared according to the general procedure using **1e** (122.2 mg, 0.50 mmol). The crude product was purified by flash column chromatography on silica gel (pentane/EtOAc = 50:1) to afford **4e** as a colorless oil (98 mg, 85% yield).

Formula: $C_{15}H_{12}F_2$

Molecular weight: 230.26

Isolated yield: 85% (98 mg) as a colorless oil

Rf: 0.4 (Pentane/EtOAc = 50:1)

^{1}H NMR (400 MHz, CDCl$_3$, 298 K) δ 7.36 – 7.29 (m, 2H), 7.24 – 7.17 (m, 2H), 7.05 – 6.95 (m, 4H), 6.41 (d, *J* = 15.8 Hz, 1H), 6.24 (dt, *J* = 15.8, 6.8 Hz, 1H), 3.52 (d, *J* = 6.8 Hz, 2H)

^{13}C NMR (101 MHz, CDCl$_3$, 298 K) δ 162.2 (d, *J* = 246.2 Hz), 161.7 (d, *J* = 243.9 Hz), 135.8 (d, *J* = 3.2 Hz), 133.6 (d, *J* = 3.3 Hz), 130.2 (d, *J* = 7.8 Hz), 130.1, 128.9 (d, *J* = 1.5 Hz), 127.7 (d, *J* = 7.9 Hz), 115.5 (d, *J* = 21.6 Hz), 115.4 (d, *J* = 21.2 Hz), 38.5

^{19}F NMR (376 MHz, CDCl$_3$, 298 K) δ -115.1, -117.1

(*E*)-3,3'-(Prop-1-ene-1,3-diyl)bis(fluorobenzene) (4f)[19]

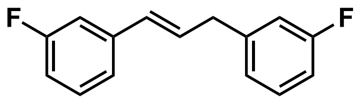

Prepared according to a modified general procedure using **1f** (61.1 mg, 0.25 mmol) with 3 mol% of **C1** at 100 °C. The crude product was purified by flash column chromatography on silica gel (pentane/EtOAc = 100:3) to afford **4f** as a colorless oil (28 mg, 49% yield).

Formula: $C_{15}H_{12}F_2$

Molecular weight: 230.26

Isolated yield: 49% (28 mg) as a colorless oil

Rf: 0.4 (Pentane/EtOAc = 100:3)

^{1}H NMR (400 MHz, CDCl$_3$, 298 K) δ 7.34 – 7.27 (m, 2H), 7.19 – 7.03 (m, 3H), 7.01 – 6.93 (m, 3H), 6.47 (d, *J* = 15.9 Hz, 1H), 6.43 – 6.29 (m, 1H), 3.59 (d, *J* = 6.5 Hz, 2H)

^{13}C NMR (101 MHz, CDCl$_3$, 298 K) δ 163.3 (d, *J* = 245.1 Hz), 163.1 (d, *J* = 246.7 Hz), 142.5 (d, *J* = 7.1 Hz), 139.7 (d, *J* = 7.7 Hz), 130.7 (d, *J* = 2.6 Hz), 130.1, 130.1, 129.9, 124.4 (d, *J* = 2.8 Hz), 122.2 (d, *J* = 2.7 Hz), 115.7 (d, *J* = 21.1 Hz), 114.2 (d, *J* = 21.4 Hz), 113.4 (d, *J* = 21.1 Hz), 112.7 (d, *J* = 21.8 Hz), 39.0 (d, *J* = 1.6 Hz)

^{19}F NMR (376 MHz, CDCl$_3$, 298 K) δ -113.4, -113.6

(*E*)-2,2'-(Prop-1-ene-1,3-diyl)bis(fluorobenzene) (4g)[19]

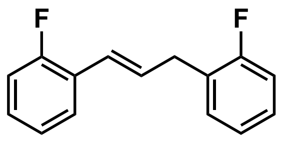

Prepared according to a modified general procedure using **1g** (61.1 mg, 0.25 mmol) with 3 mol% of **C1** at 100 °C. The crude product was purified by flash column chromatography on silica gel (pentane/EtOAc = 100:3) to afford **4g** as a colorless oil (35 mg, 61% yield).

Formula: $C_{15}H_{12}F_2$

Molecular weight: 230.26

Isolated yield: 61% (35 mg) as a colorless oil

Rf: 0.4 (Pentane/EtOAc = 100:3)

^{1}H NMR (400 MHz, CDCl$_3$, 298 K) δ 7.48 – 7.35 (m, 1H), 7.25 – 7.13 (m, 3H), 7.11 – 6.97 (m, 4H), 6.62 (d, *J* = 15.9 Hz, 1H), 6.41 (dt, *J* = 15.9, 6.9 Hz, 1H), 3.59 (d, *J* = 6.9 Hz, 2H)

^{13}C NMR (101 MHz, CDCl$_3$, 298 K) δ 161.1 (d, *J* = 245.5 Hz), 160.2 (d, *J* = 249.6 Hz), 130.8 (d, *J* = 4.7 Hz), 130.4 (d, *J* = 4.3 Hz), 128.5 (d, *J* = 8.4 Hz), 128.2 (d, *J* = 8.0 Hz), 127.4 (d, *J* = 3.9 Hz), 127.0 (d, *J* = 15.8 Hz), 125.2 (d, *J* = 12.3 Hz), 124.3 (d, *J* = 3.6 Hz), 124.1 (d, *J* = 3.5 Hz), 124.0 (d, *J* = 3.7 Hz), 115.8 (d, *J* = 22.2 Hz), 115.5 (d, *J* = 22.0 Hz), 32.8 (d, *J* = 3.0 Hz)

^{19}F NMR (376 MHz, CDCl$_3$, 298 K) δ -118.4, -118.5

(*E*)-4,4'-(Prop-1-ene-1,3-diyl)bis(chlorobenzene) (4h)[17]

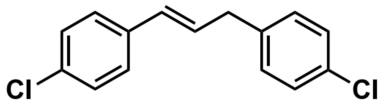

Prepared according to a modified general procedure using **1h** (69.3 mg, 0.25 mmol) at 100 °C. The crude product was purified by flash column chromatography on silica gel (pentane/EtOAc = 100:3) to afford **4h** as a white solid (55 mg, 84% yield).

Formula: $C_{15}H_{12}Cl_2$

Molecular weight: 263.16

Isolated yield: 84% (55 mg) as a white solid

Rf: 0.4 (Pentane/EtOAc = 100:3)

^{1}H NMR (400 MHz, CDCl$_3$, 298 K) δ 7.32 – 7.25 (m, 6H), 7.17 (d, *J* = 8.3 Hz, 2H), 6.39 (d, *J* = 15.8 Hz, 1H), 6.30 (dt, *J* = 15.8, 6.5 Hz, 1H), 3.51 (d, *J* = 6.5 Hz, 2H)

^{13}C NMR (101 MHz, CDCl$_3$, 298 K) δ 138.4, 135.9, 133.0, 132.2, 130.4, 130.1, 129.5, 128.8, 128.8, 127.5, 38.7

(*E*)-4,4''-(Prop-1-ene-1,3-diyl)di-1,1'-biphenyl (4i)[19]

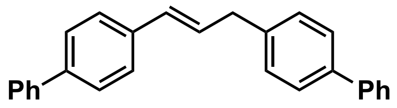

Prepared according to a modified general procedure using **1i** (90.1 mg, 0.25 mmol) at 100 °C. The crude product was purified by flash column chromatography on silica gel (pentane/EtOAc = 25:1) to afford **4i** as a white solid (65 mg, 75% yield).

Formula: $C_{27}H_{22}$

Molecular weight: 346.47

Isolated yield: 75% (65 mg) as a white solid

Rf: 0.4 (Pentane/EtOAc = 25:1)

^{1}H NMR (400 MHz, $CDCl_3$, 298 K) δ 7.63 – 7.54 (m, 8H), 7.48 – 7.41 (m, 6H), 7.37 – 7.31 (m, 4H), 6.54 (d, *J* = 15.8 Hz, 1H), 6.44 (dt, *J* = 15.8, 6.6 Hz, 1H), 3.63 (d, *J* = 6.6 Hz, 2H)

^{13}C NMR (101 MHz, $CDCl_3$, 298 K) δ 141.2, 140.9, 140.1, 139.4, 136.6, 130.9, 129.4, 129.3, 128.9, 128.9, 127.4, 127.4, 127.3,127.2, 127.1, 126.7, 39.2

(*E*)-2,2'-(prop-1-ene-1,3-diyl)dinaphthalene (4j)[19]

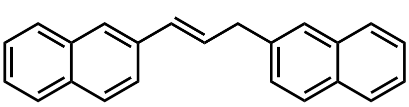

Prepared according to the general procedure using **1j** (154.2 mg, 0.50 mmol). The crude product was purified by flash column chromatography on silica gel (pentane/DCM = 5:1) to afford **4j** as a white solid (125 mg, 85% yield).

Formula: $C_{23}H_{18}$

Molecular weight: 294.40

Isolated yield: 85% (125 mg) as a white solid

Rf: 0.3 (Pentane/DCM = 5:1)

^{1}H NMR (400 MHz, $CDCl_3$, 298 K) δ 7.87 – 7.78 (m, 6H), 7.74 (s, 2H), 7.66 – 7.60 (m, 1H), 7.51 – 7.43 (m, 5H), 6.69 (d, *J* = 15.9 Hz, 1H), 6.60 (dt, *J* = 15.9, 6.4 Hz, 1H), 3.80 (d, *J* = 6.4 Hz, 2H)

^{13}C NMR (101 MHz, $CDCl_3$, 298 K) δ 137.7, 135.0, 133.8, 133.8, 132.9, 132.3, 131.5, 129.7, 128.3, 128.2, 128.0, 127.8, 127.8, 127.7, 127.6, 127.0, 126.3, 126.2, 125.9, 125.8, 125.5, 123.7, 39.7

Dimethyl 4,4'-(prop-1-ene-1,3-diyl)(*E*)-dibenzoate (4l)

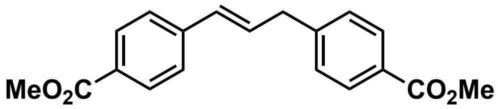

Prepared according to a modified general procedure using **1l** (81.1 mg, 0.25 mmol) with 5 mol% of **C1** at 120 °C. The crude product was purified by flash column chromatography on silica gel (pentane/EtOAc = 5:1) to afford **4l** as a white solid (48 mg, 62% yield).

Formula: $C_{19}H_{18}O_4$

Molecular weight: 310.35

Isolated yield: 62% (48 mg) as a white solid

Rf: 0.3 (Pentane/EtOAc = 5:1)

^{1}H NMR (400 MHz, CDCl$_3$, 298 K) δ 8.04 – 7.09 (m, 4H), 7.40 (d, *J* = 8.3 Hz, 2H), 7.30 (d, *J* = 8.3 Hz, 2H), 6.55 – 6.37 (m, 2H), 3.91 (s, 3H), 3.90 (s, 3H), 3.62 (d, *J* = 5.0 Hz, 2H)

^{13}C NMR (101 MHz, CDCl$_3$, 298 K) δ 167.1, 167.0, 145.1, 141.8, 131.1, 131.0, 130.0, 130.0, 128.9, 128.8, 128.5, 126.1, 52.2, 39.4

HRMS (ESI$^+$): Calcd. for $C_{19}H_{18}O_4Na^+$ $[M+Na]^+$: 333.10973; Found 333.10950

1-Cinnamyl-4-methylbenzene (4m)

(*E*)-1-Methyl-4-(3-phenylprop-1-en-1-yl)benzene (4m')[17]

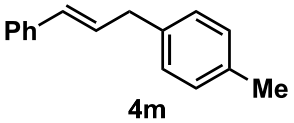

4m

\+

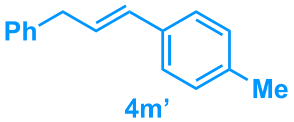

4m'

Prepared according to the general procedure using **1m** (111.1 mg, 0.50 mmol). The crude product was purified by flash column chromatography on silica gel (pentane/EtOAc = 50:1) to afford a mixture of **4m** and **4m'** (58:42 ratio) as a colorless oil (97 mg, 93% yield).

Ratio 4m:4m' = 58:42

Formula: $C_{16}H_{16}$

Molecular weight: 208.30

Isolated yield: 93% (97 mg) as a colorless oil

Rf: 0.4 (Pentane/EtOAc = 50:1)

^{1}H NMR (400 MHz, CDCl$_3$, 298 K) δ 7.34 – 7.05 (m, 9H), 6.41 (d, *J* = 15.8 Hz, 0.58 × 1H), 6.40 (d, *J* = 15.8 Hz, 0.42 × 1H), 6.36 – 6.21 (m, 1H), 3.50 (d, *J* = 6.8 Hz, 0.42 ×2H), 3.47 (d, *J* = 6.6 Hz, 0.58 ×2H), 2.30 (s, 0.58 × 3H), 2.29 (s, 0.42 × 3H)

^{13}C NMR (101 MHz, CDCl$_3$, 298 K) δ 140.5, 137.7, 137.2, 136.9, 135.8, 134.8, 131.0, 131.0, 129.6, 129.3, 129.3, 128.8, 128.7, 128.6, 128.6, 128.3, 127.1, 126.3, 126.2, 126.1, 39.5, 39.1, 21.3, 21.2

1-Cinnamyl-4-methoxybenzene (4n)

(*E*)-1-Methoxy-4-(3-phenylprop-1-en-1-yl)benzene (4n')[17]

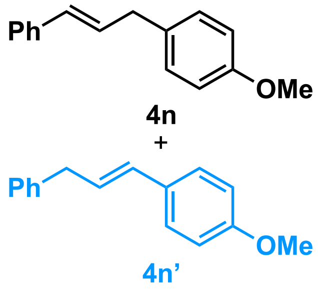

Prepared according to the general procedure using **1n** (119.2 mg, 0.50 mmol). The crude product was purified by flash column chromatography on silica gel (pentane/EtOAc = 25:1) to afford a mixture of **4n** and **4n'** (54:46 ratio) as a colorless oil (108 mg, 96% yield).

Ratio 4n:4n' = 54:46

Formula: $C_{16}H_{16}O$

Molecular weight: 224.30

Isolated yield: 96% (108 mg) as a colorless oil

Rf: 0.4 (Pentane/EtOAc = 25:1)

^{1}H NMR (400 MHz, $CDCl_3$, 298 K) δ 7.33 – 7.05 (m, 7H), 6.84 – 6.71 (m, 2H), 6.41 – 6.22 (m, 0.54 ×2H + 0.46 ×1H), 6.20 – 6.09 (m, 0.46 ×1H), 3.75 – 3.66 (m, 3H), 3.46 (d, *J* = 6.9 Hz, 0.46 ×2H), 3.42 (d, *J* = 6.5 Hz, 0.54 ×2H)

^{13}C NMR (101 MHz, $CDCl_3$, 298 K) δ 159.0, 158.2, 140.6, 137.6, 132.3, 130.8, 130.5, 130.4, 129.8, 129.7, 128.8, 128.6, 128.6, 127.3, 127.2, 127.1, 126.2, 114.0, 114.0, 55.4, 55.4, 39.4, 38.6

1-Cinnamyl-4-fluorobenzene (4q)

(*E*)-1-fluoro-4-(3-phenylprop-1-en-1-yl)benzene (4q')[17]

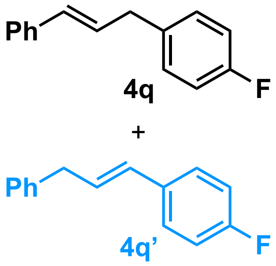

Prepared according to the general procedure using **1q** (113.1 mg, 0.50 mmol). The crude product was purified by flash column chromatography on silica gel (pentane/EtOAc = 50:1) to afford a mixture of **4q** and **4q'** (40:60 ratio) as a colorless oil (88 mg, 83% yield).

Ratio 4q:4q' = 40:60

Formula: $C_{15}H_{13}F$

Molecular weight: 212.27

Isolated yield: 83% (88 mg) as a colorless oil

Rf: 0.4 (Pentane/EtOAc = 50:1)

^{1}H NMR (400 MHz, $CDCl_3$, 298 K) δ 7.35 – 7.13 (m, 7H), 7.01 – 6.91 (m, 2H), 6.46 – 6.35 (m, 1H), 6.35 – 6.19 (m, 1H), 3.54 – 3.45 (m, 2H)

^{13}C NMR (101 MHz, $CDCl_3$, 298 K) δ 162.2 (d, *J* = 246.0 Hz), 161.6 (d, *J* = 244.8 Hz), 140.2, 137.4, 135.9 (d, *J* = 3.2 Hz), 133.7 (d, *J* = 3.3 Hz), 131.3, 130.2 (d, *J* = 7.8 Hz), 130.0, 129.1, 129.1,

128.8, 128.7, 128.7, 127.7 (d, *J* = 7.9 Hz), 127.3, 126.4, 126.3, 115.5 (d, *J* = 21.5 Hz), 115.3 (d, *J* = 21.3 Hz), 39.4, 38.6

^{19}F NMR (376 MHz, CDCl$_3$, 298 K) δ -115.2, -117.1.

1-Chloro-4-cinnamylbenzene (4r)

(*E*)-1-Chloro-4-(3-phenylprop-1-en-1-yl)benzene (4r')[20]

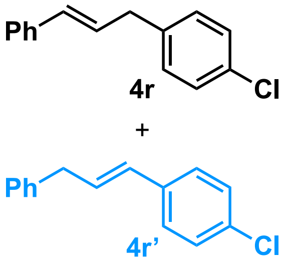

Prepared according to the general procedure using **1r** (121.4 mg, 0.50 mmol). The crude product was purified by flash column chromatography on silica gel (pentane/EtOAc = 50:1) to afford a mixture of **4r** and **4r'** (44:56 ratio) as a colorless oil (80 mg, 70% yield).

Ratio 4r:4r' = 44:56

Formula: $C_{15}H_{13}Cl$

Molecular weight: 228.72

Isolated yield: 70% (80 mg) as a colorless oil

Rf: 0.4 (Pentane/EtOAc = 50:1)

^{1}H NMR (400 MHz, CDCl$_3$, 298 K) δ 7.33 – 7.08 (m, 9H), 6.44 – 6.18 (m, 2H), 3.49 (d, *J* = 6.0 Hz, 0.56 ×2H), 3.45 (d, *J* = 6.5 Hz, 0.44 ×2H)

^{13}C NMR (101 MHz, CDCl$_3$, 298 K) δ 140.0, 138.7, 137.4, 136.1, 132.8, 132.1, 131.6, 130.1, 129.9, 128.8, 128.7, 128.7, 128.7, 128.6, 127.4, 127.4, 126.4, 126.3, 39.4, 38.7

1-Cinnamyl-4-(trifluoromethyl)benzene (4s)

(*E*)-1-(3-Phenylprop-1-en-1-yl)-4-(trifluoromethyl)benzene (4s')[17]

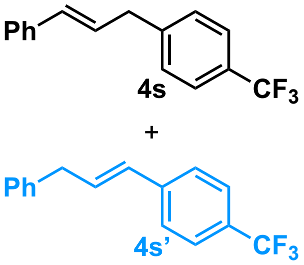

Prepared according to the general procedure using **1s** (138.1 mg, 0.50 mmol). The crude product was purified by flash column chromatography on silica gel (pentane/EtOAc = 50:1) to afford a mixture of **4s** and **4s'** (36:64 ratio) as a colorless oil (81 mg, 62% yield).

Ratio 4s:4s' = 36:64

Formula: $C_{16}H_{13}F_3$

Molecular weight: 262.27

Isolated yield: 62% (81 mg) as a colorless oil

Rf: 0.3 (Pentane/EtOAc = 50:1)

^{1}H NMR (400 MHz, CDCl$_3$, 298 K) δ 7.60 – 7.53 (m, 2H), 7.45 (d, J = 8.2 Hz, 0.64 ×2H), 7.39 – 7.23 (m, 5H + 0.36 ×2H), 6.60 – 6.42 (m, 0.36 ×1H + 0.64 ×2H), 6.34 (dt, J = 15.7, 6.8 Hz, 0.36 ×1H), 3.65 – 3.57 (m, 2H)

^{13}C NMR (101 MHz, CDCl$_3$, 298 K) δ 144.4, 141.1, 139.7, 137.2, 132.3, 132.1, 129.9, 129.1, 128.8, 128.7, 128.7, 128.7 (q, J = 32.2 Hz), 128.7 (q, J = 32.5 Hz), 128.0, 127.5, 126.5, 126.4, 126.3, 125.6 (q, J = 3.9 Hz), 125.5 (q, J = 3.9 Hz), 124.5 (q, J = 272.8 Hz), 124.4 (q, J = 272.7 Hz), 39.5, 39.2

^{19}F NMR (376 MHz, CDCl$_3$, 298 K) δ -62.3, -62.4

Methyl 4-cinnamylbenzoate (4t)[21]

Methyl (*E*)-4-(3-phenylprop-1-en-1-yl)benzoate (4t')[22]

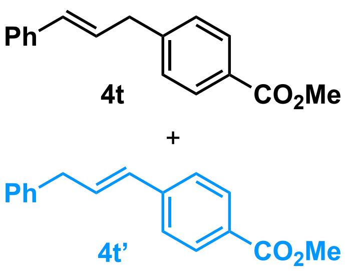

Prepared according to the general procedure using **1t** (66.6 mg, 0.25 mmol). The crude product was purified by flash column chromatography on silica gel (pentane/EtOAc = 20:1) to afford a mixture of **4t** and **4t'** (55:45 ratio) as a colorless oil (44 mg, 70% yield).

Ratio 4t:4t' = 55:45

Formula: $C_{17}H_{16}O_2$

Molecular weight: 252.31

Isolated yield: 70% (44 mg) as a colorless oil

Rf: 0.3 (Pentane/EtOAc = 20:1)

^{1}H NMR (400 MHz, CDCl$_3$, 298 K) δ 8.02-7.90 (m, 2H), 7.43 – 7.26 (m, 5H), 7.24 – 7.17 (m, 2H), 6.50 – 6.40 (m, 0.45 ×2H + 0.55× 1H), 6.31 (dt, J = 16.0, 6.9 Hz, 0.55 ×1H), 3.89 (s, 0.55 ×3H), 3.88 (s, 0.45 ×3H), 3.61 – 3.52 (m, 2H)

^{13}C NMR (101 MHz, CDCl$_3$, 298 K δ 167.2, 167.1, 145.7, 142.1, 139.7, 137.3, 132.3, 131.9, 130.3, 130.0, 130.0, 128.8, 128.7, 128.7, 128.3, 128.2, 127.5, 126.5, 126.3, 126.1, 52.2, 39.6, 39.4

***N*-(4-cinnamylphenyl)acetamide (4u)**[23]

(*E*)-*N*-(4-(3-phenylprop-1-en-1-yl)phenyl)acetamide (4u')[24]

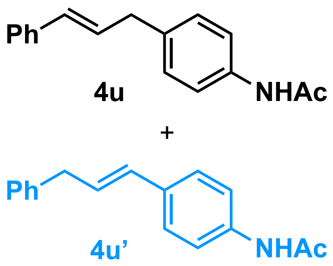

Prepared according to the general procedure using **1u** (132.7 mg, 0.50 mmol). The crude product was purified by flash column chromatography on silica gel (pentane/EtOAc = 1:2) to afford a mixture of **4u** and **4u'** (49:51 ratio) as a white solid (83 mg, 66% yield).

Ratio 4u:4u' = 49:51

Formula: $C_{17}H_{17}NO$

Molecular weight: 251.33

Isolated yield: 66% (83 mg) as a white solid

Rf: 0.4 (Pentane/EtOAc = 1:2)

^{1}H NMR (400 MHz, CDCl$_3$, 298 K) δ 7.44 (d, *J* = 8.4 Hz, 2H), 7.37 – 7.15 (m, 8H), 6.49 – 6.21 (m, 2H), 3.54 (d, *J* = 6.7 Hz, 0.51 ×2H), 3.50 (d, *J* = 6.6 Hz, 0.49 ×2H), 2.15 (s, 0.55 ×3H), 2.15 (s, 0.49 ×3H)

^{13}C NMR (101 MHz, CDCl$_3$, 298 K) δ 168.6, 168.6, 140.3, 137.5, 137.0, 136.3, 136.2, 133.7, 131.1, 130.4, 129.3, 128.8, 128.8, 128.6, 128.6, 128.4, 127.2, 126.7, 126.3, 126.2, 120.4, 120.0, 39.4, 38.8, 24.7, 24.6

2-Cinnamylnaphthalene (4v)[25]

(*E*)-2-(3-Phenylprop-1-en-1-yl)naphthalene (4v')[22]

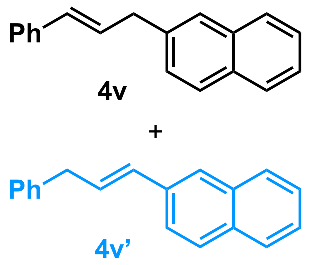

Prepared according to the general procedure using **1v** (129.2 mg, 0.50 mmol). The crude product was purified by flash column chromatography on silica gel (pentane/EtOAc = 50:1) to afford a mixture of **4v** and **4v'** (57:43 ratio) as a white solid (110 mg, 90% yield).

Ratio 4v:4v' = 57:43

Formula: $C_{19}H_{16}$

Molecular weight: 244.34

Isolated yield: 90% (110 mg) as a white solid

Rf: 0.4 (Pentane/EtOAc = 50:1)

^{1}H NMR (400 MHz, CDCl$_3$, 298 K) δ 7.82 – 7.71 (m, 3H), 7.67 (d, *J* = 5.1 Hz, 1H), 7.49 – 7.16 (m, 8H), 6.60 (d, *J* = 16.8 Hz, 0.43 ×1H), 6.53 – 6.36 (m, 0.43 ×1H + 0.57 ×2H), 3.69 (d, *J* = 6.2 Hz, 0.57 ×2H), 3.59 (d, *J* = 6.7 Hz, 0.43 ×2H)

^{13}C NMR (101 MHz, CDCl$_3$, 298 K) δ 140.3, 137.8, 137.6, 135.0, 133.8, 133.8, 132.9, 132.3, 131.4, 131.3, 129.8, 129.2, 128.9, 128.7, 128.7, 128.2, 128.2, 128.0, 127.8, 127.8, 127.6, 127.6, 127.3, 126.9, 126.4, 126.3, 126.3, 126.1, 125.9, 125.7, 125.5, 123.7, 39.6, 39.6

2-Cinnamylthiophene (4w) [25]

(*E*)-2-(3-Phenylprop-1-en-1-yl)thiophene (4w')[22]

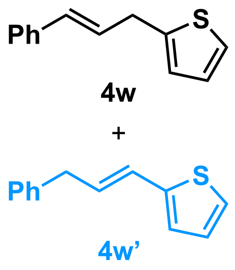

Prepared according to the general procedure using **1w** (107.2 mg, 0.50 mmol). The crude product was purified by flash column chromatography on silica gel (pentane/EtOAc = 50:1) to afford a mixture of **4w** and **4w'** (52:48 ratio) as a brown oil (76 mg, 76% yield).

Ratio 4w:4w' = 52:48

Formula: $C_{13}H_{12}S$

Molecular weight: 200.30

Isolated yield: 76% (76 mg) as a brown oil

Rf: 0.4 (Pentane/EtOAc = 50:1)

^{1}H NMR (400 MHz, $CDCl_3$, 298 K) δ 7.37 – 7.25 (m, 3H), 7.24 – 7.17 (m, 2H), 7.14 (dd, *J* = 5.1, 1.1 Hz, 0.52 ×1H), 7.06 (d, *J* = 5.0 Hz, 0.48 ×1H), 6.97 – 6.80 (m, 2H), 6.60 – 6.42 (m, 1H), 6.34 (dt, *J* = 15.7, 6.8 Hz, 0.52 ×1H), 6.19 (dt, *J* = 15.6, 6.8 Hz, 0.48 ×1H), 3.71 (d, *J* = 6.8 Hz, 0.52 ×2H), 3.49 (d, *J* = 6.8 Hz, 0.48 ×2H)

^{13}C NMR (101 MHz, $CDCl_3$, 298 K) δ 143.2, 142.8, 139.9, 137.3, 131.5, 129.3, 128.8, 128.7, 128.6, 128.3, 127.4, 127.4, 127.1, 126.4, 126.4, 124.9, 124.8, 124.4, 123.9, 123.6, 39.2, 33.5

(*E*)-(3-Cyclohexyl-3-isopropoxyprop-1-en-1-yl)benzene (4x)

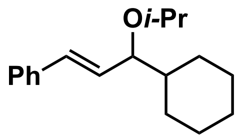

Prepared from either **1x** (107.1 mg, 0.50 mmol) or **1y** (107.1 mg, 0.50 mmol). The crude product was purified by flash column chromatography on silica gel (pentane/EtOAc/Et_3N = 100:2:1) to afford **4x** as a colorless oil (65 mg, 50% yield from **1x**; 97 mg, 75% yield from **1y**).

Formula: $C_{18}H_{26}O$

Molecular weight: 258.41

Isolated yield: 50% (65 mg) from **1x**, 75% (97 mg) from **1y**, as a colorless oil

Rf: 0.3 (Pentane/EtOAc/Et_3N = 100:2:1)

^{1}H NMR (400 MHz, $CDCl_3$, 298 K) δ 7.38 (d, *J* = 7.3 Hz, 2H), 7.33 – 7.27 (m, 2H), 7.24 – 7.20 (m, 1H), 6.44 (d, *J* = 16.0 Hz, 1H), 6.09 (dd, *J* = 16.0, 7.9 Hz, 1H), 3.80 – 3.49 (m, 2H), 1.99 (d, *J* = 12.7 Hz, 1H), 1.75 – 1.62 (m, 4H), 1.50 – 1.41 (m, 1H), 1.28 – 1.10 (m, 9H), 1.03 – 0.91 (m, 2H)

^{13}C NMR (101 MHz, $CDCl_3$, 298 K) δ 137.1, 131.6, 131.0, 128.7, 127.5, 126.5, 83.1, 68.8, 43.0, 29.7, 29.4, 26.8, 26.3, 26.2, 23.7, 21.6

HR-MS (ESI): Calcd. for $C_{18}H_{26}ONa^+$ $[M+Na]^+$: 281.18759, found 281.18780.

7.2 Compounds monitored by crude ¹H NMR

The following compounds were monitored by crude ¹H NMR due to low conversion or loss during purification, attributed to volatility and/or instability under the purification conditions. Yields were estimated from the crude reaction mixtures using mesitylene as an internal standard.

(*E*)-4,4'-(prop-1-ene-1,3-diyl)bis(methoxybenzene) (4d)

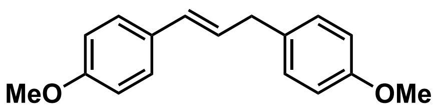

Prepared according to a modified general procedure using **1d** (134.1 mg, 0.50 mmol) at 40 °C in MeOH. The reaction mixture was analyzed by ¹H NMR spectroscopy, giving **4d** in 45% NMR yield (no purification performed).

Formula: $C_{17}H_{18}O_2$

Molecular weight: 254.33

NMR yield: 45%

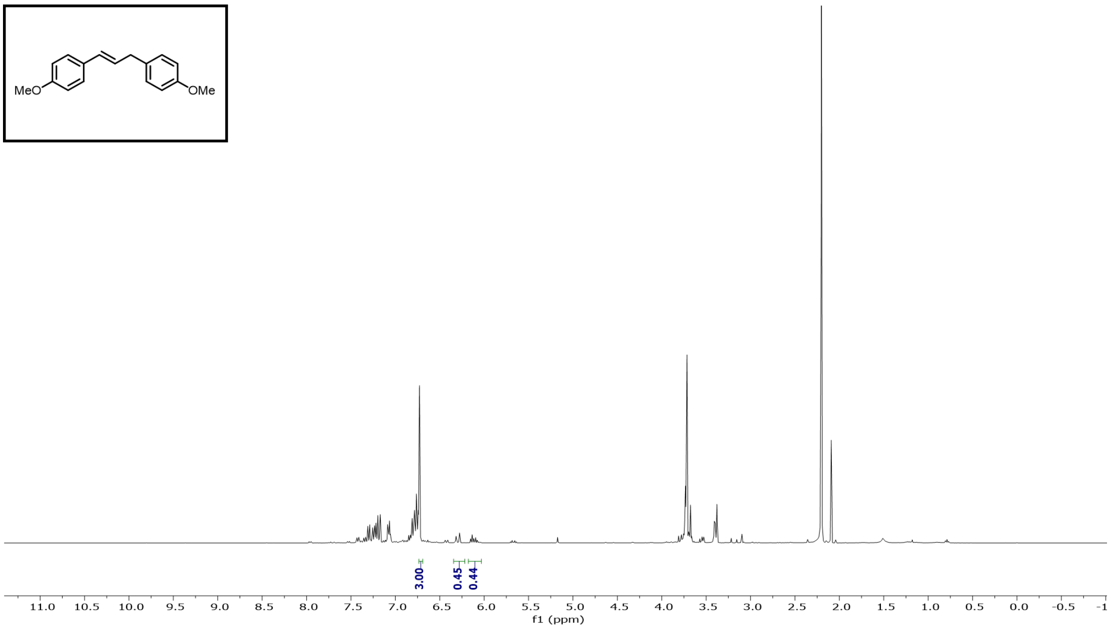

Figure S6 – ¹H NMR (400 MHz, CDCl₃, 298 K) spectrum of the crude reaction mixture showing **4d**.

(*E*)-4,4'-(prop-1-ene-1,3-diyl)bis((trifluoromethyl)benzene) (4k)

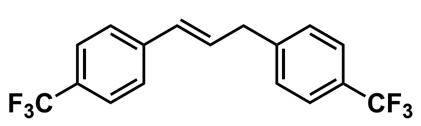

Prepared according to a modified general procedure using **1k** (62.5 mg, 0.25 mmol) with 5 mol% of **C1** at 120 °C. The reaction mixture was analyzed by ^{1}H NMR spectroscopy, giving **4k** in 26% NMR yield (no purification performed).

Formula: $C_{17}H_{18}O_2$

Molecular weight: 254.33

NMR yield: 26%

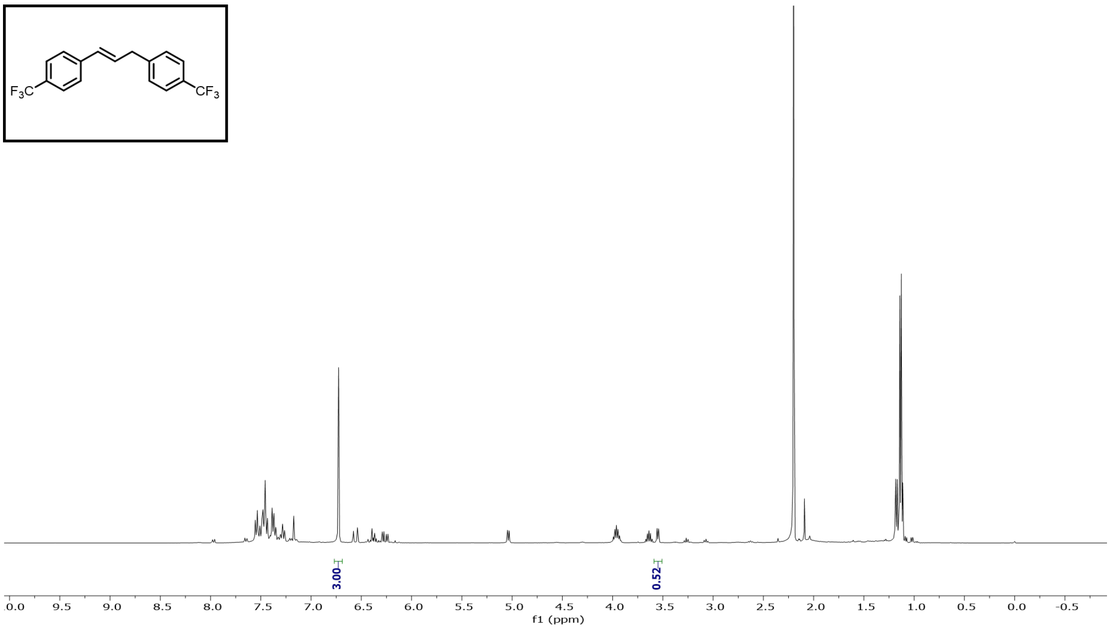

Figure S7 – ^{1}H NMR (400 MHz, CDCl$_3$, 298 K) spectrum of the crude reaction mixture showing **4k**.

8. *Gram-scale* Procedure for the Synthesis of 2n, 3a, and 4c

8.1 Synthesis of 2n

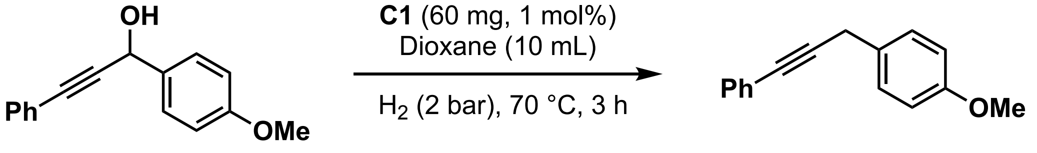

1n
(4.5 mmol)

2n
NMR yield: 80%
Isolated yield: 75% (0.75 g)

In an oven-dried Fisher–Porter tube under an argon atmosphere, propargylic alcohol **1n** (1.07 g, 4.50 mmol), complex **C1** (60 mg, 1 mol%), and dioxane (10 mL) were added. The mixture was flash-frozen using liquid nitrogen, evacuated, and then pressurized with H_2 (2 bar). The sealed tube was stirred at 70 °C for 3 h. *(Note: the reaction was paused after 1 h and the Fisher–Porter tube was refilled with H_2 to 2 bar.)* After completion, the reaction was cooled to room temperature, and the pressure was carefully released. The solvent was removed under reduced pressure. Conversion and yield were determined by ^{1}H NMR spectroscopy using mesitylene (4.50 mmol) as an internal standard. Purification by flash column chromatography on silica gel (EtOAc/pentane/Et_3N = 100:3:1) afforded **2n** (0.75 g) as the pure product.

8.2 Synthesis of 3a

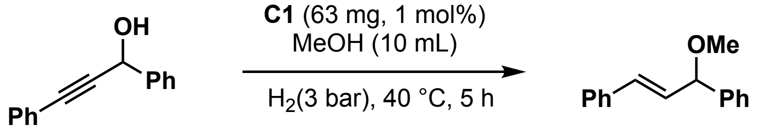

1a
(4.75 mmol)

3a
NMR yield: 92%
Isolated yield: 87% (0.93 g)

In an oven-dried Fisher–Porter tube under an argon atmosphere, propargylic alcohol **1a** (0.99 g, 4.75 mmol), complex **C1** (63 mg, 1 mol%), and MeOH (10 mL) were added. The mixture was flash-frozen using liquid nitrogen, evacuated, and then pressurized with H_2 (3 bar). The sealed tube was stirred at 40 °C for 5 h. *(Note: The reaction was paused after 3 h, and the Fisher–Porter tube was refilled with H_2 to 3 bar.)* After completion, the reaction was cooled to room temperature, and the pressure was carefully released. The solvent was removed under reduced pressure. Conversion and yield were determined by ^{1}H NMR spectroscopy using mesitylene (4.75 mmol) as an internal standard. Purification by flash column chromatography on silica gel (EtOAc/pentane/Et_3N = 100:3:1) afforded **3a** (0.93 g) as the pure product.

8.3 Synthesis of 4c

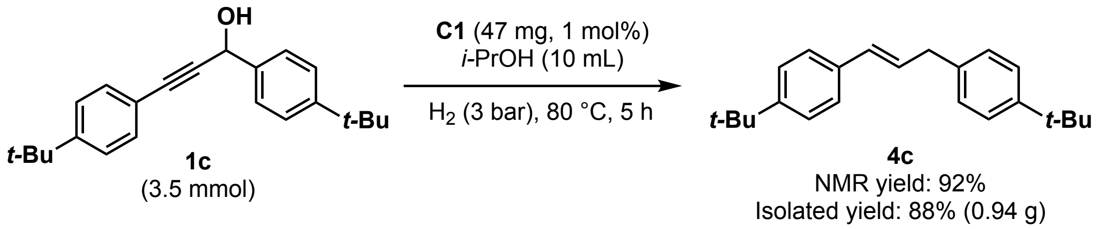

In an oven-dried Fisher–Porter tube under an argon atmosphere, propargylic alcohol **1c** (1.12 g, 3.50 mmol), complex **C1** (47 mg, 1 mol%), and *i*-PrOH (10 mL) were added. The mixture was flash-frozen using liquid nitrogen, evacuated, and then pressurized with H_2 (3 bar). The sealed tube was stirred at 80 °C for 5 h. *(Note: The reaction was paused after 2 h, and the Fisher–Porter tube was refilled with H_2 to 3 bar.)* After completion, the reaction was cooled to room temperature, and the pressure was carefully released. The solvent was removed under reduced pressure. Conversion and yield were determined by ^{1}H NMR spectroscopy using mesitylene (3.50 mmol) as an internal standard. Purification by flash column chromatography on silica gel (EtOAc/pentane = 100:3) afforded **4c** (0.94 g) as the pure product.

9. Additional Tests with Cyano-, Ketone-, and Nitro-Substituted Propargylic Alcohols

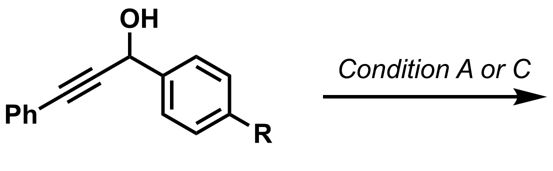

R = CN, COMe, or NO_2

Experimental Procedure: Reactions were conducted according to the general procedure for the preparation of alkyne **2** (*Condition A*) or *trans*-alkene **4** (*Condition C*), substituting the test substrate (cyano-, ketone-, or nitro-containing propargylic alcohol) for **1a**. Each reaction was performed on a 0.50 mmol scale using catalyst **C1** (1 mol%) and the corresponding solvent and H_2 pressure as described in the general procedures. After the designated reaction time, the mixture was worked up as per the general procedure. Conversions and yields were determined by ^{1}H NMR spectroscopy with mesitylene (0.50 mmol) as an internal standard.

For the cyano substrate, <1% conversion was observed under both conditions, which may reflect catalyst deactivation by coordination. The ketone substrate afforded only ether products (20% under *Condition A*; 12% *i*-Pr ether under *Condition C*), with no hydrogenation products. For the nitro substrate, no desired hydrogenation products (**2**/**3**/**4**) were obtained under either condition; instead, competing nitroarene hydrogenation was observed.

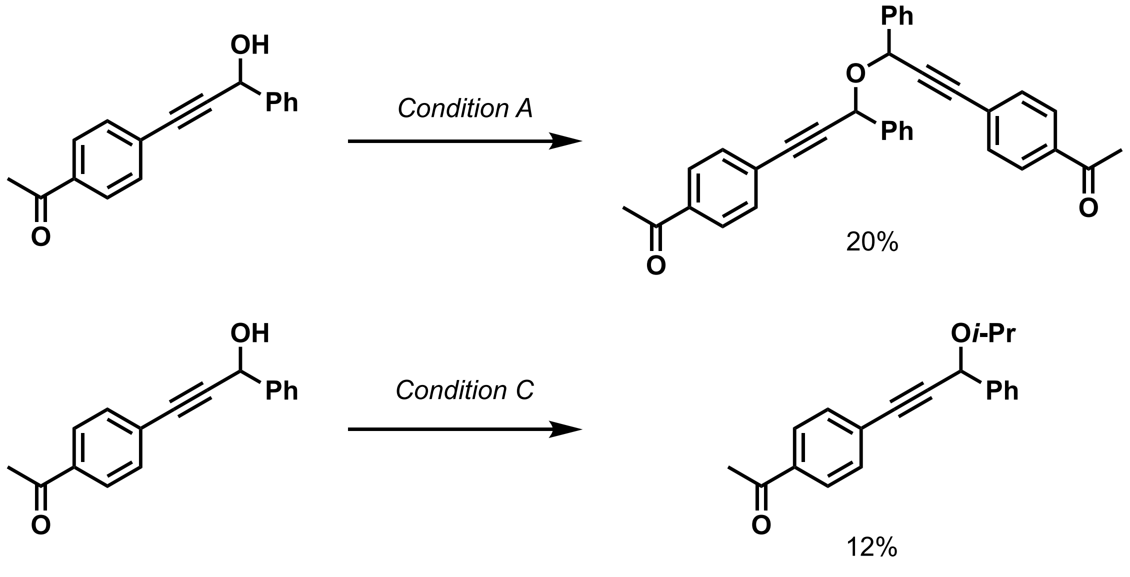

10. Catalyst Screening under Optimized Conditions

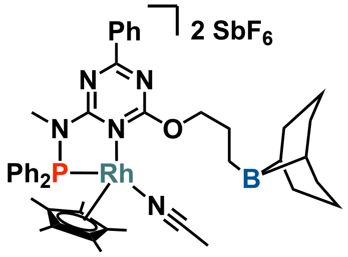

C1

Complex **C1** was evaluated under three optimized sets of conditions corresponding to alkyne reduction (*Condition A*), allyl ether synthesis (*Condition B*), and alkene synthesis (*Condition C*).

Results (NMR yields):

Condition A: **2a:** 76%

Condition B: **3a:** 90%; **4a:** 2%

Condition C: **4a** 92%

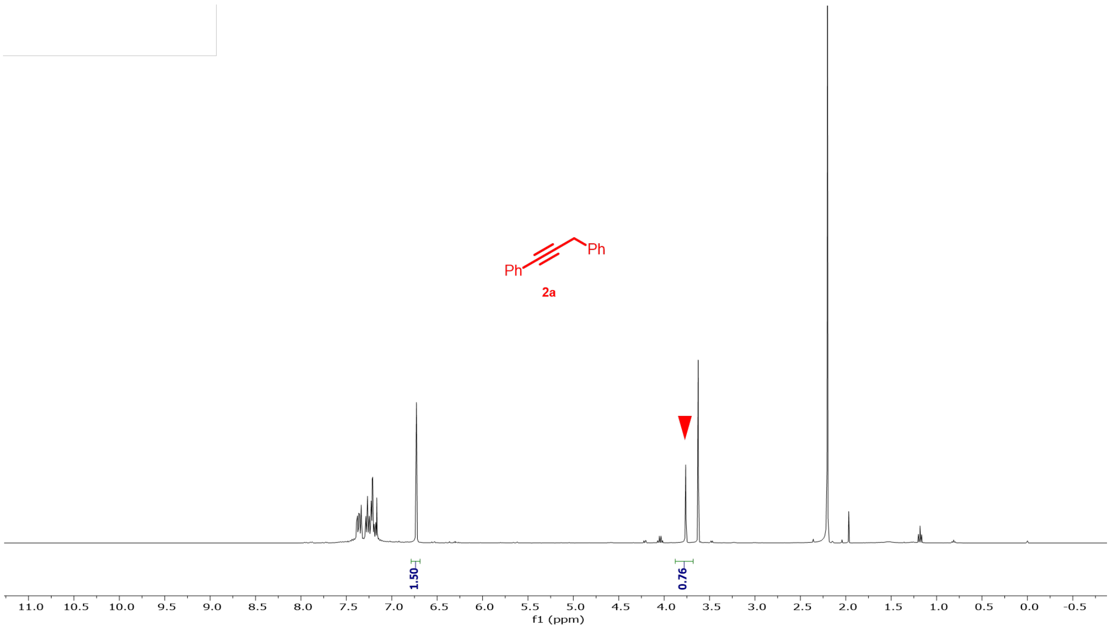

Figure S8 – ^{1}H NMR (400 MHz, $CDCl_3$, 298 K) spectrum of the crude reaction mixture obtained under *Condition A* using **C1** as a catalyst.

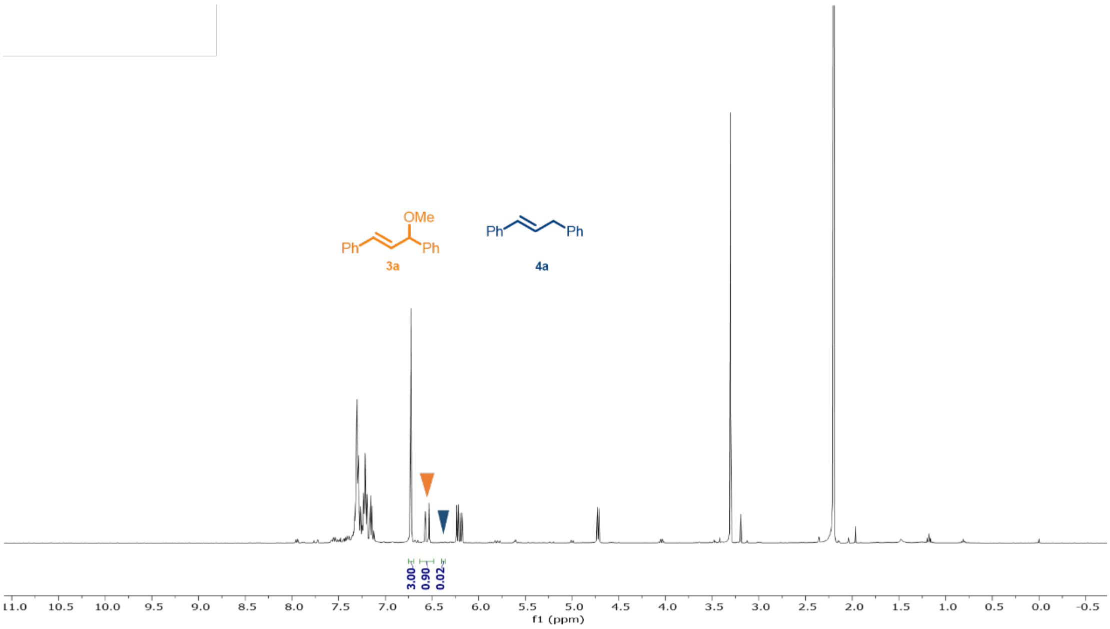

Figure S9 – ^{1}H NMR (400 MHz, $CDCl_3$, 298 K) spectrum of the crude reaction mixture obtained under *Condition B* using **C1** as a catalyst.

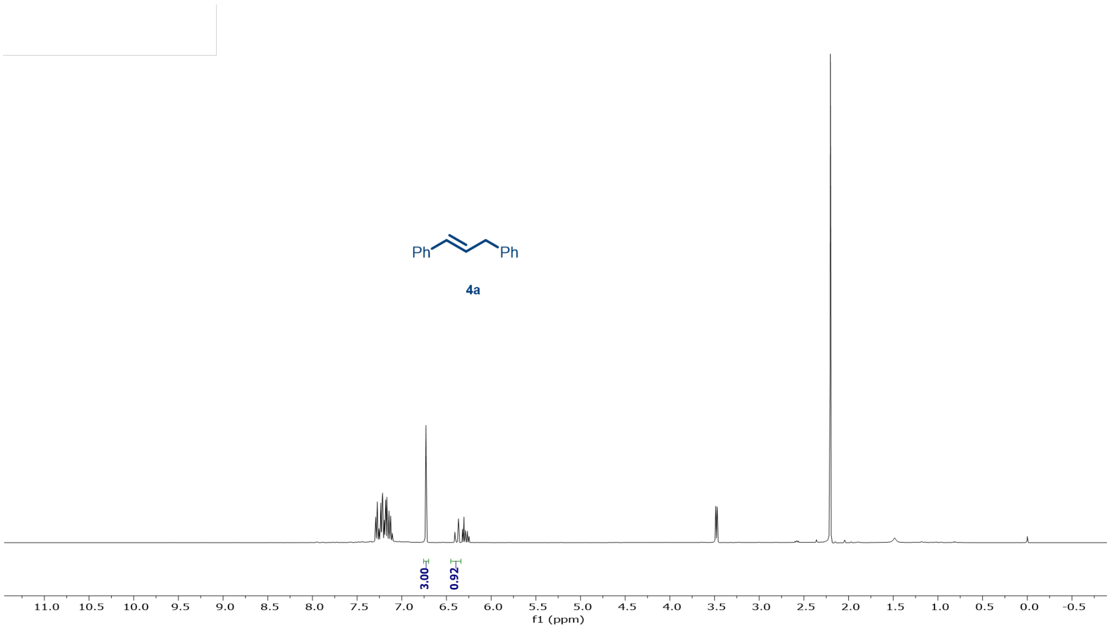

Figure S10 – ^{1}H NMR (400 MHz, $CDCl_3$, 298 K) spectrum of the crude reaction mixture obtained under *Condition C* using **C1** as a catalyst.

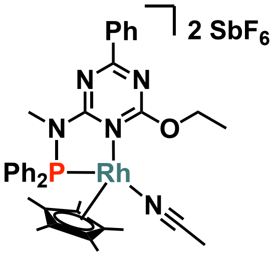

C2

Complex **C2** was evaluated under three optimized sets of conditions corresponding to alkyne reduction (*Condition A*), allyl ether synthesis (*Condition B*), and alkene synthesis (*Condition C*).

Results (NMR yields):

Condition A: **1a**: 21%; **2a**: 30%; **5a-1**: 49%

Condition B: **3a:** 90%; **3a-2:** 3%

Condition C: **3a-1:** 16%; **3a-2:** 4%; **4a:** 72%

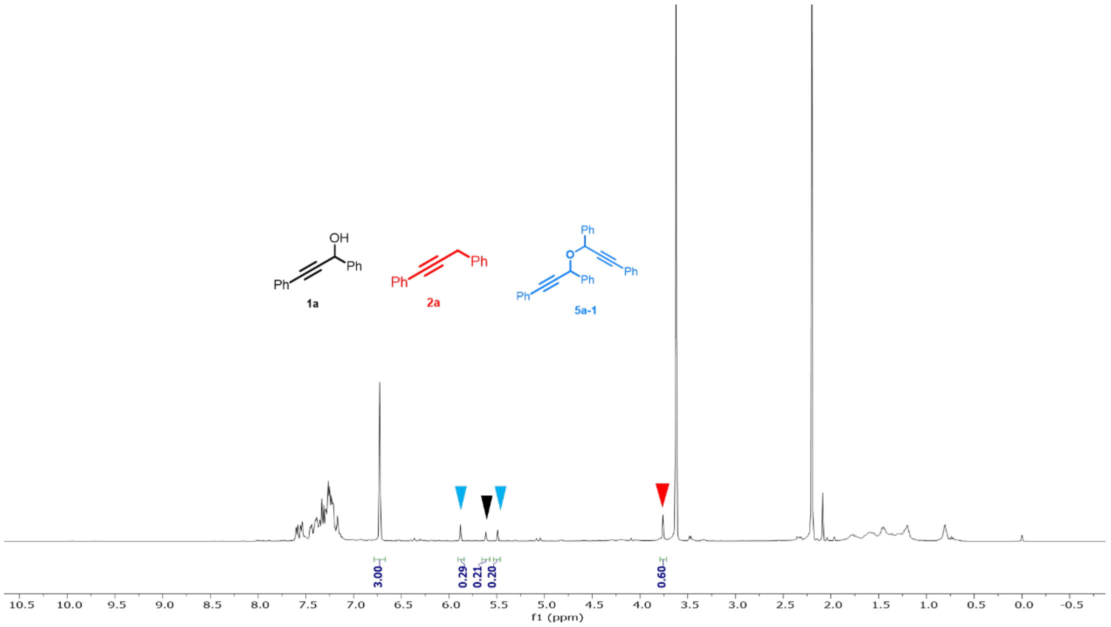

Figure S11 – ^{1}H NMR (400 MHz, $CDCl_3$, 298 K) spectrum of the crude reaction mixture obtained under *Condition A* using **C2** as a catalyst.

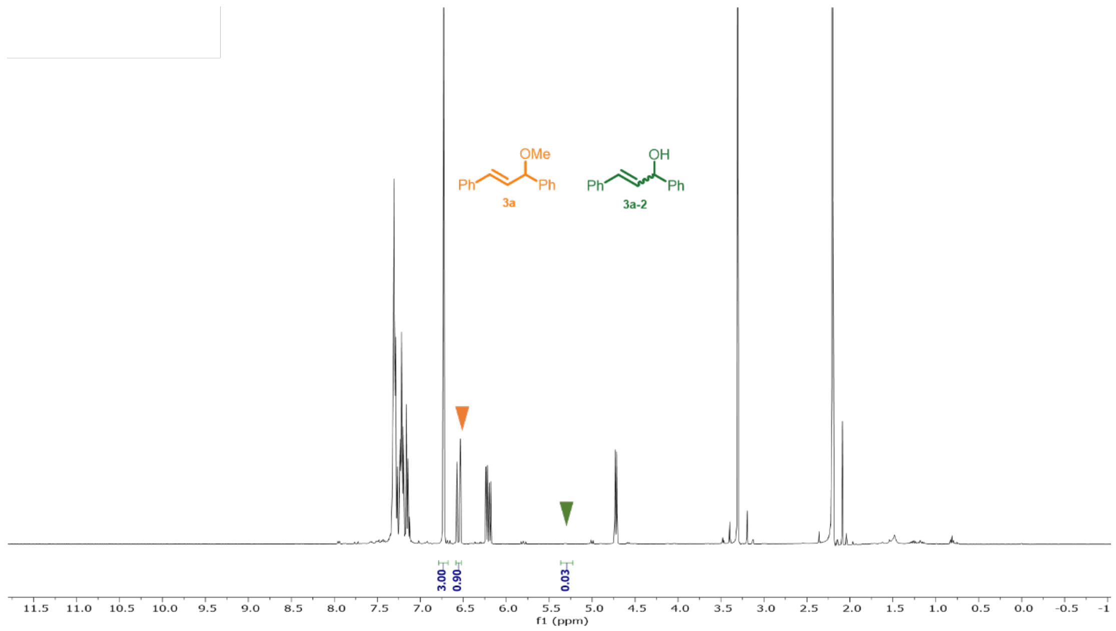

Figure S12 – ^{1}H NMR (400 MHz, $CDCl_3$, 298 K) spectrum of the crude reaction mixture obtained under *Condition B* using **C2** as a catalyst.

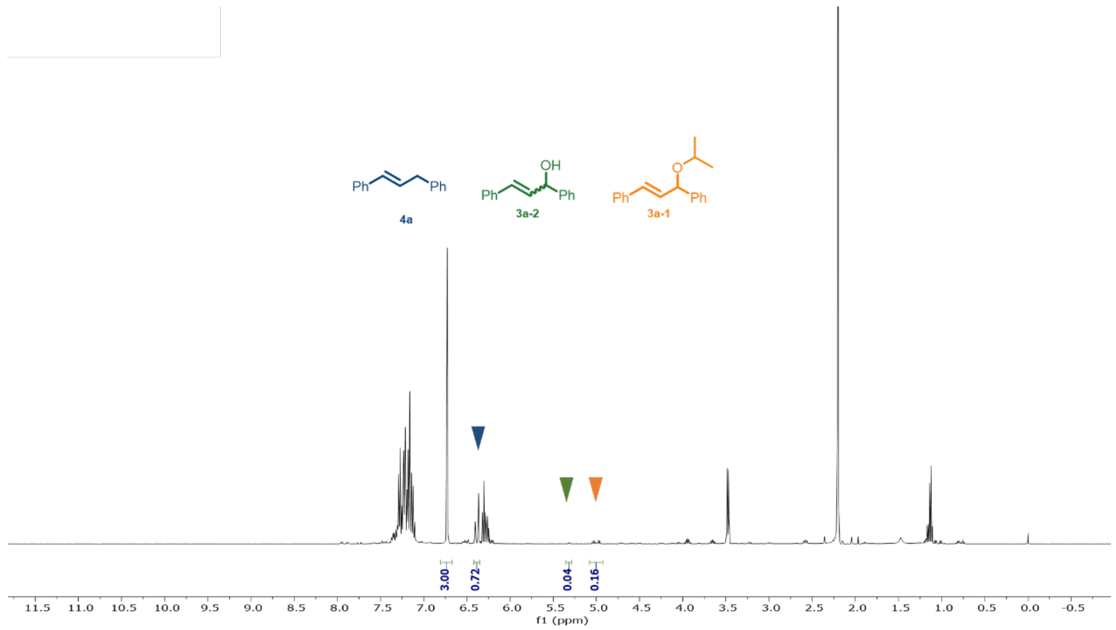

Figure S13 – ^{1}H NMR (400 MHz, $CDCl_3$, 298 K) spectrum of the crude reaction mixture obtained under *Condition C* using **C2** as a catalyst.

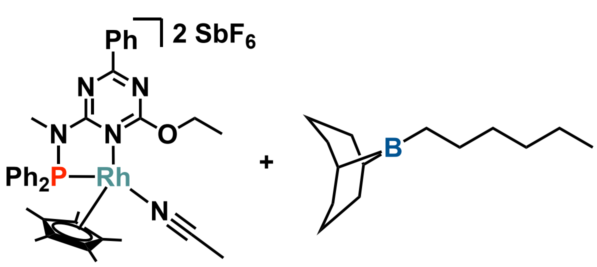

C3

Complex **C3** was evaluated under three modified optimized sets of conditions corresponding to alkyne reduction (*Condition A*), allyl ether synthesis (*Condition B*), and alkene synthesis (*Condition C*). **Modifications:** All reactions were carried out in the presence of alkyl borane (10 mol%).

Results (NMR yields):

Condition A: **1a:** 14%; **2a:** 61%; **4a:** 8%; **5a-1:** 15%

Condition B: **3a:** 89%

Condition C: **3a-1:** 4%; **3a-2:** 2%; **4a:** 84%

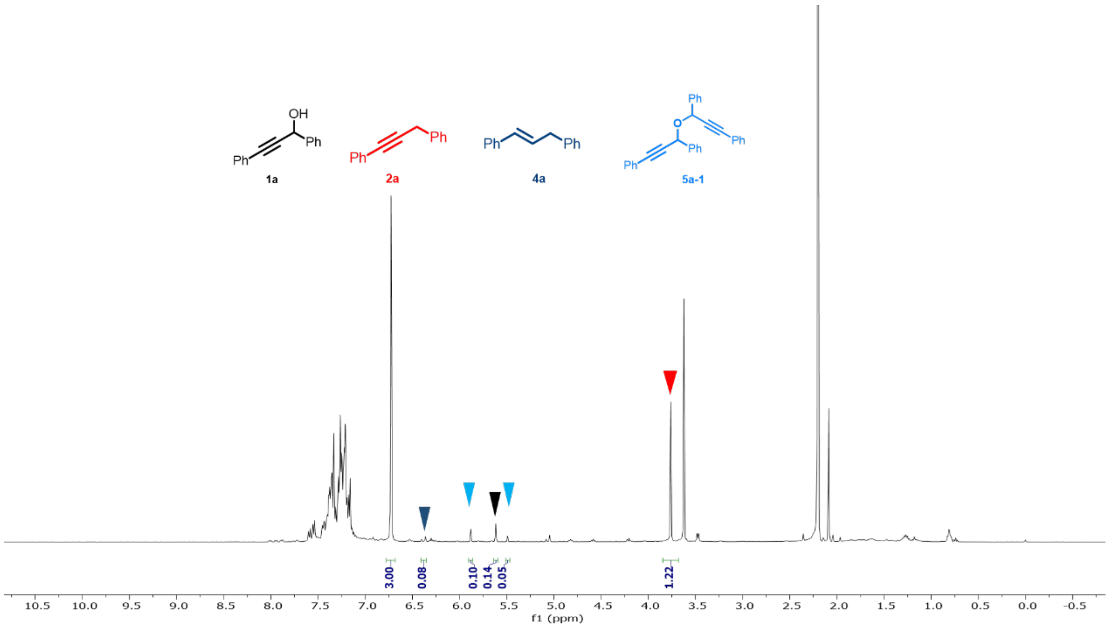

Figure S14 – ^{1}H NMR (400 MHz, $CDCl_3$, 298 K) spectrum of the crude reaction mixture obtained under *Condition A* using **C3** as a catalyst.

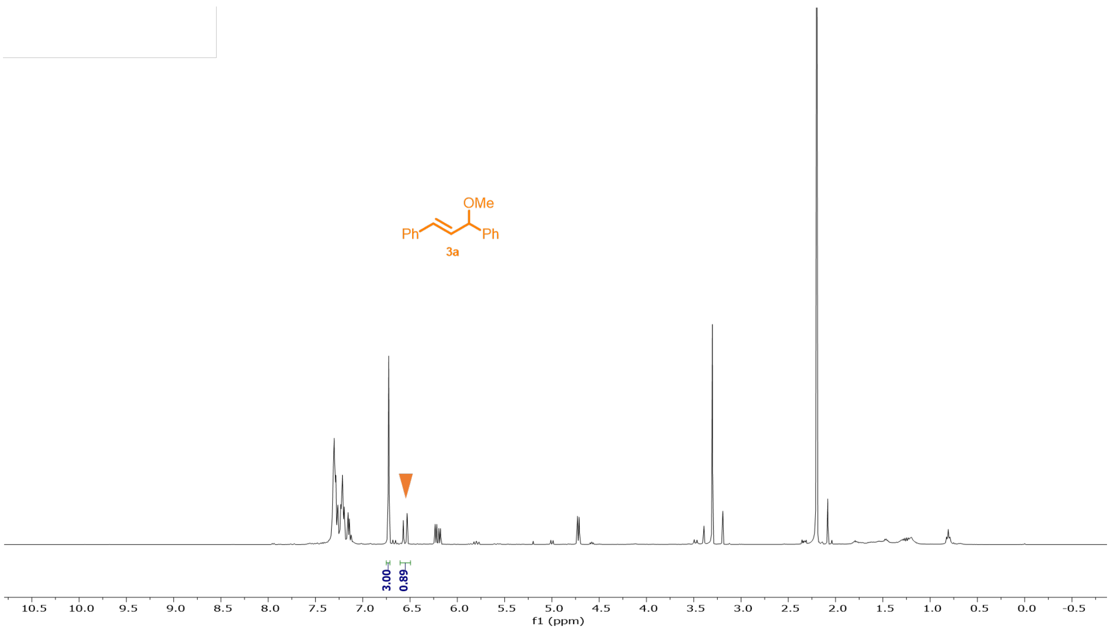

Figure S15 – ¹H NMR (400 MHz, CDCl₃, 298 K) spectrum of the crude reaction mixture obtained under *Condition B* using **C3** as a catalyst.

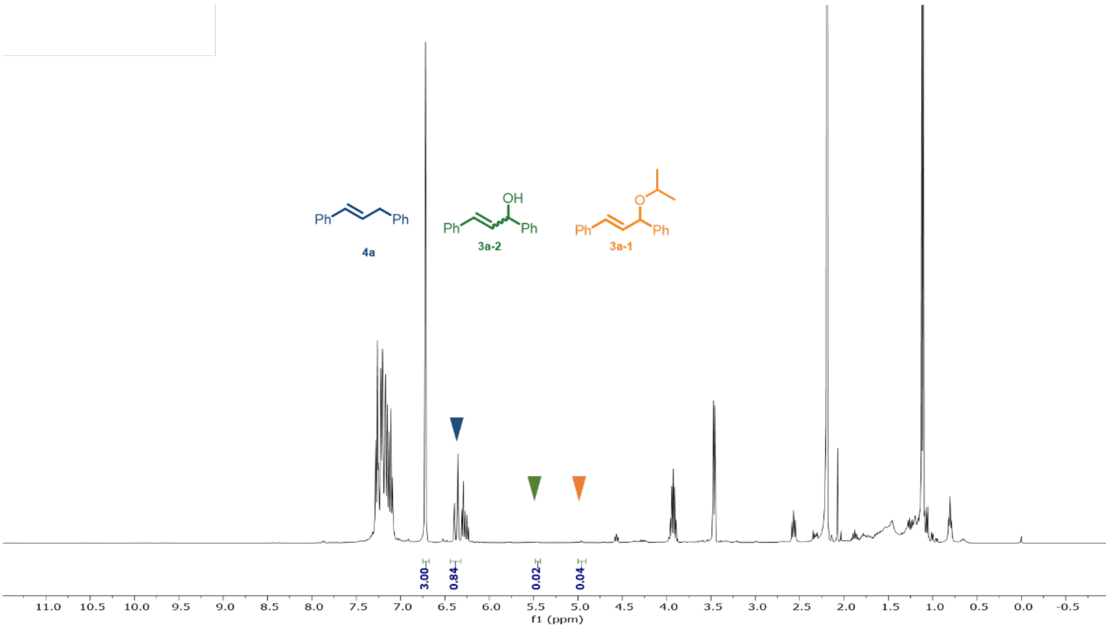

Figure S16 – ¹H NMR (400 MHz, CDCl₃, 298 K) spectrum of the crude reaction mixture obtained under *Condition C* using **C3** as a catalyst.

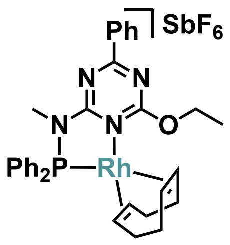

C4

Complex **C4** was evaluated under three optimized sets of conditions corresponding to alkyne reduction (*Condition A*), allyl ether synthesis (*Condition B*), and alkene synthesis (*Condition C*).

Results (NMR yields):

Condition A: **1a**: 13%

Condition B: **3a:** 26%; **3a-2:**36%; **6a-1:** 38%

Condition C: **3a-1:** 10%; **6a-1:** 22%; **6a-2:** 58%

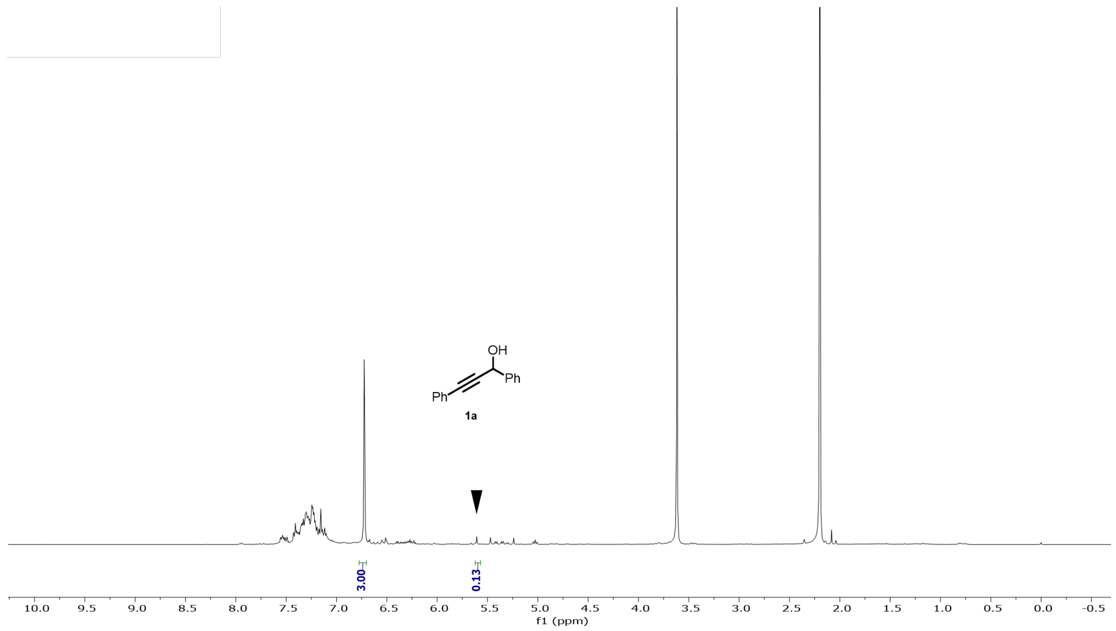

Figure S17 – ^{1}H NMR (400 MHz, $CDCl_3$, 298 K) spectrum of the crude reaction mixture obtained under *Condition A* using **C4** as a catalyst.

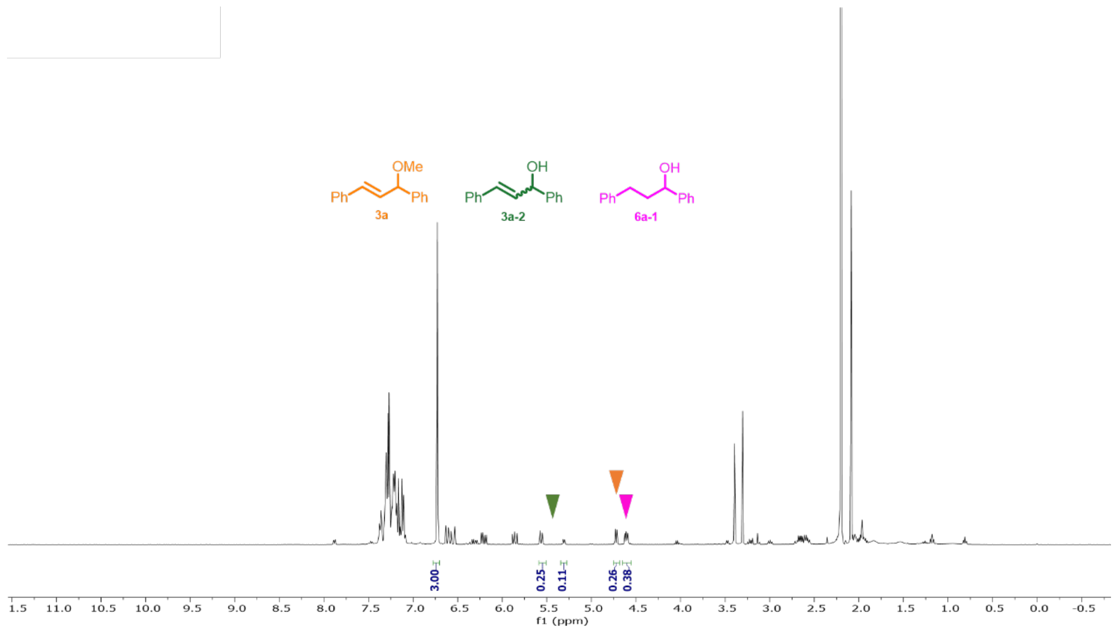

Figure S18 – ^{1}H NMR (400 MHz, $CDCl_3$, 298 K) spectrum of the crude reaction mixture obtained under *Condition B* using **C4** as a catalyst.

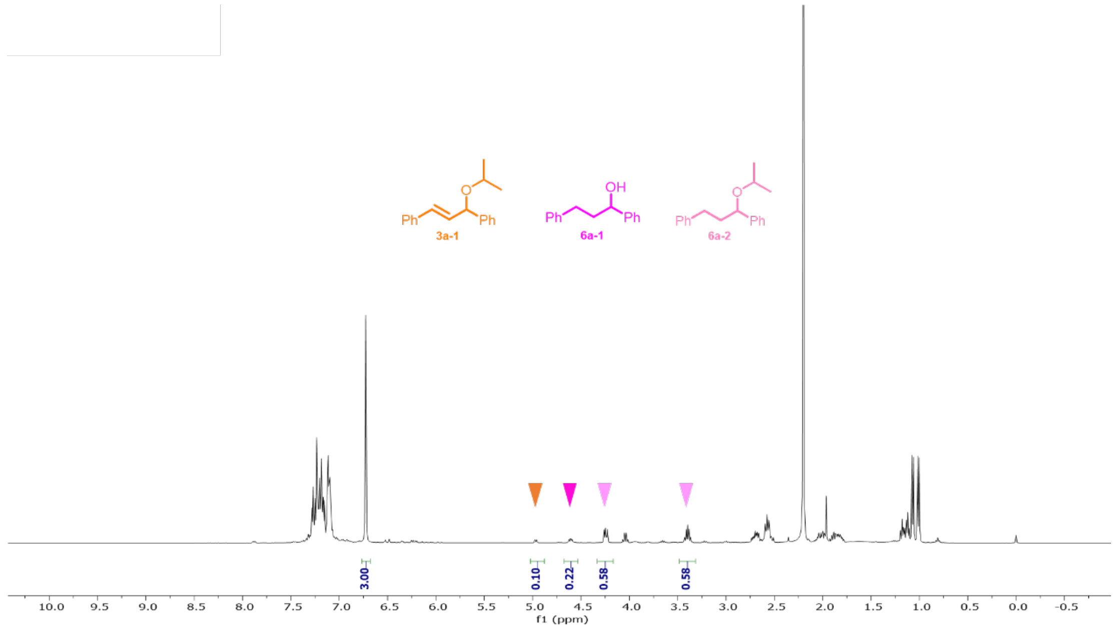

Figure S19 – ^{1}H NMR (400 MHz, $CDCl_3$, 298 K) spectrum of the crude reaction mixture obtained under *Condition C* using **C4** as a catalyst.

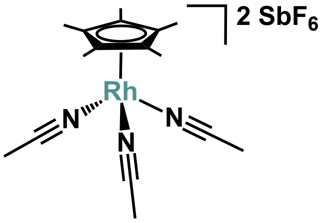

C5

Complex **C5** was evaluated under three optimized sets of conditions corresponding to alkyne reduction (*Condition A*), allyl ether synthesis (*Condition B*), and alkene synthesis (*Condition C*).

Results (NMR yields):

Condition A: **1a:** 8%; **5a-1:** 55%

Condition B: **1a:** 8%; **5a-2:** 66%

Condition C: **1a:** 10% **5a-3:** 47%

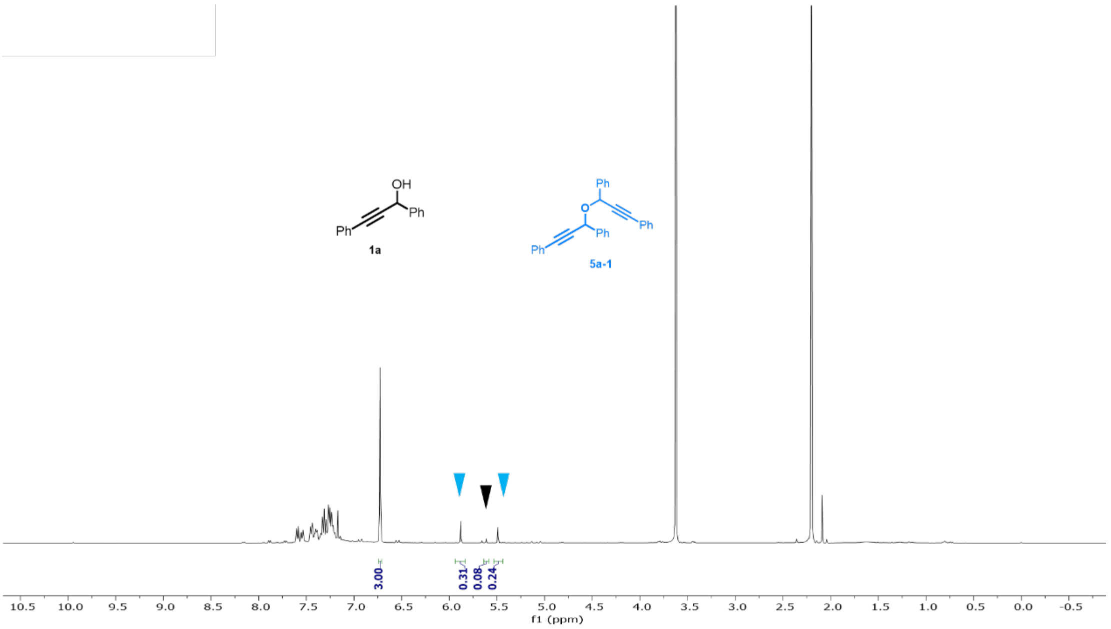

Figure S20 – ^{1}H NMR (400 MHz, $CDCl_3$, 298 K) spectrum of the crude reaction mixture obtained under *Condition A* using **C5** as a catalyst.

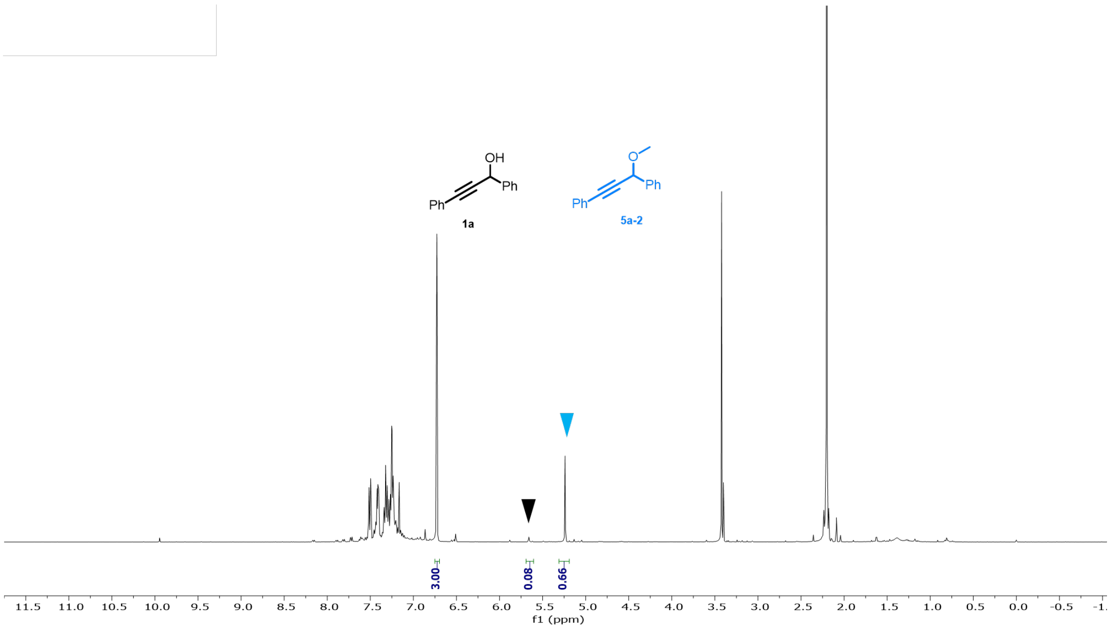

Figure S21 – ^{1}H NMR (400 MHz, $CDCl_3$, 298 K) spectrum of the crude reaction mixture obtained under *Condition B* using **C5** as a catalyst.

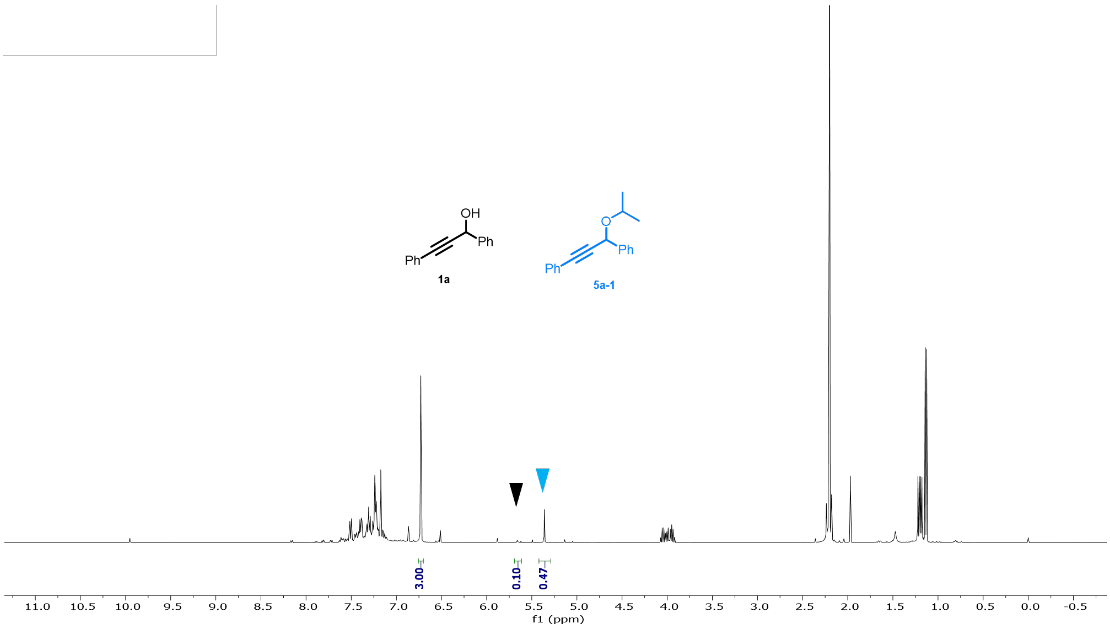

Figure S22 – ^{1}H NMR (400 MHz, $CDCl_3$, 298 K) spectrum of the crude reaction mixture obtained under *Condition C* using **C5** as a catalyst.

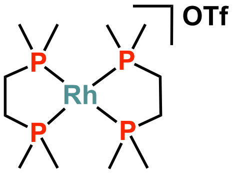

C6

Complex **C6** was evaluated under three optimized sets of conditions corresponding to alkyne reduction (*Condition A*), allyl ether synthesis (*Condition B*), and alkene synthesis (*Condition C*).

Results (NMR yields):

Condition A: **3a-2:** 95%

Condition B: **3a-2:** >99%

Condition C: **6a-1:** 95% **6a-2:** 3%

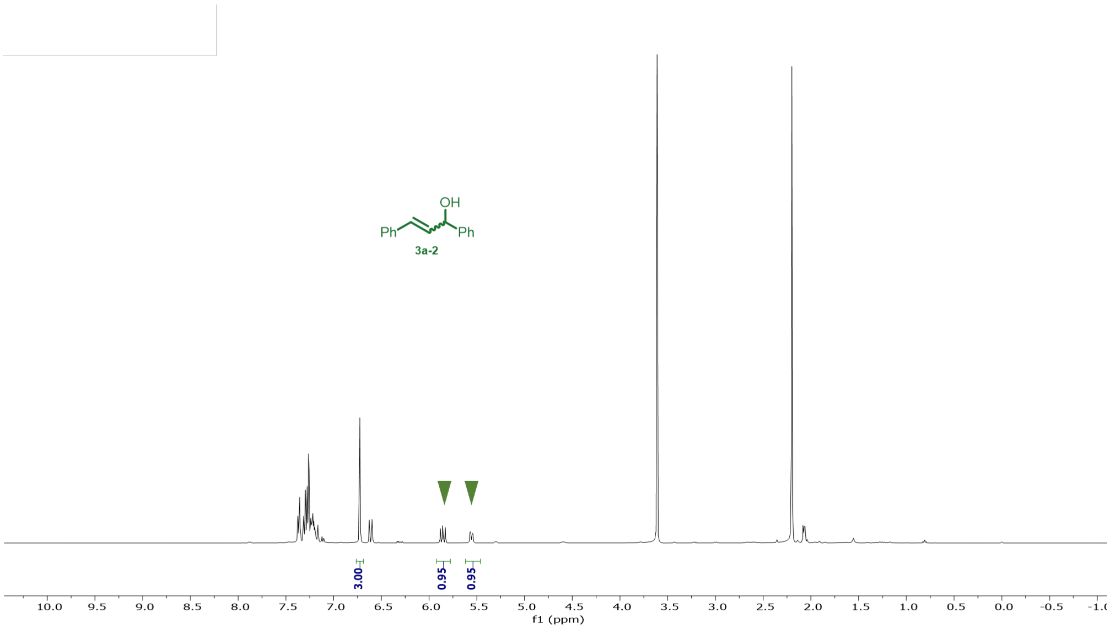

Figure S23 – ^{1}H NMR (400 MHz, $CDCl_3$, 298 K) spectrum of the crude reaction mixture obtained under *Condition A* using **C6** as a catalyst.

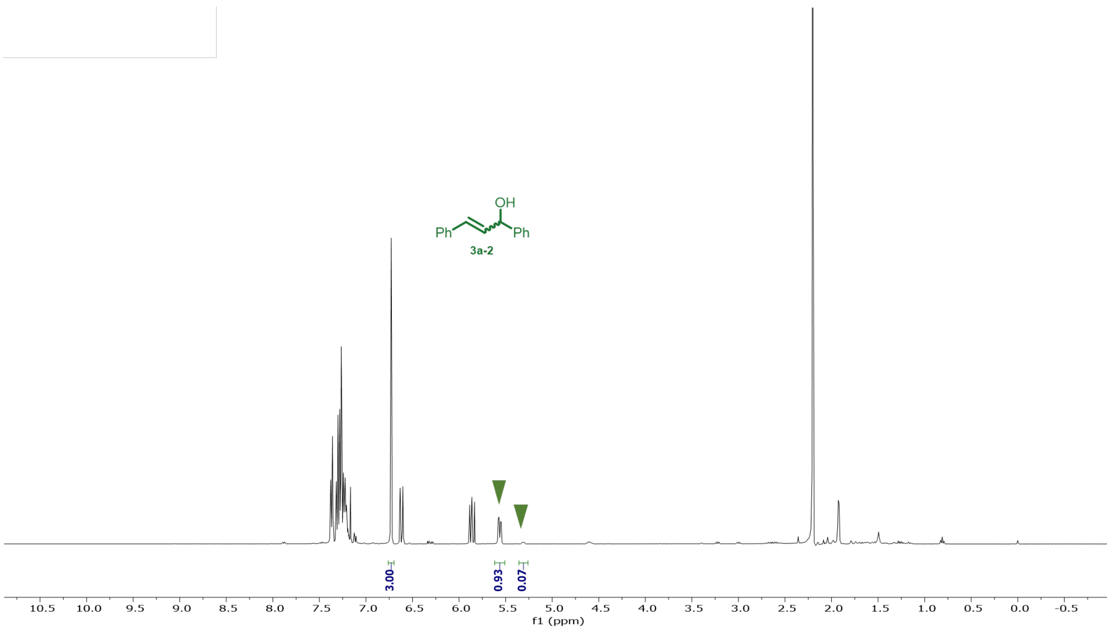

Figure S24 – ^{1}H NMR (400 MHz, $CDCl_3$, 298 K) spectrum of the crude reaction mixture obtained under *Condition B* using **C6** as a catalyst.

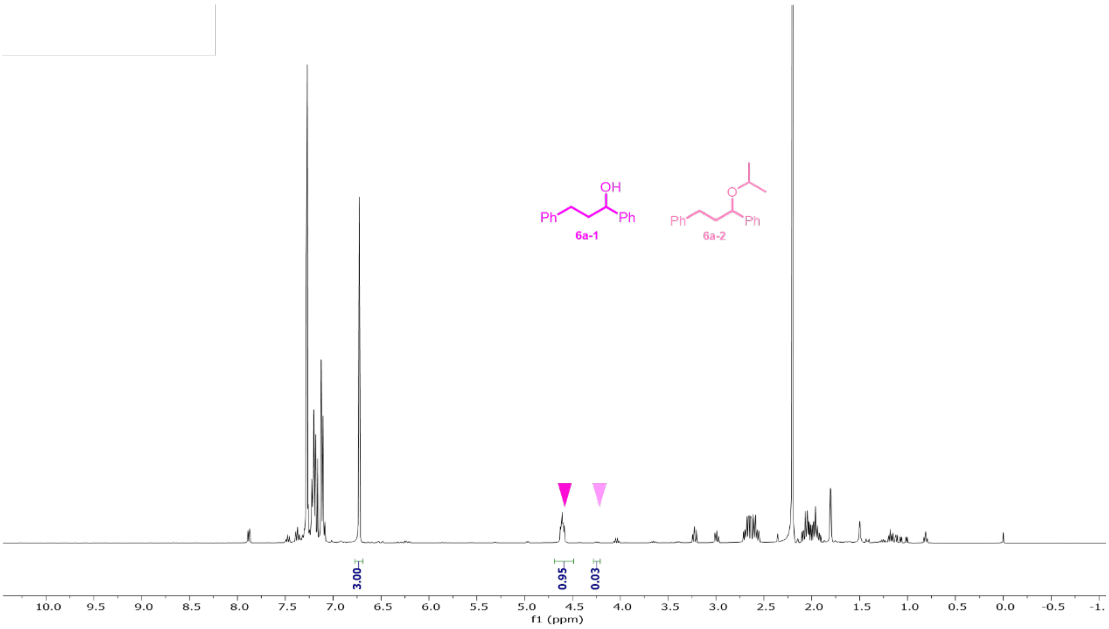

Figure S25 – ^{1}H NMR (400 MHz, $CDCl_3$, 298 K) spectrum of the crude reaction mixture obtained under *Condition C* using **C6** as a catalyst.

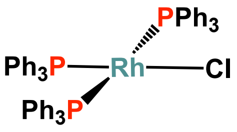

C7

Complex **C7** was evaluated under three optimized sets of conditions corresponding to alkyne reduction (*Condition A*), allyl ether synthesis (*Condition B*), and alkene synthesis (*Condition C*).

Results (NMR yields):

Condition A: **6a-1:** 99%

Condition B: **6a-1:** >99%

Condition C: **6a-1:** 97%

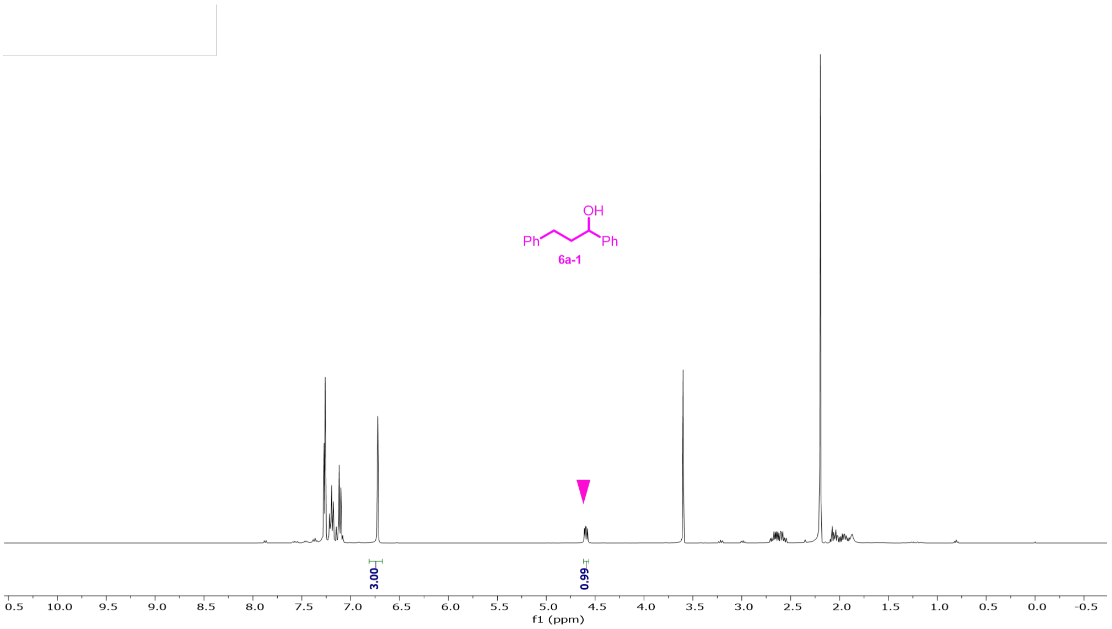

Figure S26 – ^{1}H NMR (400 MHz, $CDCl_3$, 298 K) spectrum of the crude reaction mixture obtained under *Condition A* using **C7** as a catalyst.

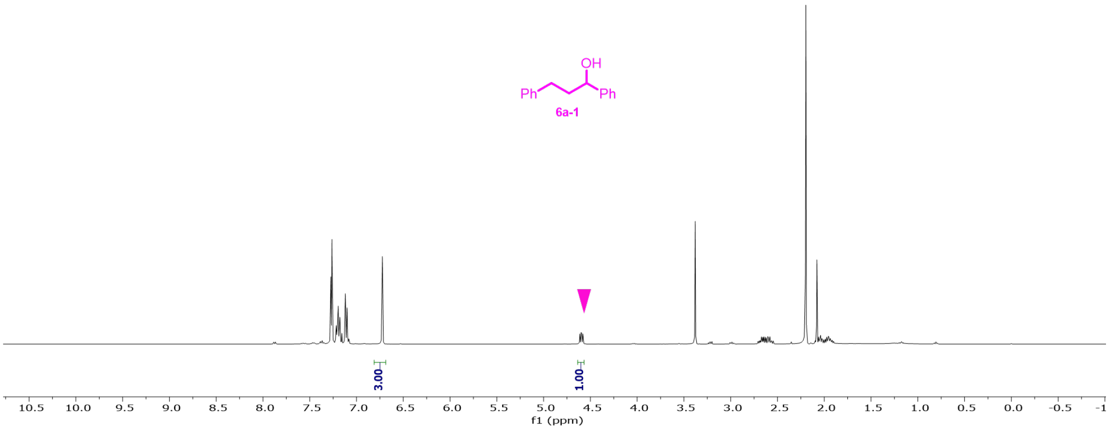

Figure S27 – ¹H NMR (400 MHz, $CDCl_3$, 298 K) spectrum of the crude reaction mixture obtained under *Condition B* using **C7** as a catalyst.

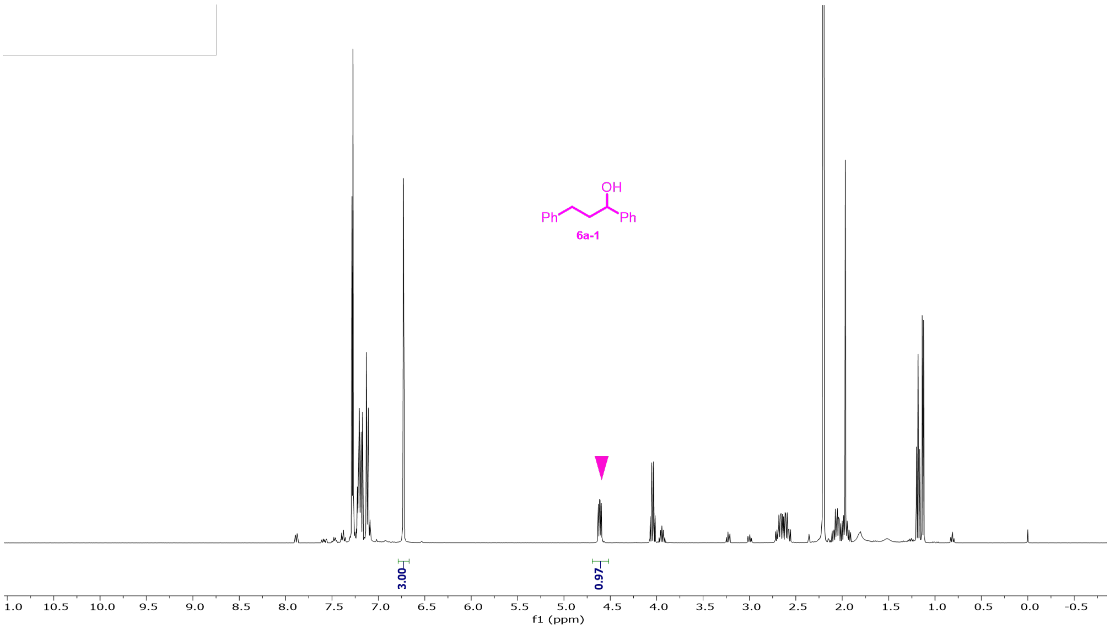

Figure S28 – ¹H NMR (400 MHz, $CDCl_3$, 298 K) spectrum of the crude reaction mixture obtained under *Condition C* using **C7** as a catalyst.

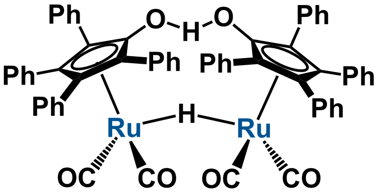

C8

Complex **C8** was evaluated under three optimized sets of conditions corresponding to alkyne reduction (*Condition A*), allyl ether synthesis (*Condition B*), and alkene synthesis (*Condition C*).

Results (NMR yields):

Condition A: **1a:** 97%

Condition B: **1a:** >99%

Condition C: **1a:** 85% **3a-2:** 14%

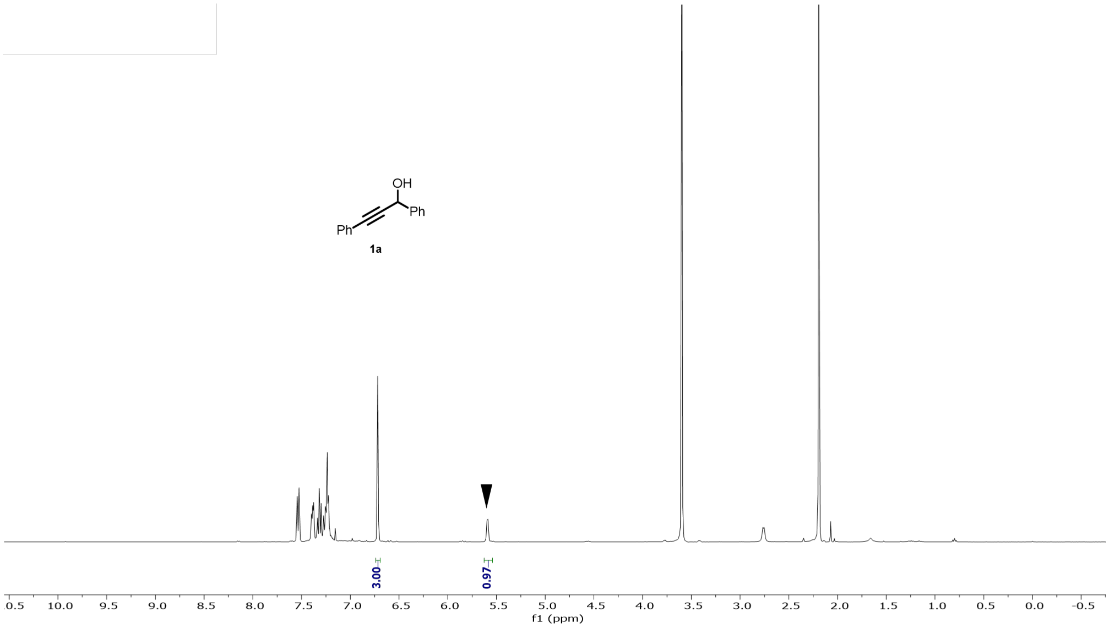

Figure S29 – ^{1}H NMR (400 MHz, CDCl$_3$, 298 K) spectrum of the crude reaction mixture obtained under *Condition A* using **C8** as a catalyst.

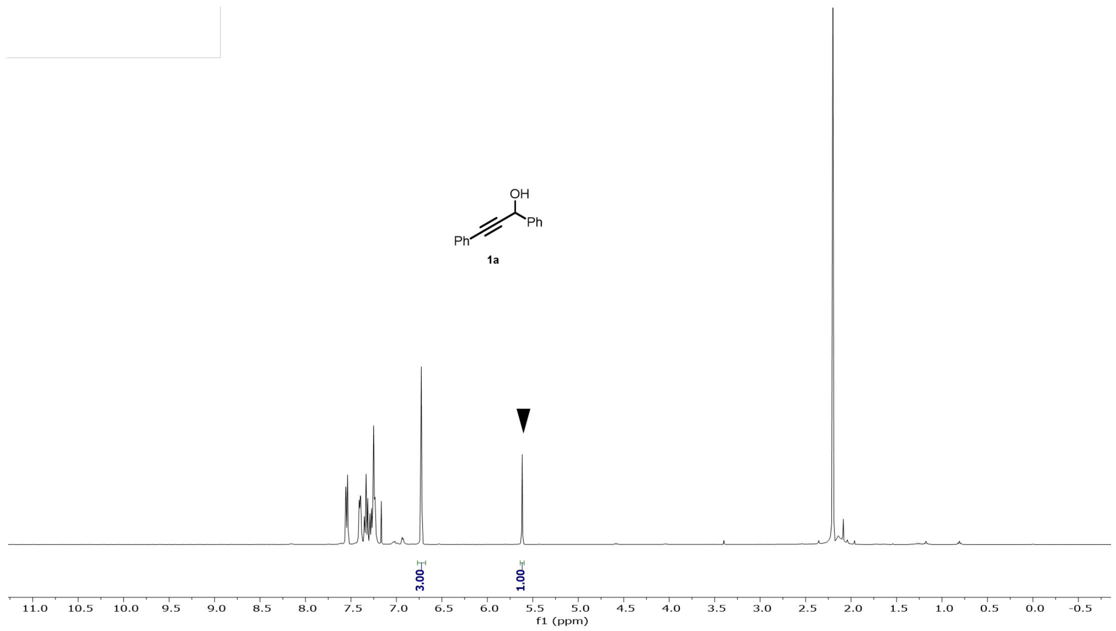

Figure S30 – ^{1}H NMR (400 MHz, $CDCl_3$, 298 K) spectrum of the crude reaction mixture obtained under *Condition B* using **C8** as a catalyst.

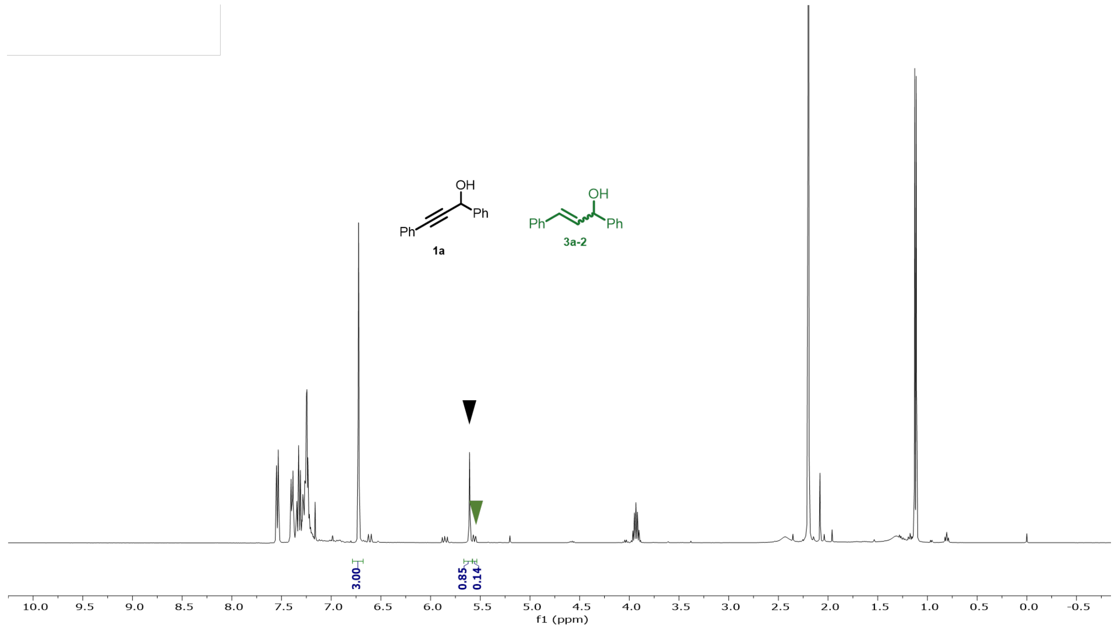

Figure S31 – ^{1}H NMR (400 MHz, $CDCl_3$, 298 K) spectrum of the crude reaction mixture obtained under *Condition C* using **C8** as a catalyst.

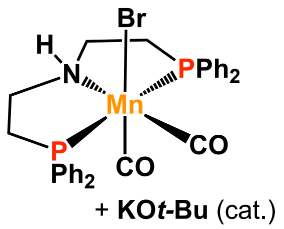

C9

Complex **C9** was evaluated under three modified optimized sets of conditions corresponding to alkyne reduction (*Condition A*), allyl ether synthesis (*Condition B*), and alkene synthesis (*Condition C*).
Modifications: All reactions were carried out in the presence of [Mn] (5 mol%) and *t*-BuOK (5 mol%).

Results (NMR yields):

Condition A: **1a:** 37%; **5a-1:** 10%

Condition B: **1a:** >99%

Condition C: **6a-1:** 95%

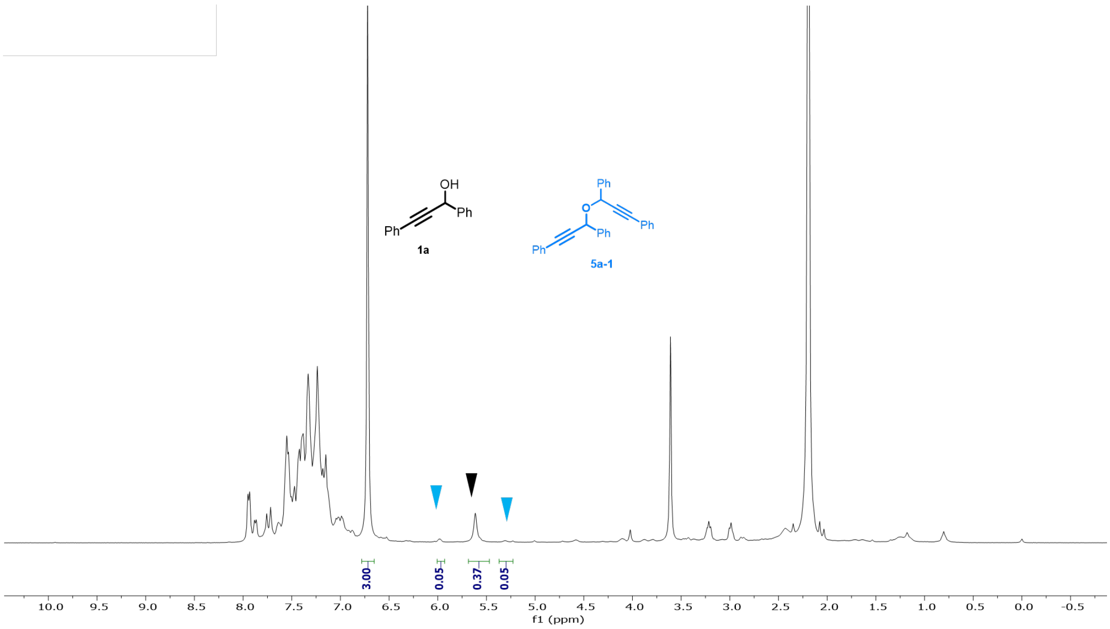

Figure S32 – ^{1}H NMR (400 MHz, $CDCl_3$, 298 K) spectrum of the crude reaction mixture obtained under *Condition A* using **C9** as a catalyst.

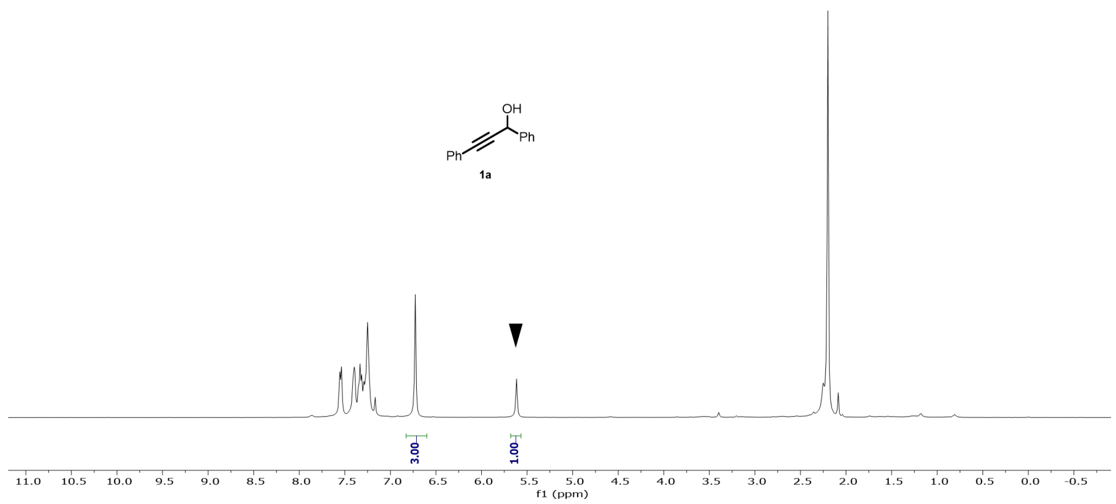

Figure S33 – ^{1}H NMR (400 MHz, $CDCl_3$, 298 K) spectrum of the crude reaction mixture obtained under *Condition B* using **C9** as a catalyst.

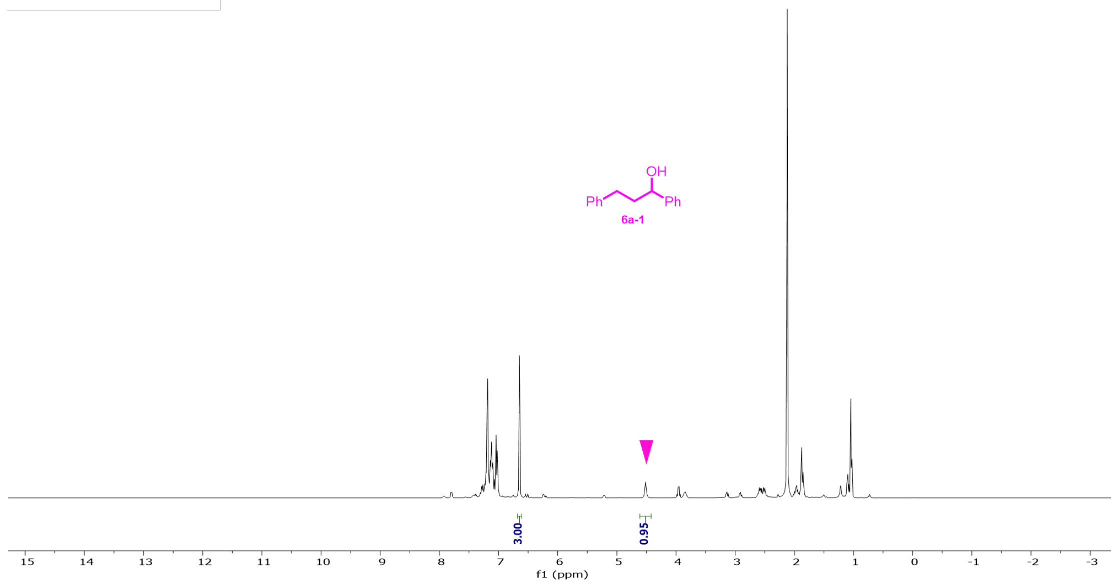

Figure S34 – ^{1}H NMR (400 MHz, $CDCl_3$, 298 K) spectrum of the crude reaction mixture obtained under *Condition C* using **C9** as a catalyst.

11. Mechanistic Studies

11.1 Control Experiments in the Absence of H_2

Table S4 – Reactions in the absence of H_2 under argon.

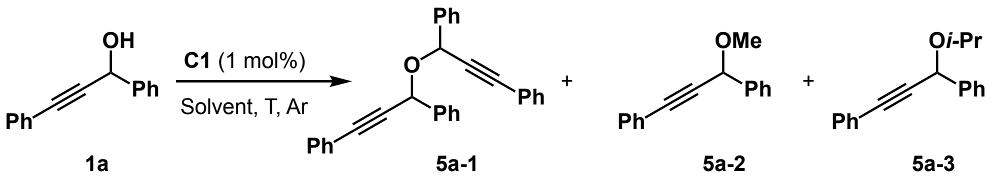

Entry	Conditions	Conv.(%)[a]	Yield of 5a-1(%)[a]	Yield of 5a-2(%)[a]	Yield of 5a-3(%)[a]
**1**	Dioxane, 70 °C	85	83	-	-
**2**	MeOH, 40 °C	82	53	24	-
**3**	*i*-PrOH, 80 °C	>99	-	-	>99

[a]Reaction conditions: **1a** (0.25 mmol, 1 equiv.), **C1** (1 mol%), solvent (1 mL) under argon. Conversions (%) and yields (%) were determined using mesitylene (0.25 mmol) as an internal standard.

11.2 Control Experiments with Boron-Based Additive Alone

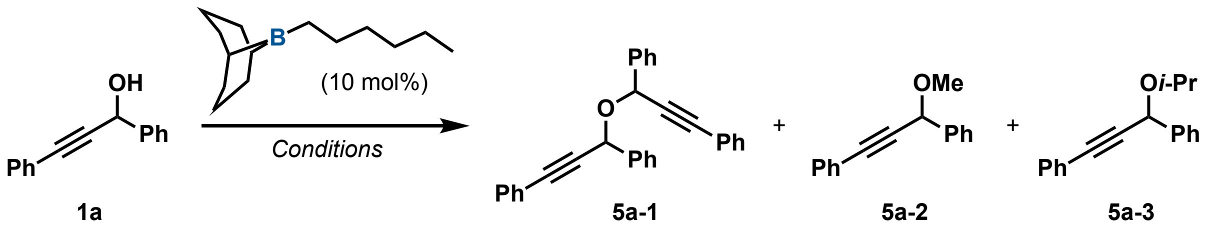

In a glovebox under an argon atmosphere, propargylic alcohol **1a** (0.50 mmol, 104.1 mg), boron-based additive (the same as in catalyst system **C3**; 0.050 mmol, 10.3 mg, 10 mol%), and solvent (2.0 mL) were added to a sealed reaction vial equipped with a magnetic stir bar. The vial was removed from the glovebox and heated at the specified temperature for the indicated time: Entry 1 – 70 °C, 3 h (dioxane); Entry 2 – 40 °C, 5 h (MeOH); Entry 3 – 80 °C, 5 h (*i*-PrOH). After cooling to room temperature, the solvent was removed under reduced pressure. Conversion of **1a** to ether products was determined by ^{1}H NMR spectroscopy using mesitylene (0.50 mmol) as an internal standard.

Table S5 – Control experiments with the boron-based additive alone (no [Rh] catalyst) under various solvents and conditions. Conversion of propargylic alcohol **1a** to ether products determined by ^{1}H NMR with mesitylene as internal standard.

Entry	Conditions	Conv. 1a (%)
1	Dioxane, 70 °C, 3h, Ar	<1
2	MeOH, 40 °C, 5 h, Ar	<1
3	*i*-PrOH, 80 °C, 5 h, Ar	<1

11.3 Control Experiment: Formation of 2a from 5a-1 using C1

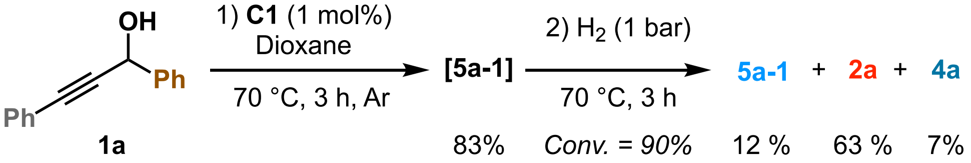

One-pot two-step. In an oven-dried Fisher–Porter tube under an argon atmosphere, propargylic alcohol **1a** (52.1 mg, 0.25 mmol), complex **C1** (3.4 mg, 1 mol%), and dioxane (1 mL) were combined. The mixture was stirred at 70 °C for 3 h, flash-frozen with liquid nitrogen, evacuated, and pressurized with H_2 (1 bar). The sealed tube was stirred at 70 °C for an additional 3 h. After cooling to room temperature, the pressure was carefully released, and the solvent was removed under reduced pressure. Conversion and yield were determined by ^{1}H NMR spectroscopy of the crude residue using mesitylene (0.25 mmol) as an internal standard.

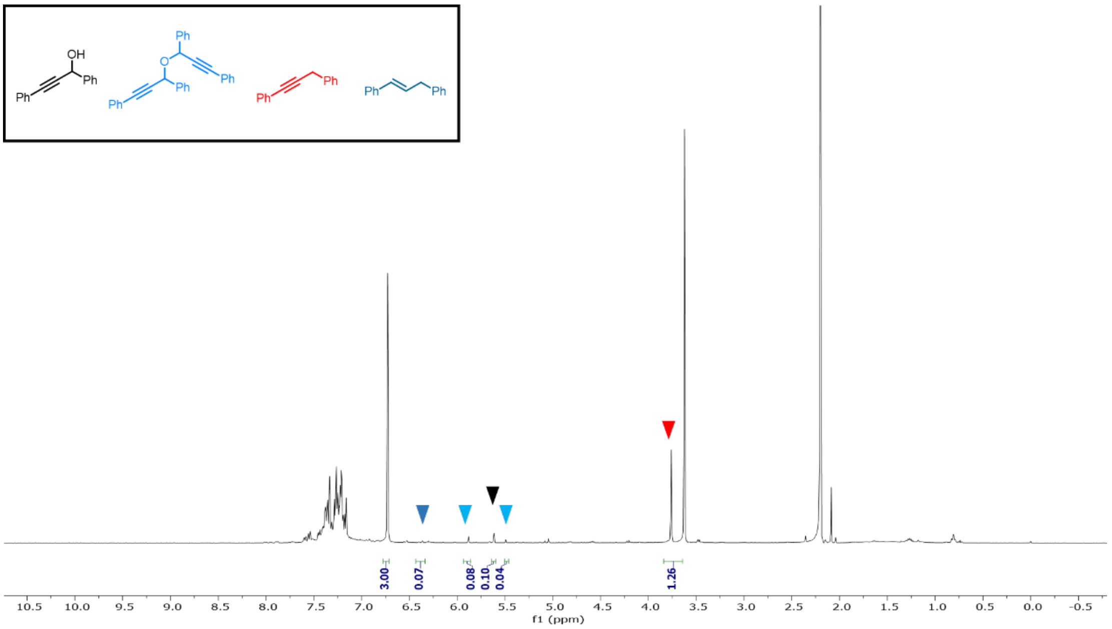

Figure S35 – ^{1}H NMR (400 MHz, $CDCl_3$, 298 K) spectrum of the crude reaction mixture obtained from the one-pot two-step conversion of **1a** to **2a** using complex **C1**.

11.4 Control Experiment: Formation of 2a from 5a-1 using C2

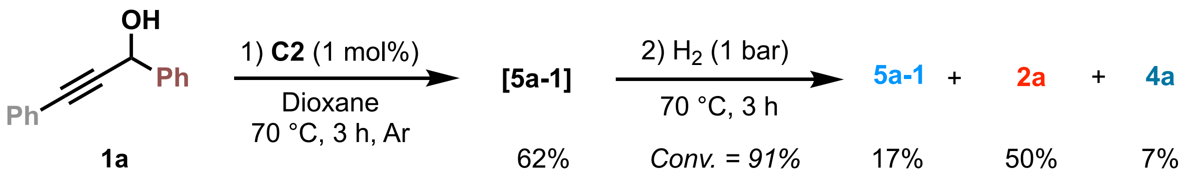

(1) *First step*. In an oven-dried Schlenk tube under an argon atmosphere, propargylic alcohol **1a** (52.1 mg, 0.25 mmol), complex **C2** (3.1 mg, 1 mol%), and dioxane (1 mL) were combined. The mixture was stirred at 70 °C for 3 h, cooled to room temperature, and the solvent was removed under reduced pressure. The yield of **5a-1** was determined by ^{1}H NMR spectroscopy of the crude residue using mesitylene (0.25 mmol) as an internal standard.

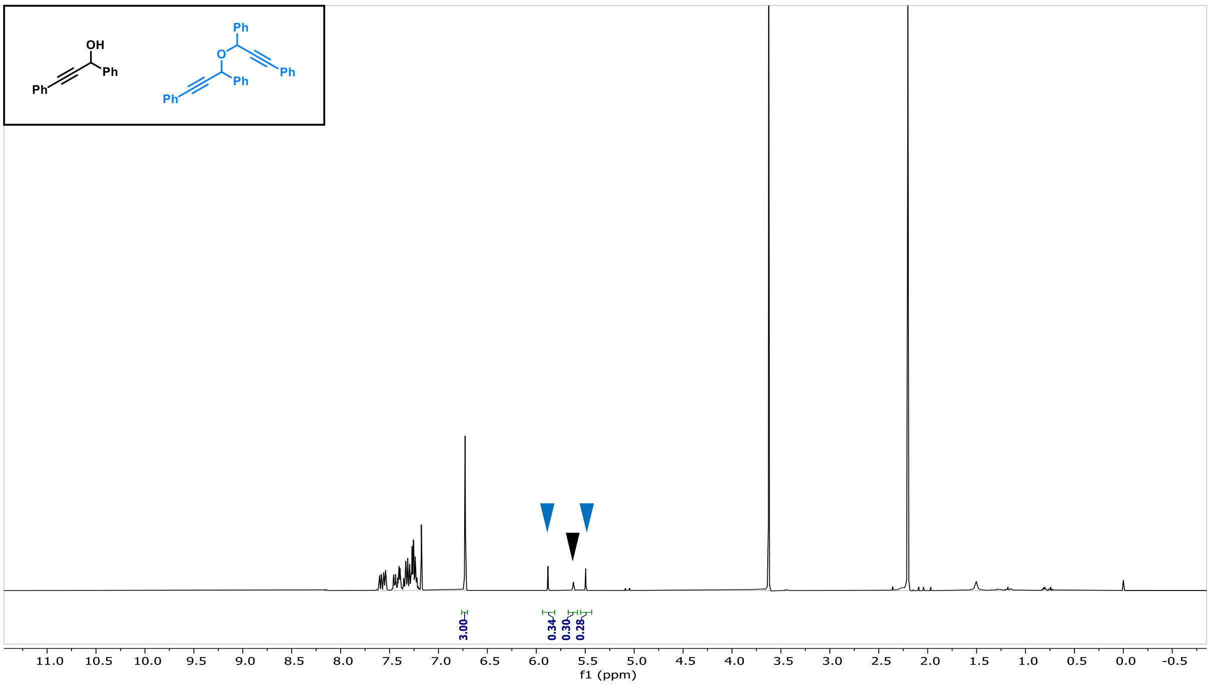

Figure S36 – ^{1}H NMR (400 MHz, CDCl$_3$, 298 K) spectrum of the crude reaction mixture obtained from the conversion of **1a** to **5a-1** using complex **C2**.

(2) *One-pot two-step*. In an oven-dried Fisher–Porter tube under an argon atmosphere, propargylic alcohol **1a** (52.1 mg, 0.25 mmol), complex **C2** (3.1 mg, 1 mol%), and dioxane (1 mL) were combined. The mixture was stirred at 70 °C for 3 h, flash-frozen with liquid nitrogen, evacuated, and pressurized with H_2 (1 bar). The sealed tube was stirred at 70 °C for an additional 3 h. After cooling to room temperature, the pressure was carefully released, and the solvent was removed under reduced pressure. Conversion and yields were determined by ^{1}H NMR spectroscopy of the crude residue using mesitylene (0.25 mmol) as an internal standard.

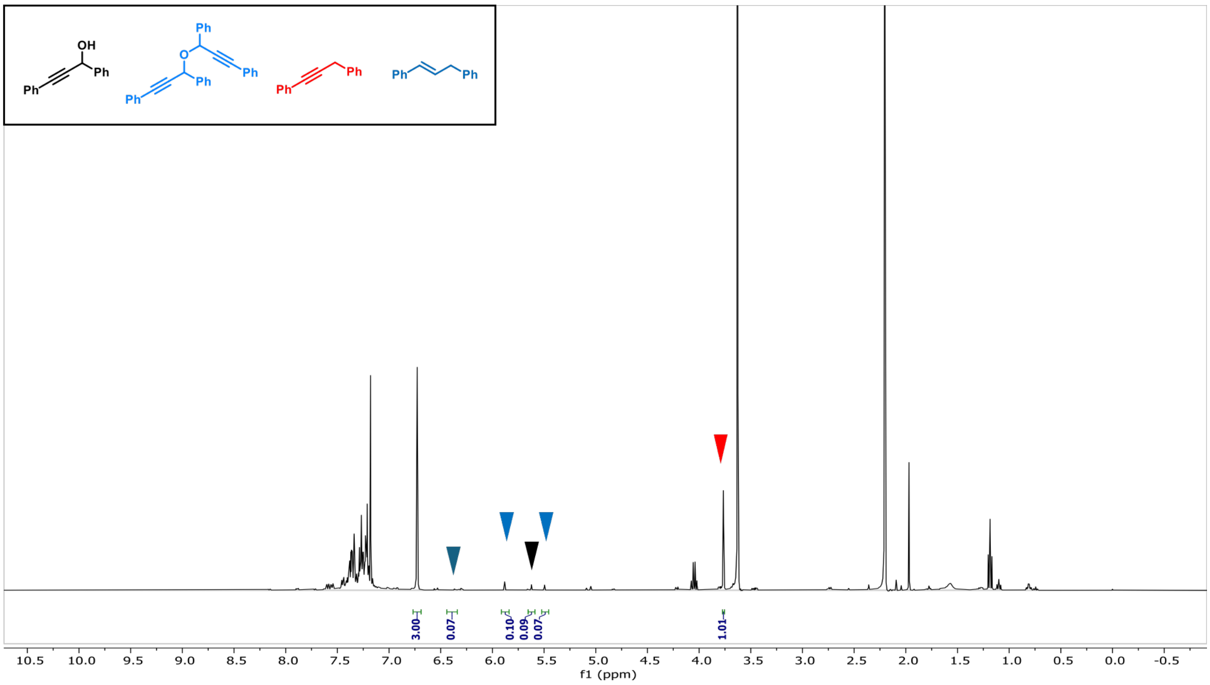

Figure S37 – ^{1}H NMR (400 MHz, $CDCl_3$, 298 K) spectrum of the crude reaction mixture obtained from the one-pot two-step conversion of **1a** to **2a** using complex **C2**.

11.5 Conversion of Isolated Propargylic Ether 5a-3 to Allylic Ether 3a under *Condition B*

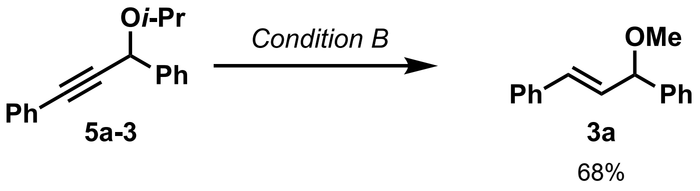

Experimental Procedure: In an oven-dried Fisher–Porter tube under an argon atmosphere, propargylic ether **5a-3** (0.50 mmol), catalyst **C1** (0.0050 mmol, 6.7 mg, 1 mol%), and methanol (2.0 mL) were added. The mixture was flash-frozen in liquid nitrogen, evacuated, and pressurized with H_2 (2 bar). The sealed vessel was stirred at 40 °C for 5 h. After cooling to room temperature, the H_2 pressure was carefully released, and the solvent was removed under reduced pressure. The yield of **3a** was determined by ^{1}H NMR spectroscopy using mesitylene (0.50 mmol) as an internal standard.

11.6 Time-Course Study for 1a → 5a-1 → 2a under *Condition A*

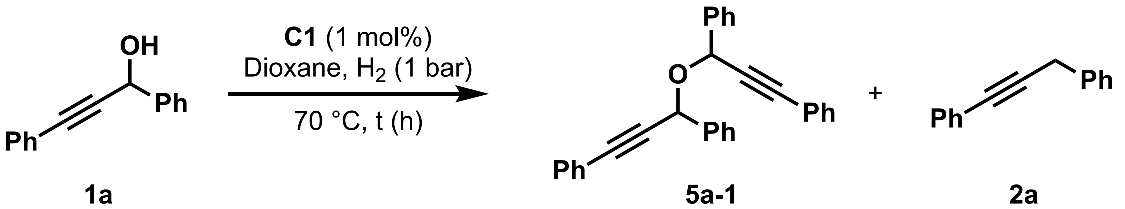

Table S6 – Time-course analysis of the hydrodeoxygenation pathway under *Condition A*. Conversion of **1a** and evolution of intermediate **5a-1** into alkyne **2a** were monitored by ^{1}H NMR using mesitylene as internal standard.

Entry	Time (h)	Conv. 1a (%)	Yield 5a-1 (%)	Yield 2a (%)
1	0.5	72	53	10
2	1	76	43	24
3	2	85	32	38
4	3	>99	0	76

Experimental Procedure: In an oven-dried Fisher–Porter tube under an argon atmosphere, propargylic alcohol **1a** (0.50 mmol), catalyst **C1** (0.0050 mmol, 6.7 mg, 1 mol%), and dioxane (2.0 mL) were added. The mixture was flash-frozen in liquid nitrogen, evacuated, and pressurized with H_2 (1 bar). Each reaction was stirred at 70 °C for the designated time (0.5, 1, 2, or 3 h) in separate, otherwise identical runs. After cooling to room temperature, the H_2 pressure was carefully released, and the solvent was removed under reduced pressure. Conversions of **1a** and yields of **5a-1** and **2a** were determined by ^{1}H NMR spectroscopy using mesitylene (0.50 mmol) as an internal standard.

11.7 Deuterium-Labeling Experiment under *Condition A*

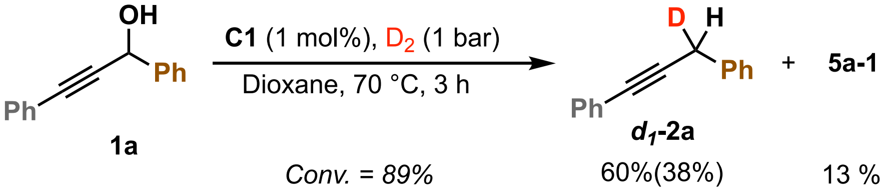

In an oven-dried Fisher–Porter tube under an argon atmosphere, propargylic alcohol **1a** (104.2 mg, 0.50 mmol), complex **C1** (6.7 mg, 1 mol%), and dioxane (2 mL) were added. The mixture was flash-frozen using liquid nitrogen, evacuated, and then pressurized with D_2 (1 bar). The sealed tube was stirred at 70 °C for 3 h. After completion, the reaction was cooled to room temperature, and the pressure was carefully released. The solvent was removed under reduced pressure. Conversion and yield were determined from the crude residue by ^{1}H NMR spectroscopy using mesitylene (0.50 mmol) as an internal standard. The product ***d₁*-2a** was purified by flash column chromatography on silica gel using EtOAc/pentane as the eluent.

(Prop-1-yne-1,3-diyl-3-*d*)dibenzene (*d_1*-2a)

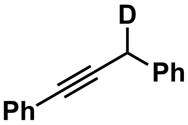

Formula: $C_{15}H_{11}D$

Molecular weight: 193.27

Isolated yield: 38% (36 mg), colorless oil

Rf: 0.4 (Pentane/EtOAc = 50:1)

^{1}H NMR (400 MHz, $CDCl_3$, 298 K) δ 7.58 – 7.13 (m, 10H), 3.82 (d, J = 6.1 Hz, 1H)

^{13}C NMR (101 MHz, $CDCl_3$, 298 K) δ 136.8, 131.8, 128.7, 128.4, 128.1, 128.0, 126.8, 123.8, 87.7, 82.8, 25.9, 25.8, 25.6, 25.4

HRMS (ESI$^+$): Calcd. for $C_{15}H_{12}D^+$ [M+H$^+$]: 194.10591; Found 194.10761

11.8 Deuterium-Labeling Experiment Starting from 3a

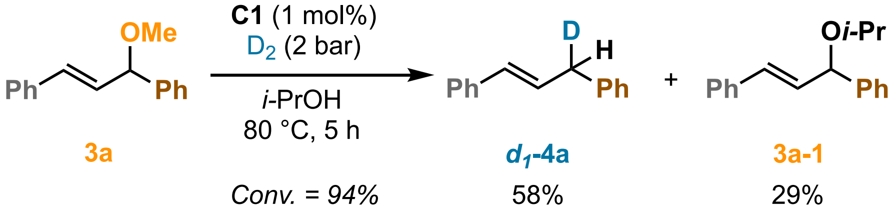

In an oven-dried Fisher–Porter tube under an argon atmosphere, propargylic alcohol **3a** (56.1 mg, 0.25 mmol), complex **C1** (3.4 mg, 1 mol%), and *i*-PrOH (1 mL) were added. The mixture was flash-frozen using liquid nitrogen, evacuated, and then pressurized with D_2 (2 bar). The sealed tube was stirred at 80 °C for 5 h. After completion, the reaction was cooled to room temperature, and the pressure was carefully released. The solvent was removed under reduced pressure. Conversion and yield were determined from the crude residue by ^{1}H NMR spectroscopy using mesitylene (0.50 mmol) as an internal standard. The crude ^{1}H NMR spectrum is shown below.

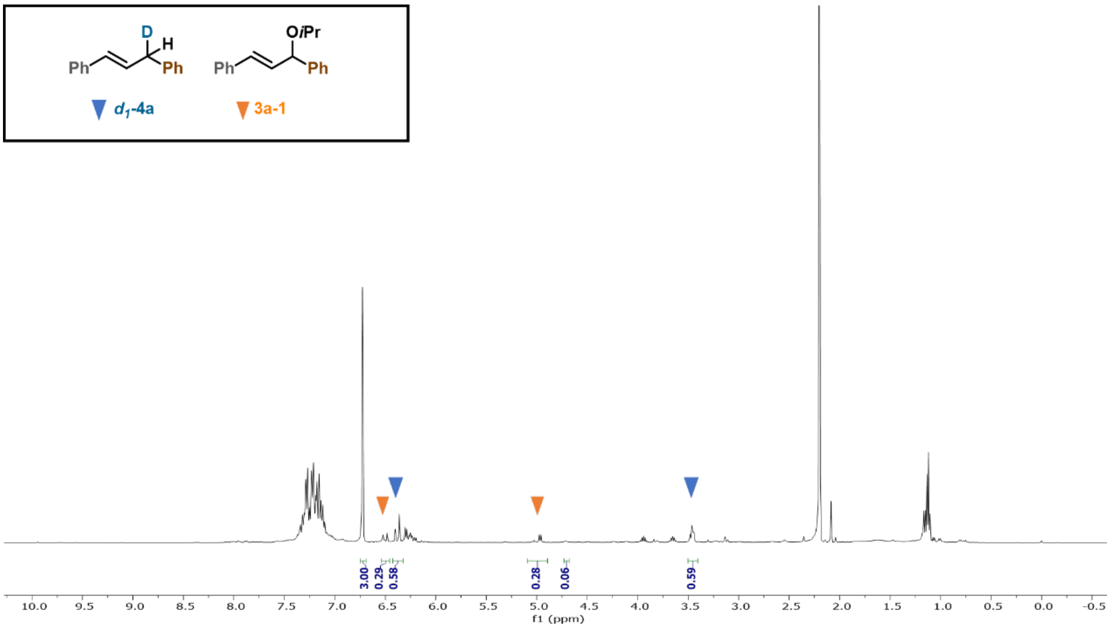

Figure S38 – ^{1}H NMR (400 MHz, $CDCl_3$, 298 K) spectrum of the crude reaction mixture obtained from the deuterium-labeling experiment of **3a** with D_2 under *Condition C.*

11.9 Formation of 2a with [Rh–H]

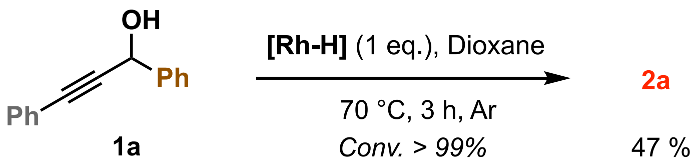

In an oven-dried Schlenk tube under an argon atmosphere, propargylic alcohol **1a** (6.3 mg, 0.030 mmol), **[Rh–H]** complex (1 equiv., prepared according to our previous work[26]), and dioxane (1 mL) were added. The mixture was stirred at 70 °C for 3 h. After completion, the reaction was cooled to room temperature, and the solvent was removed under reduced pressure. Conversion and yield were determined by ^{1}H NMR spectroscopy using 1,3,5-trimethoxybenzene (0.030 mmol) as an internal standard. The crude ^{1}H NMR spectrum is shown below.

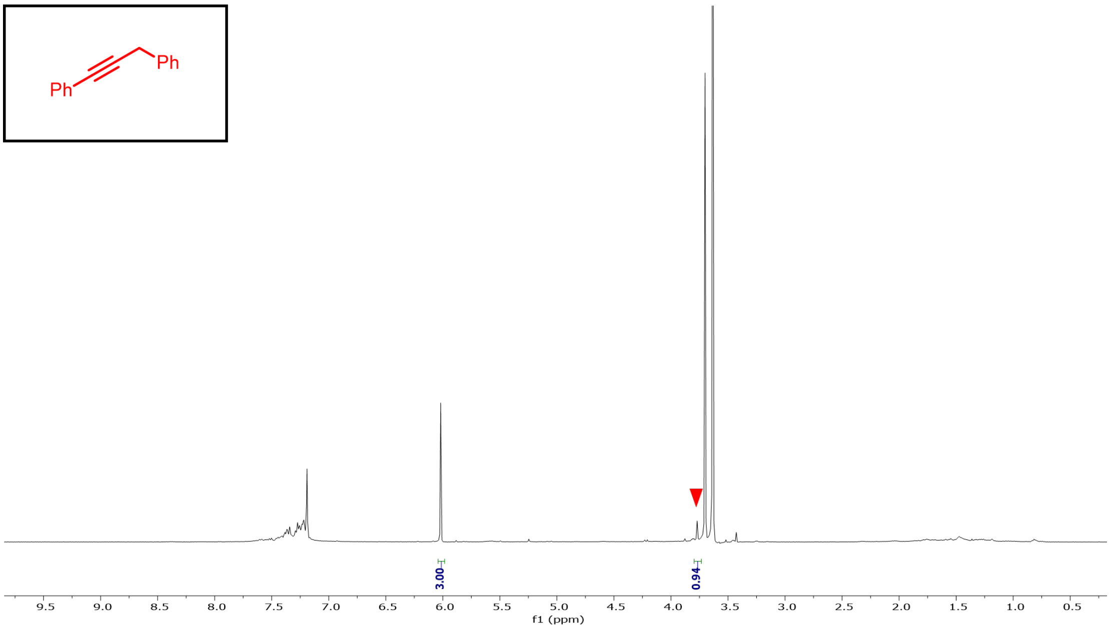

Figure S39 – ^{1}H NMR (400 MHz, $CDCl_3$, 298 K) spectrum of the crude reaction mixture obtained from the conversion of **1a** to **2a** using **[Rh–H]**.

11.10 Formation of 4a with [Rh–H]

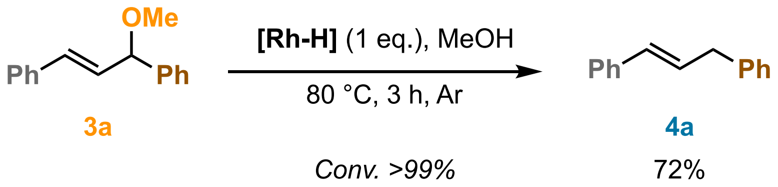

In an oven-dried Schlenk tube under an argon atmosphere, propargylic alcohol **3a** (6.7 mg, 0.030 mmol), **[Rh–H]** complex (1 equiv., prepared according to our previous research[26]), and MeOH (1 mL)

were added. The mixture was stirred at 80 °C for 3 h. After completion, the reaction was cooled to room temperature, and the solvent was removed under reduced pressure. Conversion and yield were determined by ^{1}H NMR spectroscopy using 1,3,5-trimethoxybenzene (0.030 mmol) as an internal standard. The crude ^{1}H NMR spectrum is shown below.

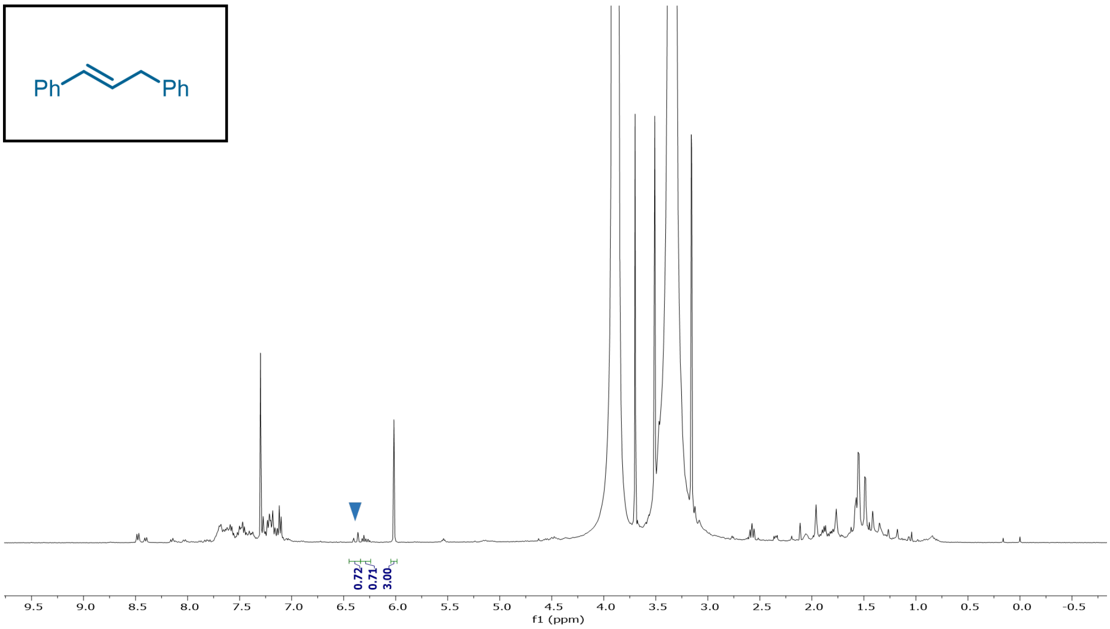

Figure S40 – ^{1}H NMR (400 MHz, $CDCl_3$, 298 K) spectrum of the crude reaction mixture obtained from the conversion of **3a** to **4a** using **[Rh–H]**.

11.11 Deuterium-Labeling Experiment under *Condition B*

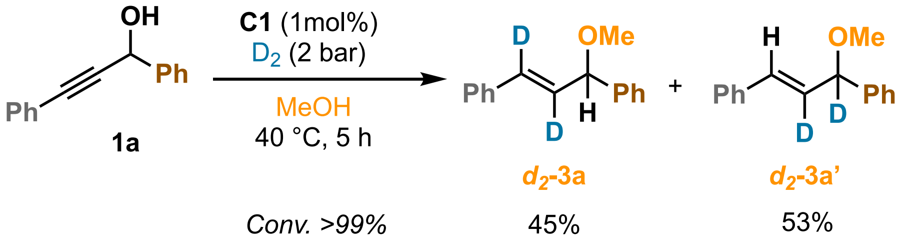

In an oven-dried Fisher–Porter tube under an argon atmosphere, propargylic alcohol **1a** (52.1 mg, 0.25 mmol), complex **C1** (3.4 mg, 1 mol%), and MeOH (1 mL) were added. The mixture was flash-frozen using liquid nitrogen, evacuated, and then pressurized with D_2 (2 bar). The sealed tube was stirred at 40 °C for 5 h. After completion, the reaction was cooled to room temperature, and the pressure was carefully released. The solvent was removed under reduced pressure. Conversion and yield were determined by ^{1}H NMR spectroscopy using mesitylene (0.50 mmol) as an internal standard. The crude ^{1}H NMR spectrum is shown below.

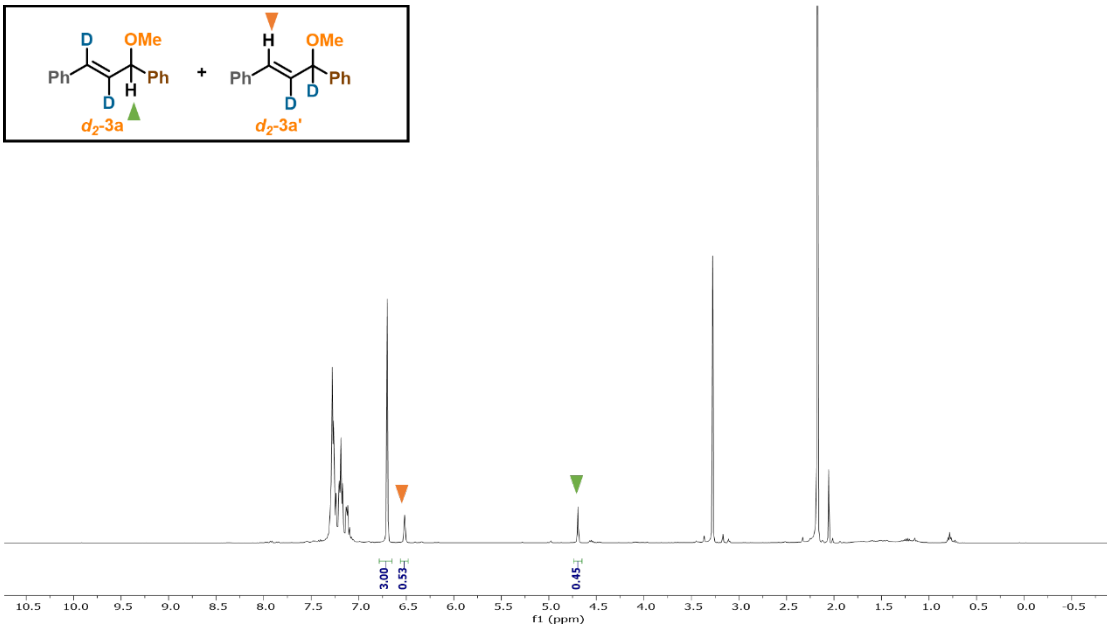

Figure S41 – [1]**H NMR (400 MHz, $CDCl_3$, 298 K)** spectrum of the crude reaction mixture obtained from the deuterium-labeling experiment of **1a** with D_2 under *Condition B.*

11.12 Control Experiments with 3m

11.12.1 Under *Condition B* with CD_3OD

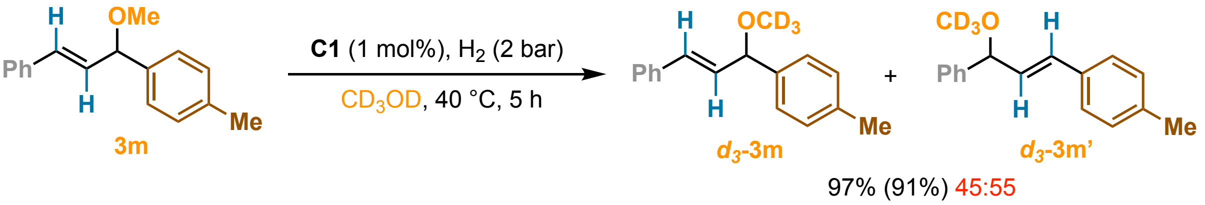

Compound **3m** (0.25 mmol) afforded a 97% combined yield of isomers ***d₃*-3m** and ***d₃*-3m'**, as determined by ^{1}H NMR spectroscopy using mesitylene (0.25 mmol) as an internal standard. The total isolated yield was 91%.

(*E*)-1-(1-(methoxy-*d*$_3$)-3-phenylallyl)-4-methylbenzene (*d*$_3$-3m)

(*E*)-1-(3-(methoxy-*d*$_3$)-3-phenylprop-1-en-1-yl)-4-methylbenzene (*d*$_3$-3m')

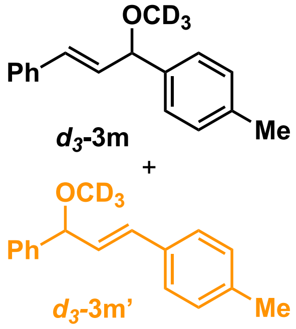

Formula: $C_{17}H_{15}D_3O$

Molecular weight: 241.35

Isolated yield: 91% (55 mg), colorless oil

Rf: 0.4 (Pentane/EtOAc = 50:1)

^{1}H NMR (400 MHz, CDCl$_3$, 298 K) δ 7.37 – 7.22 (m, 6H), 7.20 – 7.01 (m, 3H), 6.63 – 6.51 (m, 1H), 6.30 – 6.14 (m, 1H), 4.82 – 4.65 (m, 1H), 2.31 (s, 0.55×3H), 2.28 (s, 0.45×3H)

^{13}C NMR (101 MHz, CDCl$_3$, 298 K) δ 141.3, 138.1, 137.7, 137.5, 136.8, 133.9, 131.6, 131.3, 130.5, 129.4, 129.2, 128.6, 127.8, 127.0, 126.7, 126.6, 84.4, 84.2, 21.3, 21.3

HRMS (GC-EI): Calcd. for $C_{17}H_{15}D_3O^+$ [M]$^+$: 241.15405; Found 241.15425 and 241.15426

11.12.2 Under *Condition C*

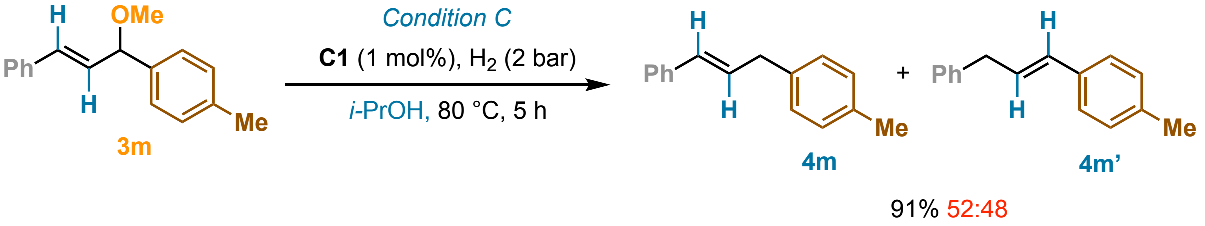

Compound **3m** (0.25 mmol) afforded a 91% combined yield of isomers **4m** and **4m'**, as determined by ^{1}H NMR spectroscopy using mesitylene (0.25 mmol) as an internal standard. The crude ^{1}H NMR spectrum is shown below.

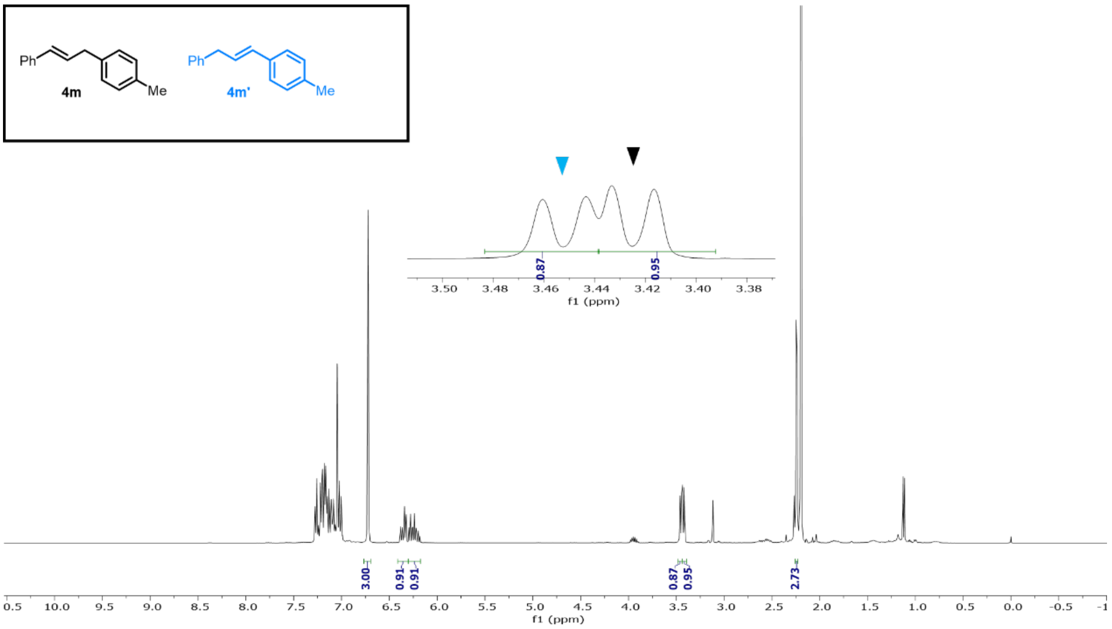

Figure S42 – ^{1}H NMR (400 MHz, $CDCl_3$, 298 K) spectrum of the crude reaction mixture obtained from the conversion of **3m** to **4m/4m'** under *Condition C*.

12. NMR spectra

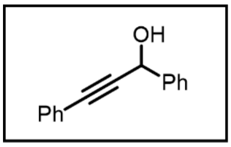

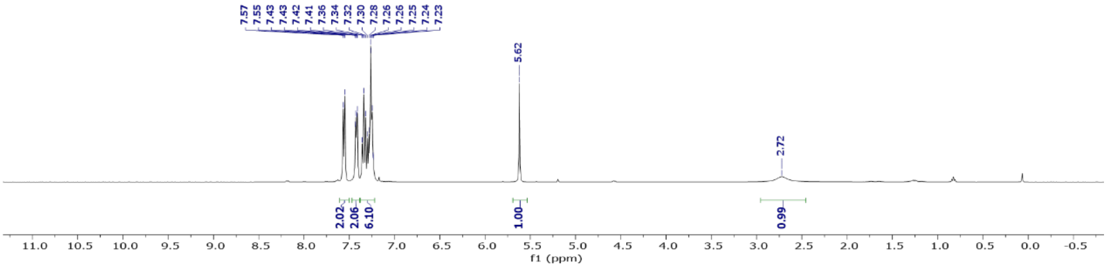

Figure S43 – ¹H NMR (400 MHz, $CDCl_3$, 298 K) spectrum of **1a**.

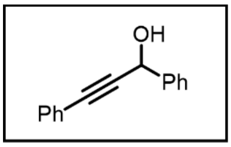

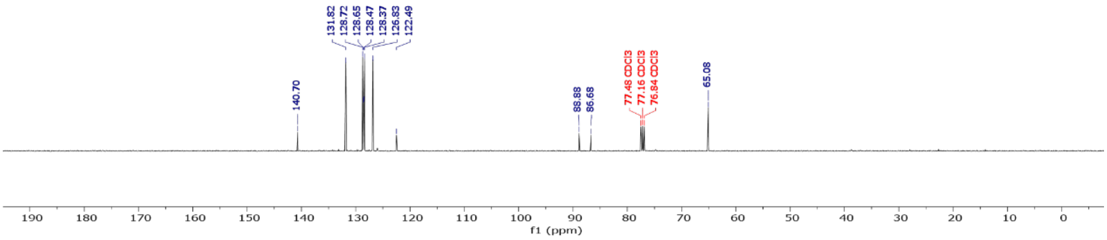

Figure S44 – ¹³C NMR (101 MHz, $CDCl_3$, 298 K) spectrum of **1a**.

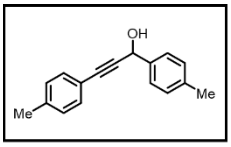

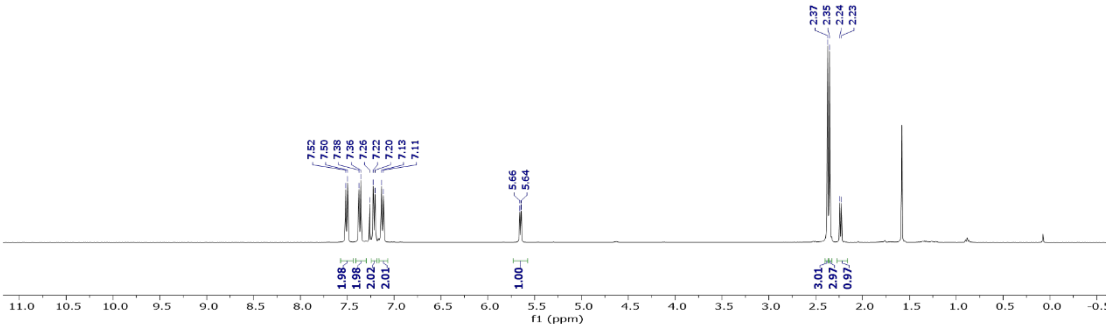

Figure S45 – ^{1}H NMR (400 MHz, $CDCl_3$, 298 K) spectrum of **1b**.

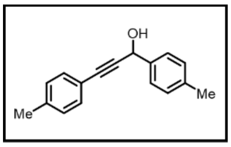

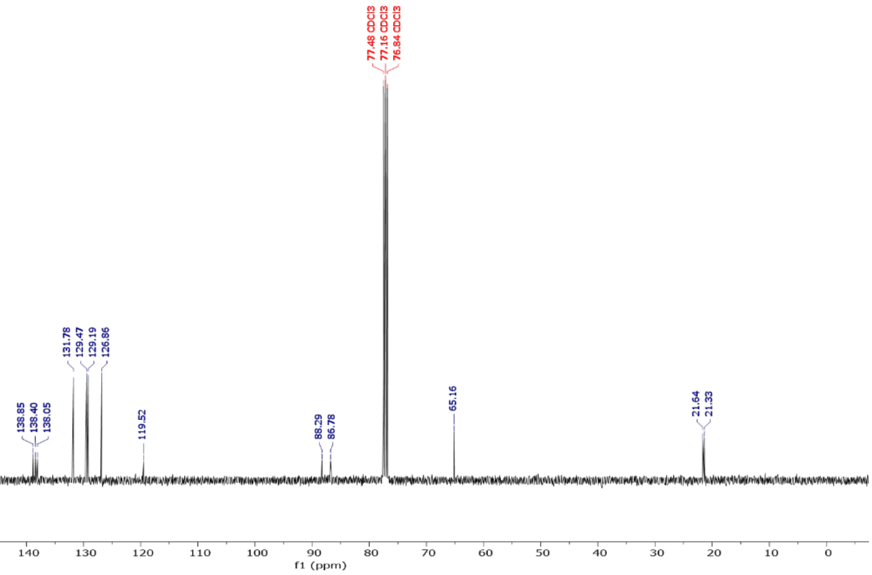

Figure S46 – ^{13}C NMR (101 MHz, $CDCl_3$, 298 K) spectrum of **1b**.

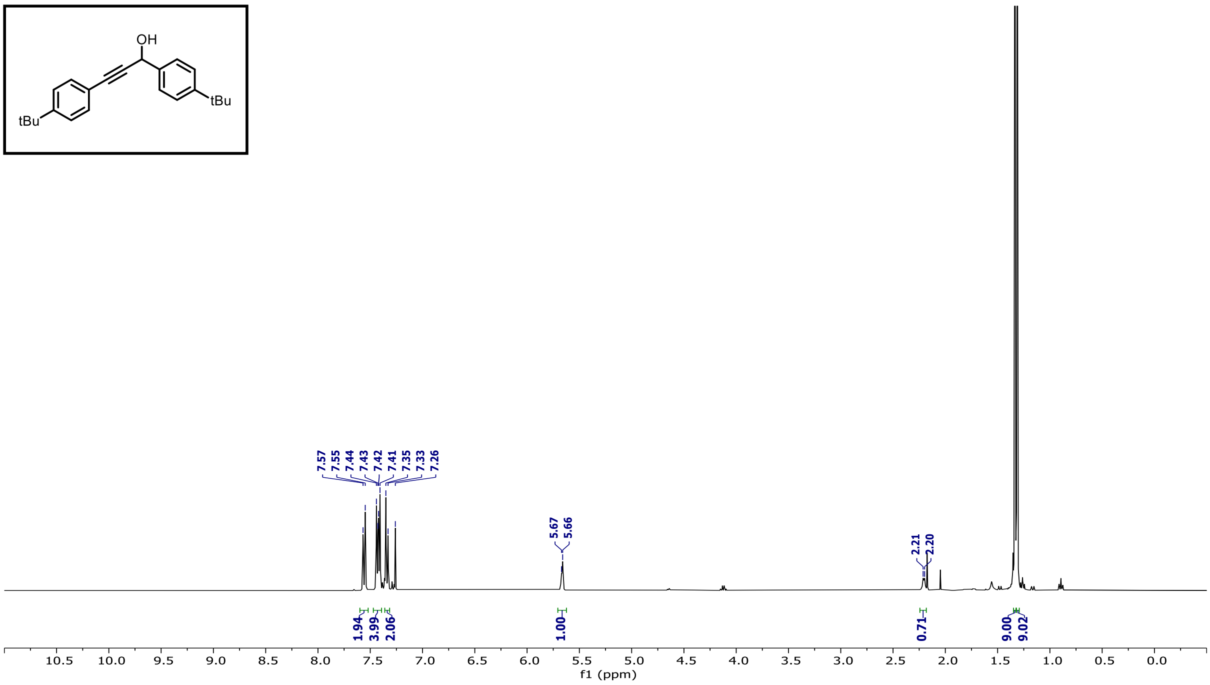

Figure S47 – ^{1}H NMR (400 MHz, CDCl$_3$, 298 K) spectrum of **1c**.

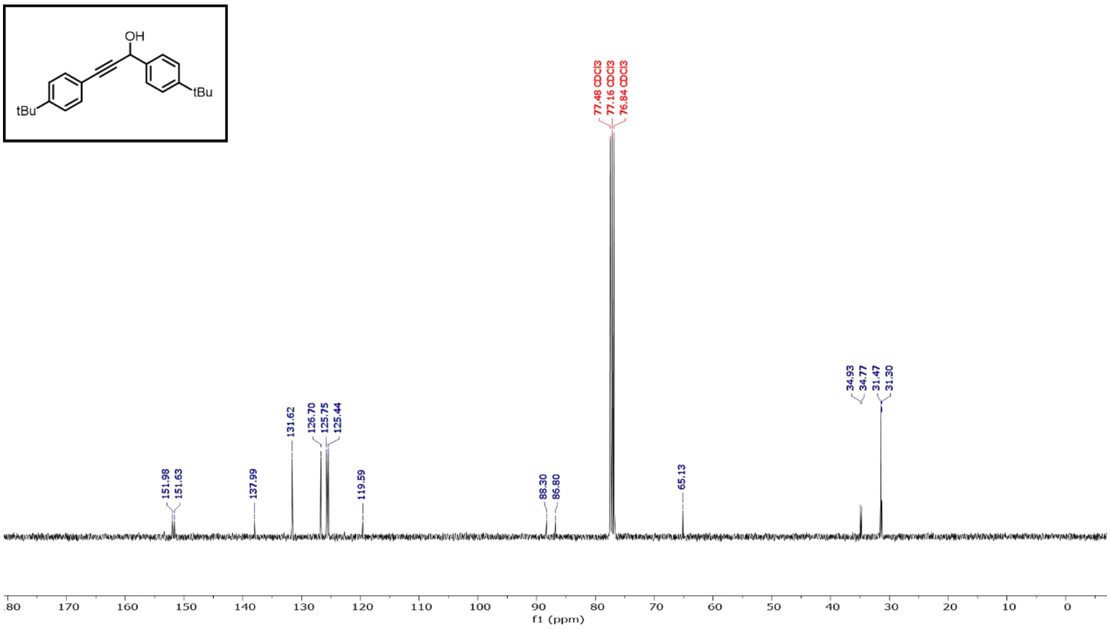

Figure S48 – ^{13}C NMR (101 MHz, CDCl$_3$, 298 K) spectrum of **1c**.

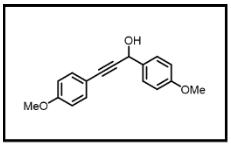

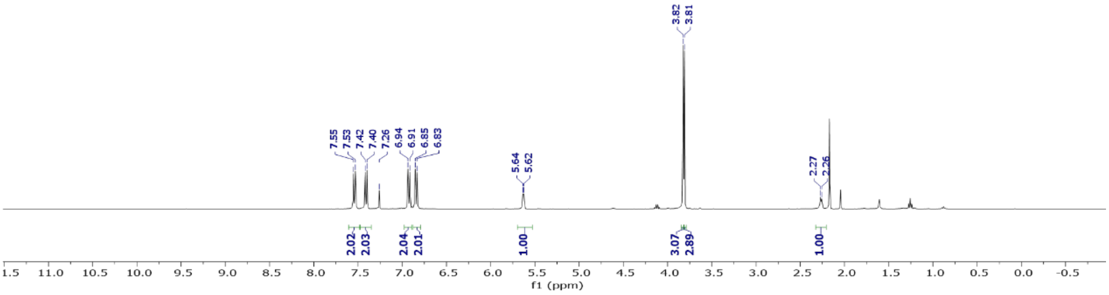

Figure S49 – ^{1}H NMR (400 MHz, CDCl$_3$, 298 K) spectrum of **1d**.

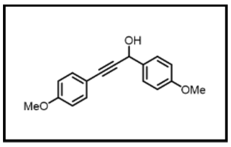

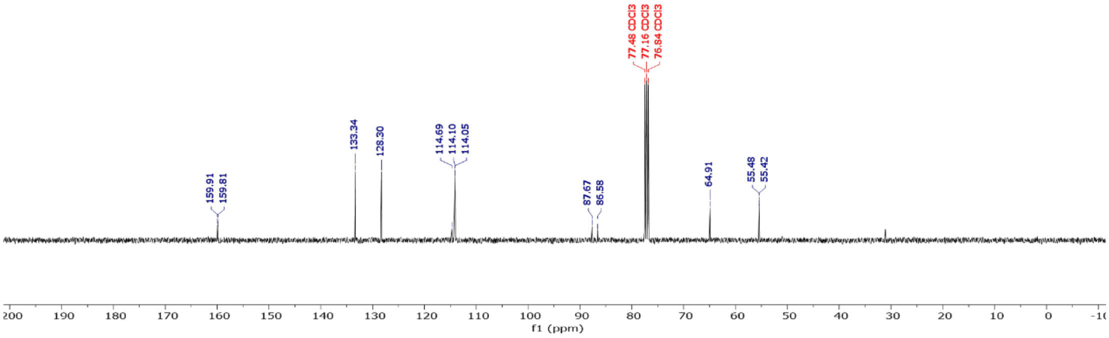

Figure S50 – ^{13}C NMR (101 MHz, CDCl$_3$, 298 K) spectrum of **1d**.

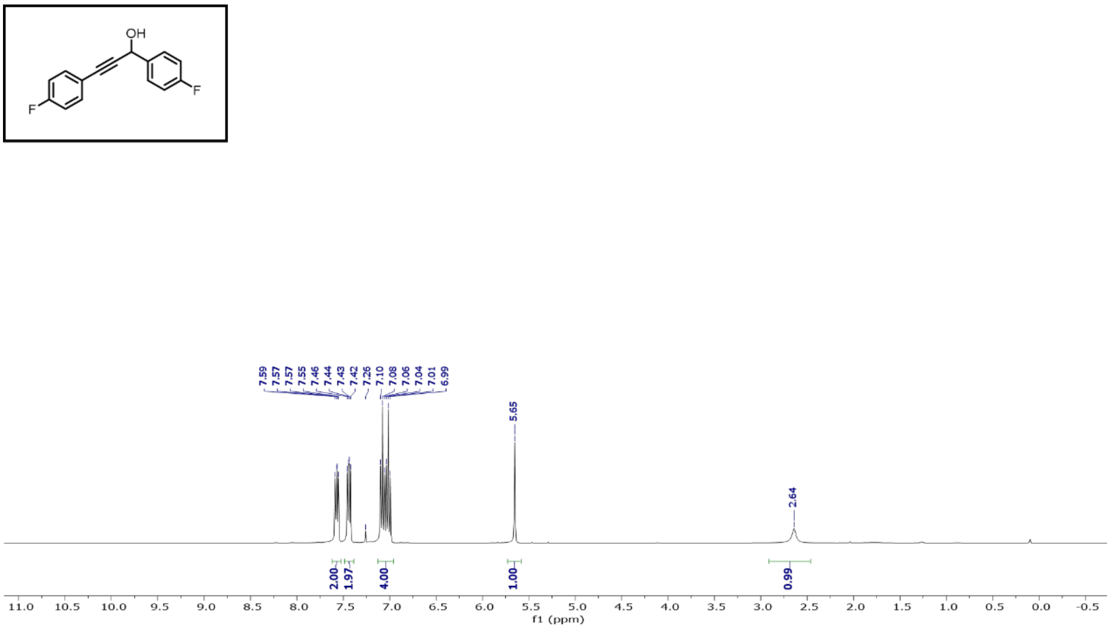

Figure S51 – ^{1}H NMR (400 MHz, $CDCl_3$, 298 K) spectrum of **1e**.

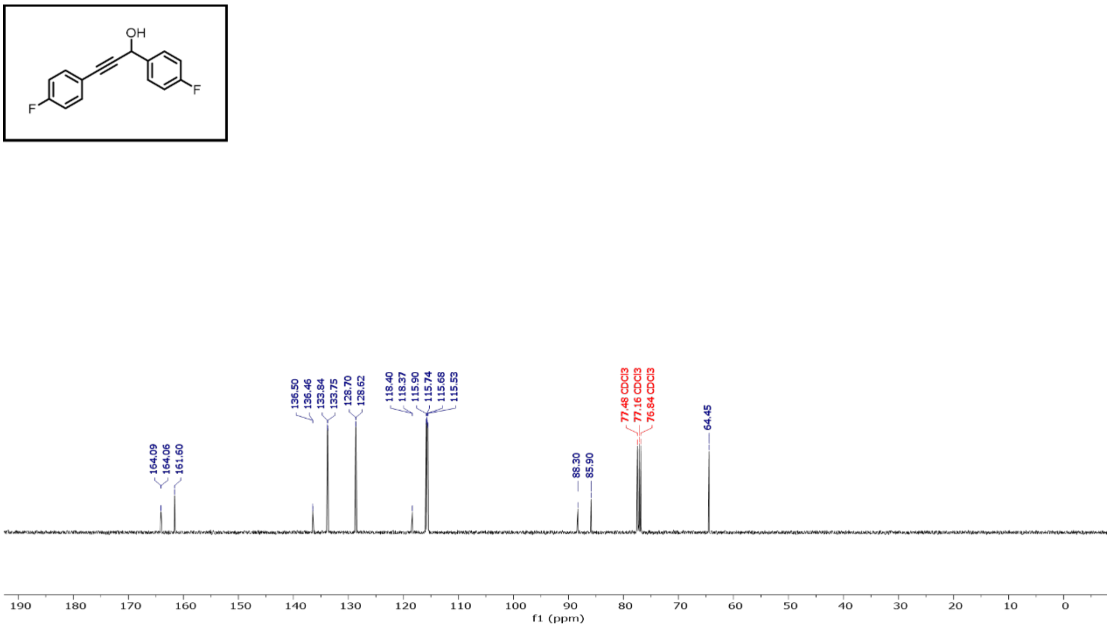

Figure S52 – ^{13}C NMR (101 MHz, $CDCl_3$, 298 K) spectrum of **1e**.

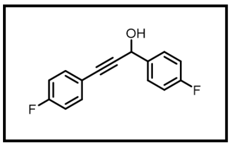

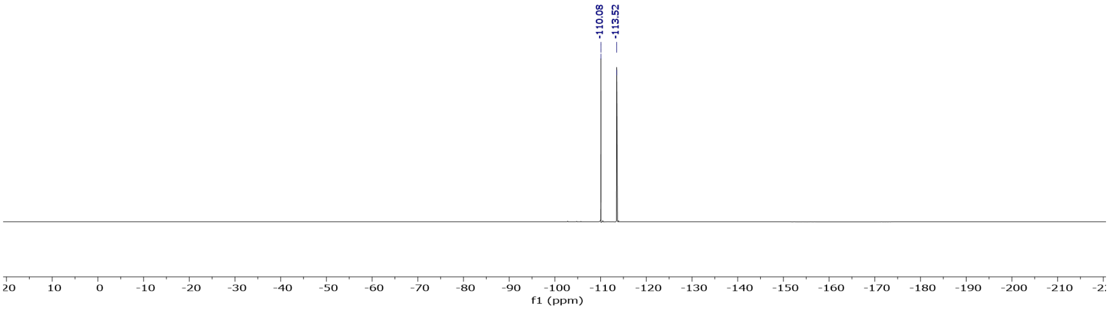

Figure S53 – ^{19}F NMR (376 MHz, $CDCl_3$, 298 K) spectrum of the reaction from **1e**.

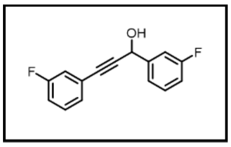

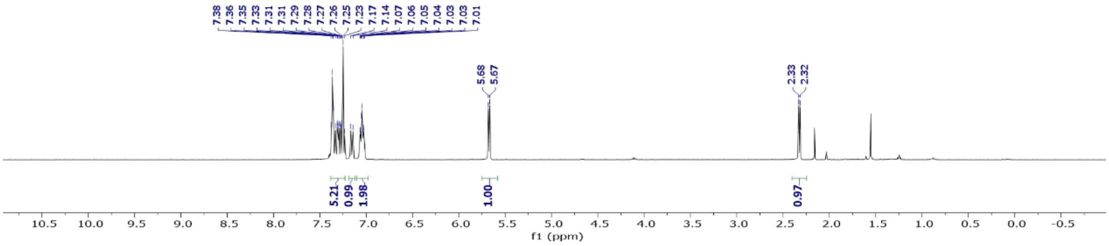

Figure S54 – ^{1}H NMR (400 MHz, $CDCl_3$, 298 K) spectrum of **1f**.

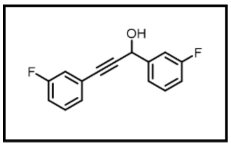

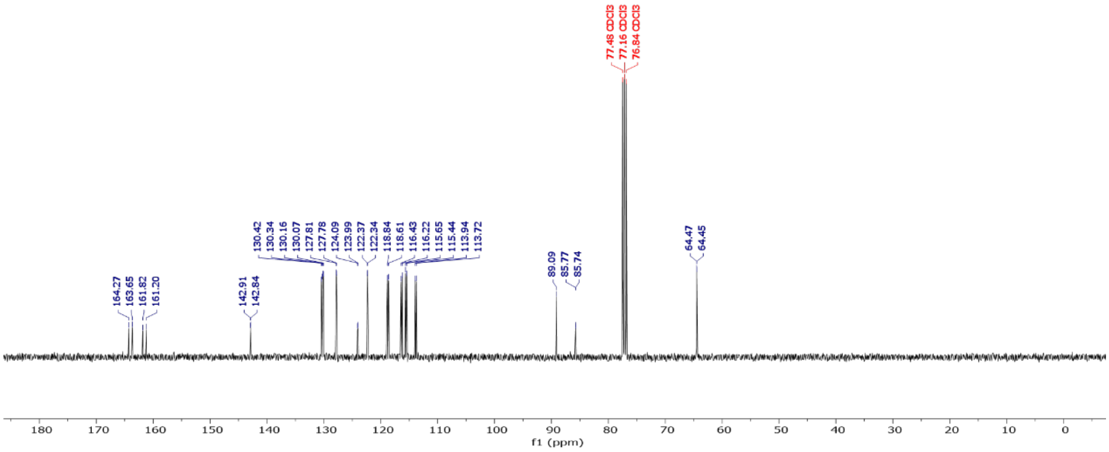

Figure S55 – ^{13}C NMR (101 MHz, CDCl$_3$, 298 K) spectrum of **1f**.

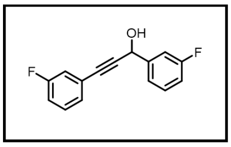

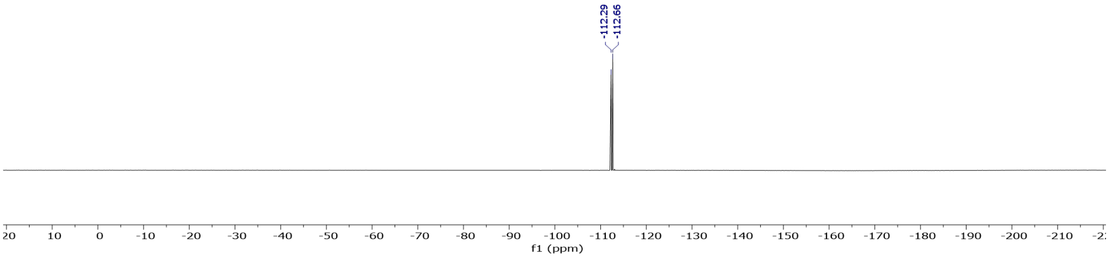

Figure S56 – ^{19}F NMR (376 MHz, CDCl$_3$, 298 K) spectrum of the reaction from **1f**.

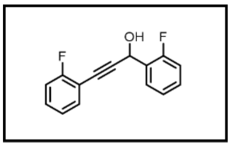

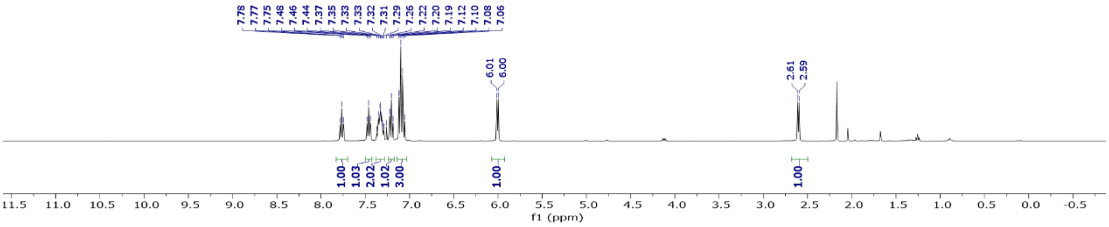

Figure S57 – ^{1}H NMR (400 MHz, CDCl$_3$, 298 K) spectrum of **1g**.

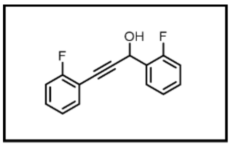

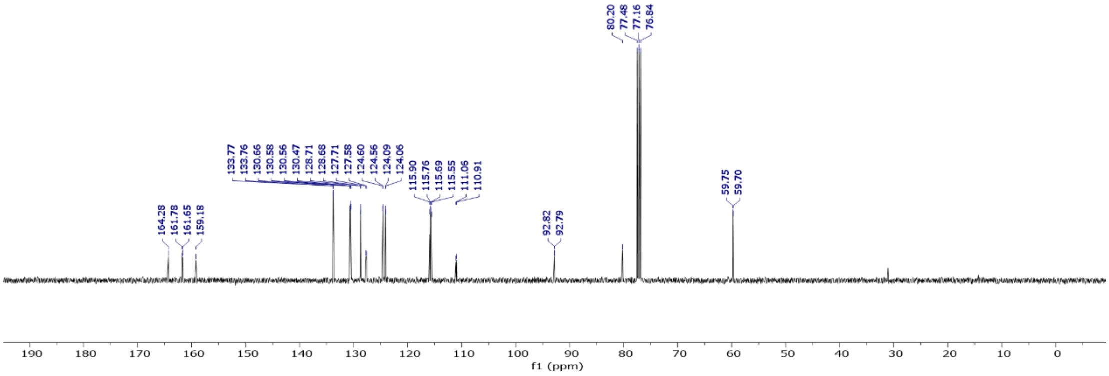

Figure S58 – ^{13}C NMR (101 MHz, CDCl$_3$, 298 K) spectrum of **1g**.

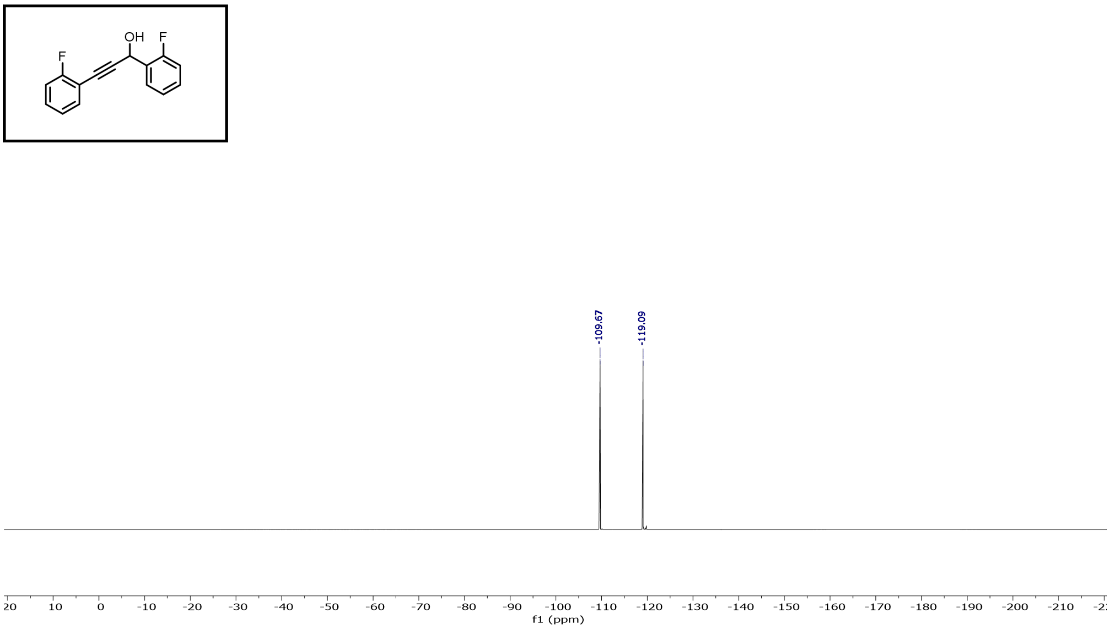

Figure S59 – ^{19}F NMR (376 MHz, $CDCl_3$, 298 K) spectrum of the reaction from **1g**.

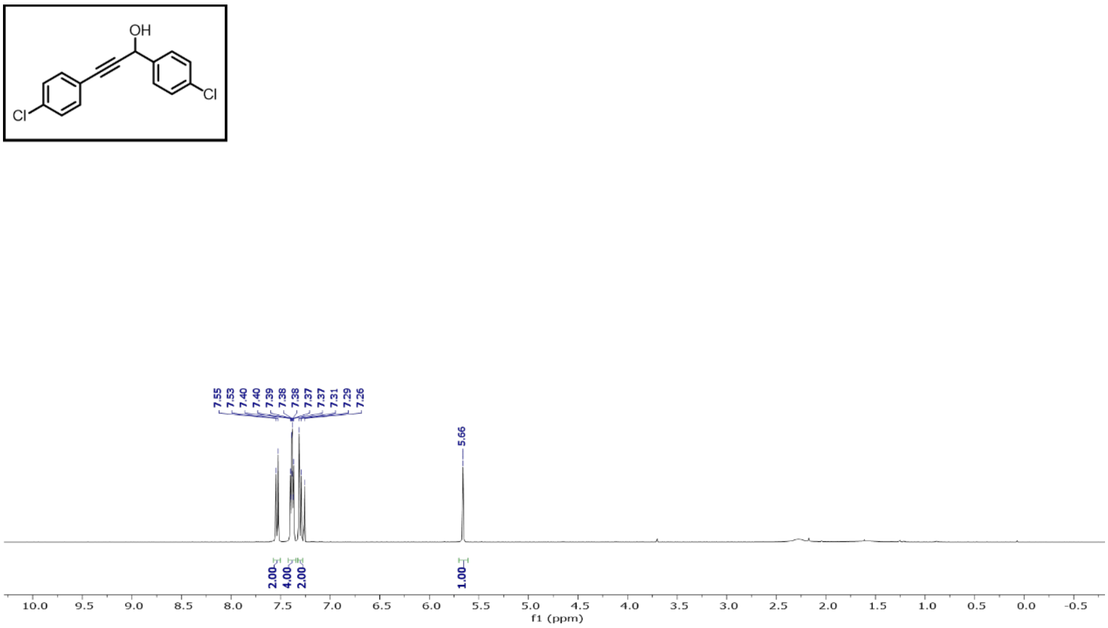

Figure S60 – ^{1}H NMR (400 MHz, $CDCl_3$, 298 K) spectrum of **1h**.

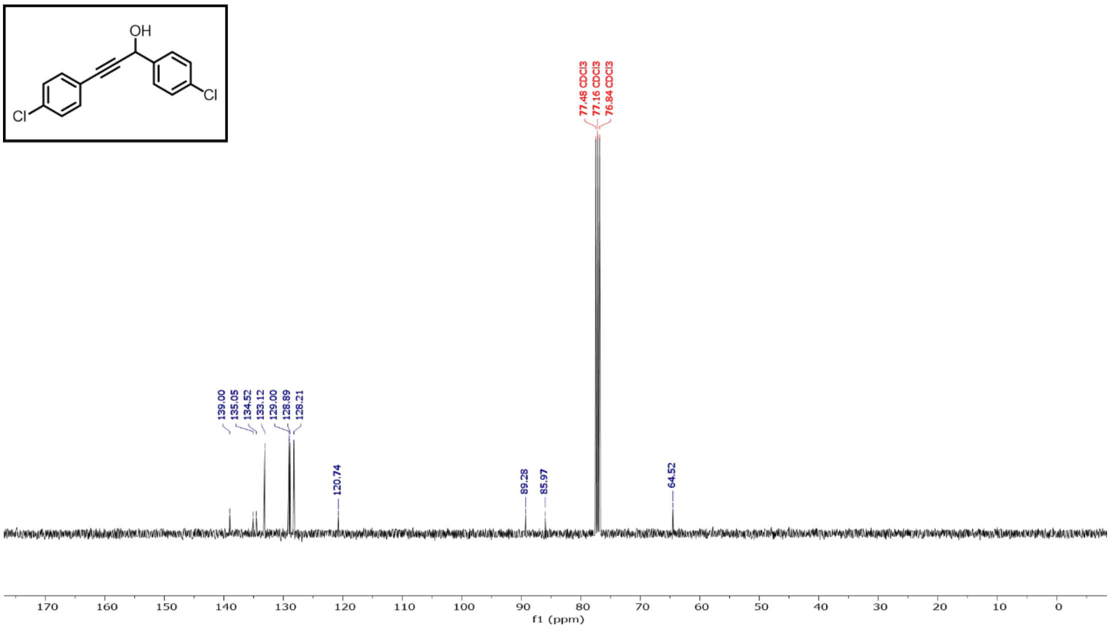

Figure S61 – ^{13}C NMR (101 MHz, $CDCl_3$, 298 K) spectrum of **1h**.

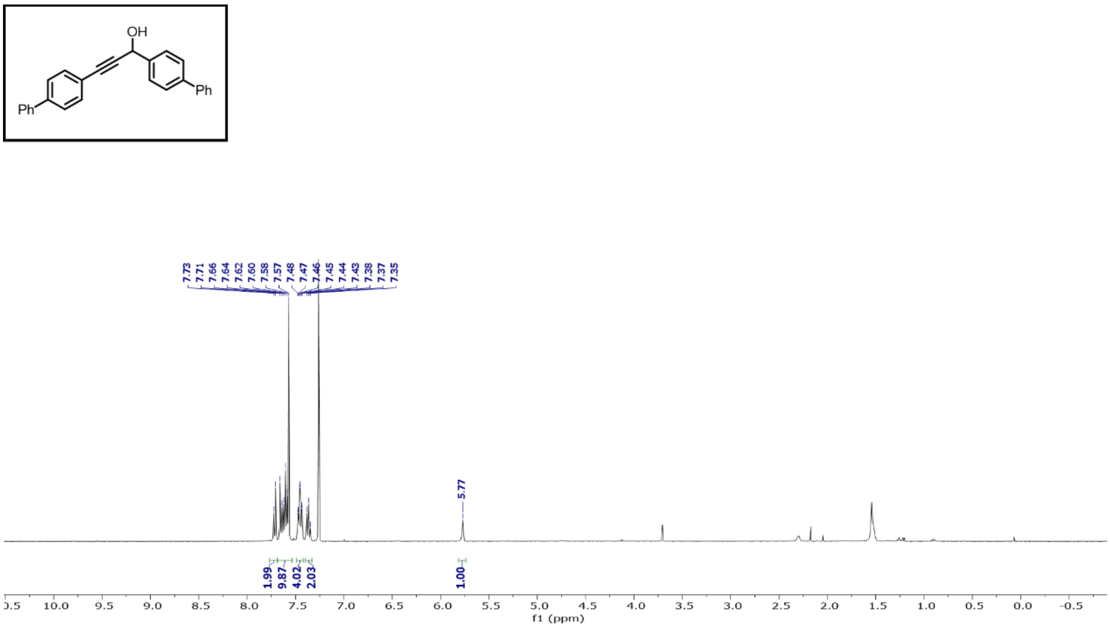

Figure S62 – ^{1}H NMR (400 MHz, $CDCl_3$, 298 K) spectrum of **1i**.

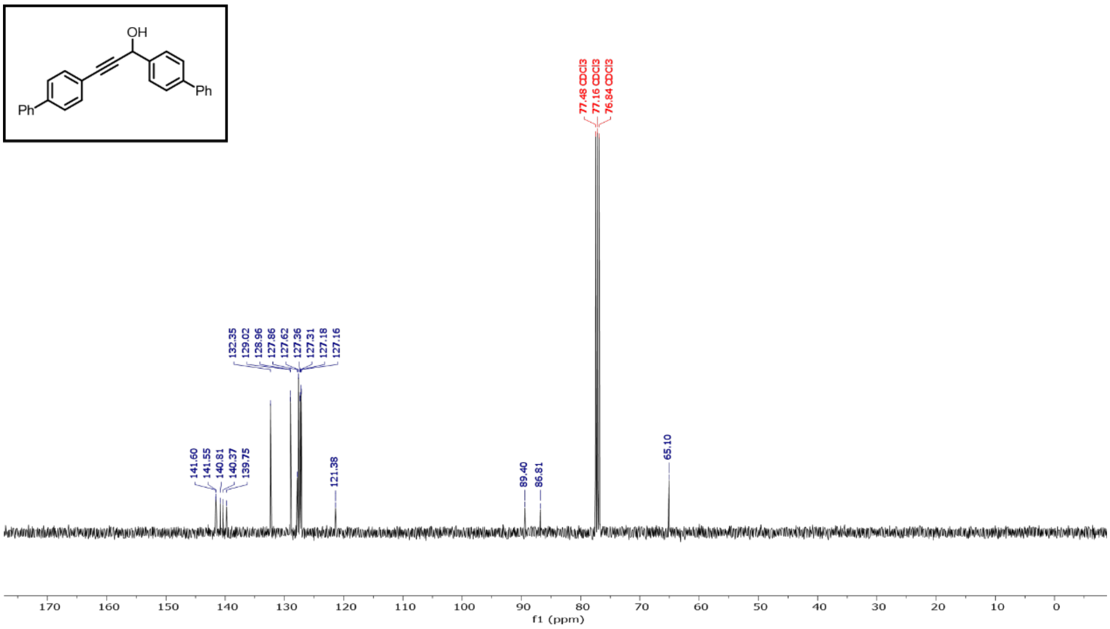

Figure S63 – ^{13}C NMR (101 MHz, CDCl$_3$, 298 K) spectrum of **1i**.

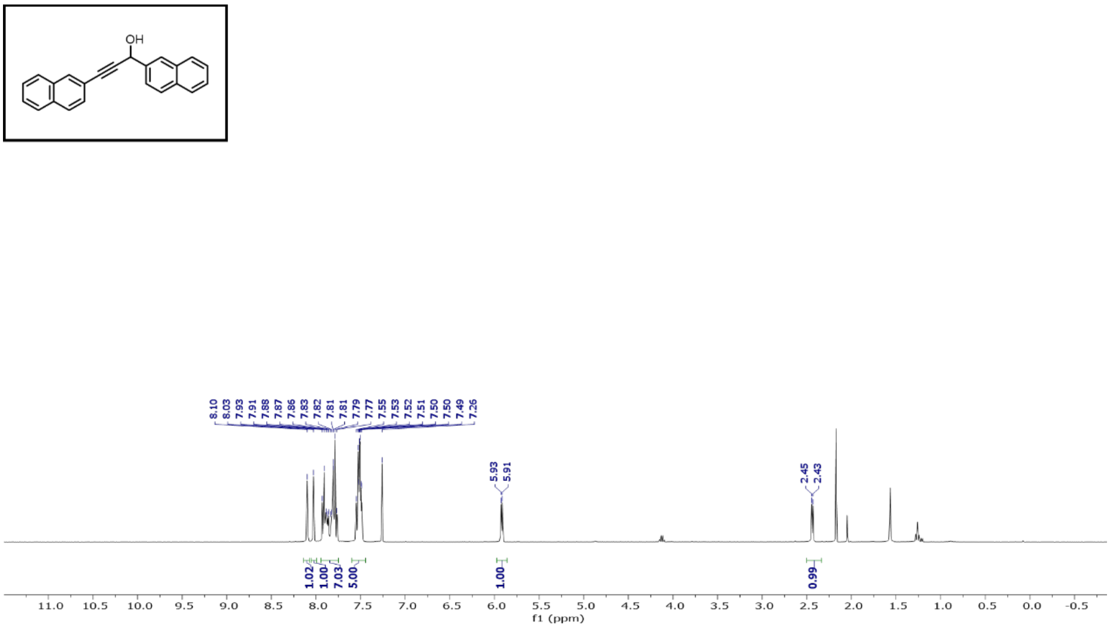

Figure S64 – ^{1}H NMR (400 MHz, CDCl$_3$, 298 K) spectrum of **1j**.

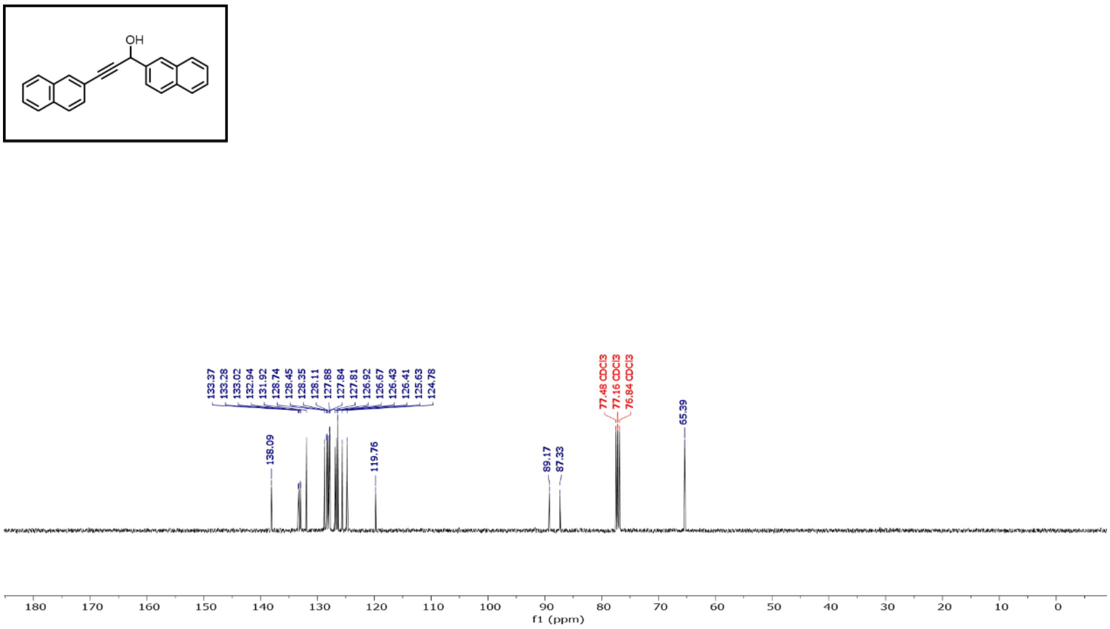

Figure S65 – ^{13}C NMR (101 MHz, $CDCl_3$, 298 K) spectrum of **1j**.

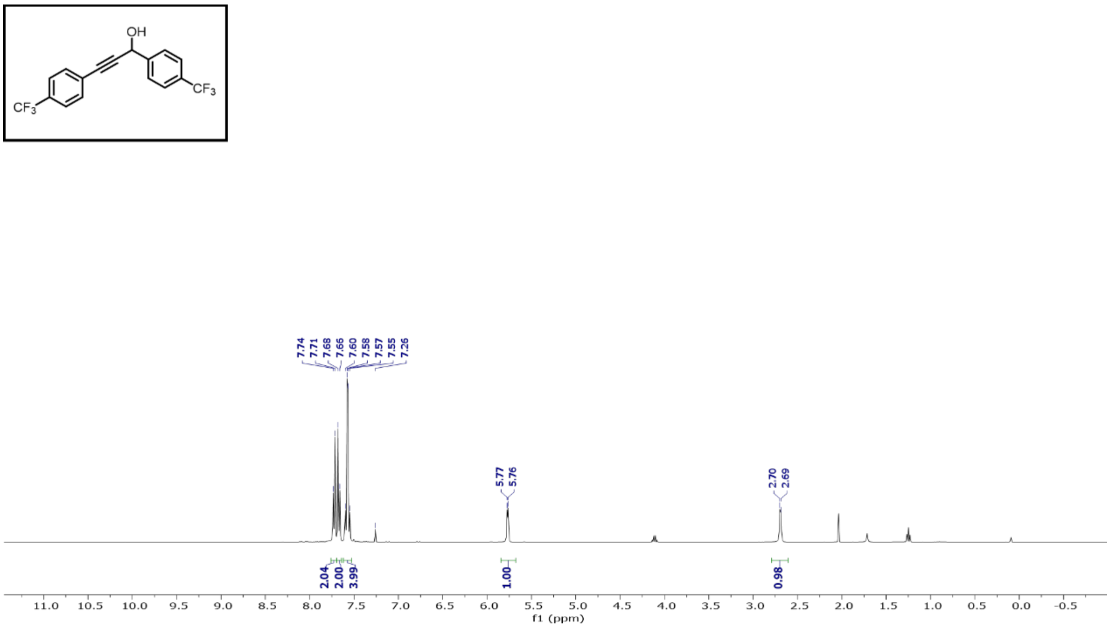

Figure S66 – ^{1}H NMR (400 MHz, $CDCl_3$, 298 K) spectrum of **1k**.

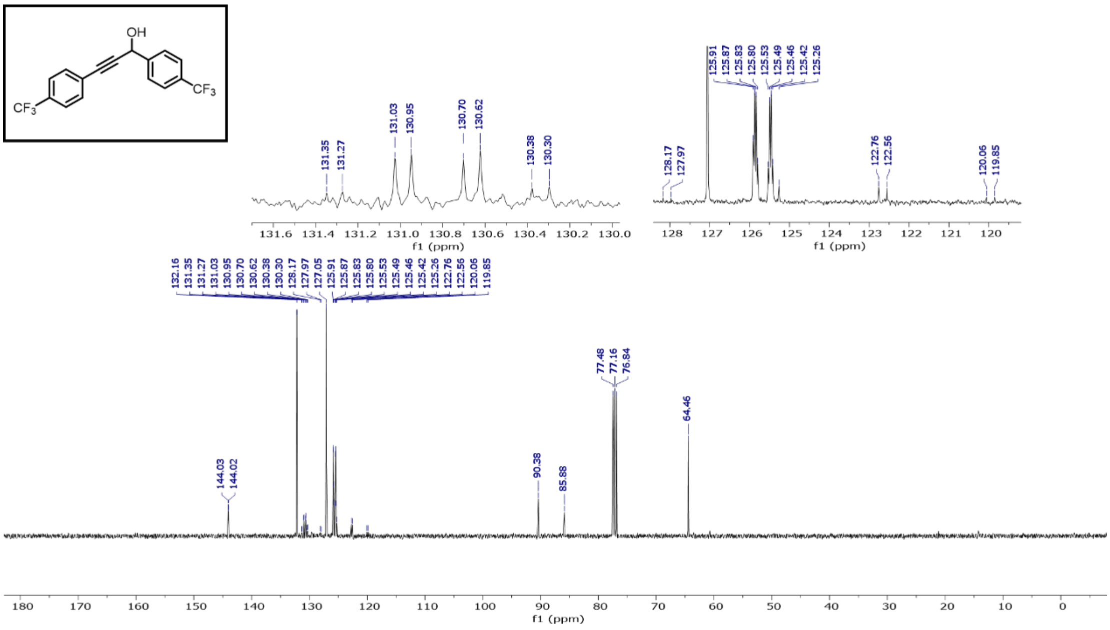

Figure S67 – ^{13}C NMR (101 MHz, $CDCl_3$, 298 K) spectrum of **1k**.

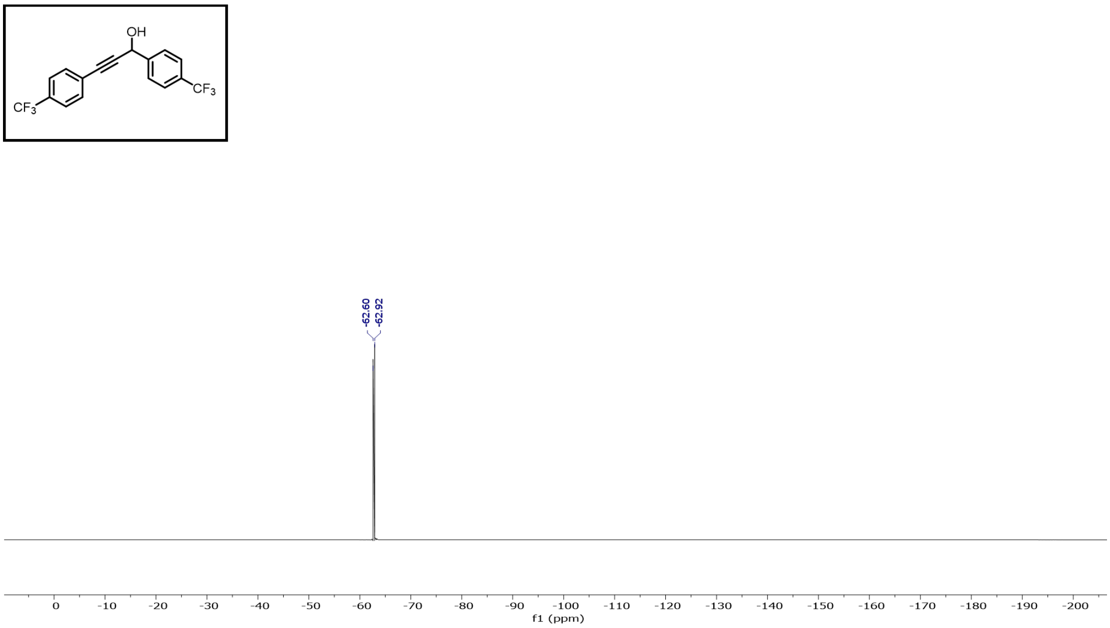

Figure S68 – ^{19}F NMR (376 MHz, $CDCl_3$, 298 K) spectrum of the reaction from **1k**.

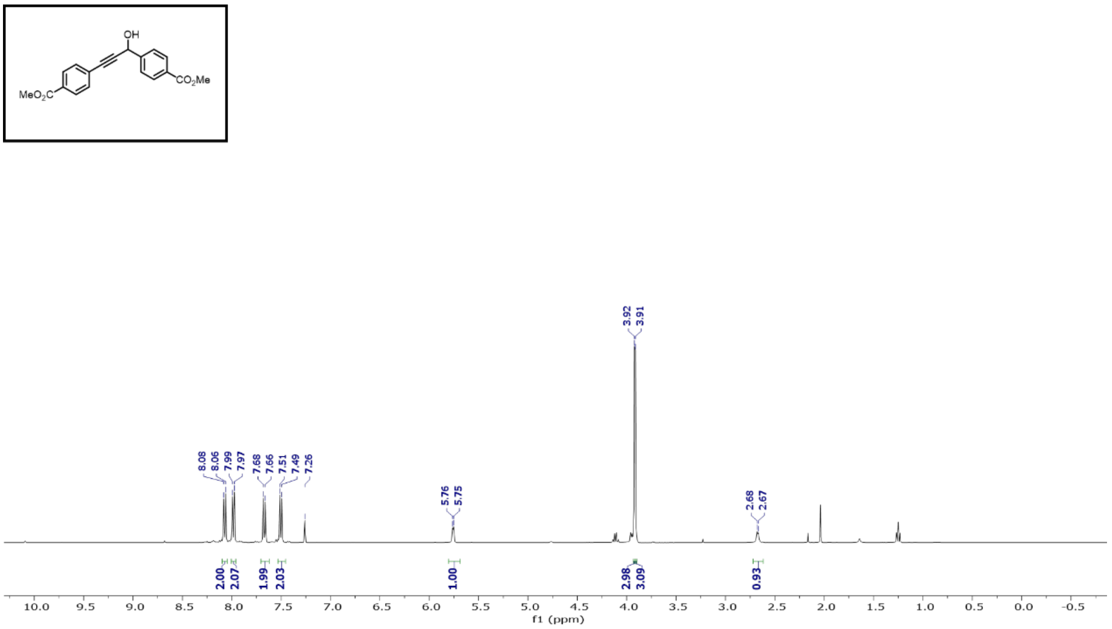

Figure S69 – ^{1}H NMR (400 MHz, $CDCl_3$, 298 K) spectrum of **1l**.

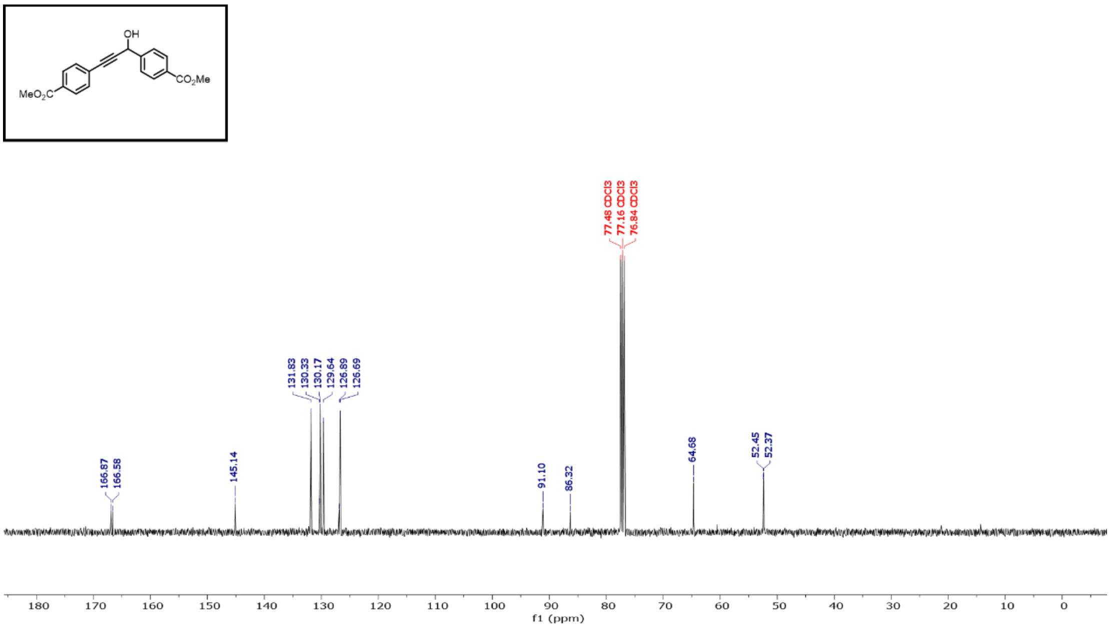

Figure S70 – ^{13}C NMR (101 MHz, $CDCl_3$, 298 K) spectrum of **1l**.

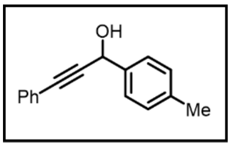

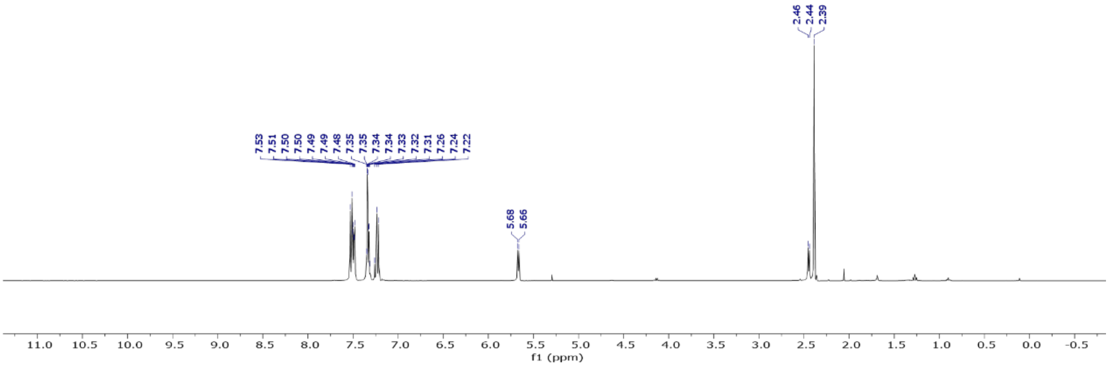

Figure S71 – ^{1}H NMR (400 MHz, CDCl$_3$, 298 K) spectrum of **1m**.

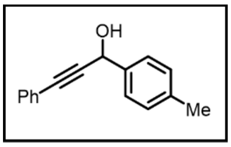

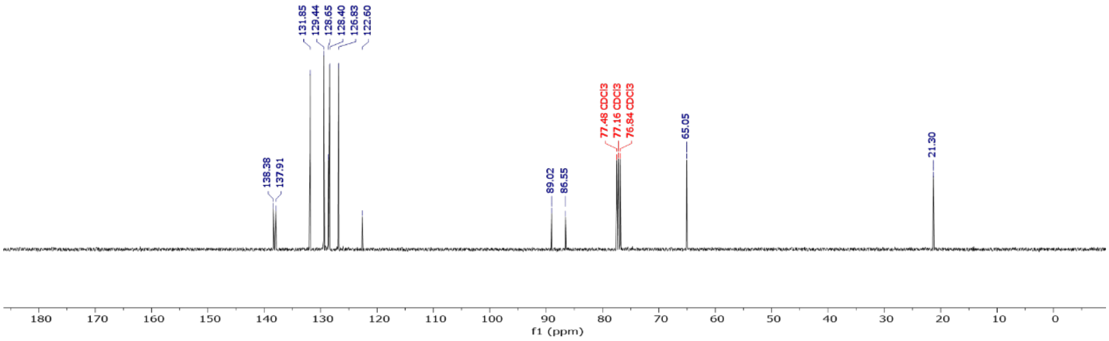

Figure S72 – ^{13}C NMR (101 MHz, CDCl$_3$, 298 K) spectrum of **1m**.

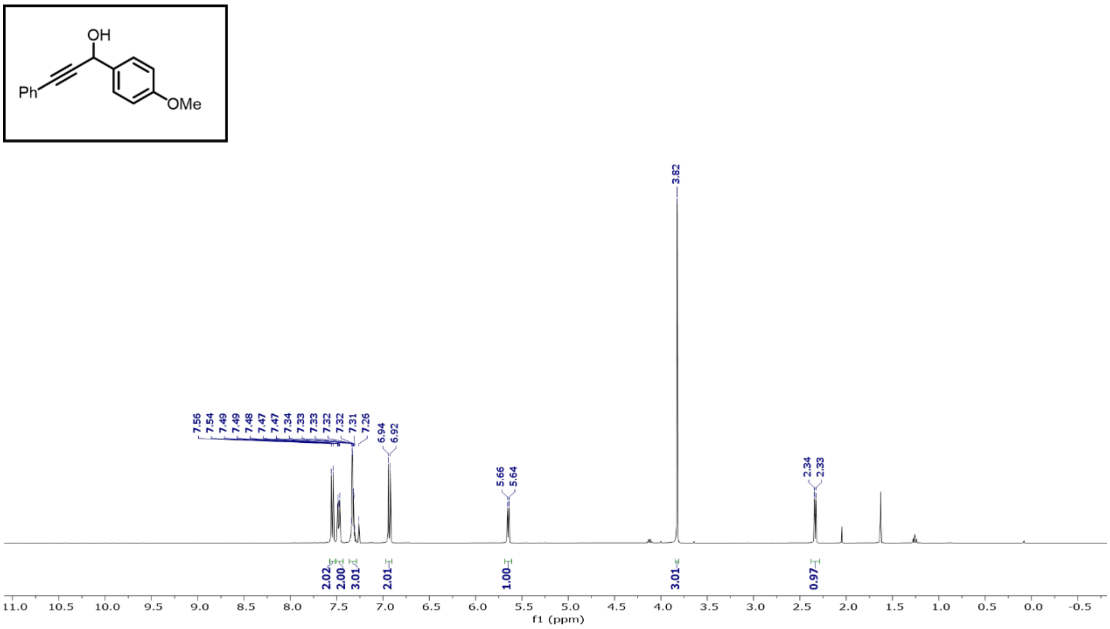

Figure S73 – ^{1}H NMR (400 MHz, $CDCl_3$, 298 K) spectrum of **1n**.

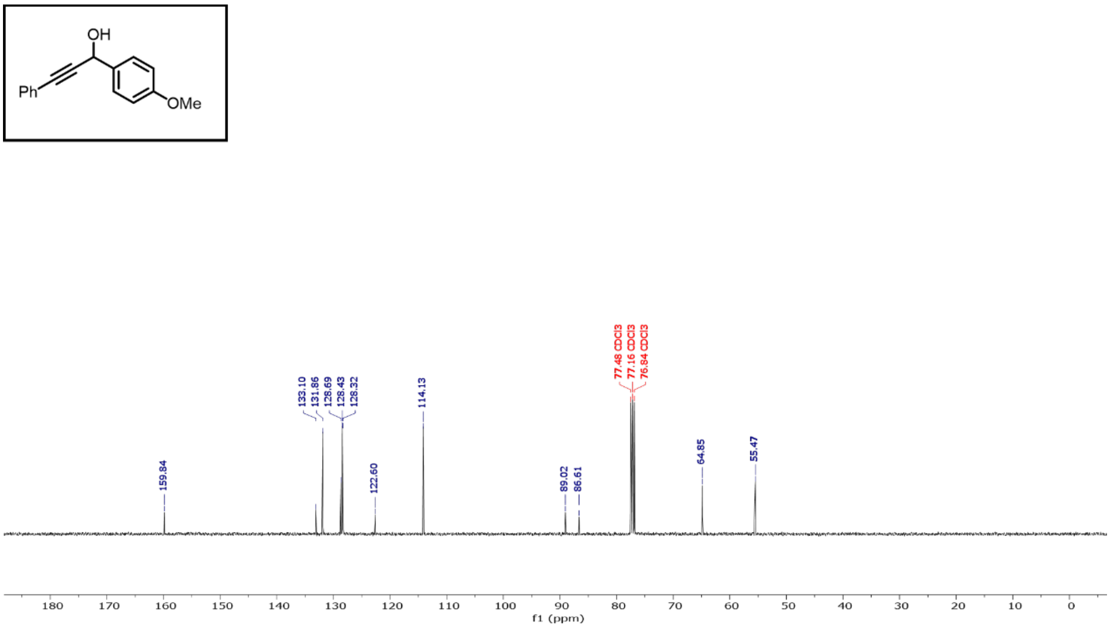

Figure S74 – ^{13}C NMR (101 MHz, $CDCl_3$, 298 K) spectrum of **1n**.

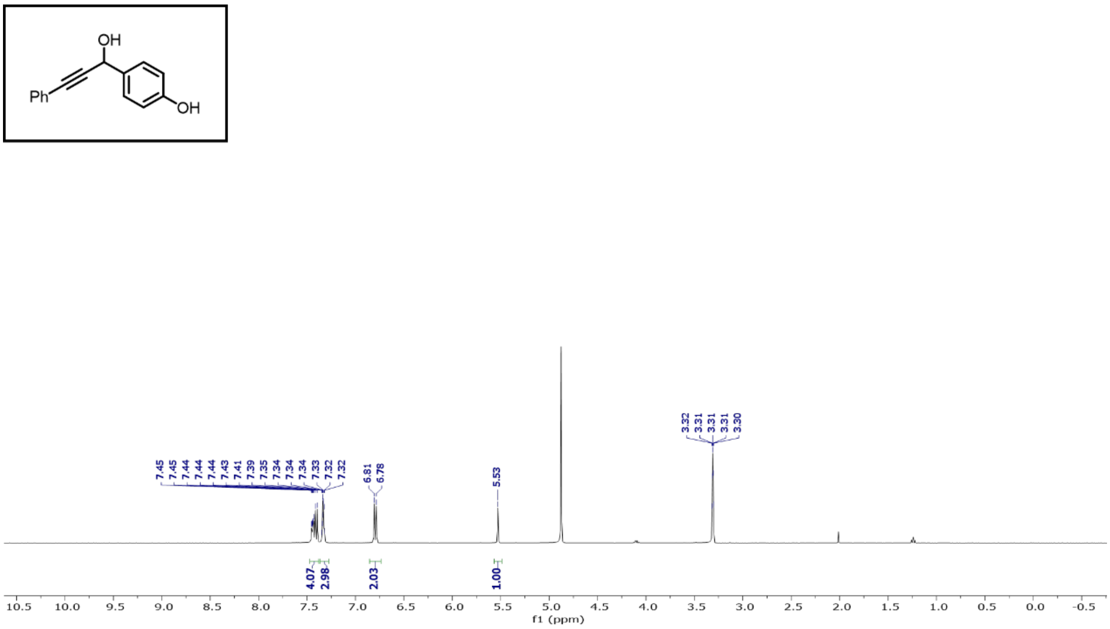

Figure S75 – ^{1}H NMR (400 MHz, $CDCl_3$, 298 K) spectrum of **1o**.

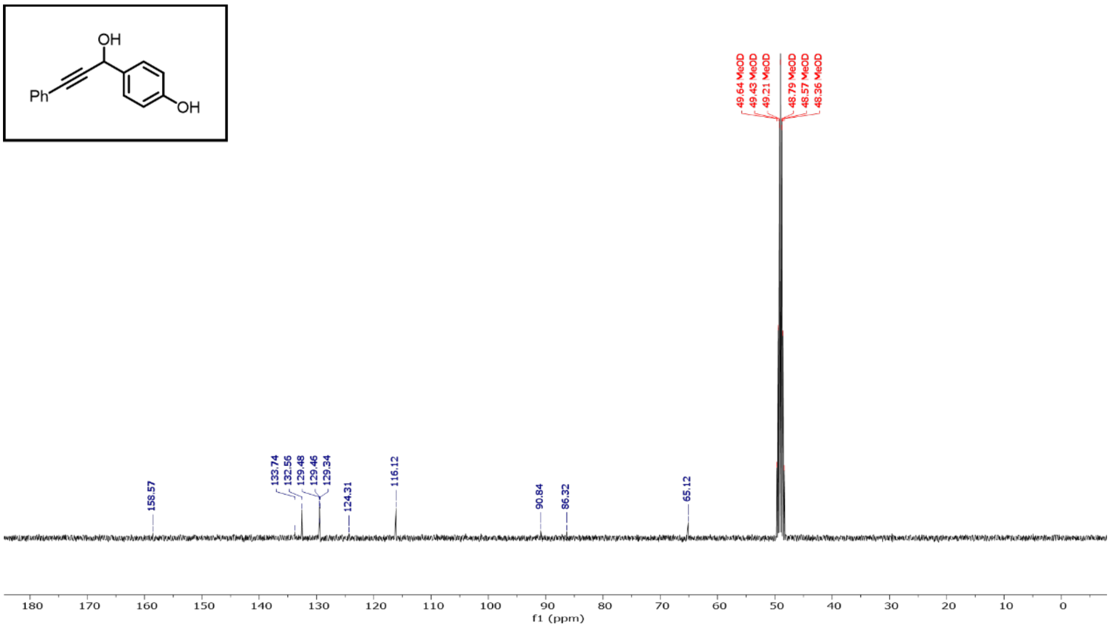

Figure S76 – ^{13}C NMR (101 MHz, $CDCl_3$, 298 K) spectrum of **1o**.

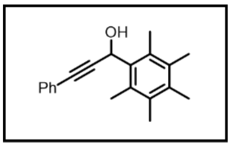

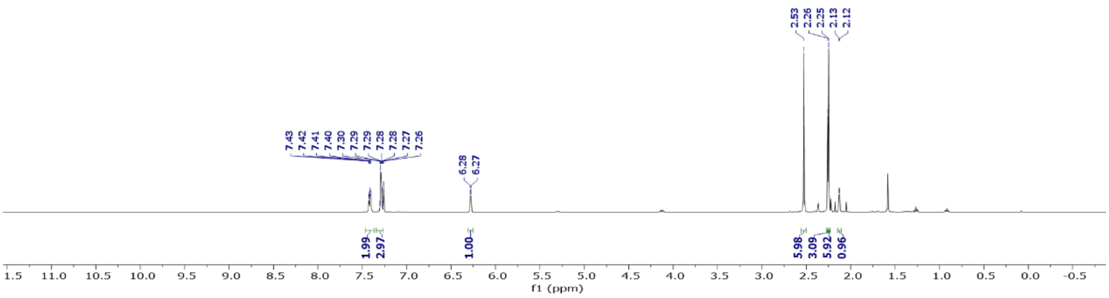

Figure S77 – ^{1}H NMR (400 MHz, $CDCl_3$, 298 K) spectrum of **1p**.

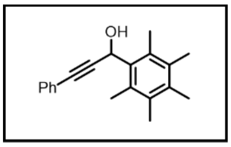

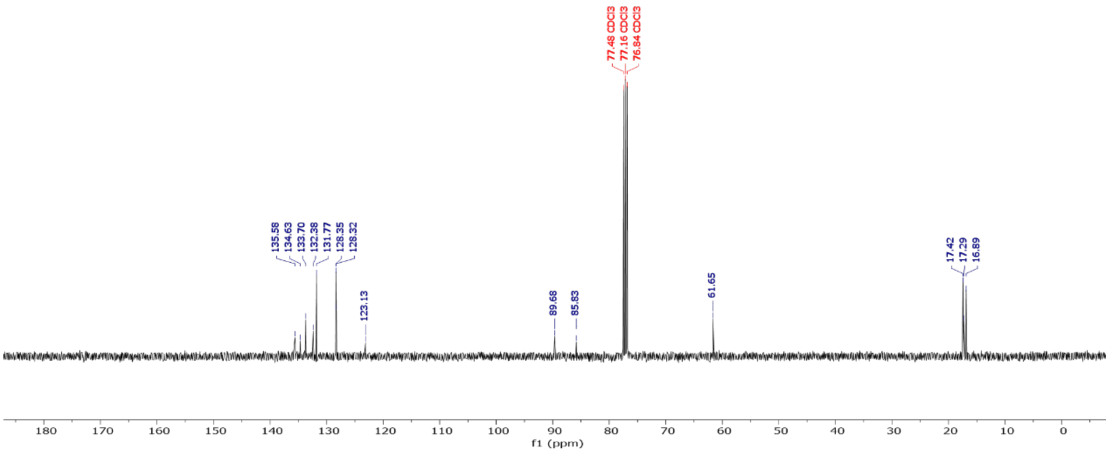

Figure S78 – ^{13}C NMR (101 MHz, $CDCl_3$, 298 K) spectrum of **1p**.

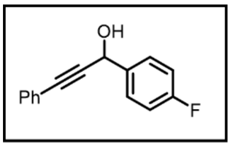

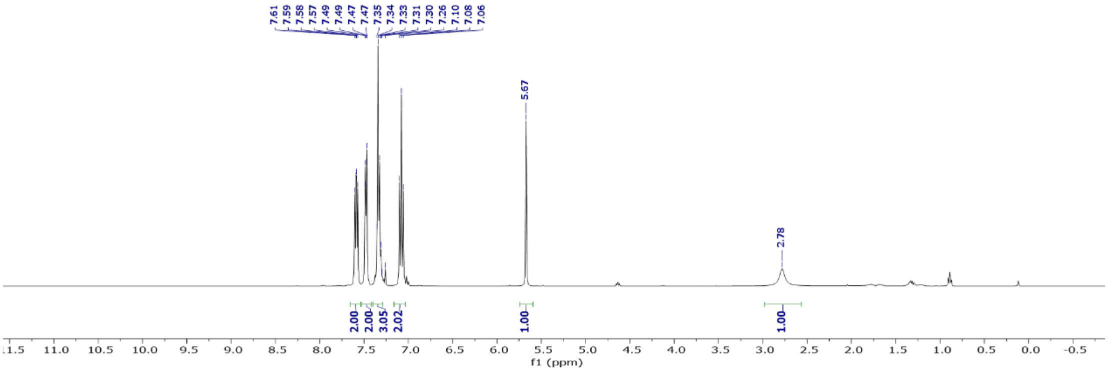

Figure S79 – ^{1}H NMR (400 MHz, CDCl$_3$, 298 K) spectrum of **1q**.

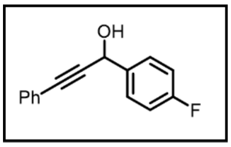

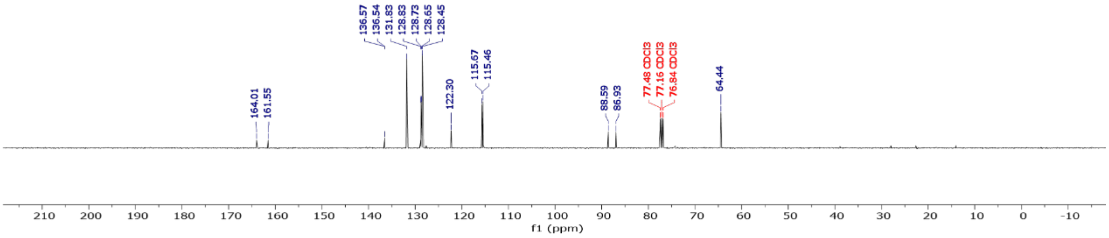

Figure S80 – ^{13}C NMR (101 MHz, CDCl$_3$, 298 K) spectrum of **1q**.

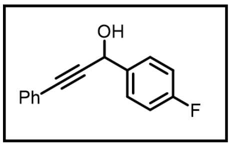

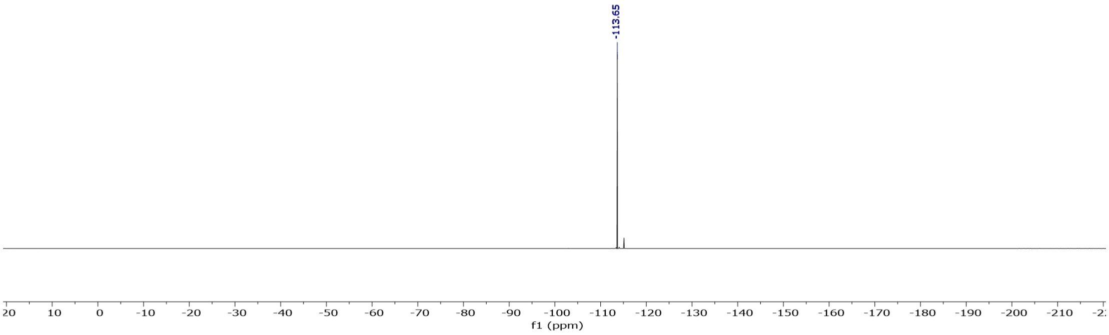

Figure S81 – ^{19}F NMR (376 MHz, CDCl$_3$, 298 K) spectrum of the reaction from **1q**.

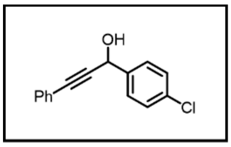

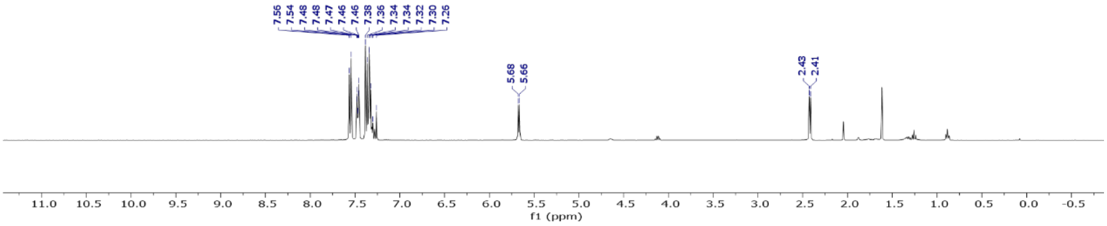

Figure S82 – ^{1}H NMR (400 MHz, CDCl$_3$, 298 K) spectrum of **1r**.

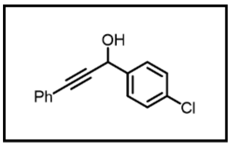

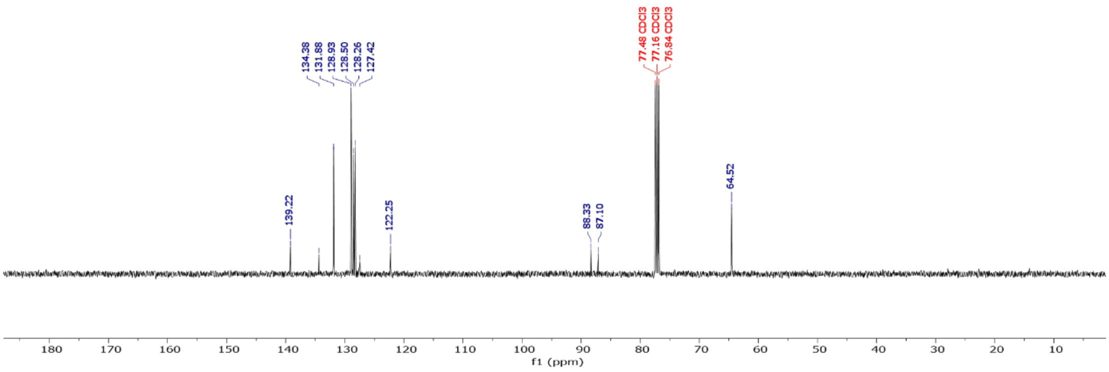

Figure S83 – ^{13}C NMR (101 MHz, CDCl$_3$, 298 K) spectrum of **1r**.

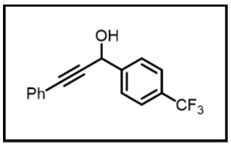

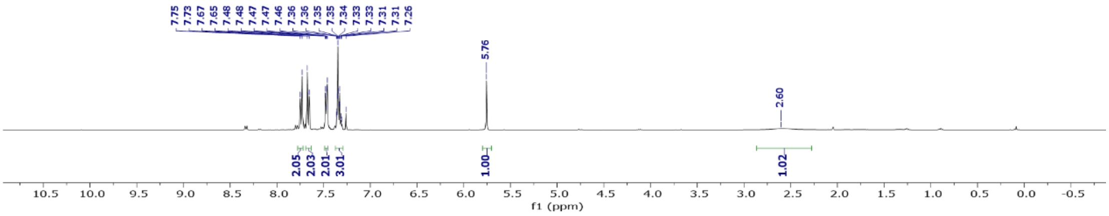

Figure S84 – ^{1}H NMR (400 MHz, CDCl$_3$, 298 K) spectrum of **1s**.

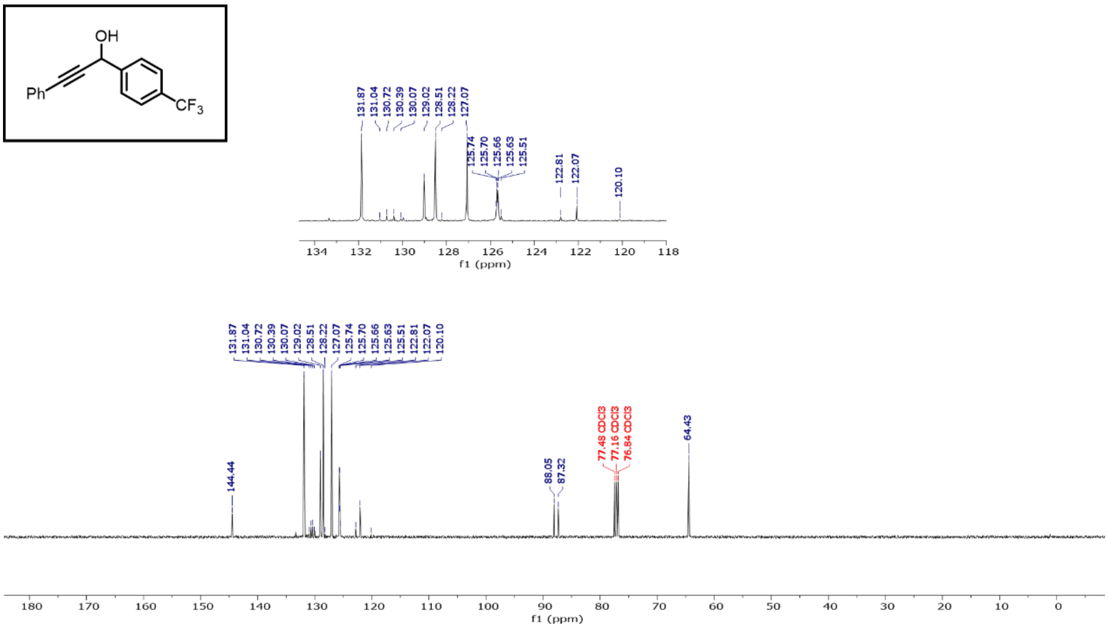

Figure S85 – ^{13}C NMR (101 MHz, $CDCl_3$, 298 K) spectrum of **1s**.

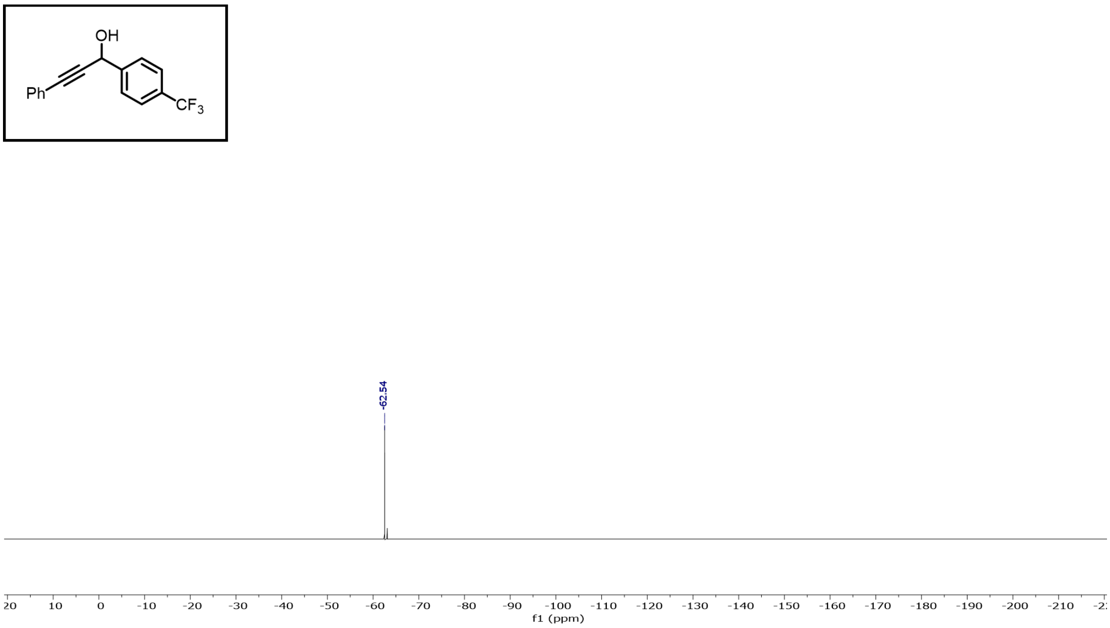

Figure S86 – ^{19}F NMR (376 MHz, $CDCl_3$, 298 K) spectrum of the reaction from **1s**.

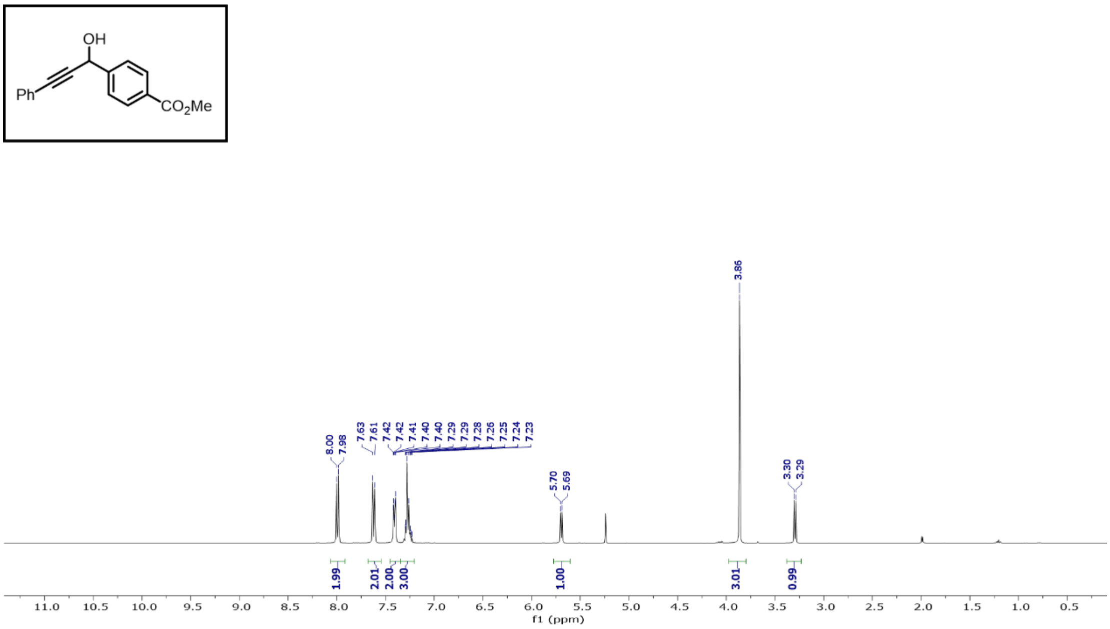

Figure S87 – ^{1}H NMR (400 MHz, CDCl$_3$, 298 K) spectrum of **1t**.

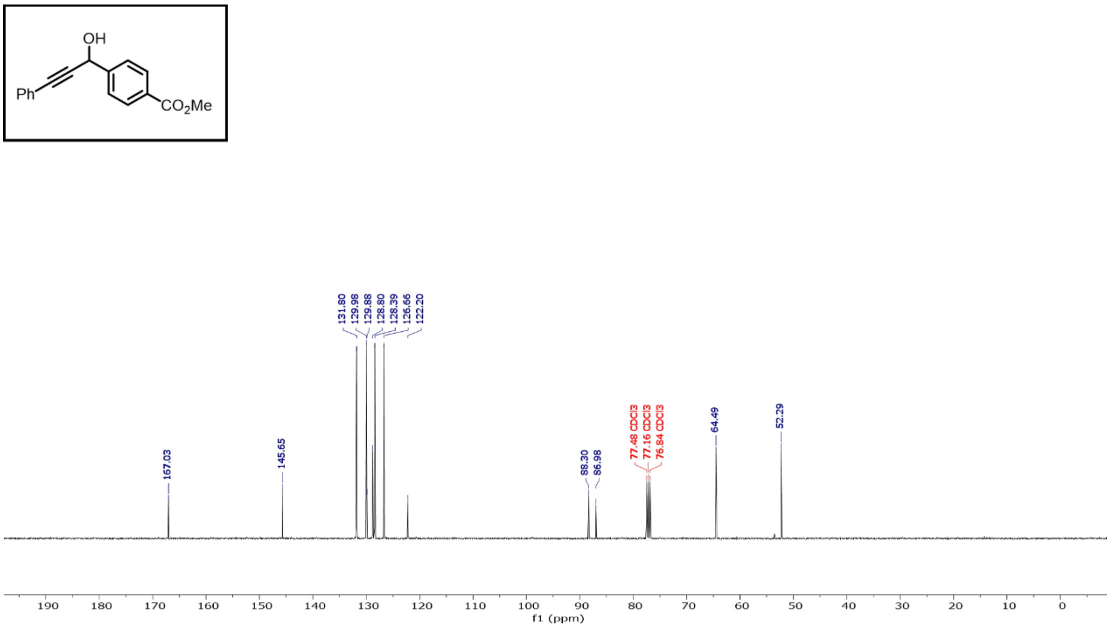

Figure S88 – ^{13}C NMR (101 MHz, CDCl$_3$, 298 K) spectrum of **1t**.

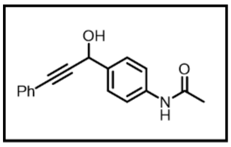

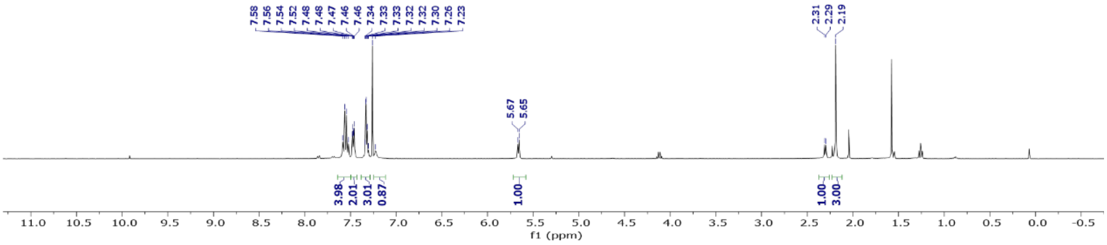

Figure S89 – ^{1}H NMR (400 MHz, CDCl$_3$, 298 K) spectrum of **1u**.

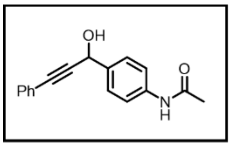

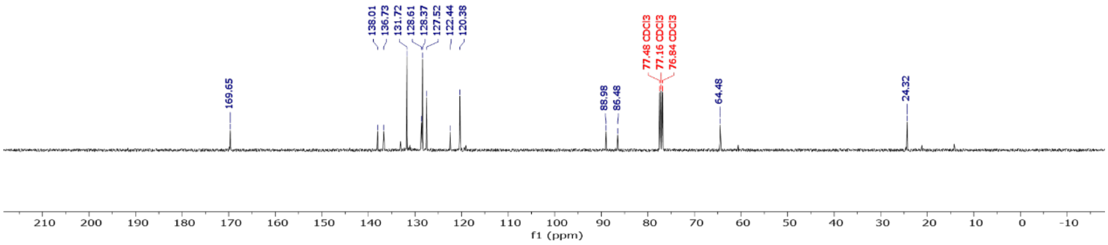

Figure S90 – ^{13}C NMR (101 MHz, CDCl$_3$, 298 K) spectrum of **1u**.

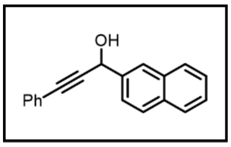

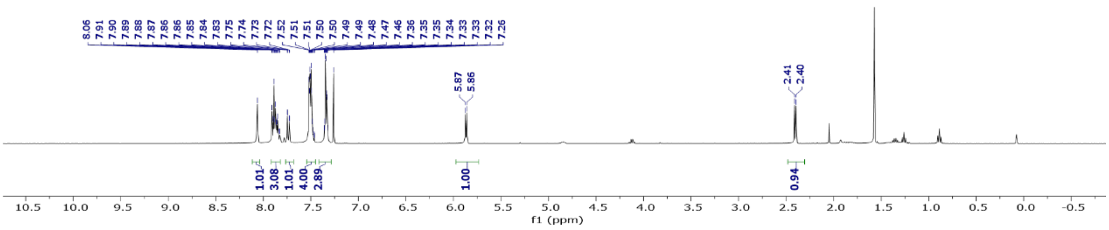

Figure S91 – ^{1}H NMR (400 MHz, CDCl$_3$, 298 K) spectrum of **1v**.

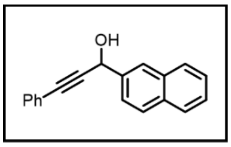

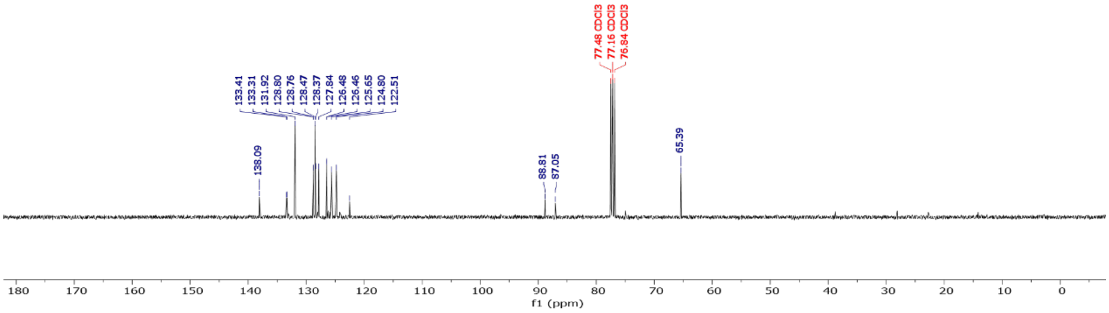

Figure S92 – ^{13}C NMR (101 MHz, CDCl$_3$, 298 K) spectrum of **1v**.

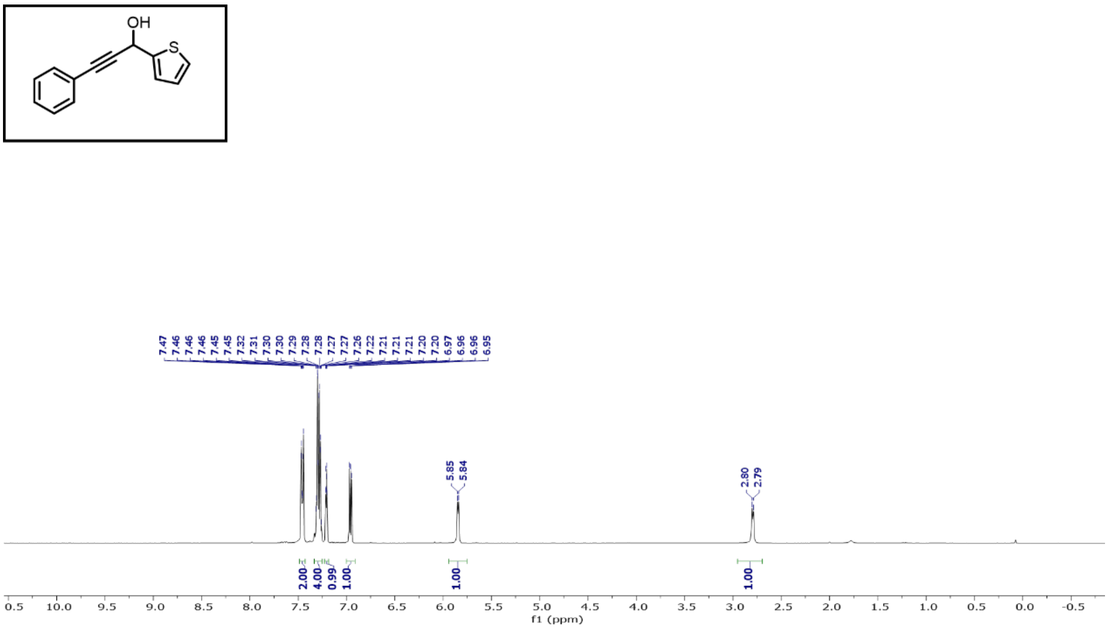

Figure S93 – ^{1}H NMR (400 MHz, CDCl$_3$, 298 K) spectrum of **1w**.

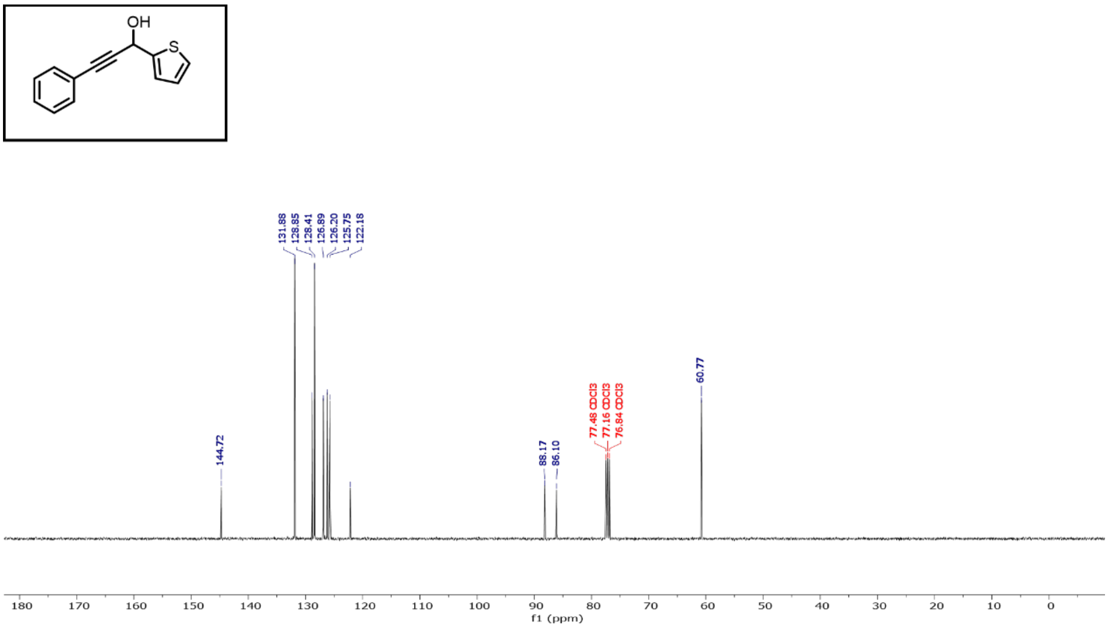

Figure S94 – ^{13}C NMR (101 MHz, CDCl$_3$, 298 K) spectrum of **1w**.

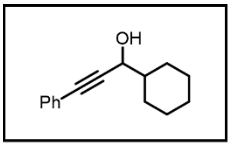

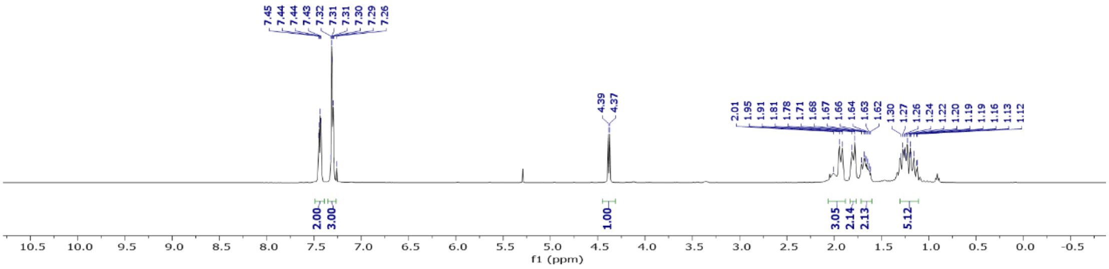

Figure S95 – ^{1}H NMR (400 MHz, $CDCl_3$, 298 K) spectrum of **1x**.

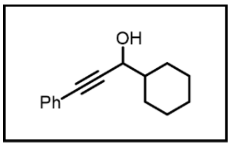

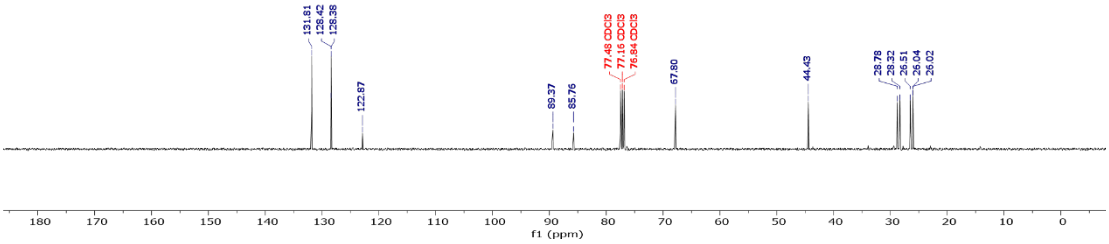

Figure S96 – ^{13}C NMR (101 MHz, $CDCl_3$, 298 K) spectrum of **1x**.

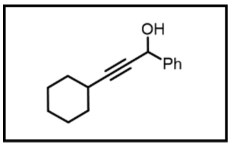

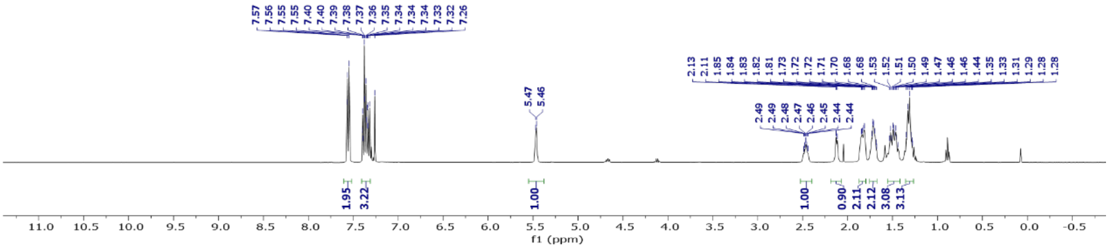

Figure S97 – ^{1}H NMR (400 MHz, $CDCl_3$, 298 K) spectrum of **1y**.

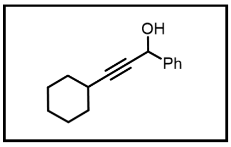

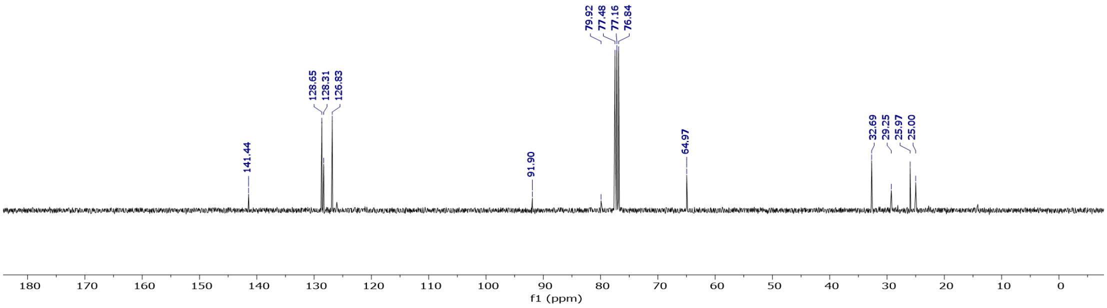

Figure S98 – ^{13}C NMR (101 MHz, $CDCl_3$, 298 K) spectrum of **1y**.

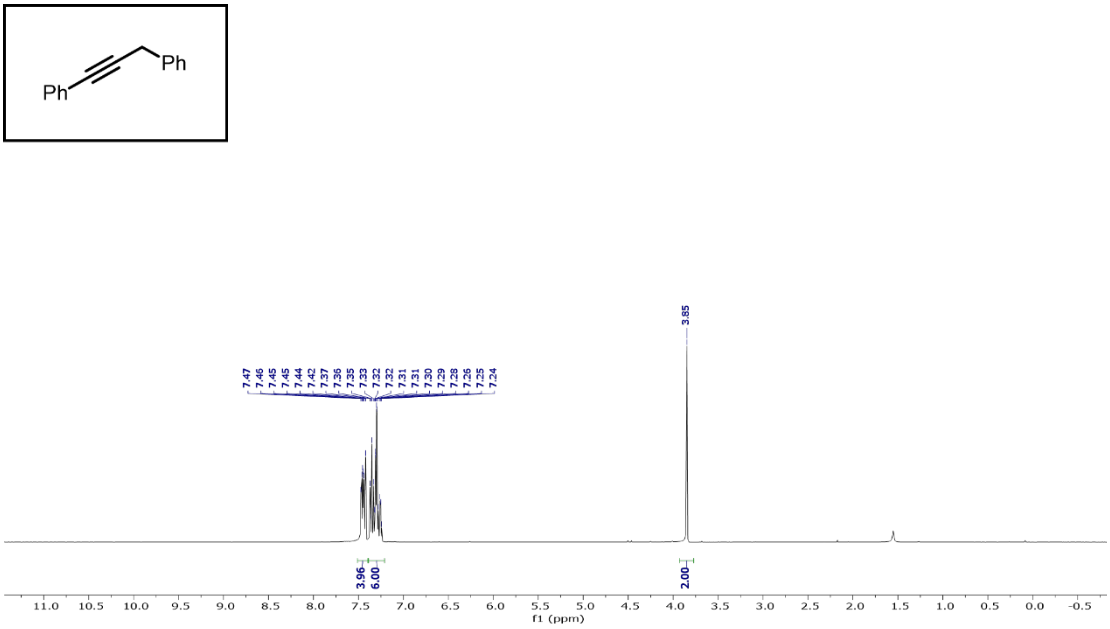

Figure S99 – ^{1}H NMR (400 MHz, $CDCl_3$, 298 K) spectrum of **2a**.

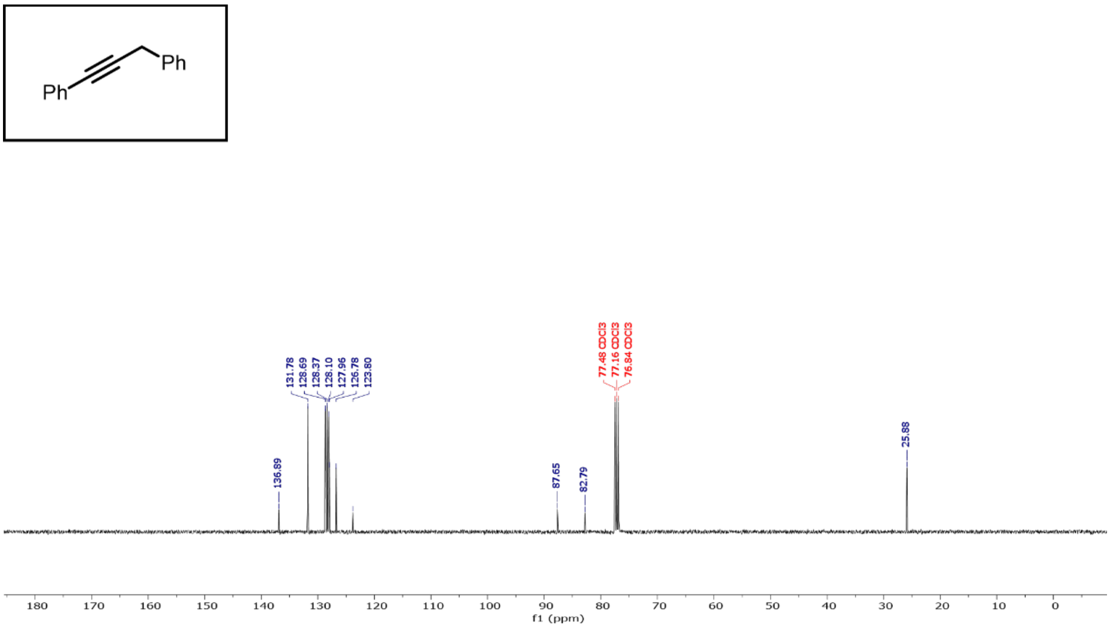

Figure S100 – ^{13}C NMR (101 MHz, $CDCl_3$, 298 K) spectrum of **2a**.

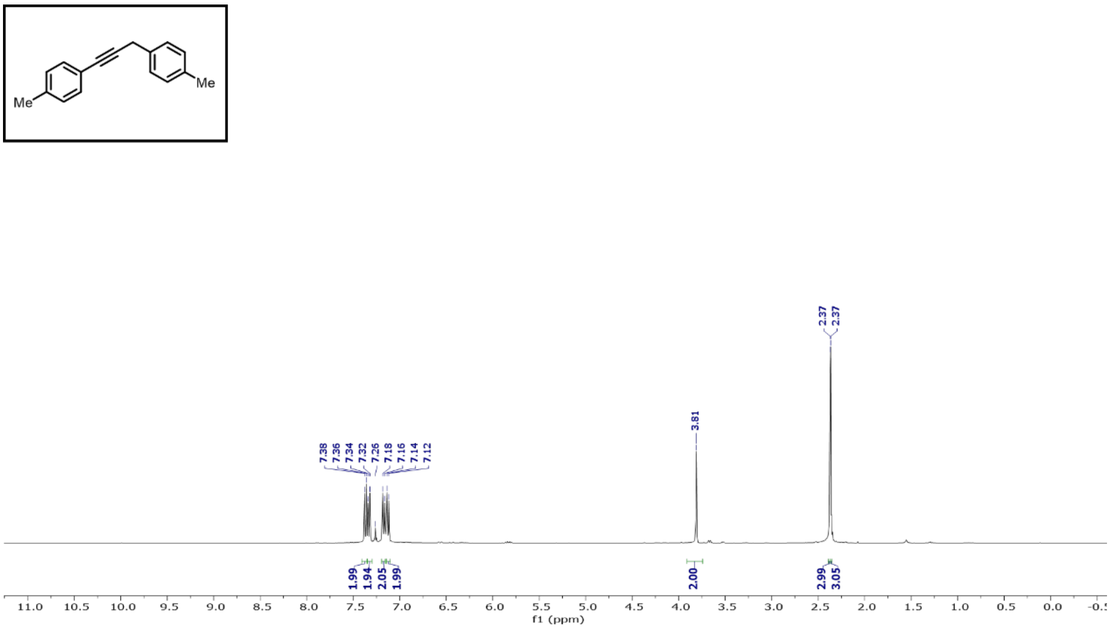

Figure S101 – ^{1}H NMR (400 MHz, $CDCl_3$, 298 K) spectrum of **2b**.

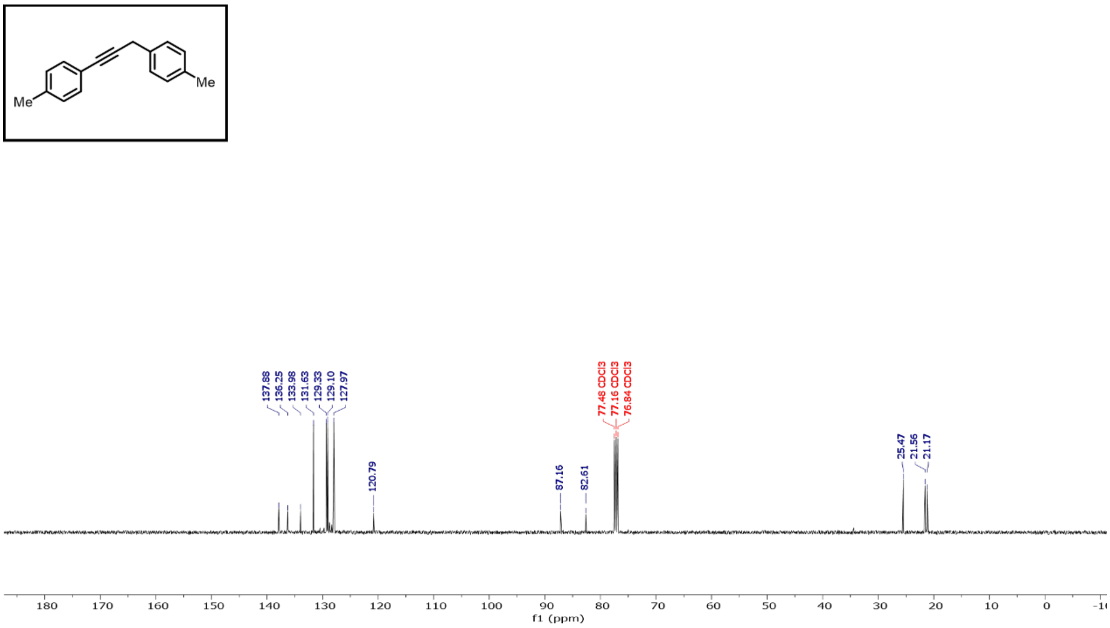

Figure S102 – ^{13}C NMR (101 MHz, $CDCl_3$, 298 K) spectrum of **2b**.

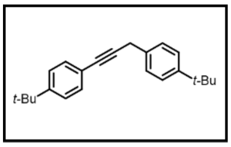

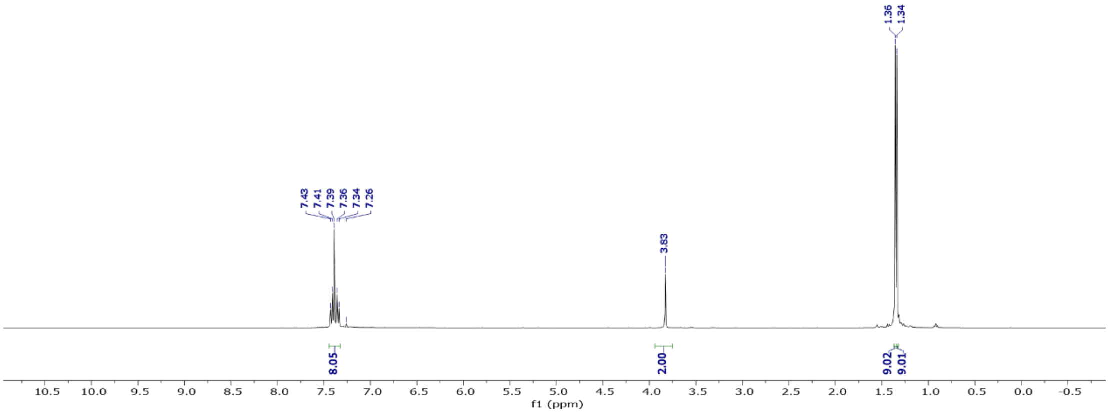

Figure S103 – ^{1}H NMR (400 MHz, $CDCl_3$, 298 K) spectrum of **2c**.

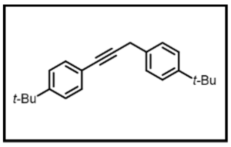

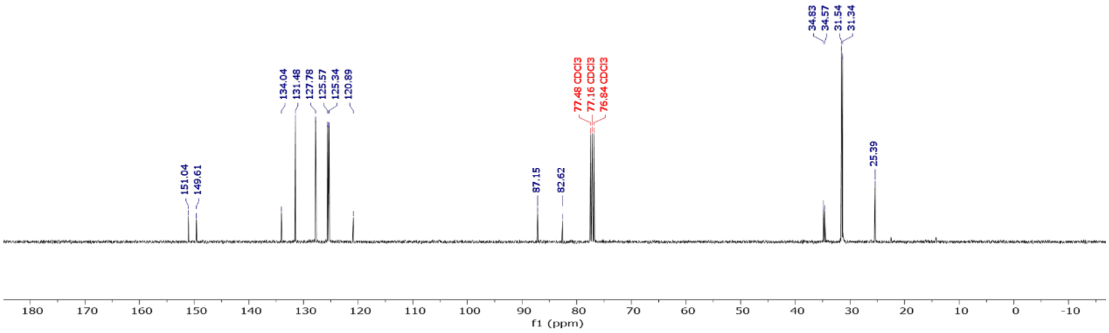

Figure S104 – ^{13}C NMR (101 MHz, $CDCl_3$, 298 K) spectrum of **2c**.

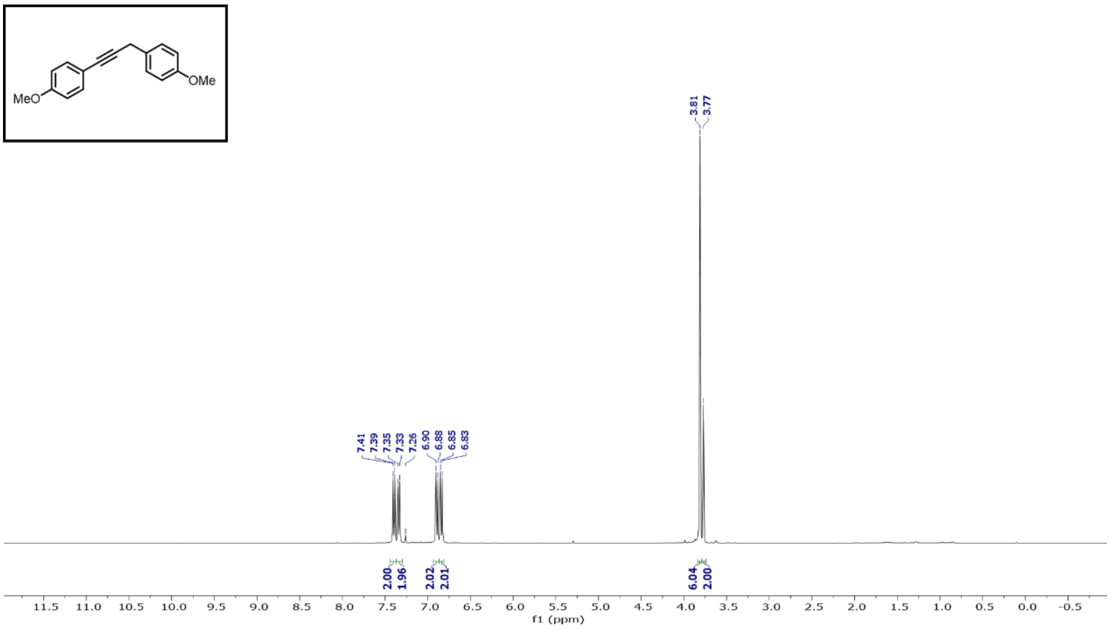

Figure S105 – ^{1}H NMR (400 MHz, CDCl$_3$, 298 K) spectrum of **2d**.

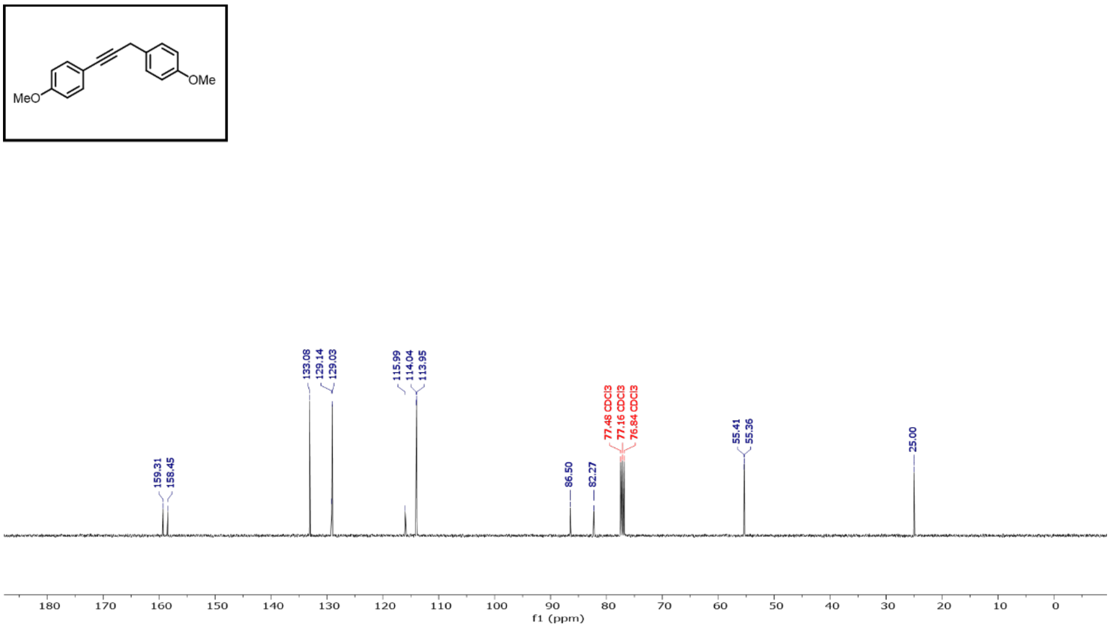

Figure S106 – ^{13}C NMR (101 MHz, CDCl$_3$, 298 K) spectrum of **2d**.

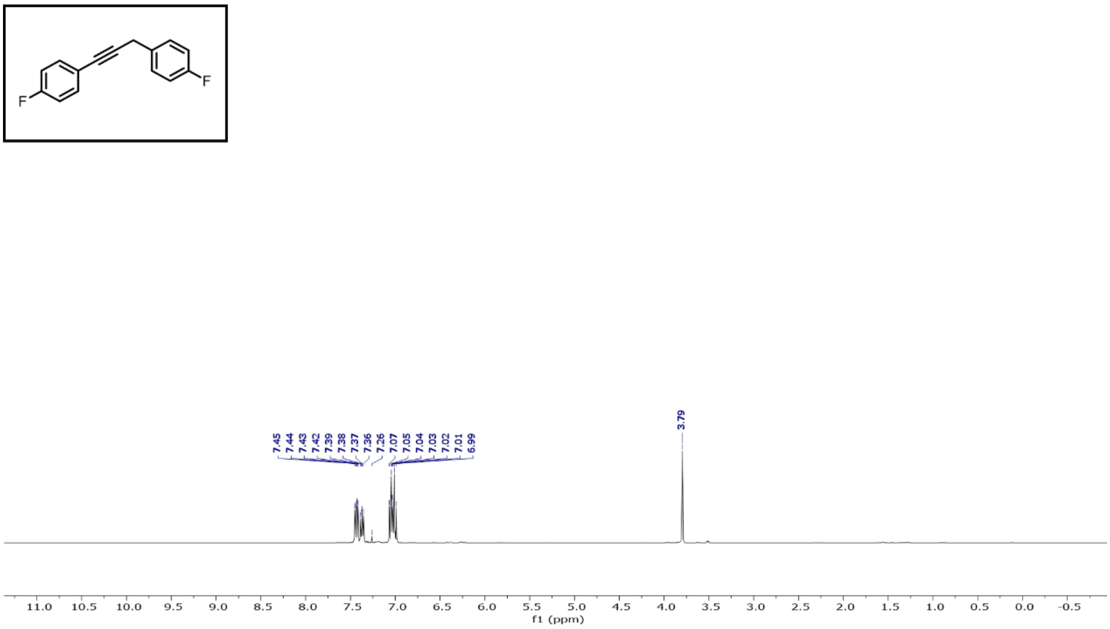

Figure S107 – ^{1}H NMR (400 MHz, $CDCl_3$, 298 K) spectrum of **2e**.

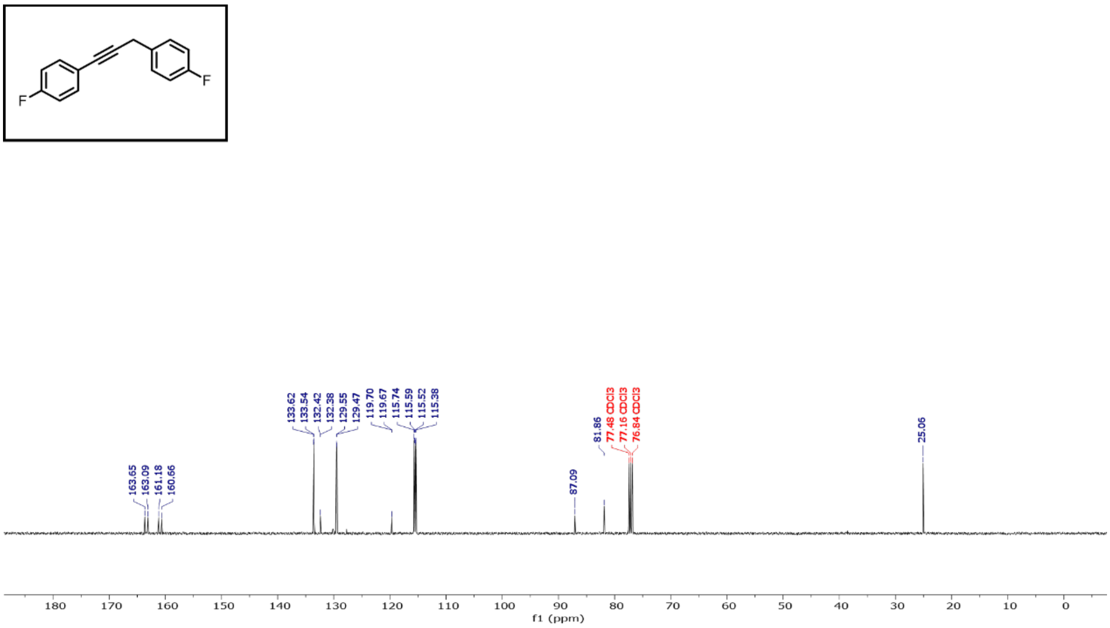

Figure S108 – ^{13}C NMR (101 MHz, $CDCl_3$, 298 K) spectrum of **2e**.

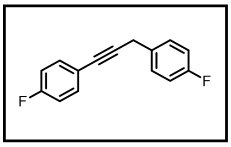

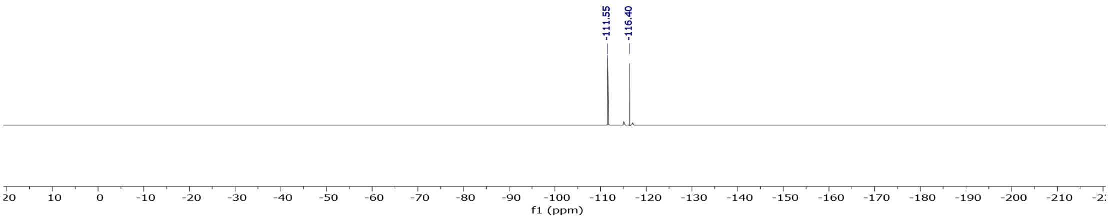

Figure S109 – ^{19}F NMR (376 MHz, CDCl$_3$, 298 K) spectrum of the reaction from **2e**.

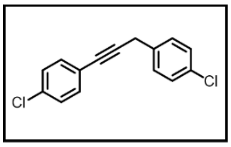

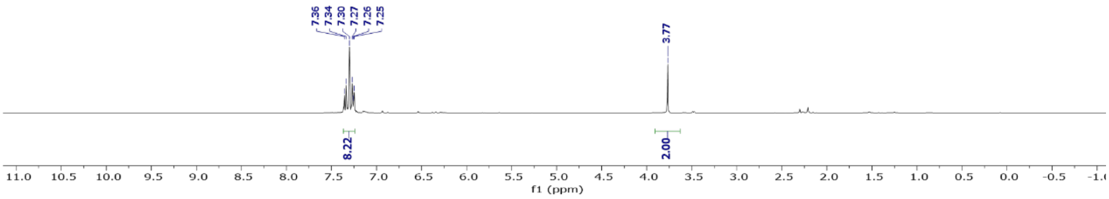

Figure S110 – ^{1}H NMR (400 MHz, CDCl$_3$, 298 K) spectrum of **2h**.

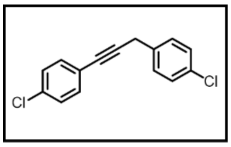

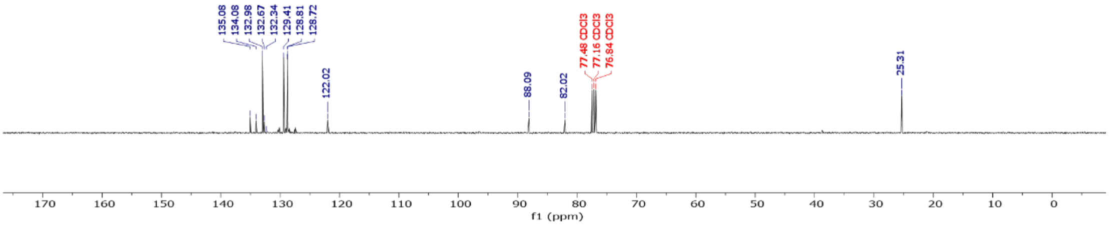

Figure S111 – ^{13}C NMR (101 MHz, $CDCl_3$, 298 K) spectrum of **2h**.

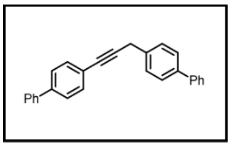

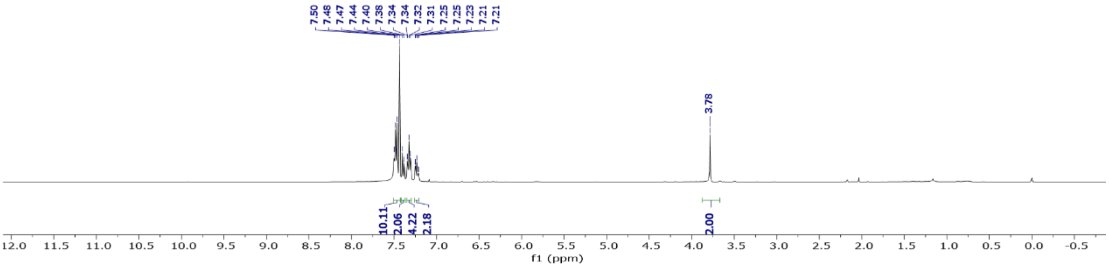

Figure S112 – ^{1}H NMR (400 MHz, $CDCl_3$, 298 K) spectrum of **2i**.

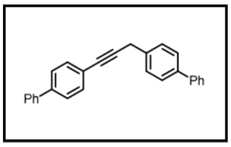

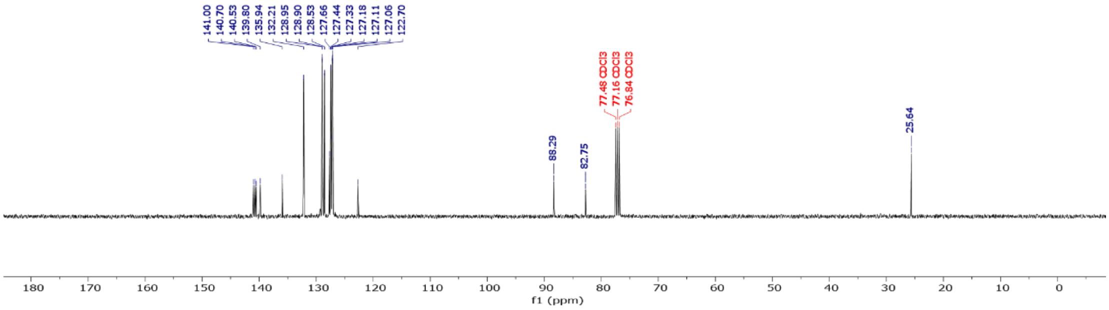

Figure S113 – ^{13}C NMR (101 MHz, $CDCl_3$, 298 K) spectrum of **2i**.

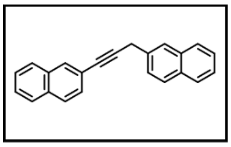

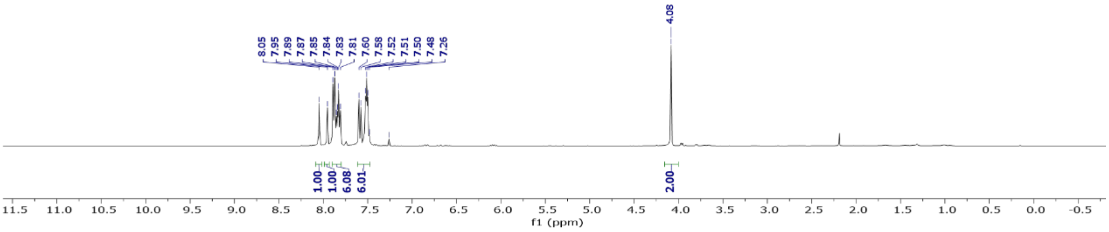

Figure S114 – ^{1}H NMR (400 MHz, $CDCl_3$, 298 K) spectrum of **2j**.

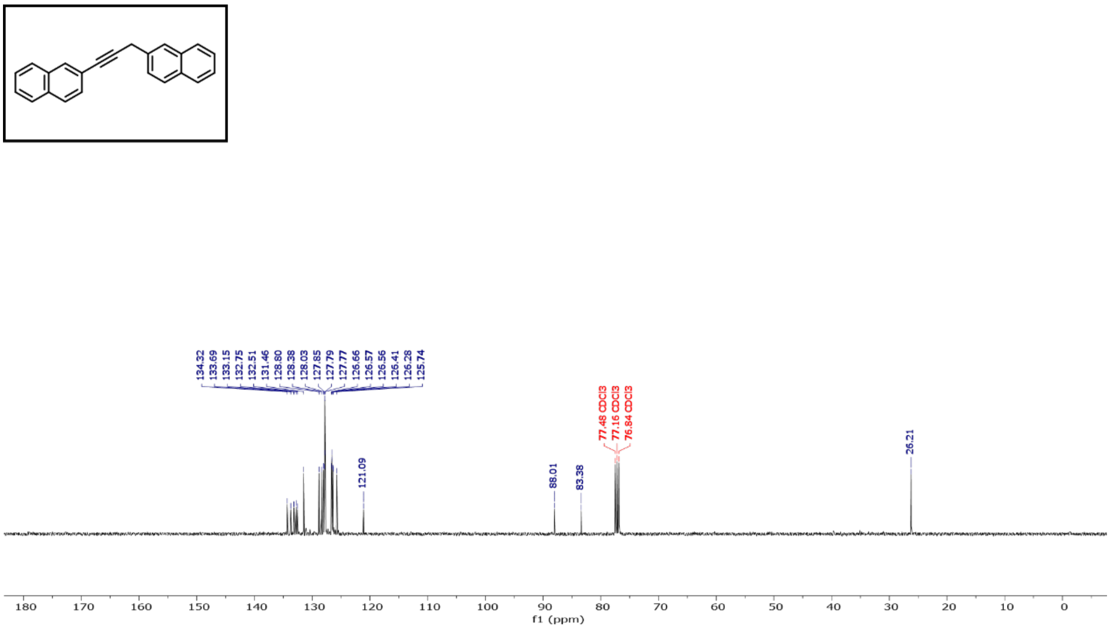

Figure S115 – ^{13}C NMR (101 MHz, CDCl$_3$, 298 K) spectrum of **2j**.

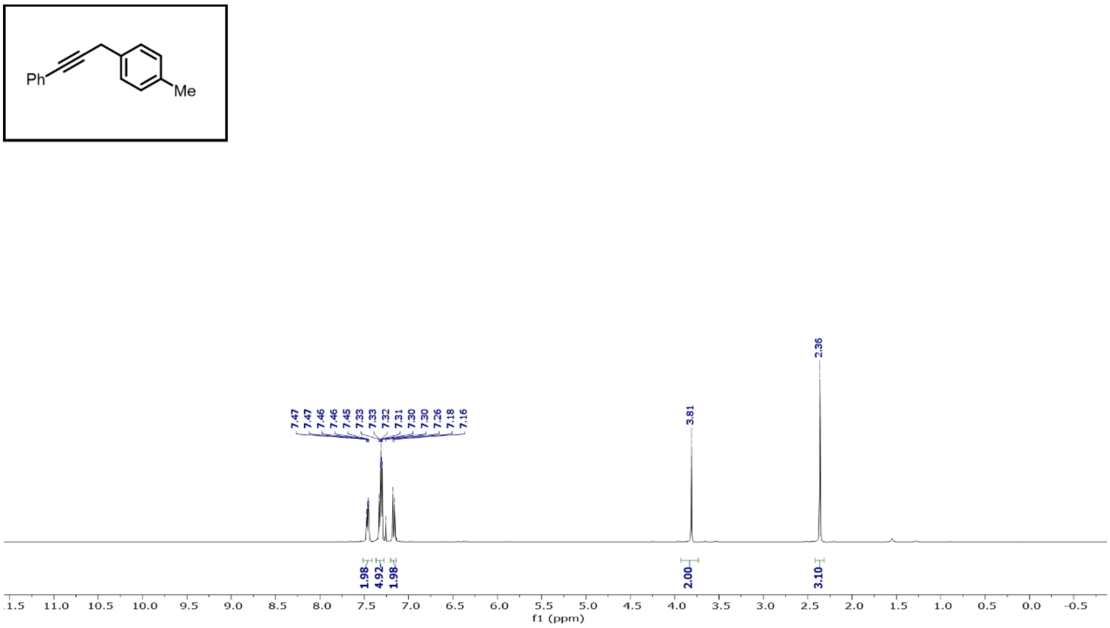

Figure S116 – ^{1}H NMR (400 MHz, CDCl$_3$, 298 K) spectrum of **2m**.

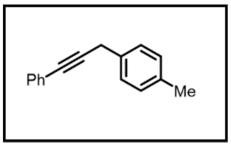

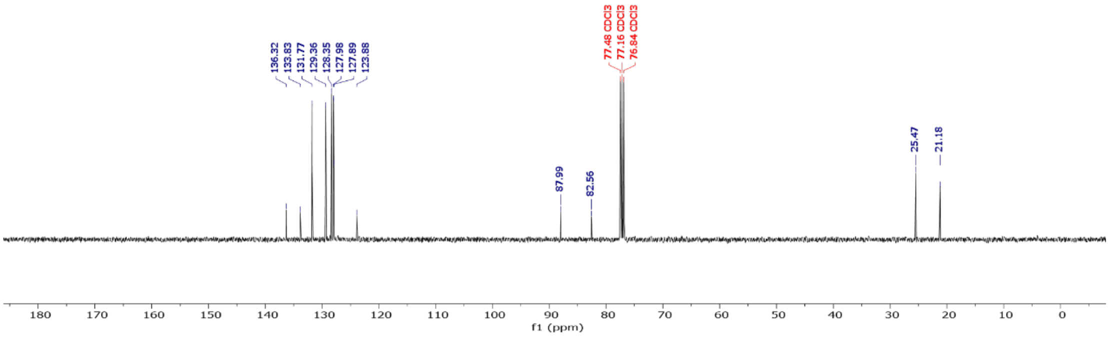

Figure S117 – ^{13}C NMR (101 MHz, $CDCl_3$, 298 K) spectrum of **2m**.

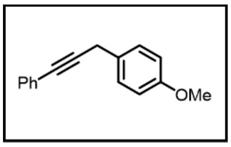

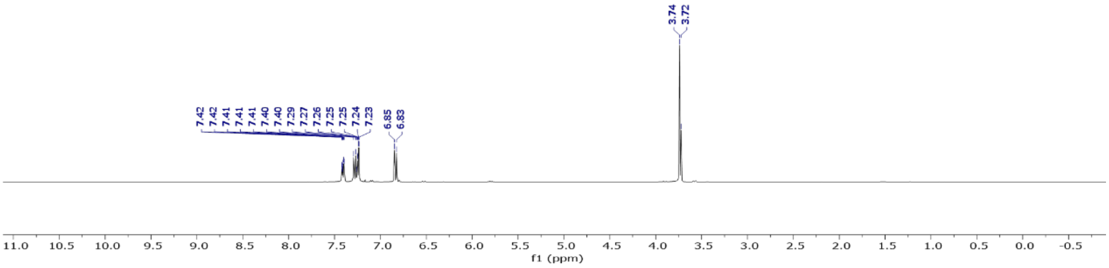

Figure S118 – ^{1}H NMR (400 MHz, $CDCl_3$, 298 K) spectrum of **2n**.

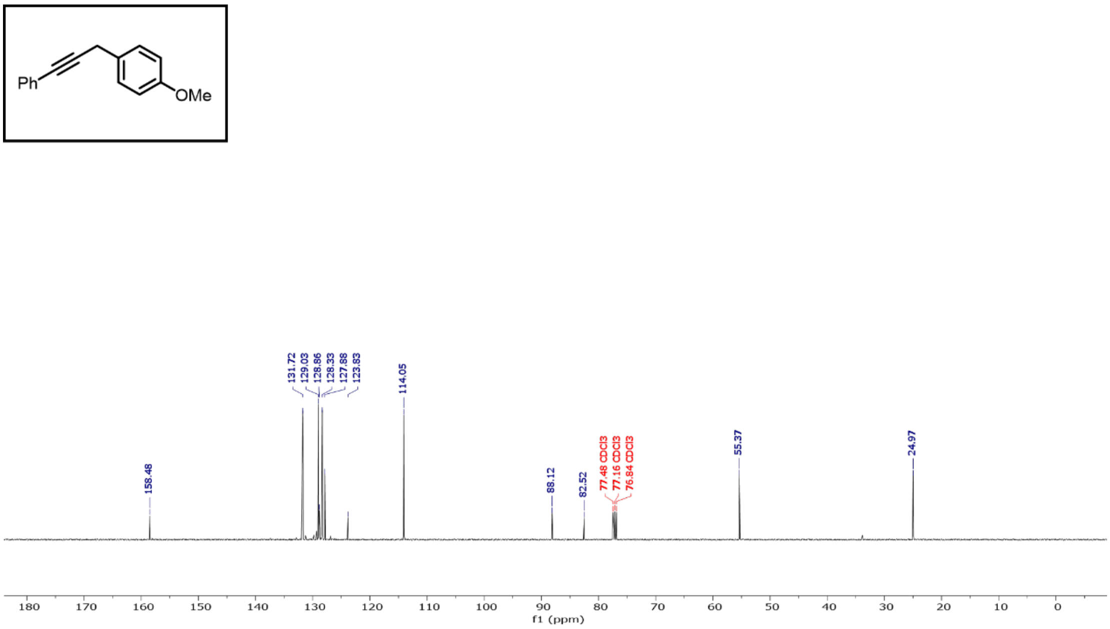

Figure S119 – ^{13}C NMR (101 MHz, CDCl$_3$, 298 K) spectrum of **2n**.

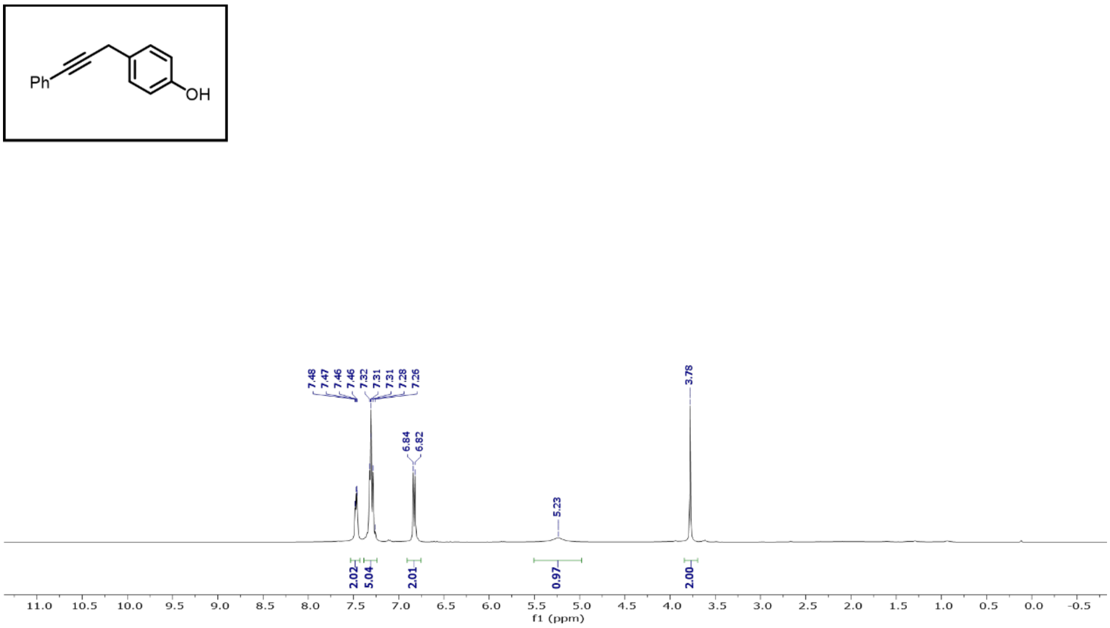

Figure S120 – ^{1}H NMR (400 MHz, CDCl$_3$, 298 K) spectrum of **2o**.

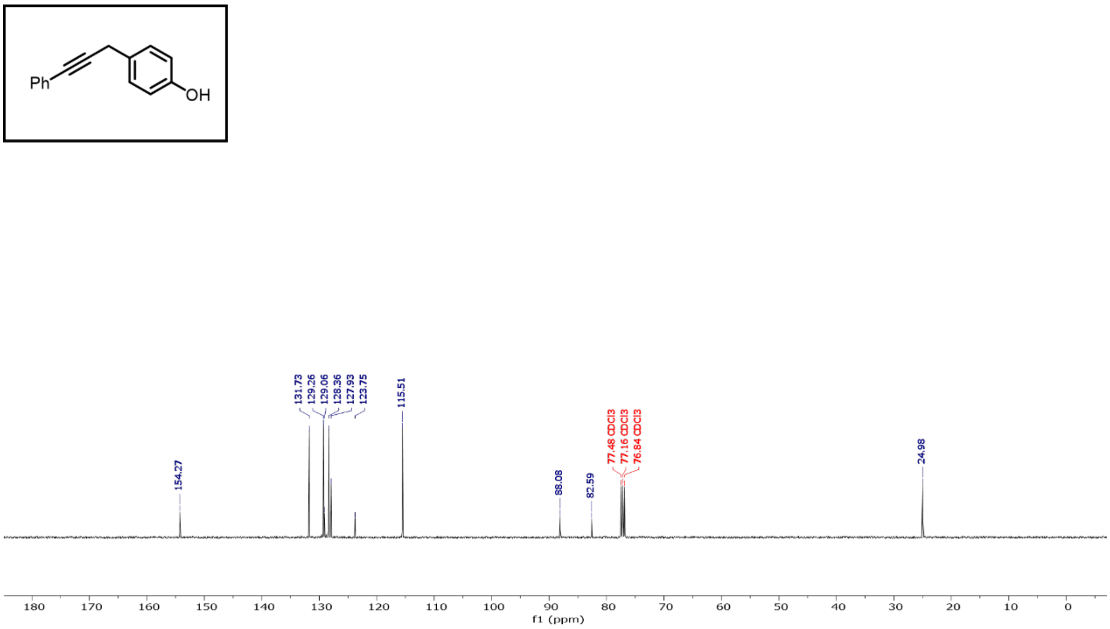

Figure S121 – ^{13}C NMR (101 MHz, $CDCl_3$, 298 K) spectrum of **2o**.

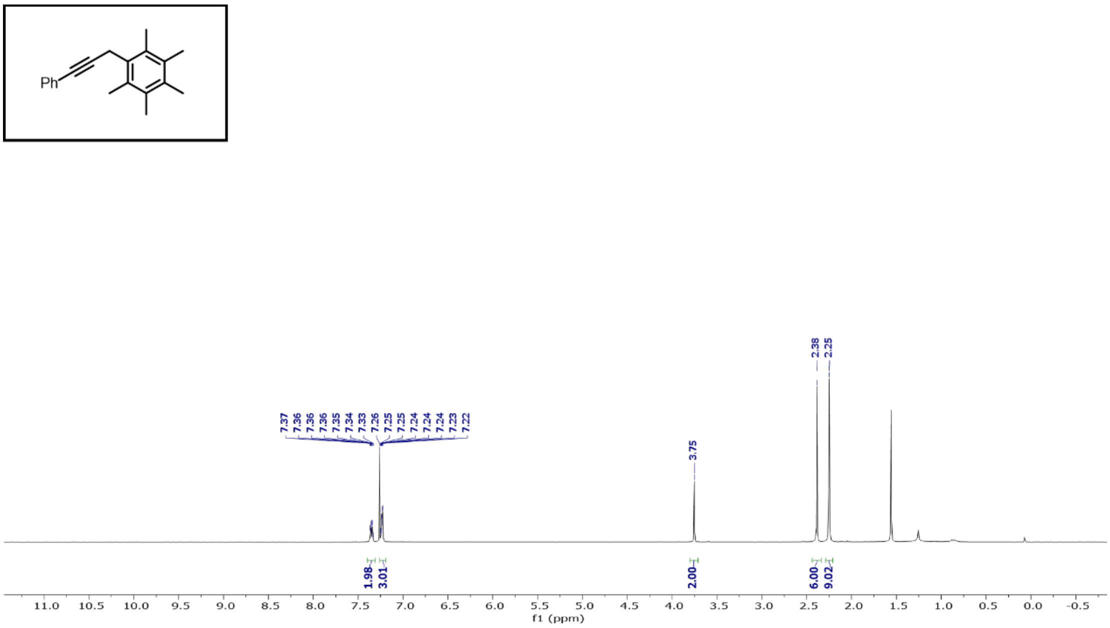

Figure S122 – ^{1}H NMR (400 MHz, $CDCl_3$, 298 K) spectrum of **2p**.

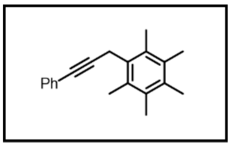

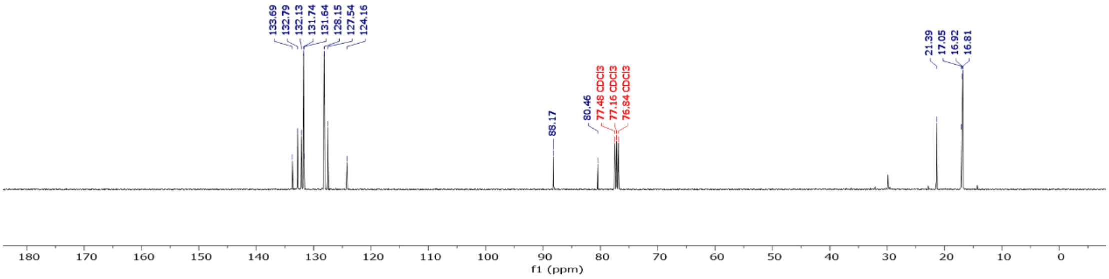

Figure S123 – ^{13}C NMR (101 MHz, CDCl$_3$, 298 K) spectrum of **2p**.

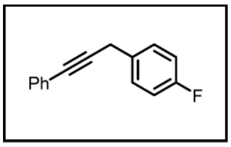

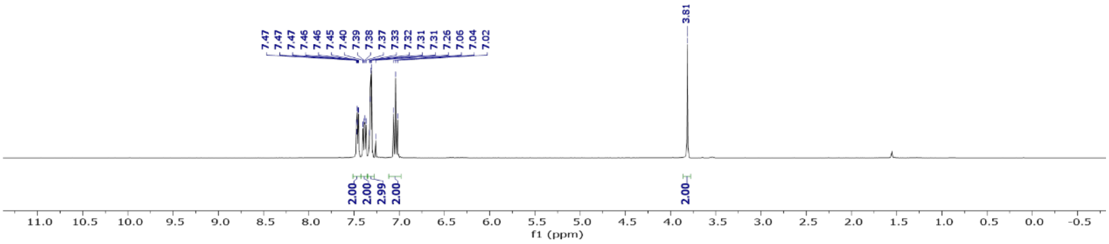

Figure S124 – ^{1}H NMR (400 MHz, CDCl$_3$, 298 K) spectrum of **2q**.

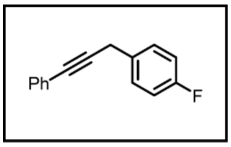

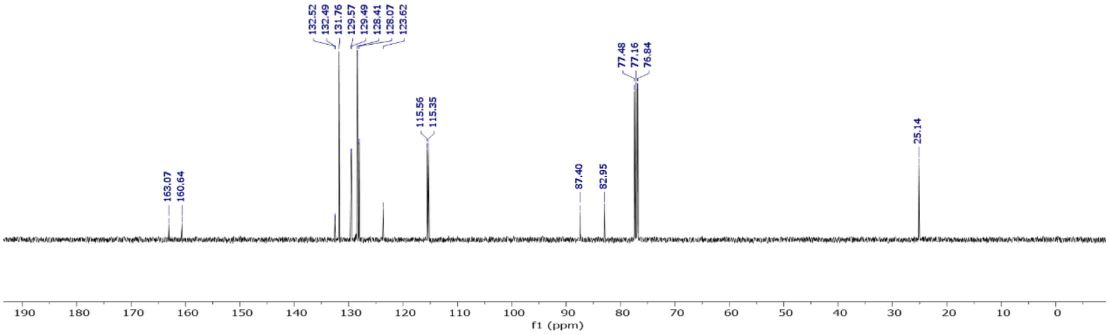

Figure S125 – ^{13}C NMR (101 MHz, CDCl$_3$, 298 K) spectrum of **2q**.

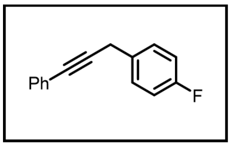

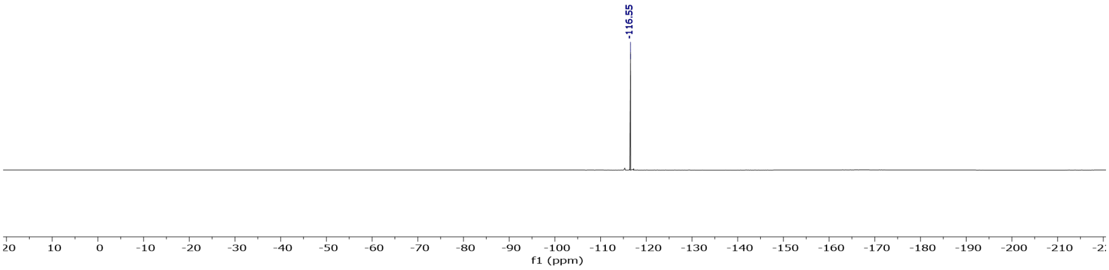

Figure S126 – ^{19}F NMR (376 MHz, CDCl$_3$, 298 K) spectrum of the reaction from **2q**.

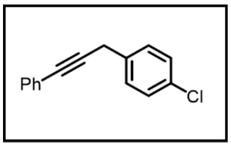

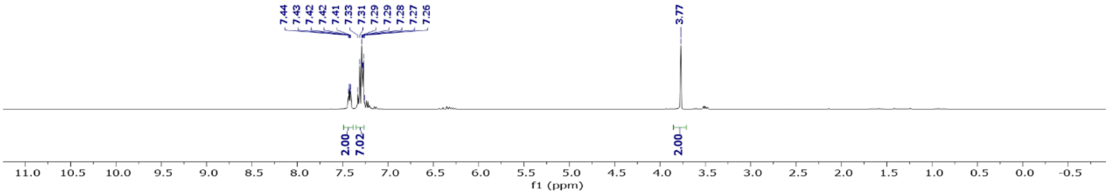

Figure S127 – ^{1}H NMR (400 MHz, CDCl$_3$, 298 K) spectrum of **2r**.

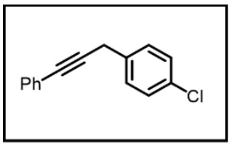

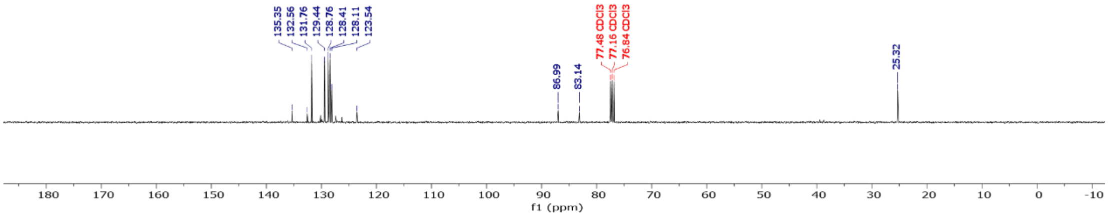

Figure S128 – ^{13}C NMR (101 MHz, CDCl$_3$, 298 K) spectrum of **2r**.

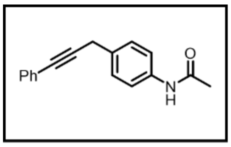

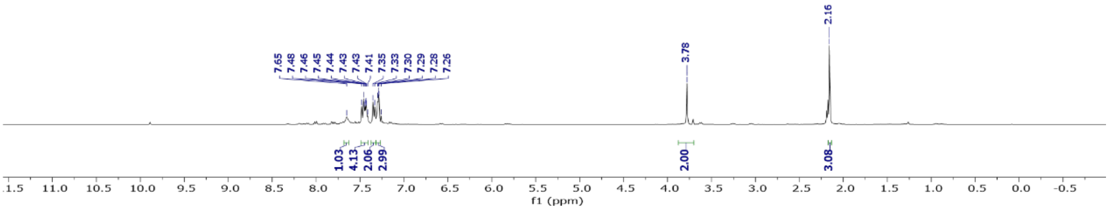

Figure S129 – ^{1}H NMR (400 MHz, $CDCl_3$, 298 K) spectrum of **2u**.

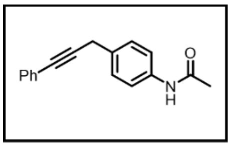

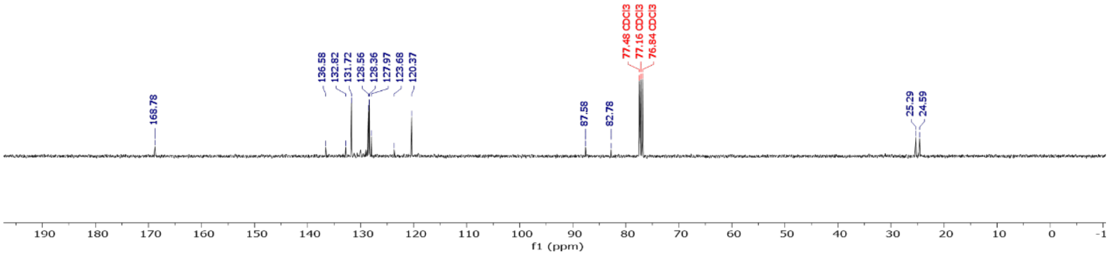

Figure S130 – ^{13}C NMR (101 MHz, $CDCl_3$, 298 K) spectrum of **2u**.

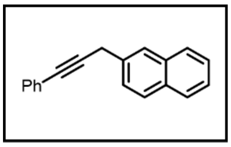

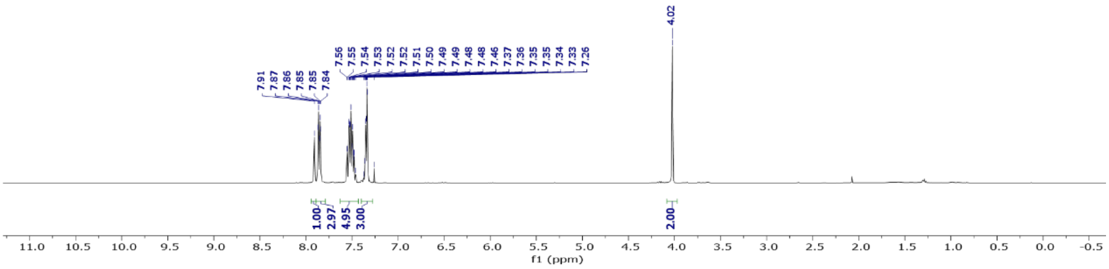

Figure S131 – ^{1}H NMR (400 MHz, $CDCl_3$, 298 K) spectrum of **2v**.

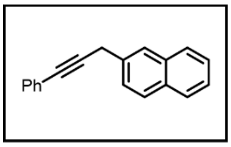

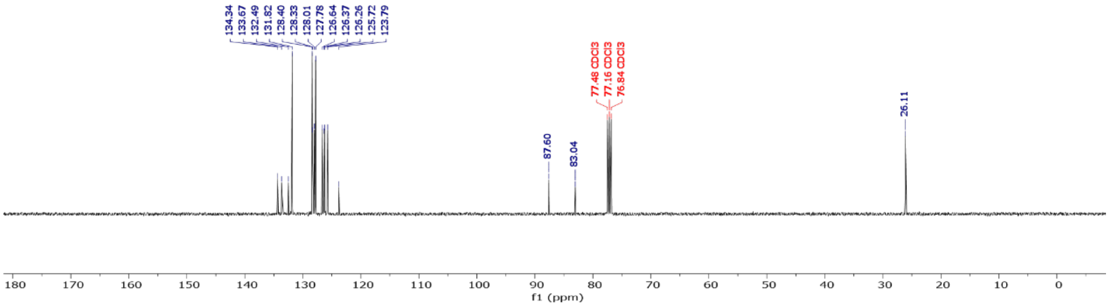

Figure S132 – ^{13}C NMR (101 MHz, $CDCl_3$, 298 K) spectrum of **2v**.

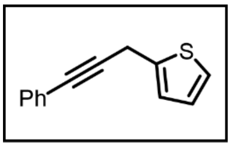

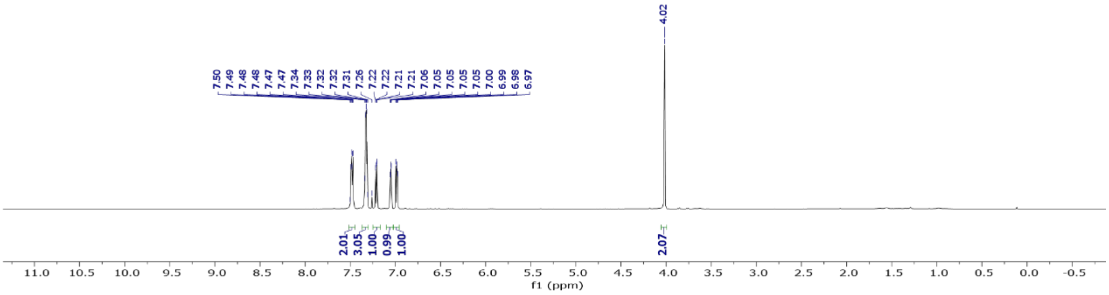

Figure S133 – ^{1}H NMR (400 MHz, $CDCl_3$, 298 K) spectrum of **2w**.

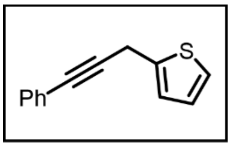

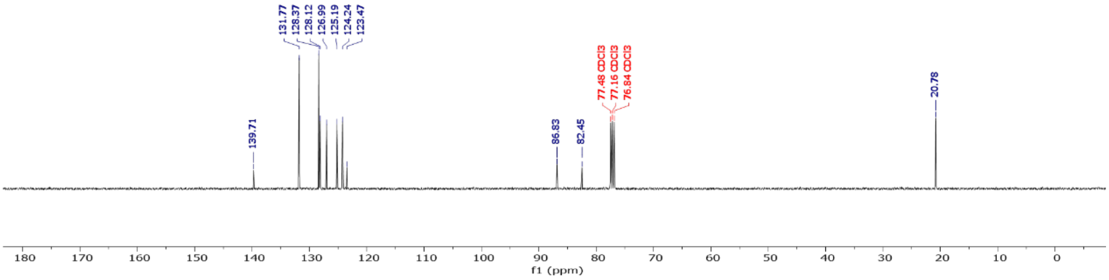

Figure S134 – ^{13}C NMR (101 MHz, $CDCl_3$, 298 K) spectrum of **2w**.

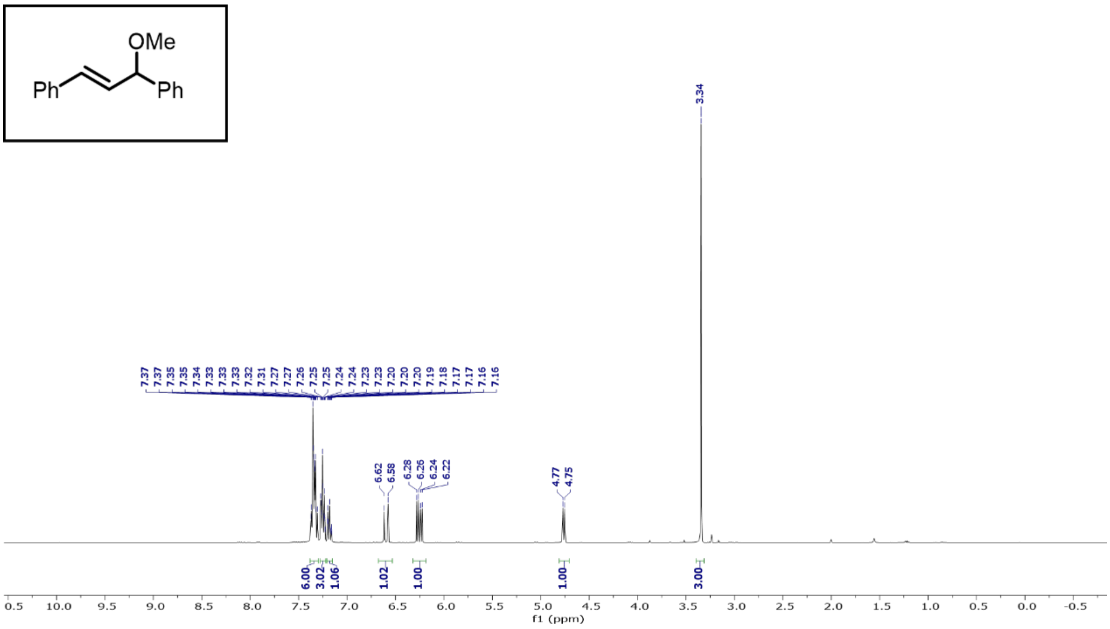

Figure S135 – ^{1}H NMR (400 MHz, $CDCl_3$, 298 K) spectrum of **3a**.

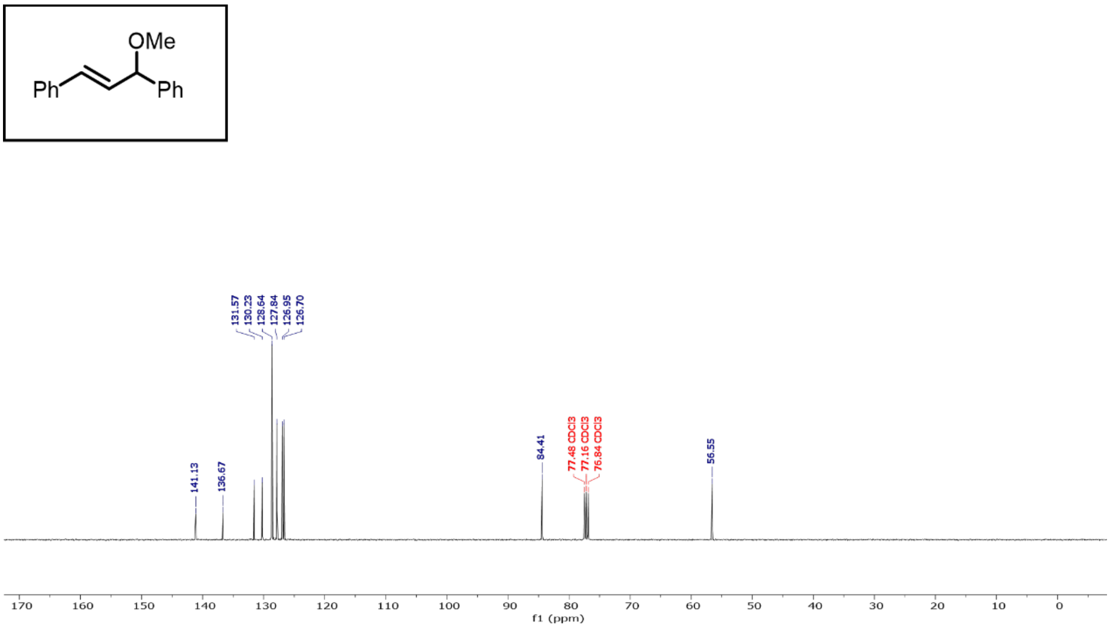

Figure S136 – ^{13}C NMR (101 MHz, $CDCl_3$, 298 K) spectrum of **3a**.

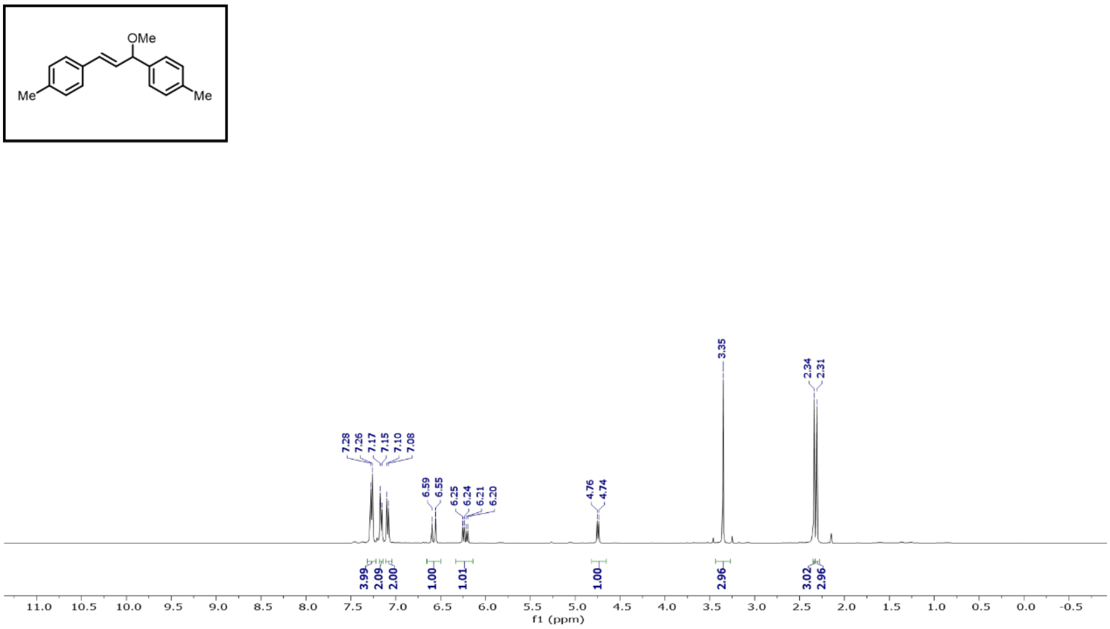

Figure S137 – ^{1}H NMR (400 MHz, $CDCl_3$, 298 K) spectrum of **3b**.

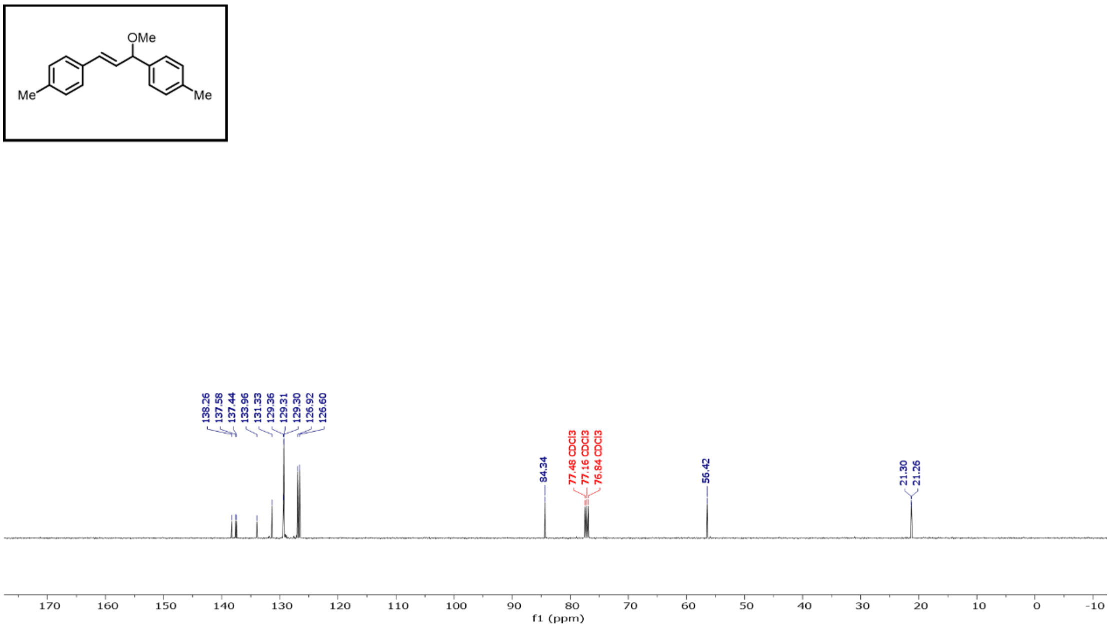

Figure S138 – ^{13}C NMR (101 MHz, $CDCl_3$, 298 K) spectrum of **3b**.

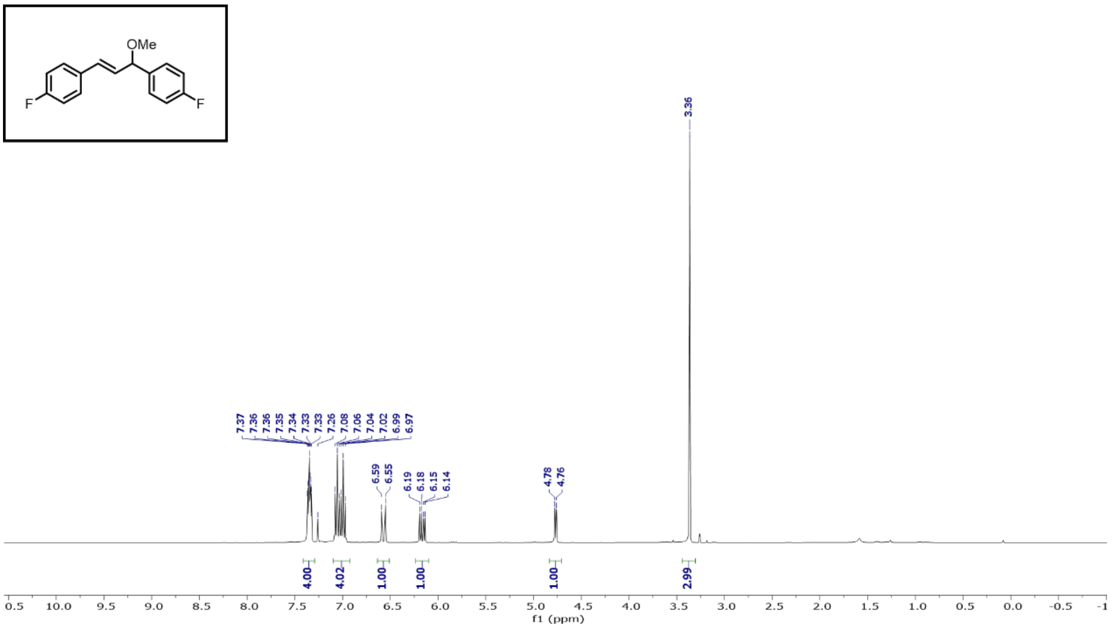

Figure S139 – ^{1}H NMR (400 MHz, $CDCl_3$, 298 K) spectrum of **3e**.

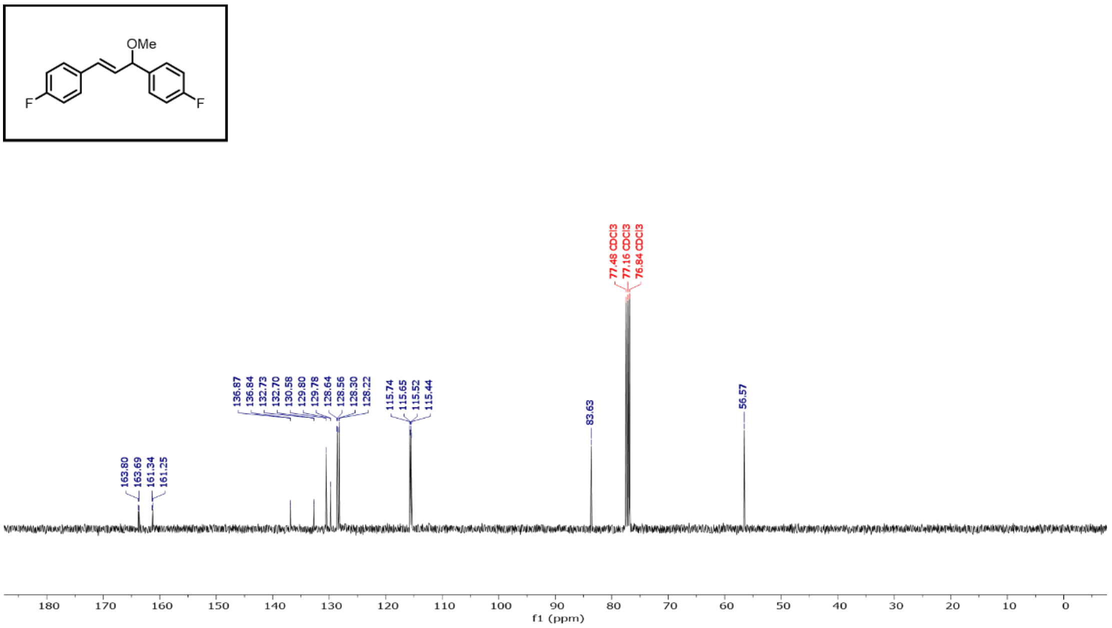

Figure S140 – ^{13}C NMR (101 MHz, $CDCl_3$, 298 K) spectrum of **3e**.

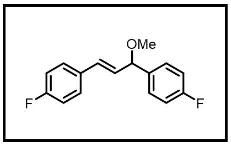

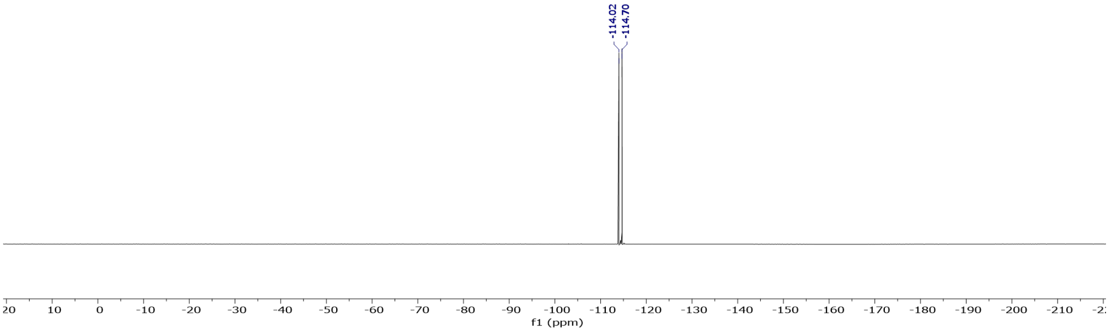

Figure S141 – ^{19}F NMR (376 MHz, CDCl$_3$, 298 K) spectrum of the reaction from **3e**.

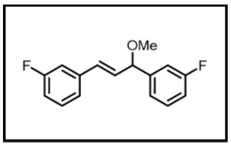

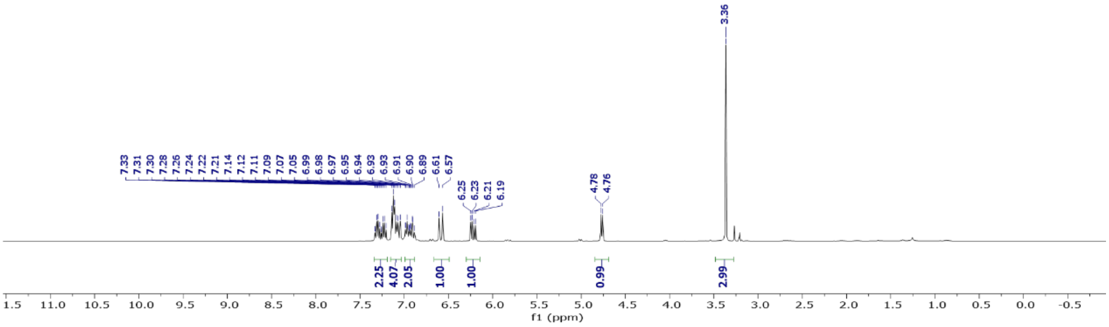

Figure S142 – ^{1}H NMR (400 MHz, CDCl$_3$, 298 K) spectrum of **3f**.

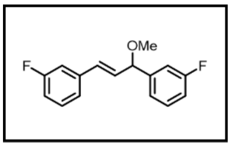

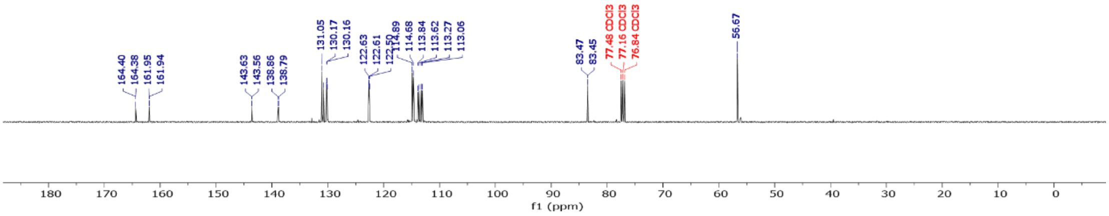

Figure S143 – ^{13}C NMR (101 MHz, $CDCl_3$, 298 K) spectrum of **3f**.

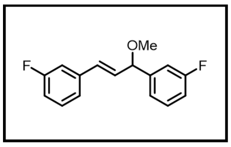

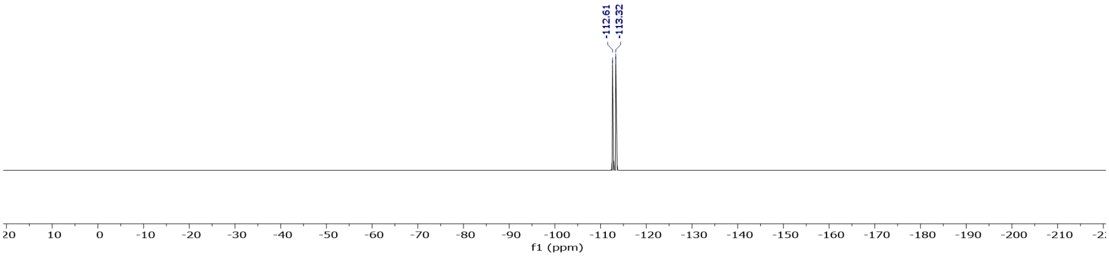

Figure S144 – ^{19}F NMR (376 MHz, $CDCl_3$, 298 K) spectrum of the reaction from **3f**.

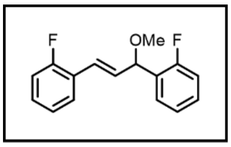

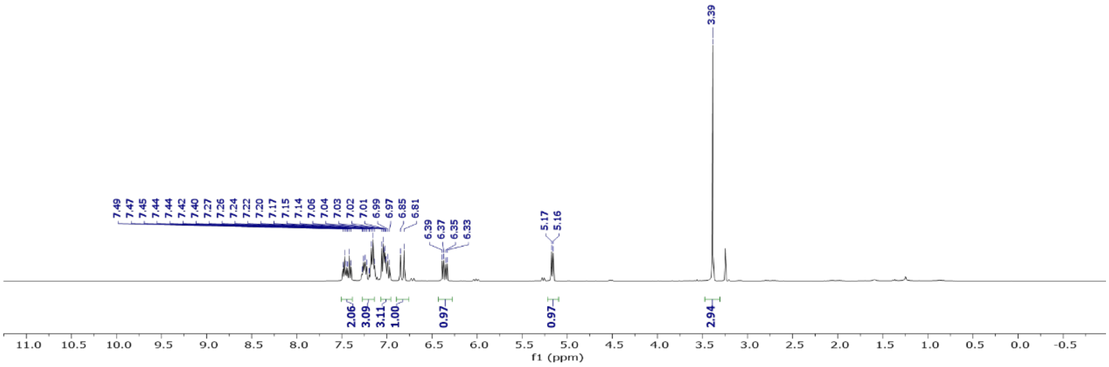

Figure S145 – ^{1}H NMR (400 MHz, CDCl$_3$, 298 K) spectrum of **3g**.

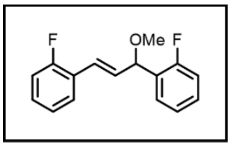

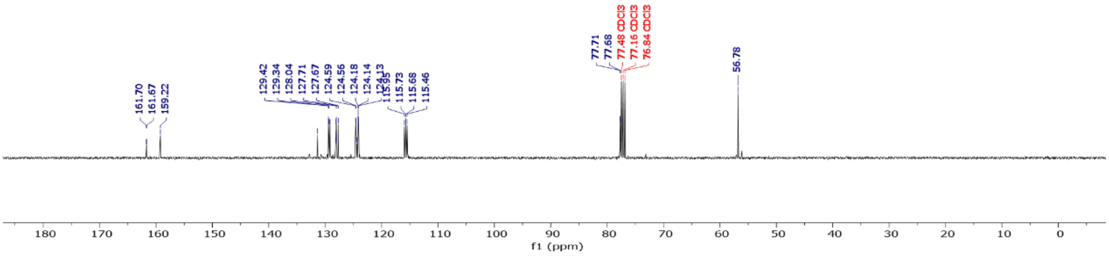

Figure S146 – ^{13}C NMR (101 MHz, CDCl$_3$, 298 K) spectrum of **3g**.

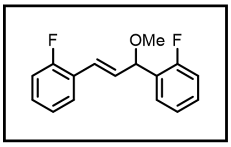

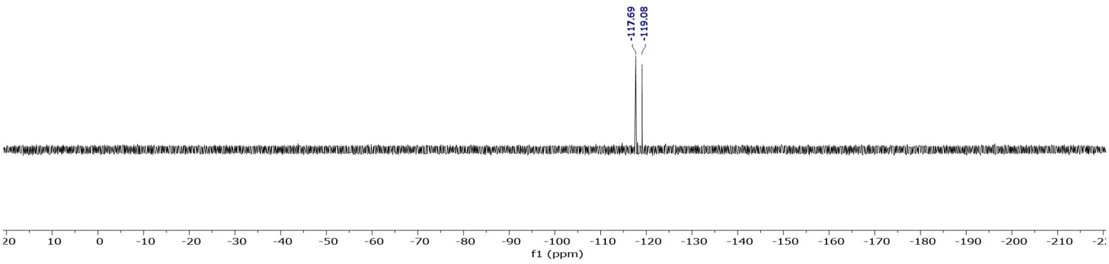

Figure S147 – ^{19}F NMR (376 MHz, CDCl$_3$, 298 K) spectrum of the reaction from **3g**.

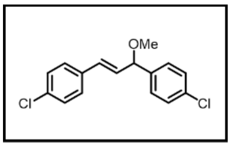

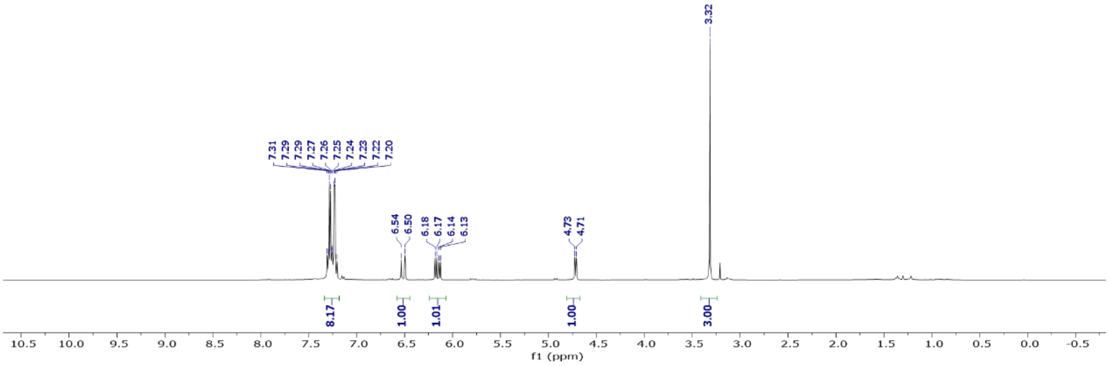

Figure S148 – ^{1}H NMR (400 MHz, CDCl$_3$, 298 K) spectrum of **3h**.

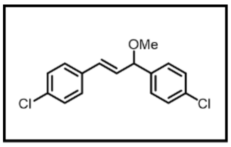

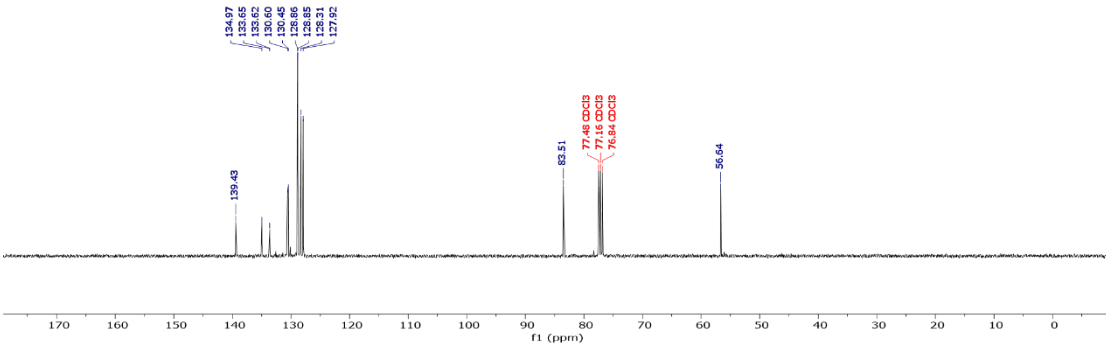

Figure S149 – ^{13}C NMR (101 MHz, $CDCl_3$, 298 K) spectrum of **3h**.

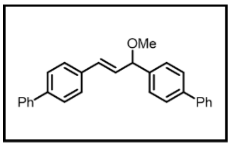

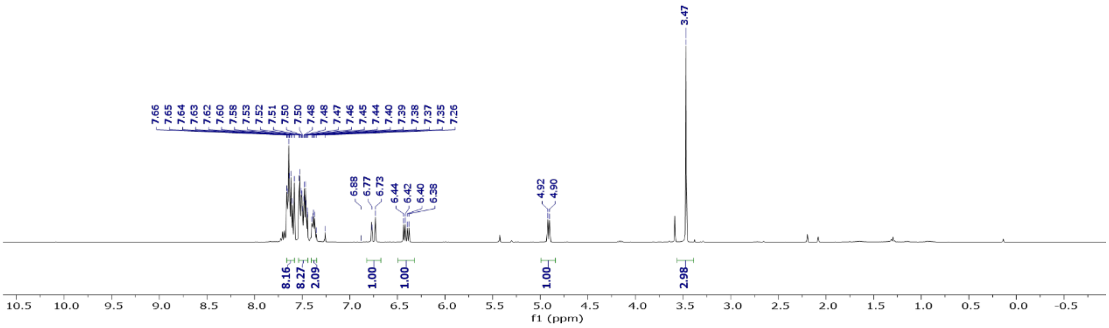

Figure S150 – ^{1}H NMR (400 MHz, $CDCl_3$, 298 K) spectrum of **3i**.

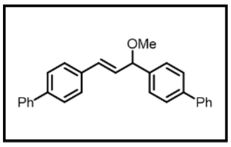

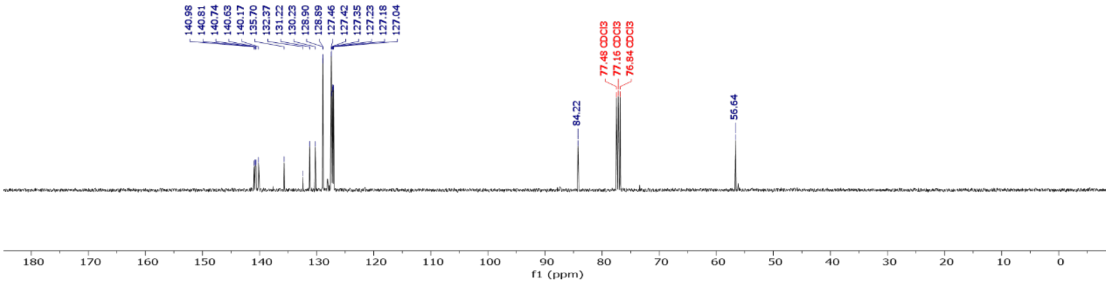

Figure S151 – ^{13}C NMR (101 MHz, CDCl$_3$, 298 K) spectrum of **3i**.

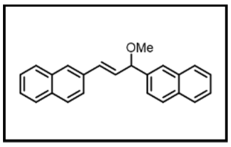

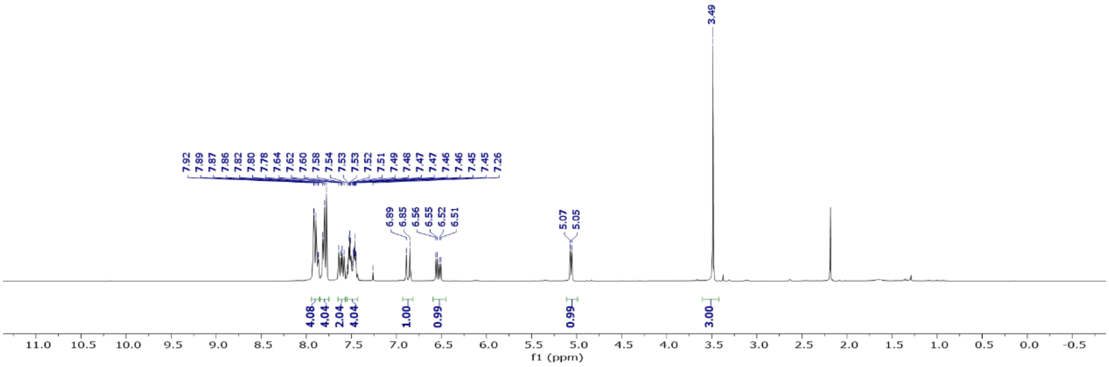

Figure S152 – ^{1}H NMR (400 MHz, CDCl$_3$, 298 K) spectrum of **3j**.

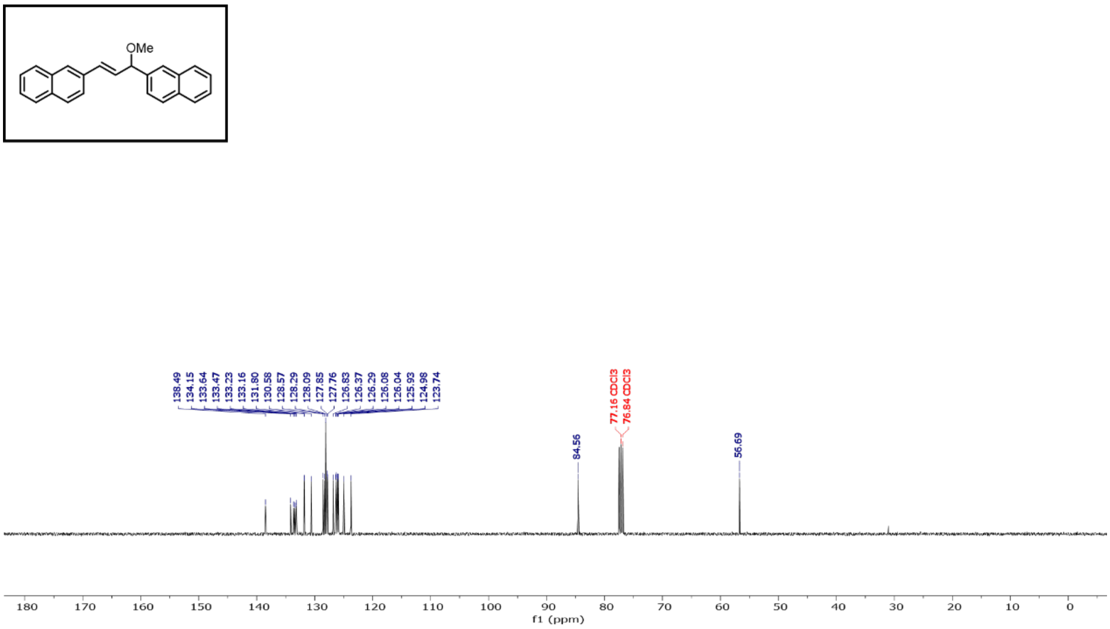

Figure S153 – ^{13}C NMR (101 MHz, CDCl$_3$, 298 K) spectrum of **3j**.

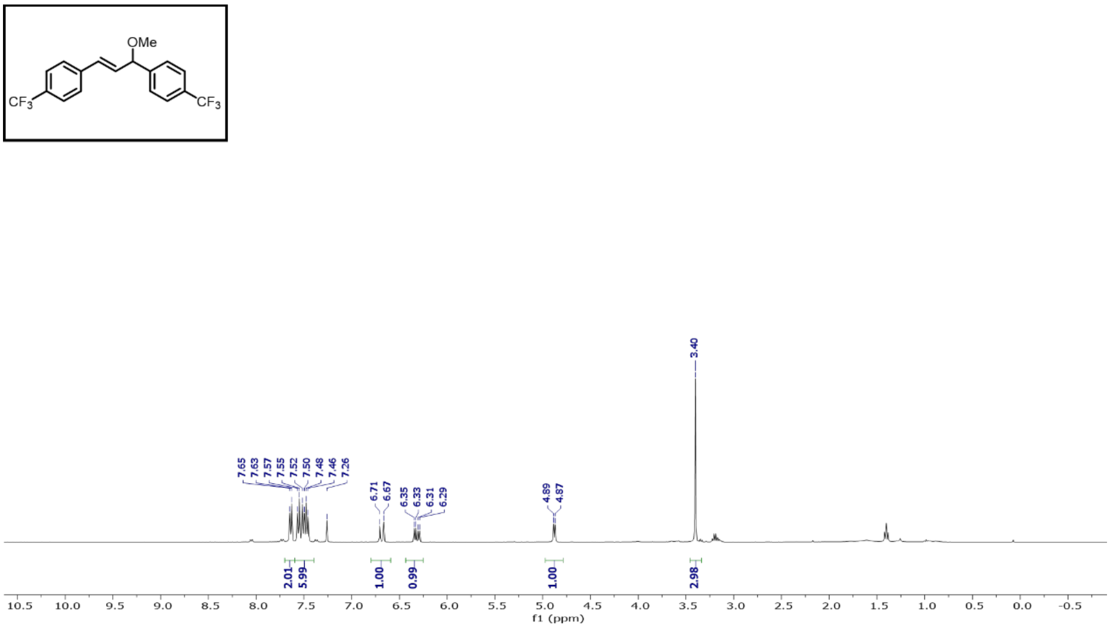

Figure S154 – ^{1}H NMR (400 MHz, CDCl$_3$, 298 K) spectrum of **3k**.

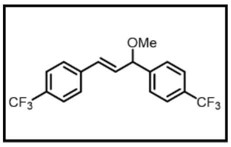

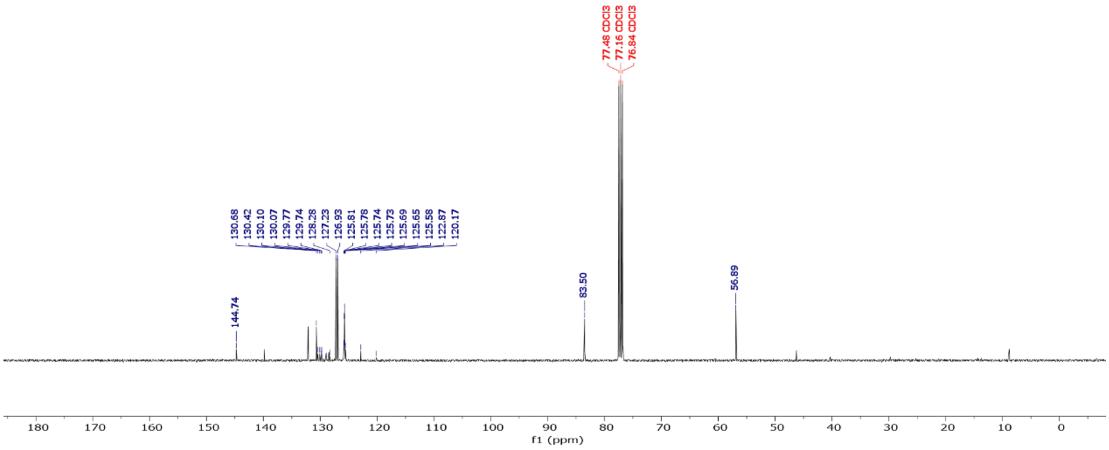

Figure S155 – ^{13}C NMR (101 MHz, $CDCl_3$, 298 K) spectrum of **3k**.

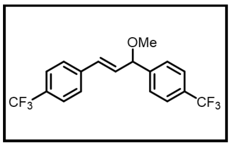

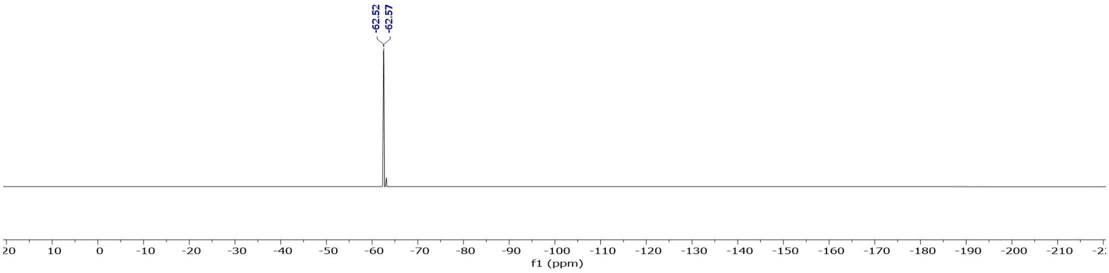

Figure S156 – ^{19}F NMR (376 MHz, $CDCl_3$, 298 K) spectrum of the reaction from **3k**.

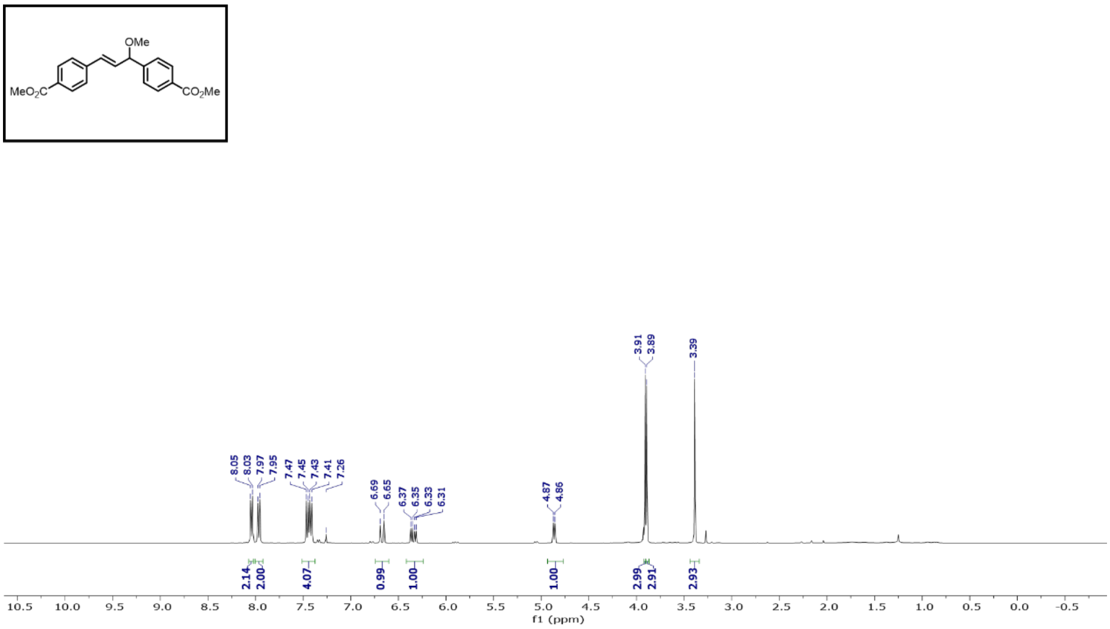

Figure S157 – ^{1}H NMR (400 MHz, CDCl$_3$, 298 K) spectrum of **3l**.

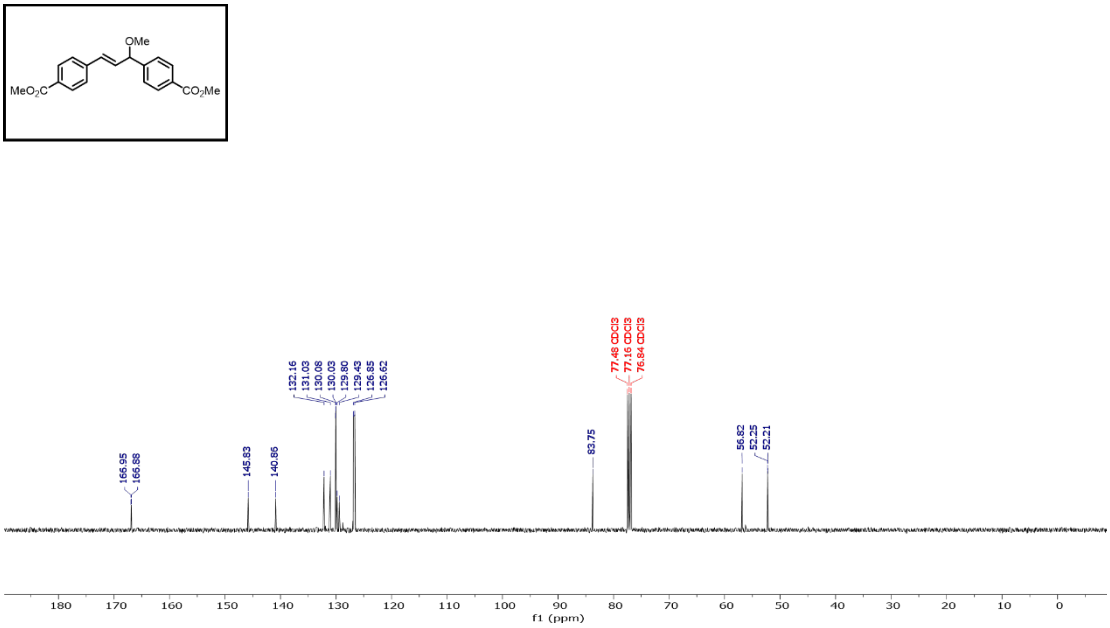

Figure S158 – ^{13}C NMR (101 MHz, CDCl$_3$, 298 K) spectrum of **3l**.

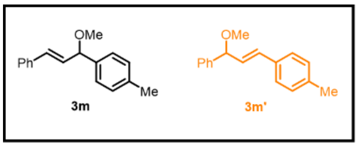

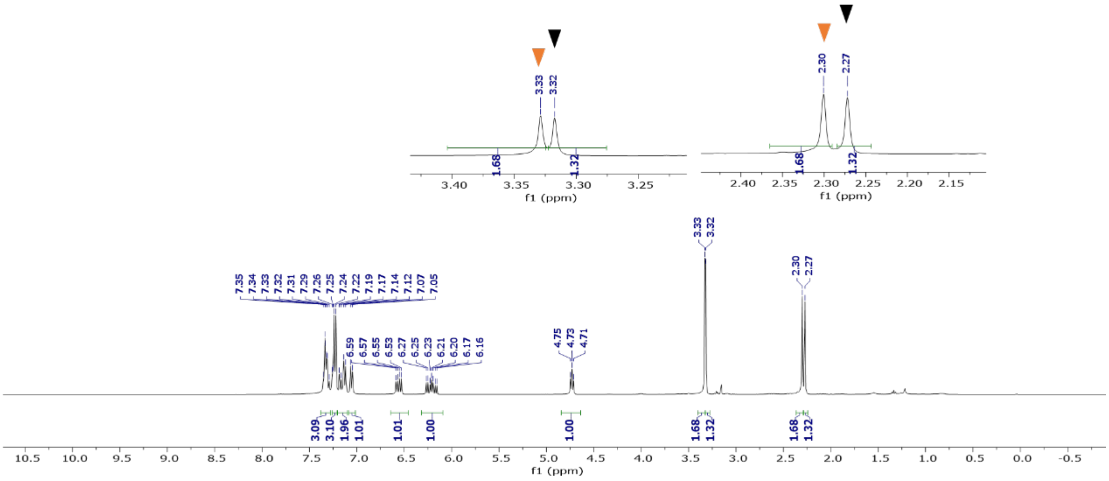

Figure S159 – ^{1}**H NMR (400 MHz,** $CDCl_3$**, 298 K)** spectrum of **3m+3m'**.

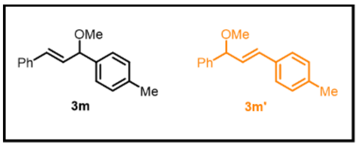

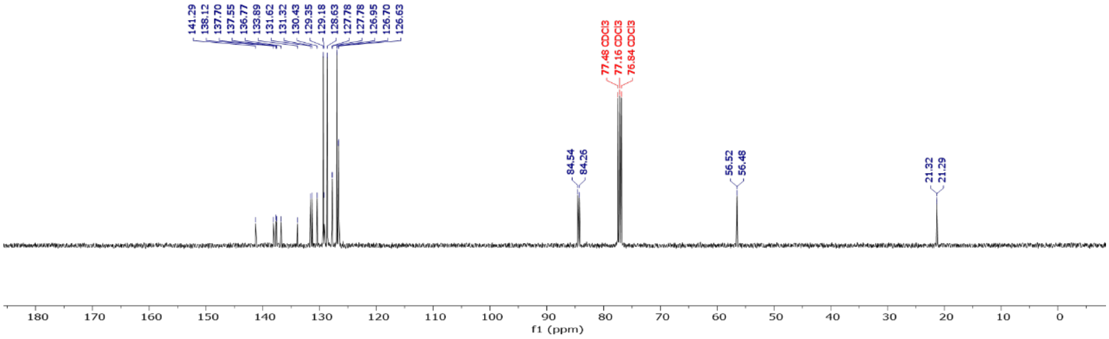

Figure S160 – ^{13}**C NMR (101 MHz,** $CDCl_3$**, 298 K)** spectrum of **3m+m'**.

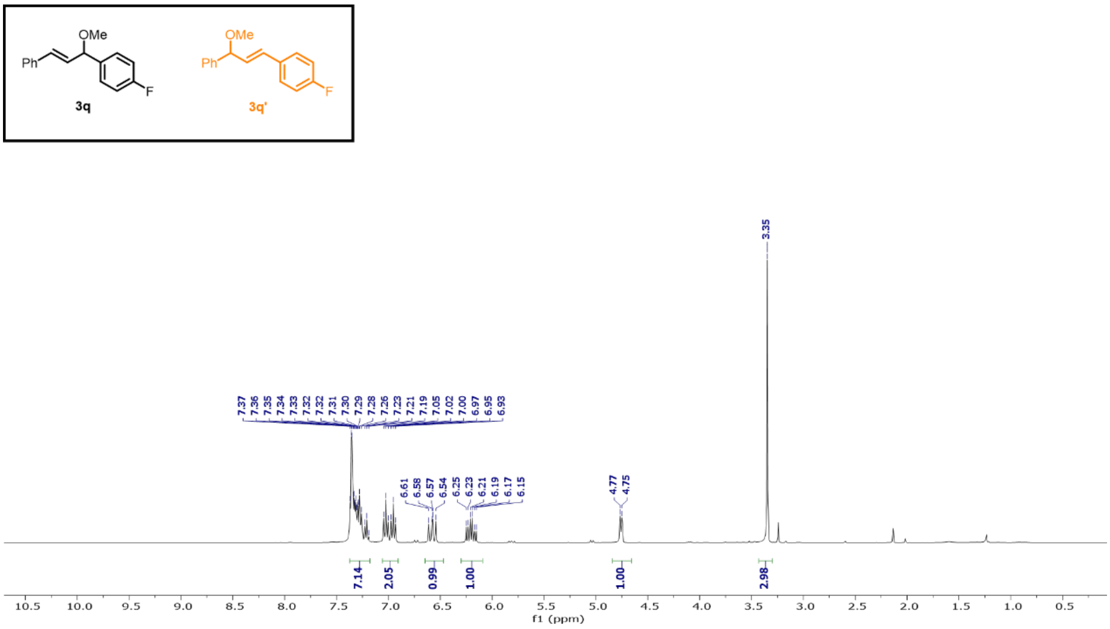

Figure S161 – ^{1}H NMR (400 MHz, CDCl$_3$, 298 K) spectrum of **3q+3q'**.

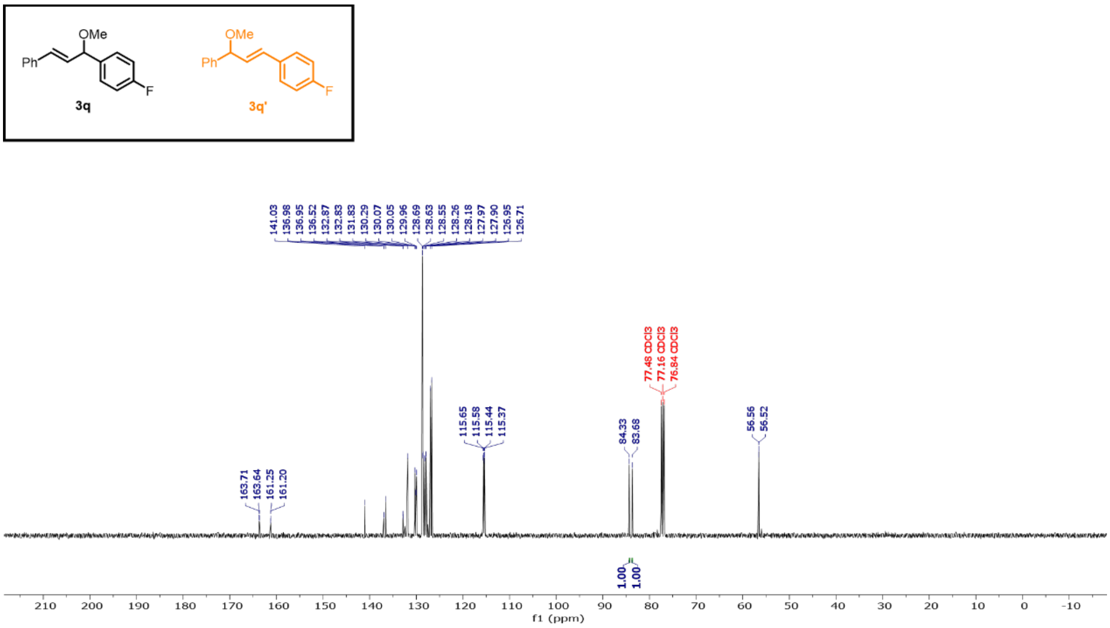

Figure S162 – ^{13}C NMR (101 MHz, CDCl$_3$, 298 K) spectrum of **3q+q'**.

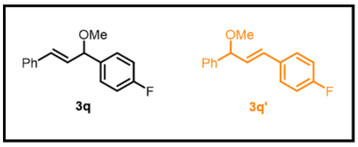

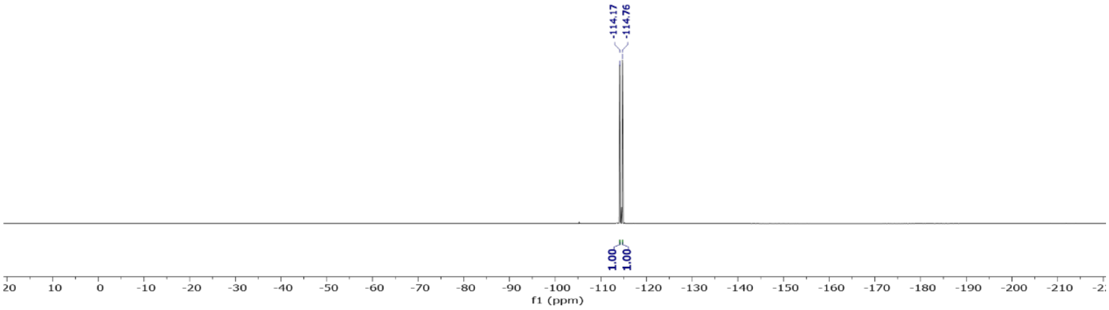

Figure S163 – ^{19}F NMR (376 MHz, $CDCl_3$, 298 K) spectrum of the reaction from **3q+3q'**.

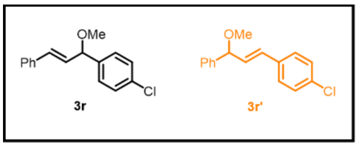

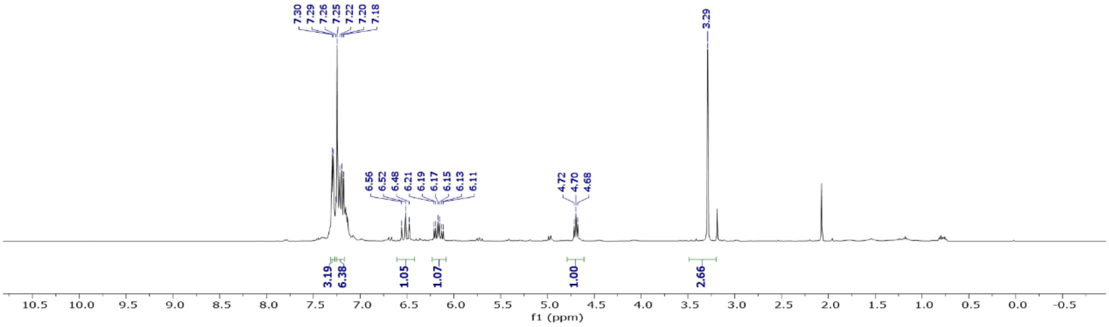

Figure S164 – ^{1}H NMR (400 MHz, $CDCl_3$, 298 K) spectrum of **3r+3r'**.

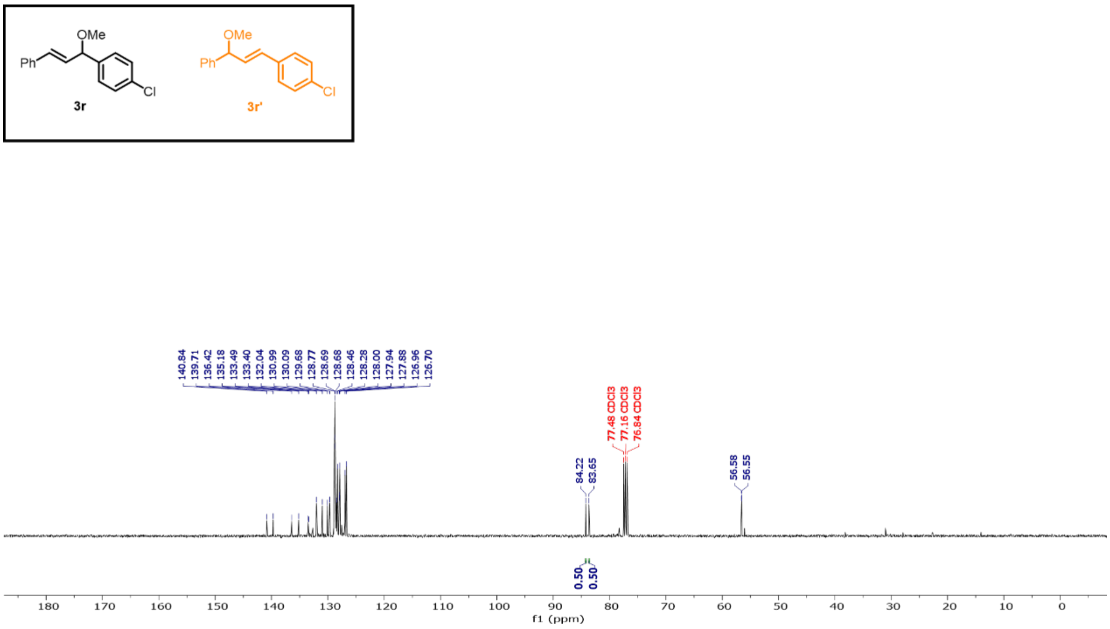

Figure S165 – ^{13}C NMR (101 MHz, CDCl$_3$, 298 K) spectrum of **3r+r'**.

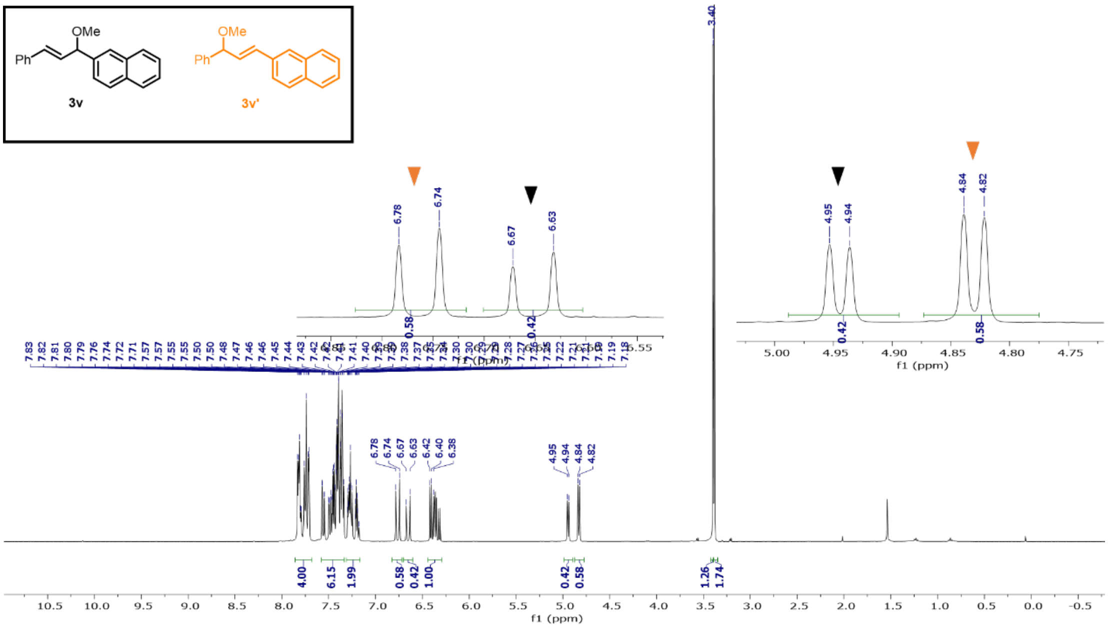

Figure S166 – ^{1}H NMR (400 MHz, CDCl$_3$, 298 K) spectrum of **3v+3v'**.

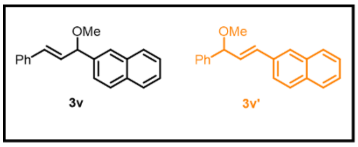

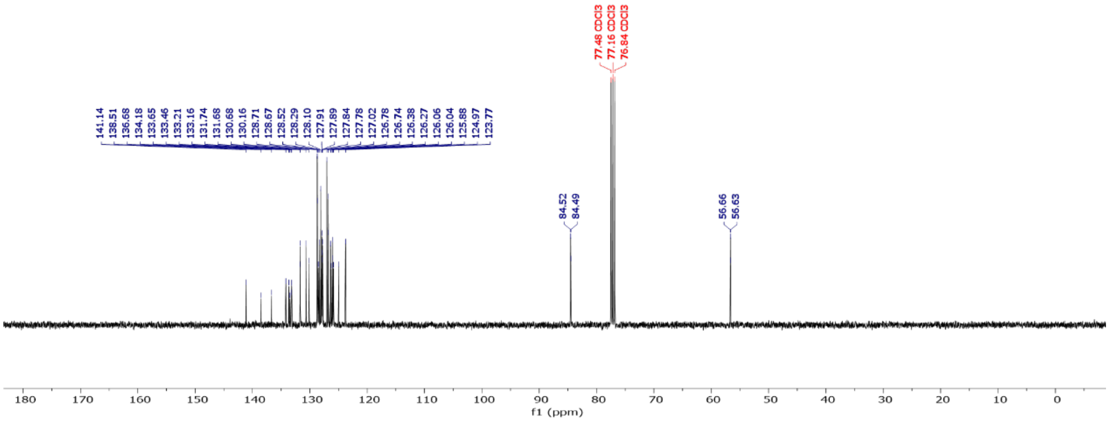

Figure S167 – ^{13}C NMR (101 MHz, CDCl$_3$, 298 K) spectrum of **3v+v'**.

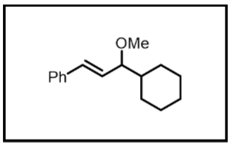

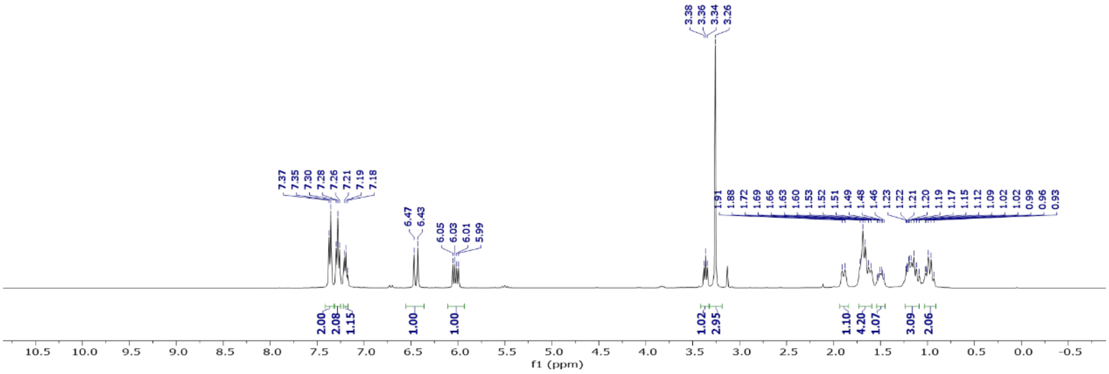

Figure S168 – ^{1}H NMR (400 MHz, CDCl$_3$, 298 K) spectrum of **3x**.

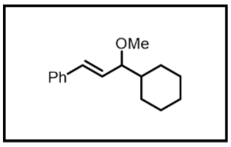

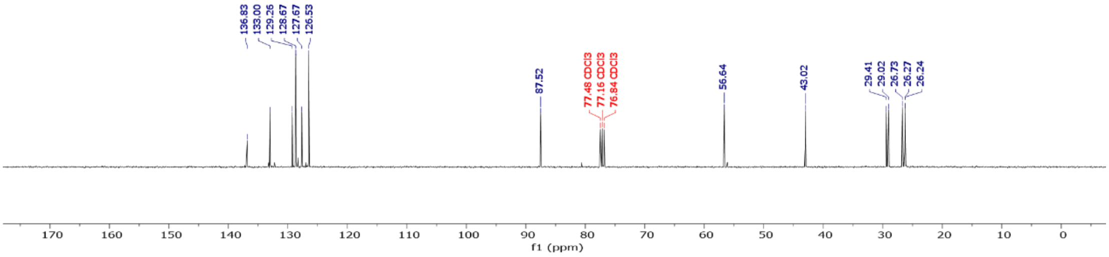

Figure S169 – ^{13}C NMR (101 MHz, CDCl$_3$, 298 K) spectrum of **3x**.

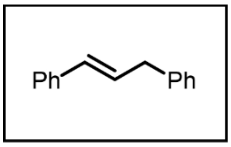

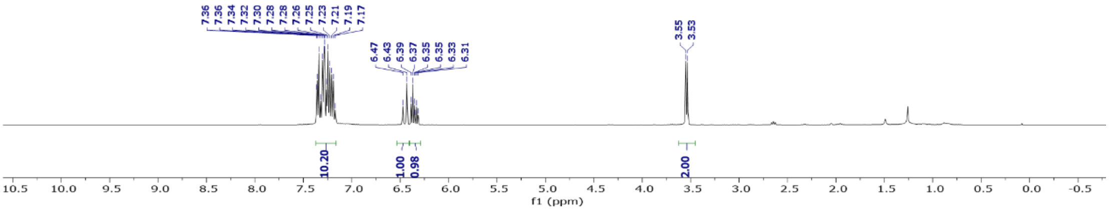

Figure S170 – ^{1}H NMR (400 MHz, CDCl$_3$, 298 K) spectrum of **4a**.

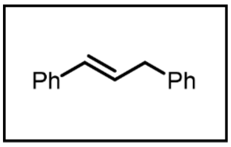

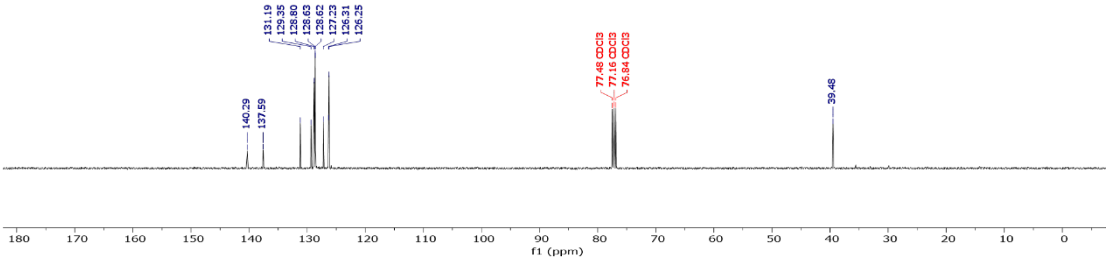

Figure S171 – ^{13}C NMR (101 MHz, CDCl$_3$, 298 K) spectrum of **4a**.

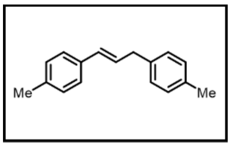

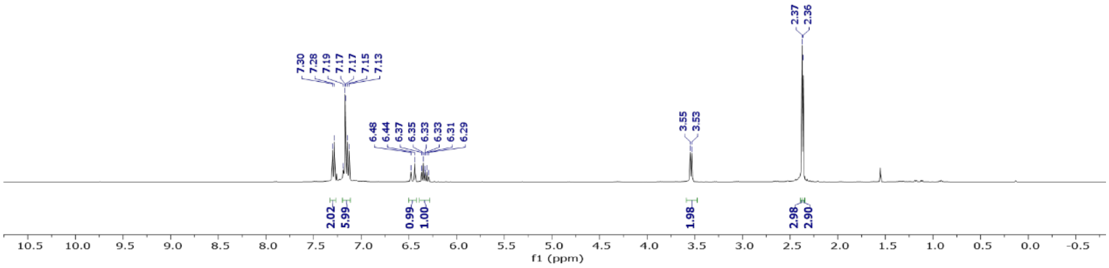

Figure S172 – ^{1}H NMR (400 MHz, CDCl$_3$, 298 K) spectrum of **4b**.

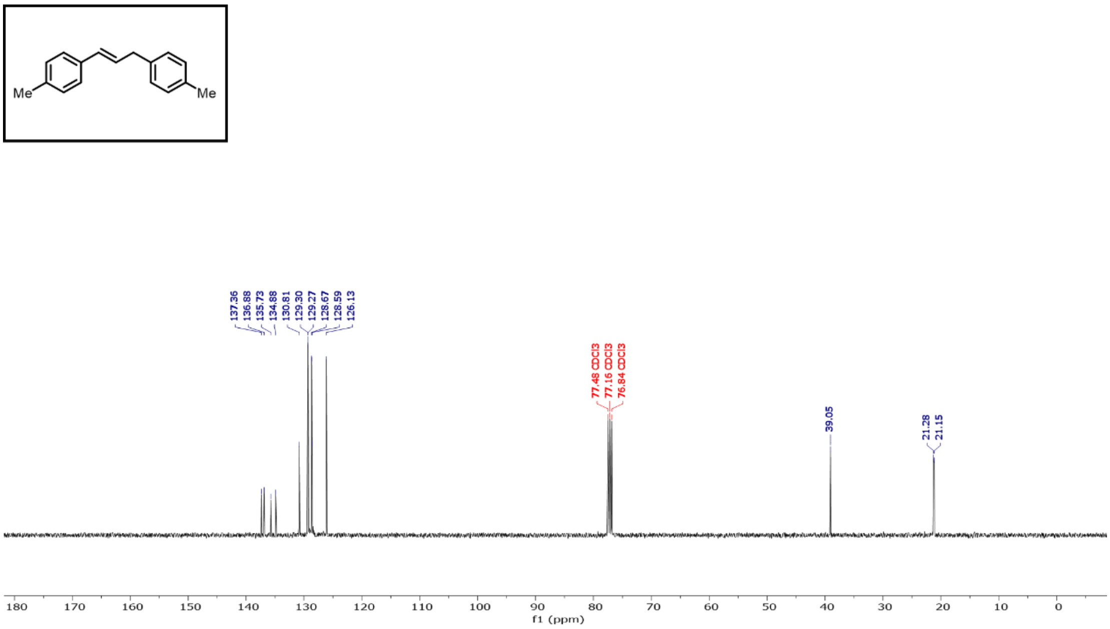

Figure S173 – ^{13}C NMR (101 MHz, $CDCl_3$, 298 K) spectrum of **4b**.

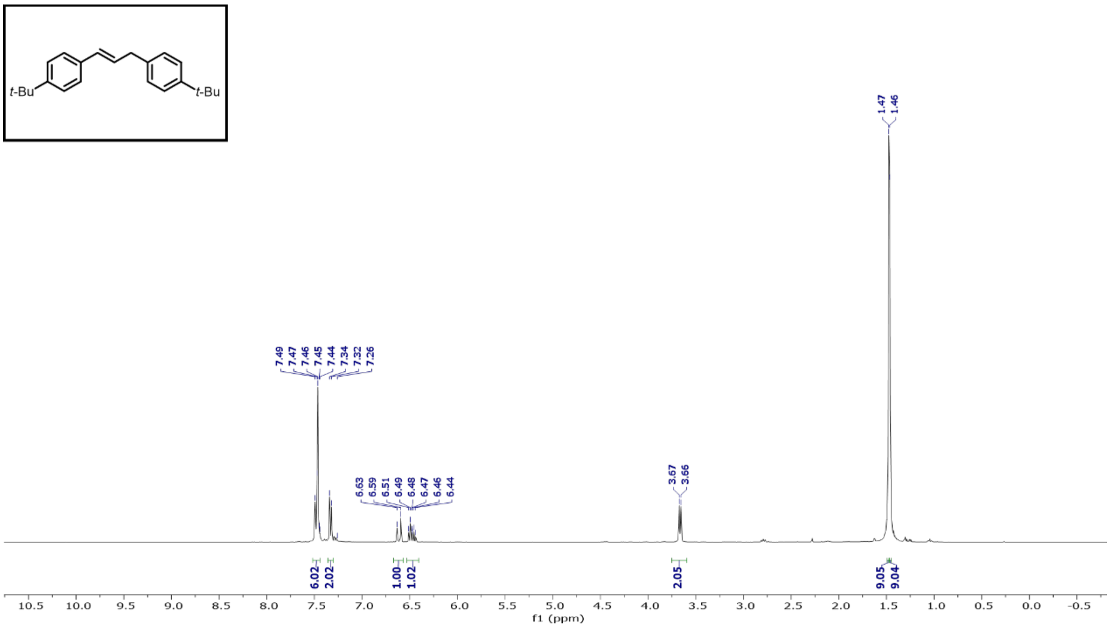

Figure S174 – ^{1}H NMR (400 MHz, $CDCl_3$, 298 K) spectrum of **4c**.

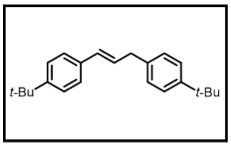

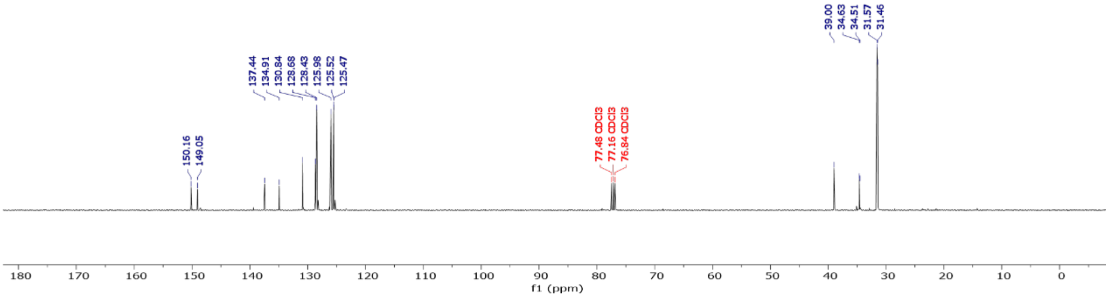

Figure S175 – ^{13}C NMR (101 MHz, $CDCl_3$, 298 K) spectrum of **4c**.

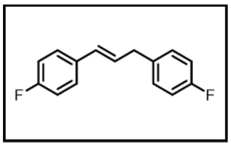

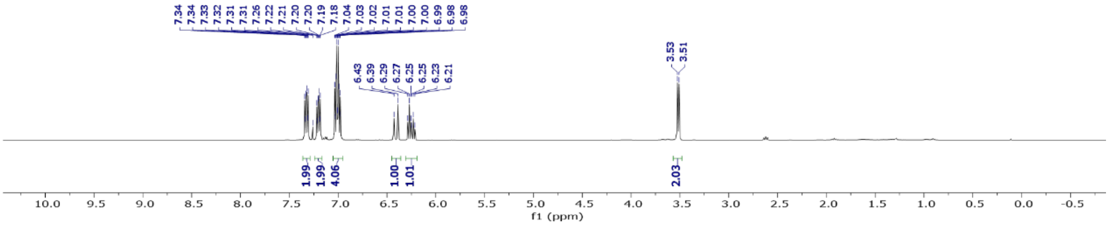

Figure S176 – ^{1}H NMR (400 MHz, $CDCl_3$, 298 K) spectrum of **4e**.

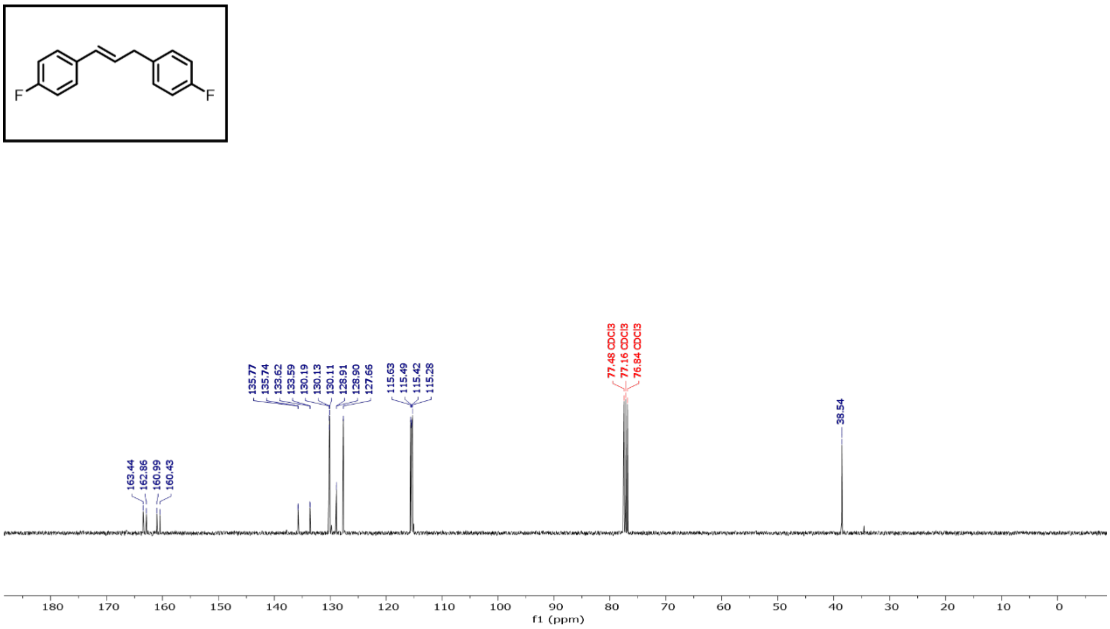

Figure S177 – ^{13}C NMR (101 MHz, $CDCl_3$, 298 K) spectrum of **4e**.

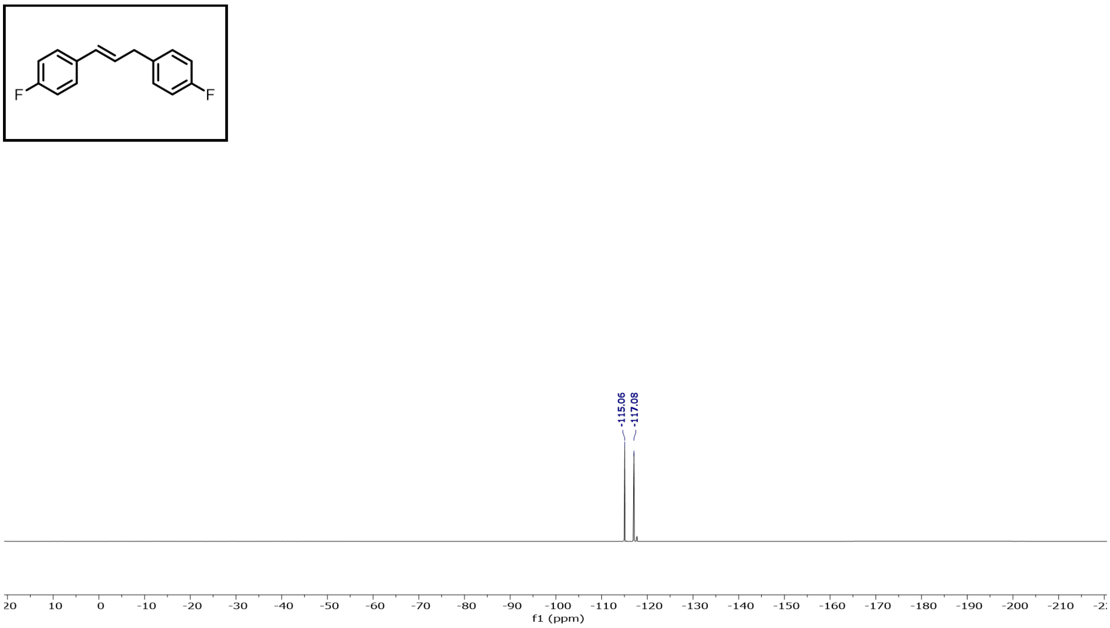

Figure S178 – ^{19}F NMR (376 MHz, $CDCl_3$, 298 K) spectrum of the reaction from **4e**.

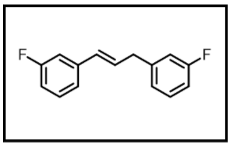

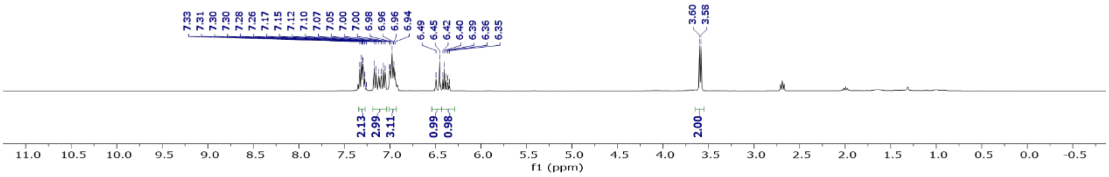

Figure S179 – ^{1}H NMR (400 MHz, $CDCl_3$, 298 K) spectrum of **4f**.

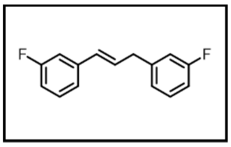

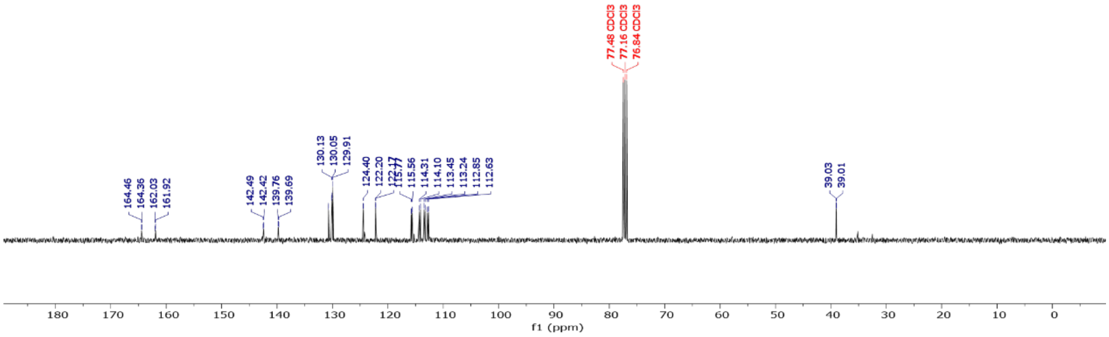

Figure S180 – ^{13}C NMR (101 MHz, $CDCl_3$, 298 K) spectrum of **4f**.

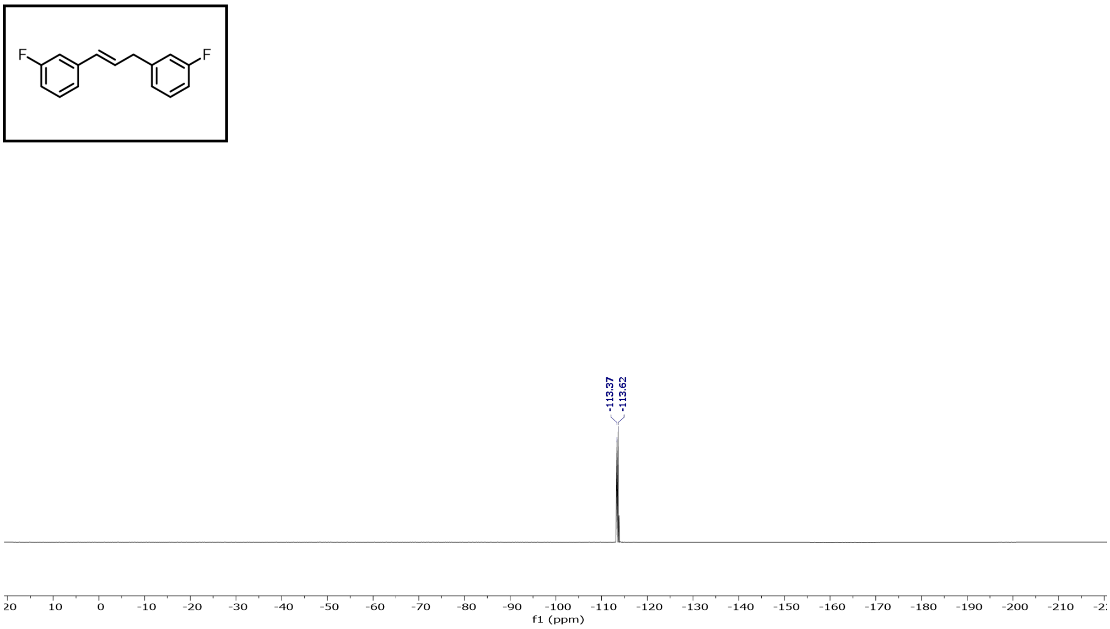

Figure S181 – ^{19}F NMR (376 MHz, CDCl$_3$, 298 K) spectrum of the reaction from **4f**.

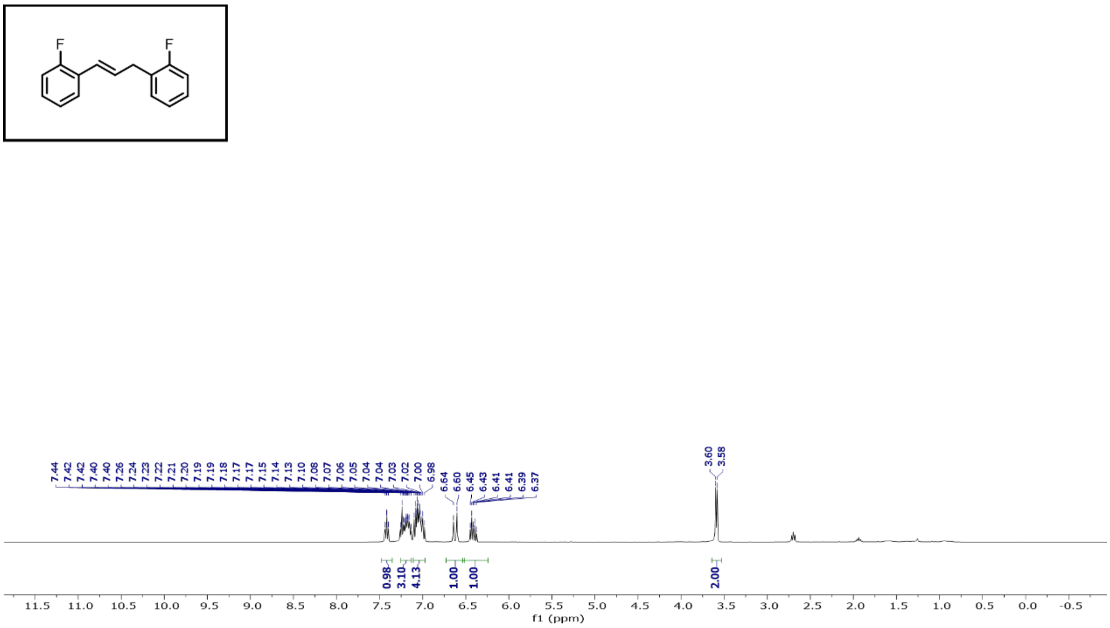

Figure S182 – ^{1}H NMR (400 MHz, CDCl$_3$, 298 K) spectrum of **4g**.

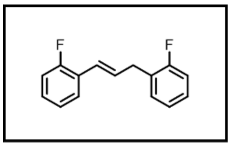

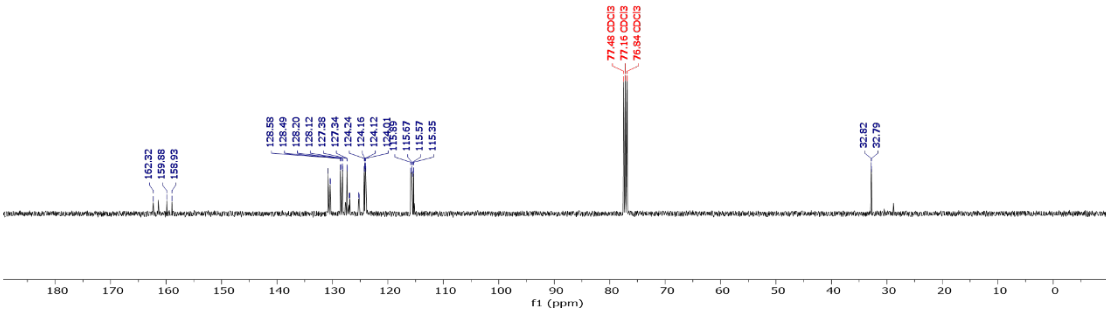

Figure S183 – ^{13}C NMR (101 MHz, $CDCl_3$, 298 K) spectrum of **4g**.

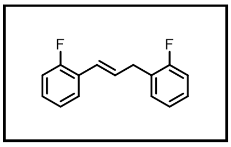

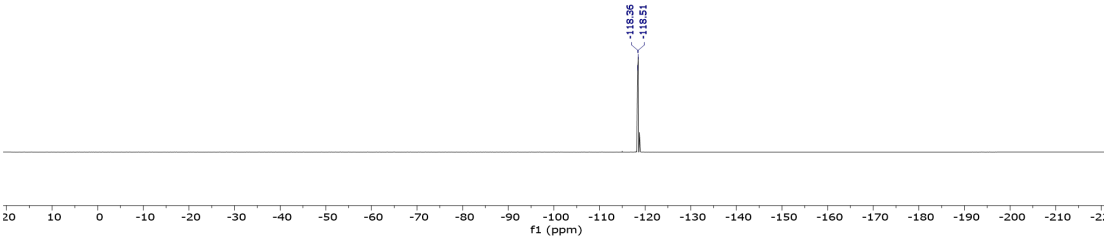

Figure S184 – ^{19}F NMR (376 MHz, $CDCl_3$, 298 K) spectrum of the reaction from **4g**.

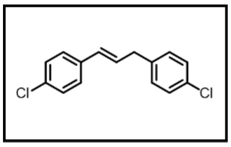

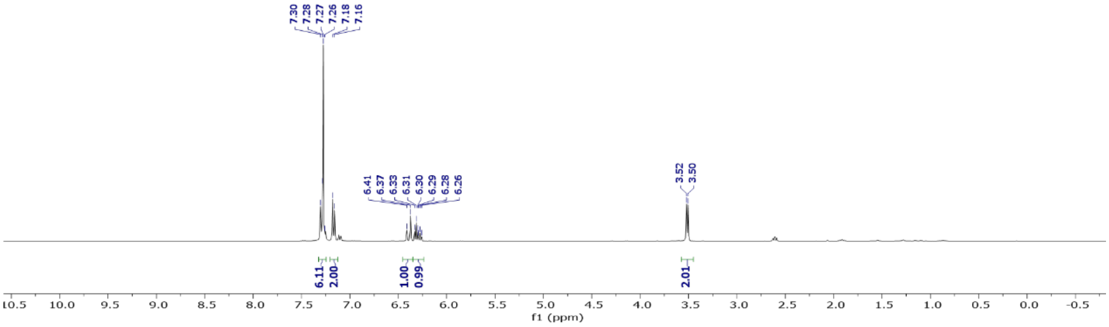

Figure S185 – ^{1}H NMR (400 MHz, $CDCl_3$, 298 K) spectrum of **4h**.

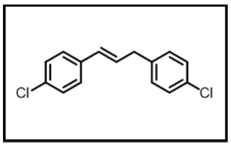

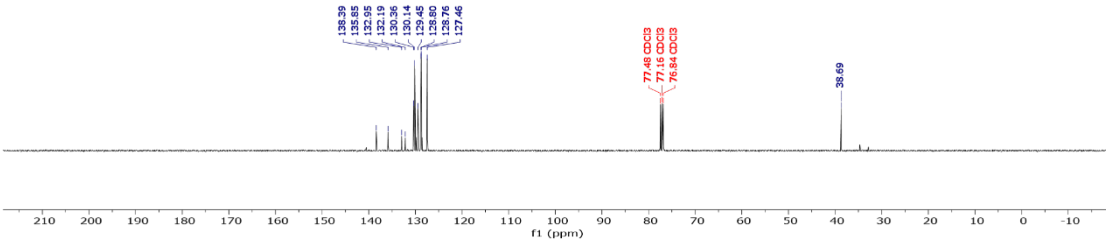

Figure S186 – ^{13}C NMR (101 MHz, $CDCl_3$, 298 K) spectrum of **4h**.

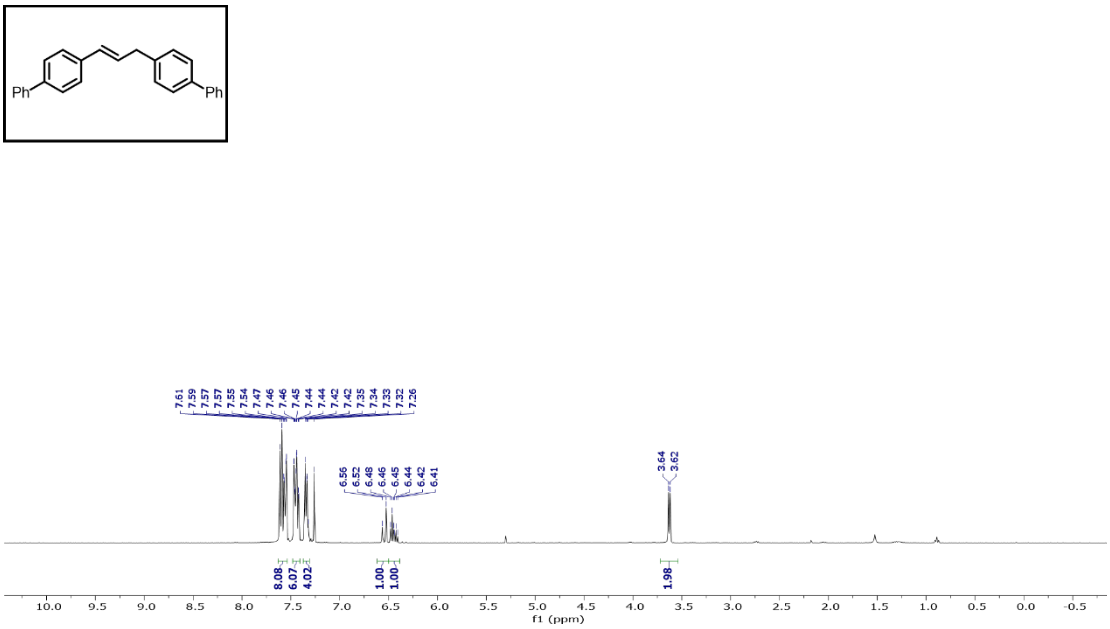

Figure S187 – ^{1}H NMR (400 MHz, $CDCl_3$, 298 K) spectrum of **4i**.

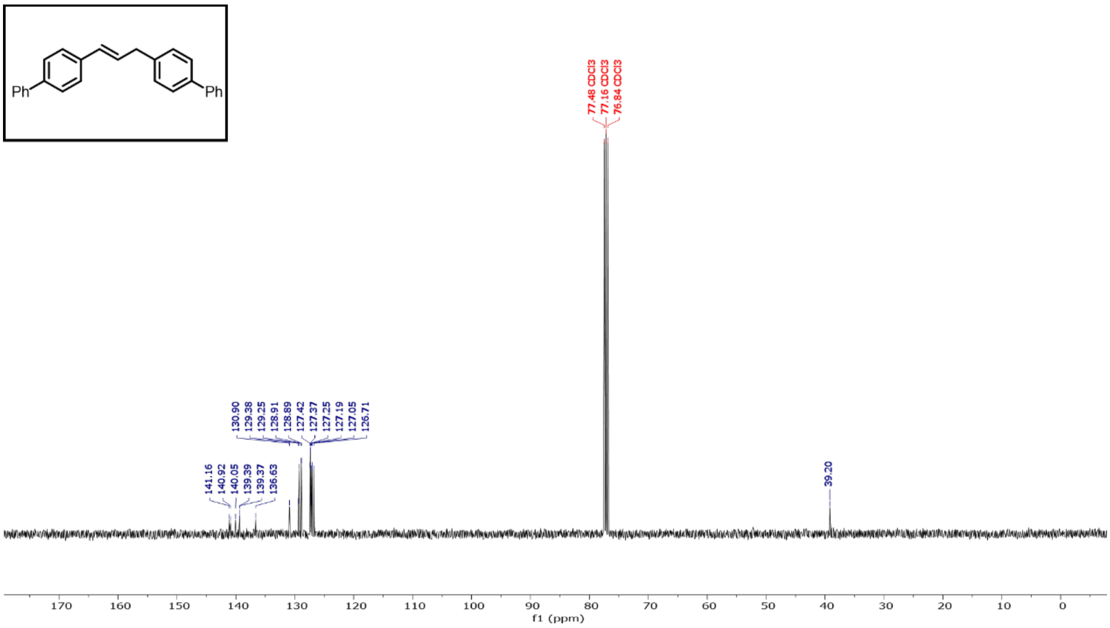

Figure S188 – ^{13}C NMR (101 MHz, $CDCl_3$, 298 K) spectrum of **4i**.

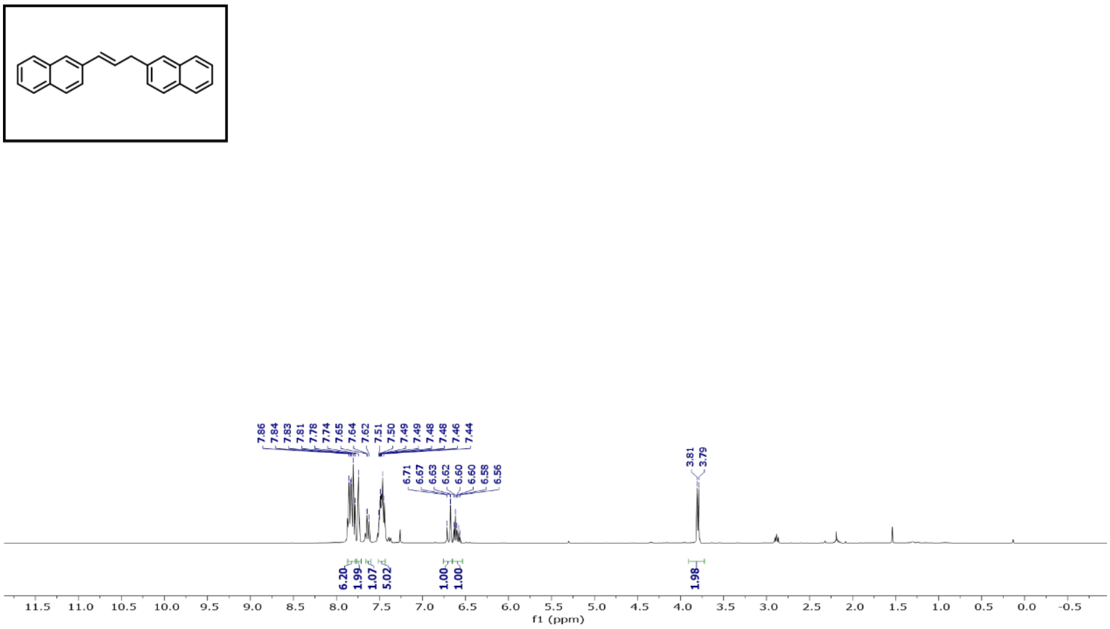

Figure S189 – ^{1}H NMR (400 MHz, $CDCl_3$, 298 K) spectrum of **4j**.

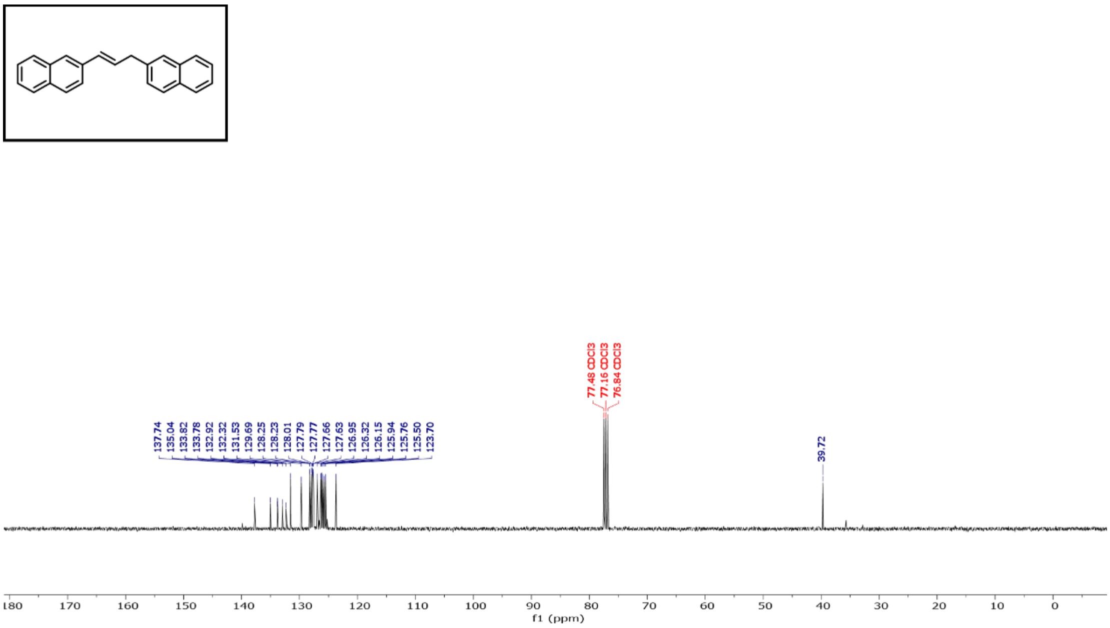

Figure S190 – ^{13}C NMR (101 MHz, $CDCl_3$, 298 K) spectrum of **4j**.

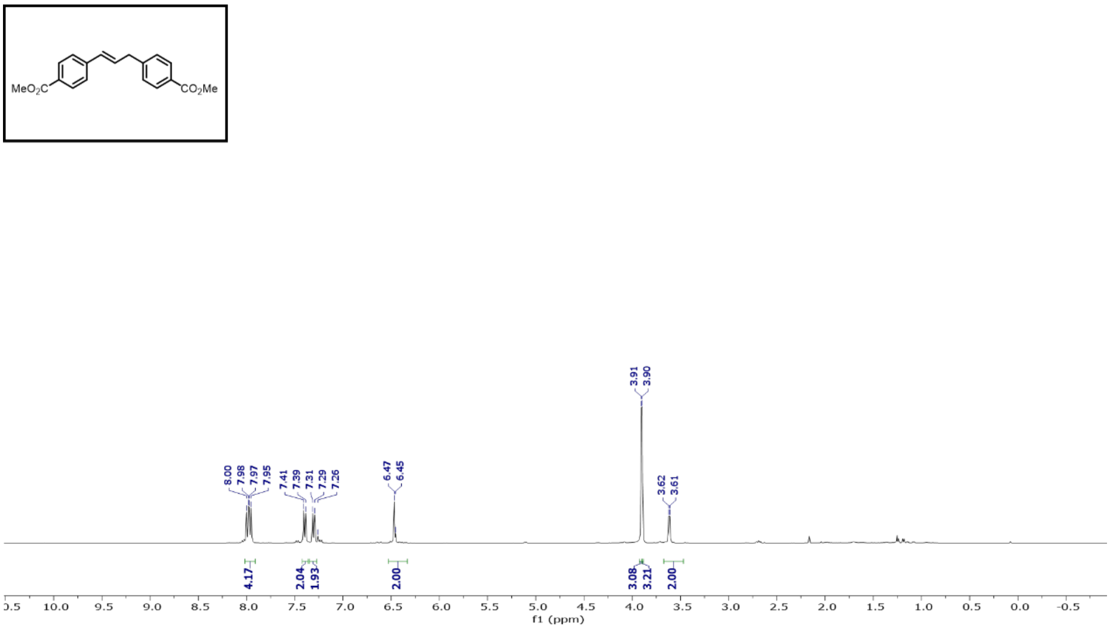

Figure S191 – ^{1}H NMR (400 MHz, CDCl$_3$, 298 K) spectrum of **4l**.

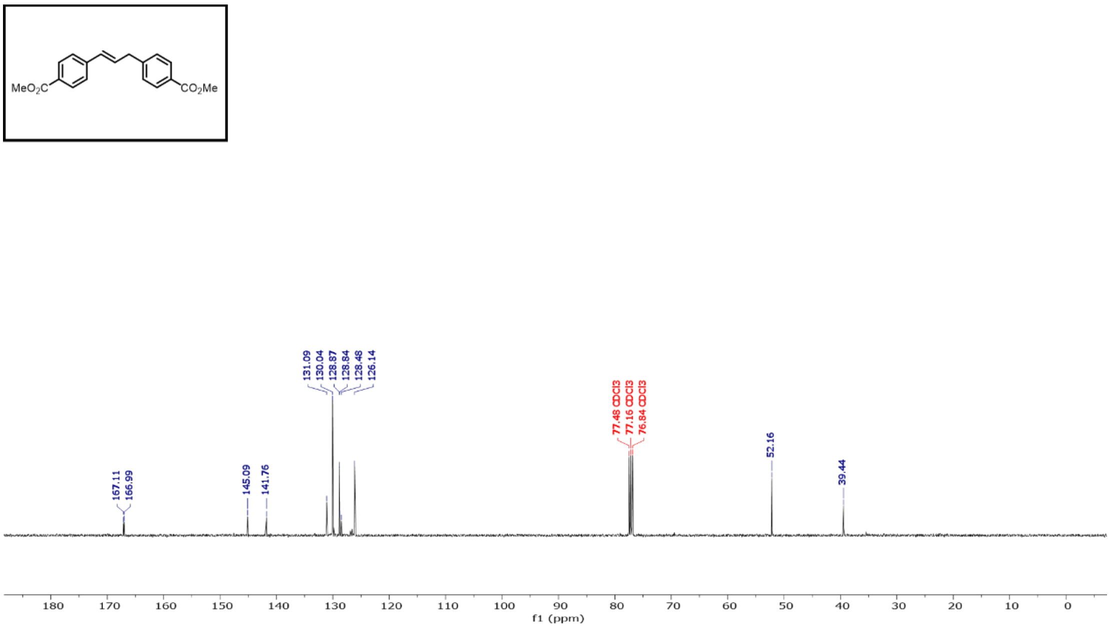

Figure S192 – ^{13}C NMR (101 MHz, CDCl$_3$, 298 K) spectrum of **4l**.

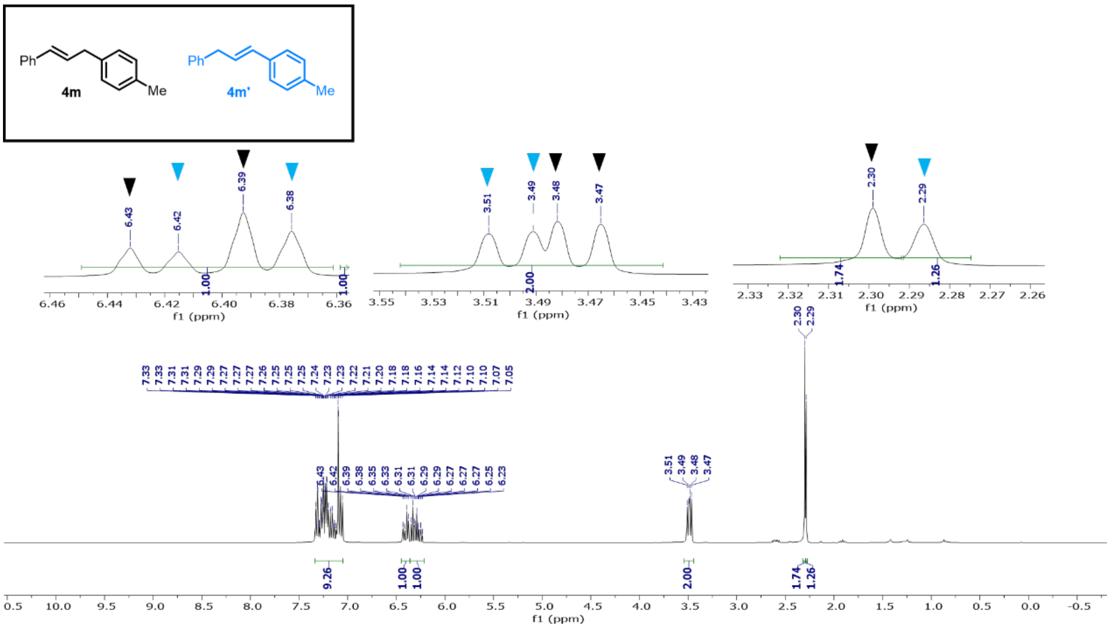

Figure S193 – ^{1}H NMR (400 MHz, CDCl$_3$, 298 K) spectrum of **4m+m'**.

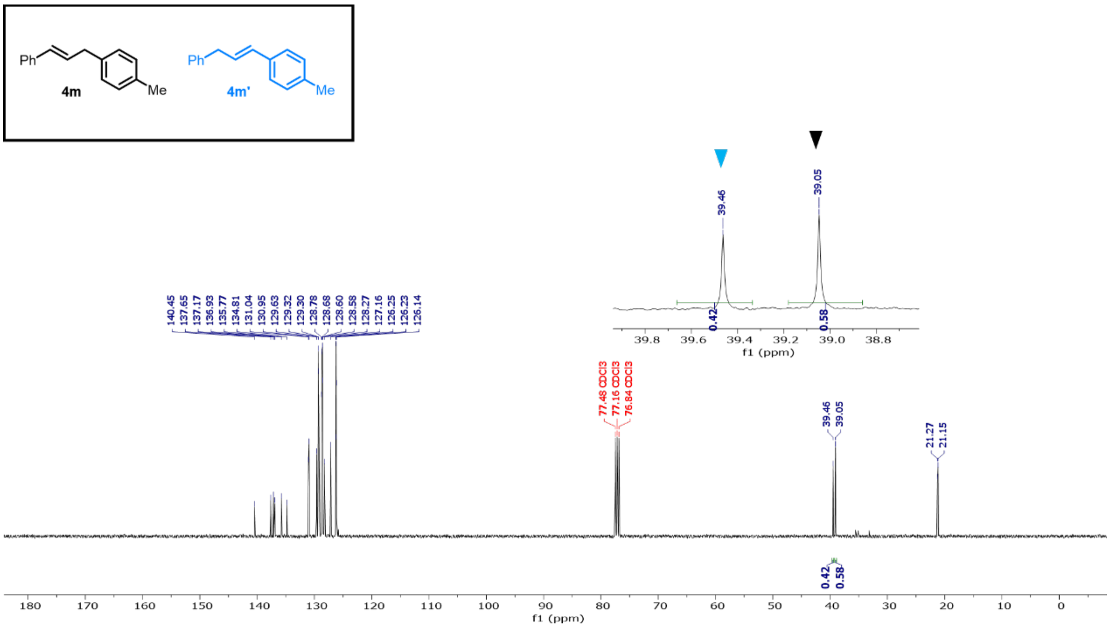

Figure S194 – ^{13}C NMR (101 MHz, CDCl$_3$, 298 K) spectrum of **4m+m'**.

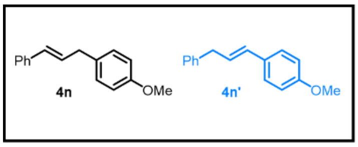

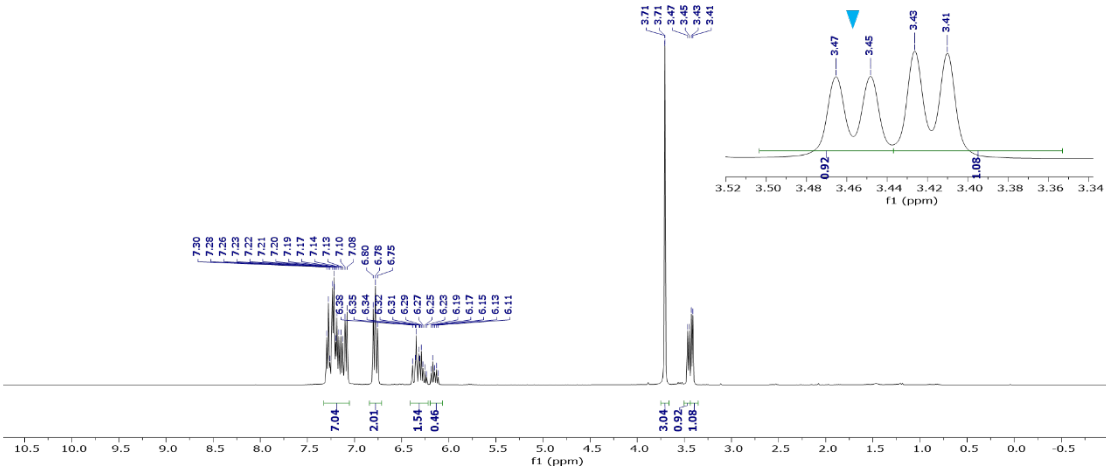

Figure S195 – ^{1}H NMR (400 MHz, CDCl$_3$, 298 K) spectrum of **4n+n'**.

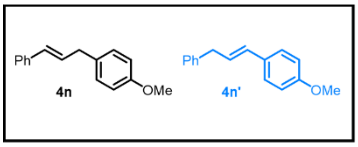

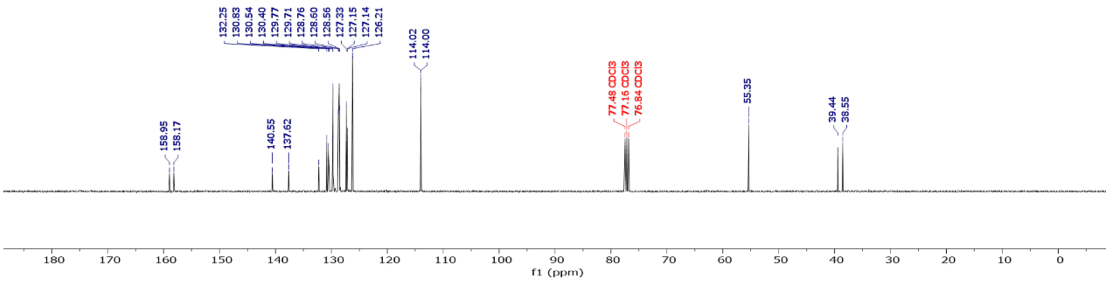

Figure S196 – ^{13}C NMR (101 MHz, CDCl$_3$, 298 K) spectrum of **4n+n'**.

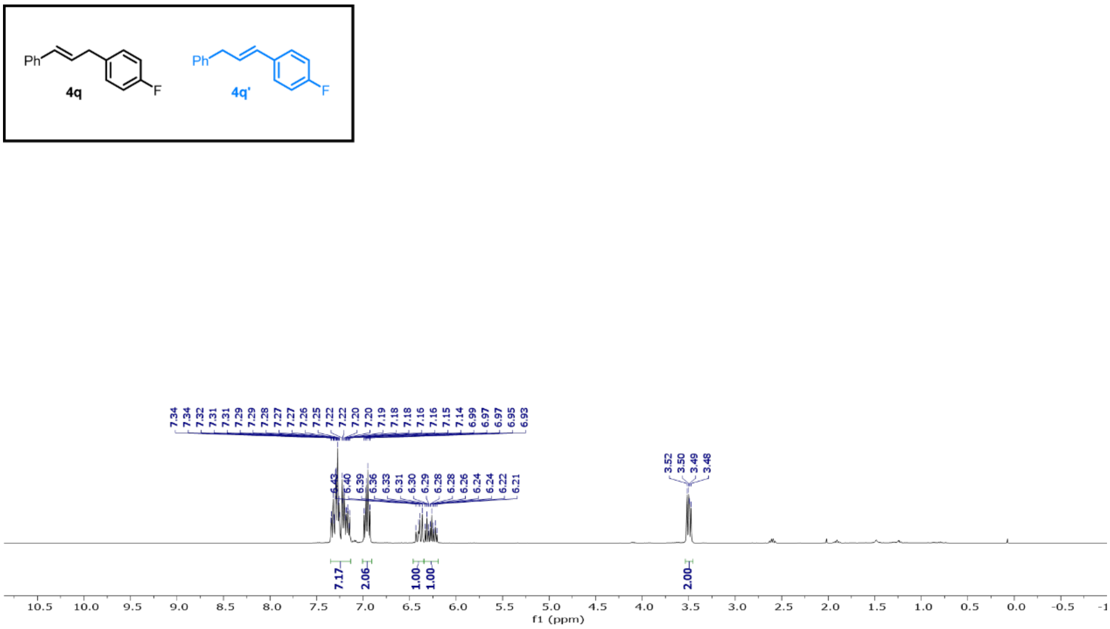

Figure S197 – ¹H NMR (400 MHz, $CDCl_3$, 298 K) spectrum of **4q+q'**.

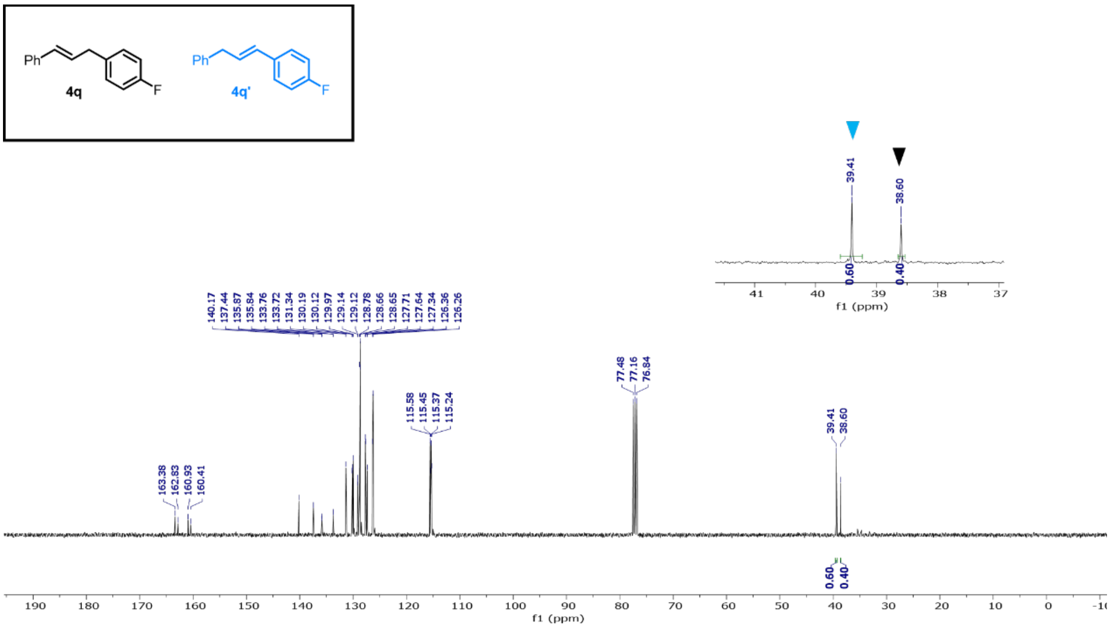

Figure S198 – ¹³C NMR (101 MHz, $CDCl_3$, 298 K) spectrum of **4q+q'**.

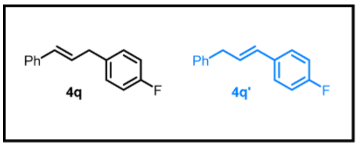

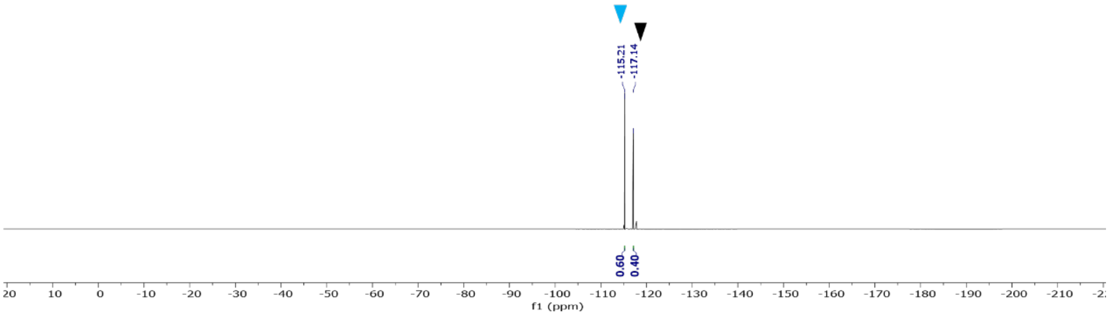

Figure S199 – ^{19}F NMR (376 MHz, CDCl$_3$, 298 K) spectrum of the reaction from **4q+4q'**.

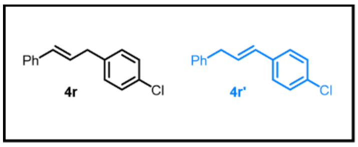

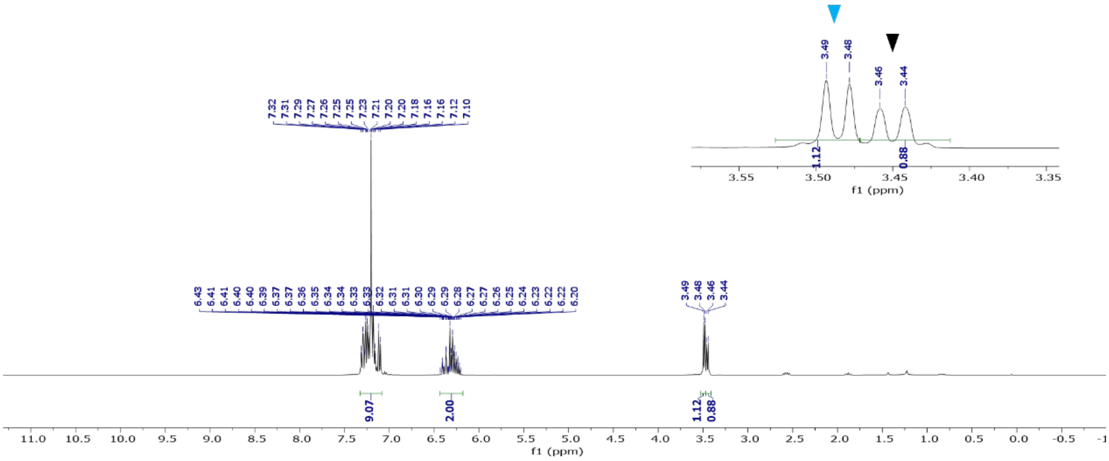

Figure S200 – ^{1}H NMR (400 MHz, CDCl$_3$, 298 K) spectrum of **4r+r'**.

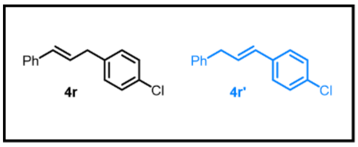

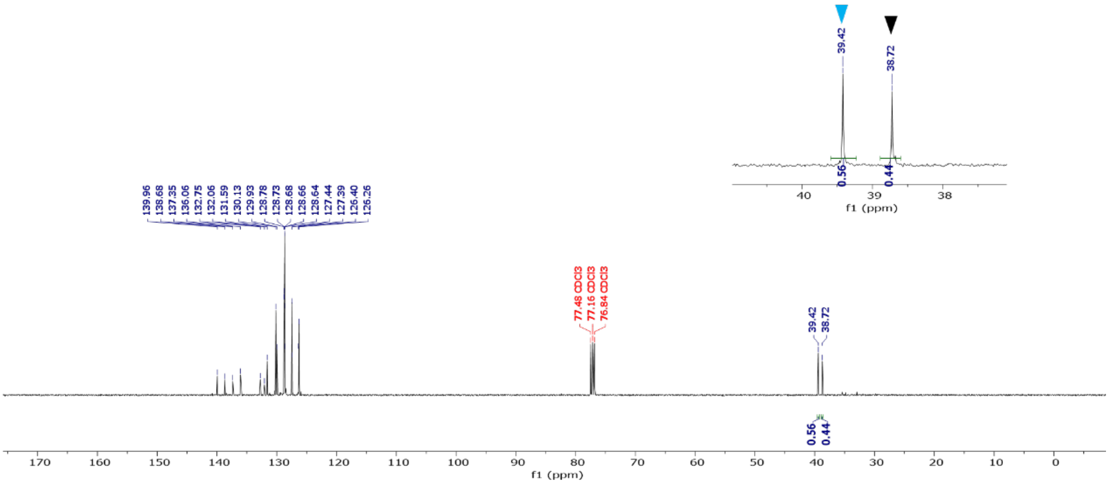

Figure S201 – ^{13}C NMR (101 MHz, CDCl$_3$, 298 K) spectrum of **4r+r'**.

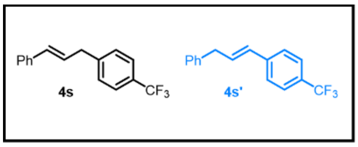

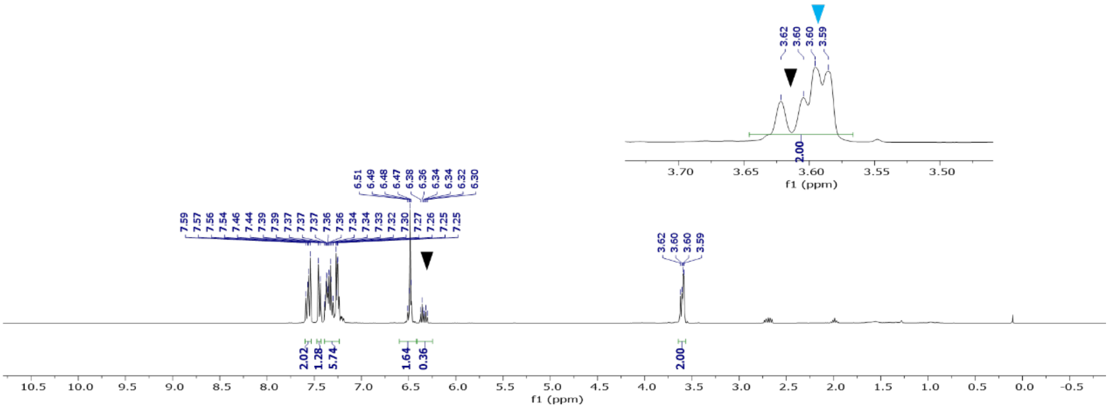

Figure S202 – ^{1}H NMR (400 MHz, CDCl$_3$, 298 K) spectrum of **4s+s'**.

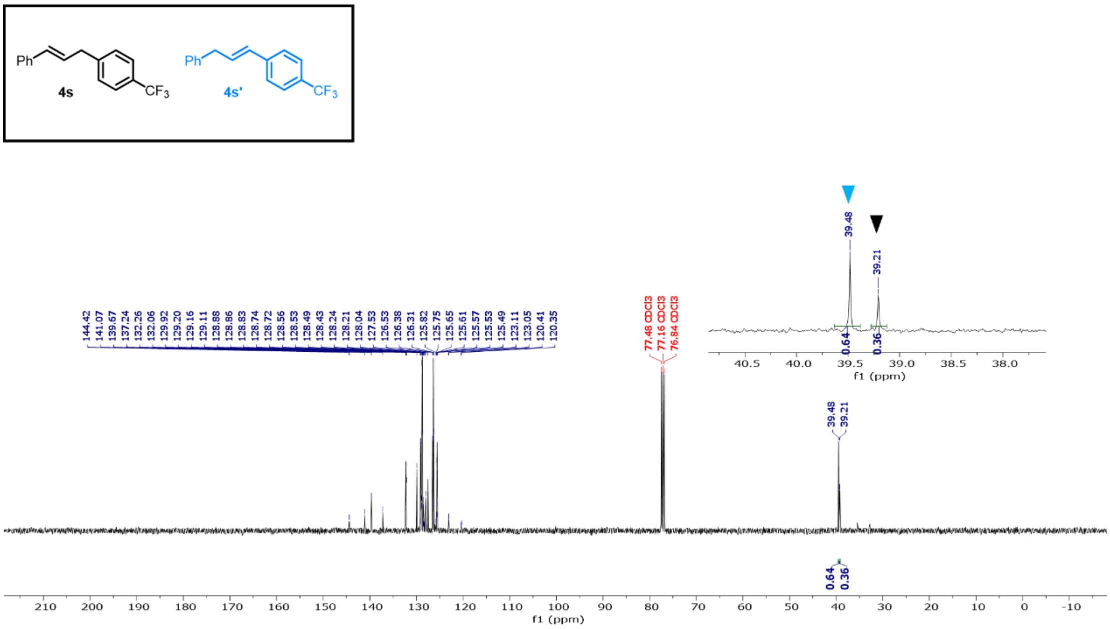

Figure S203 – ^{13}**C NMR (101 MHz, CDCl$_3$, 298 K)** spectrum of **4s+4s'**.

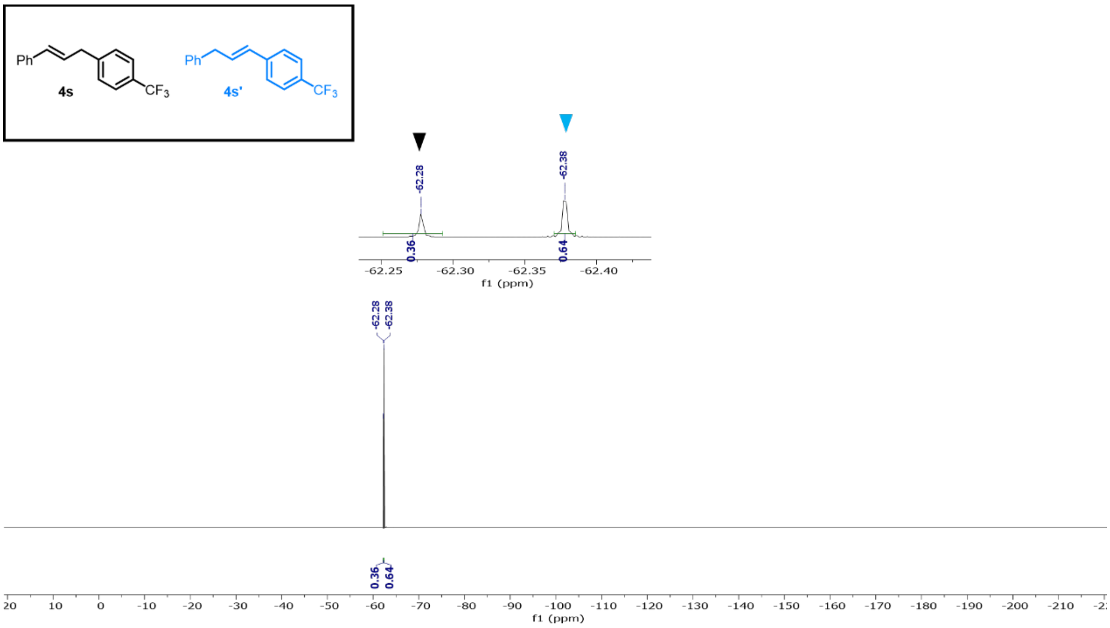

Figure S204 – ^{19}**F NMR (376 MHz, CDCl$_3$, 298 K)** spectrum of the reaction from **4s+4s'**.

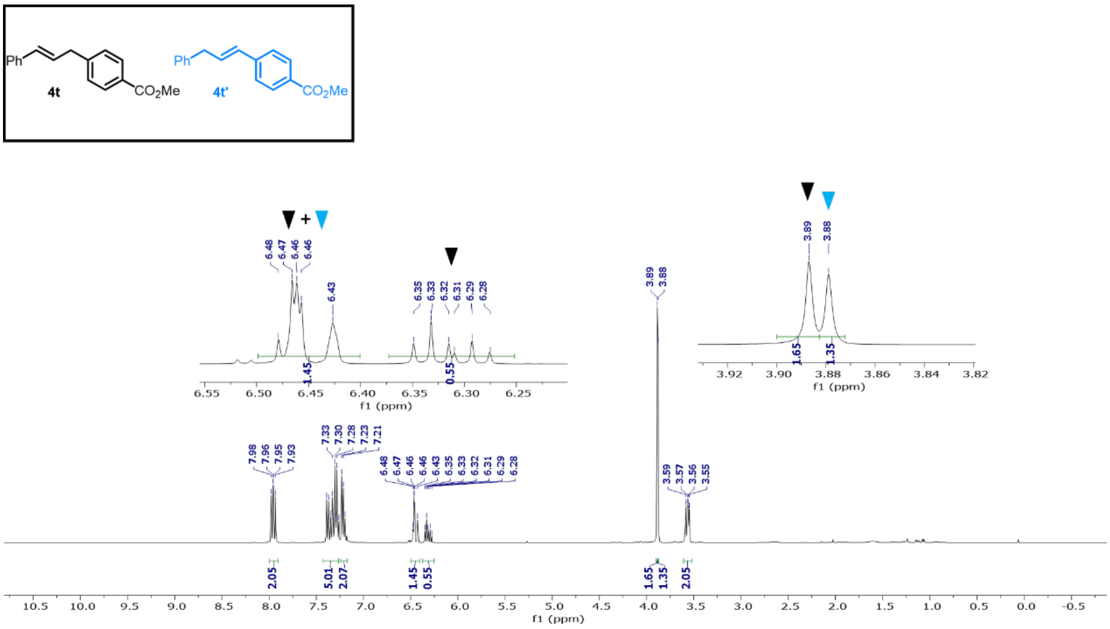

Figure S205 – ^{1}H NMR (400 MHz, CDCl$_3$, 298 K) spectrum of **4t+t'**.

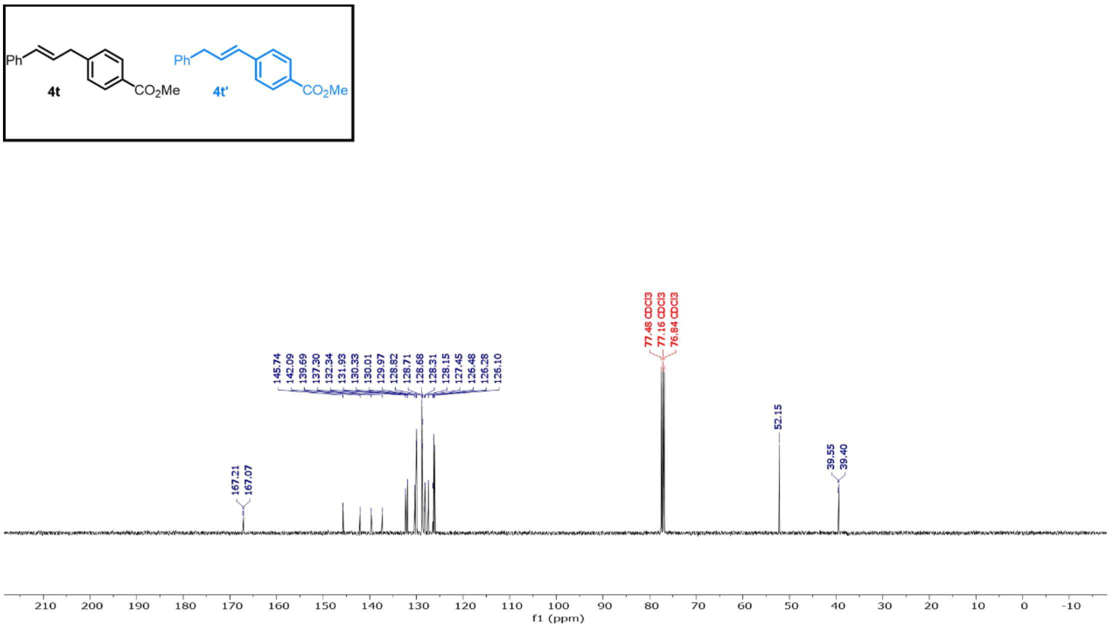

Figure S206 – ^{13}C NMR (101 MHz, CDCl$_3$, 298 K) spectrum of **4t+4t'**.

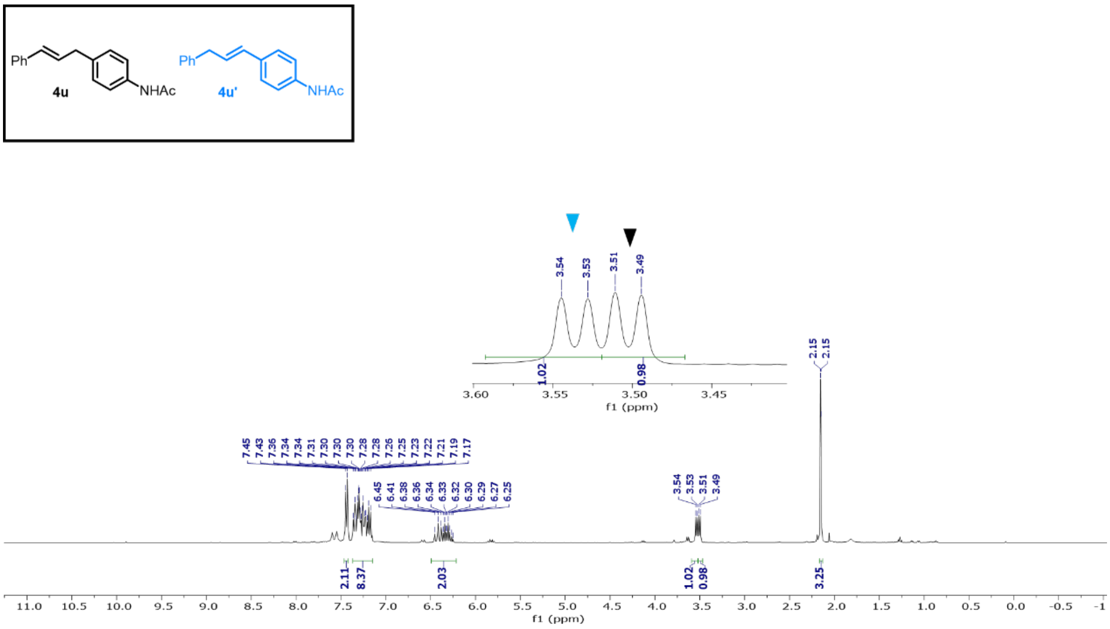

Figure S207 – ^{1}H NMR (400 MHz, CDCl$_3$, 298 K) spectrum of **4u+u'**.

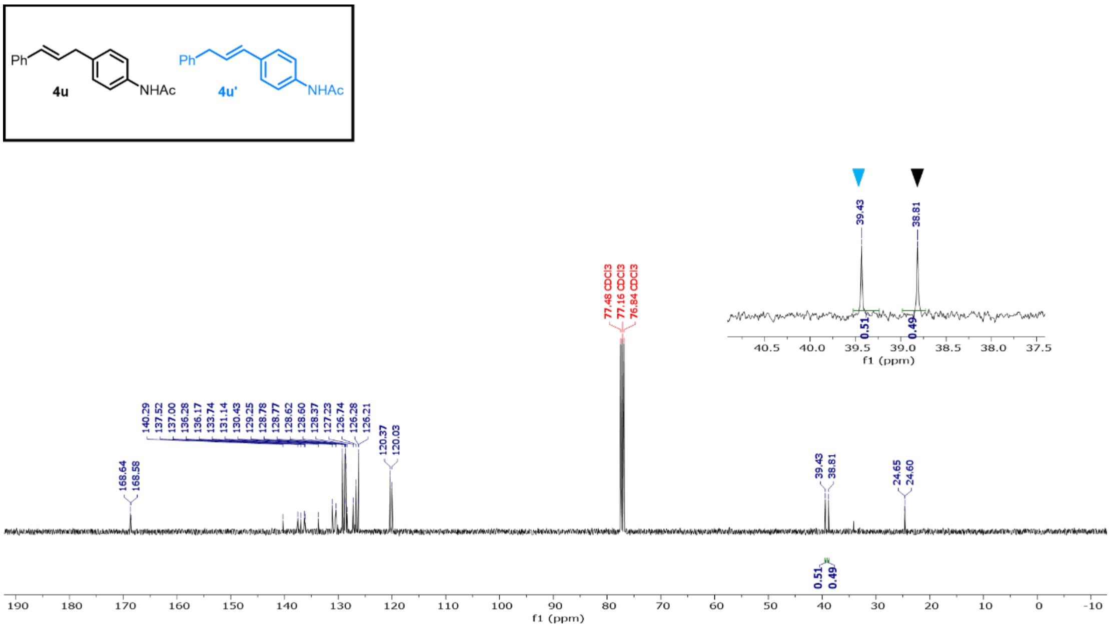

Figure S208 – ^{13}C NMR (101 MHz, CDCl$_3$, 298 K) spectrum of **4u+4u'**.

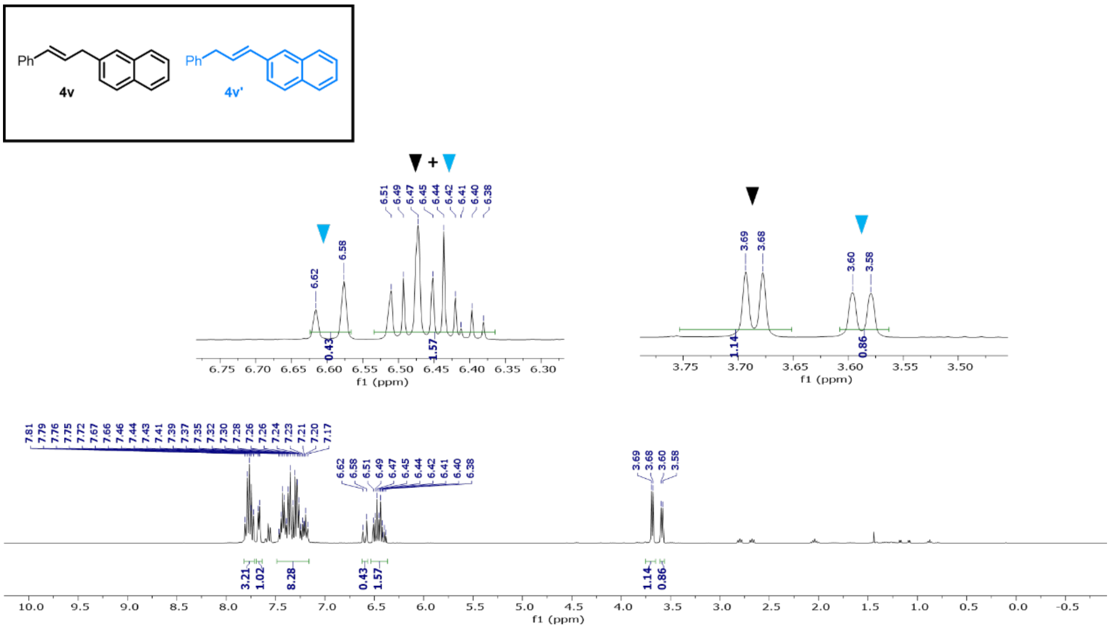

Figure S209 – ¹H NMR (400 MHz, CDCl₃, 298 K) spectrum of **4v+v'**.

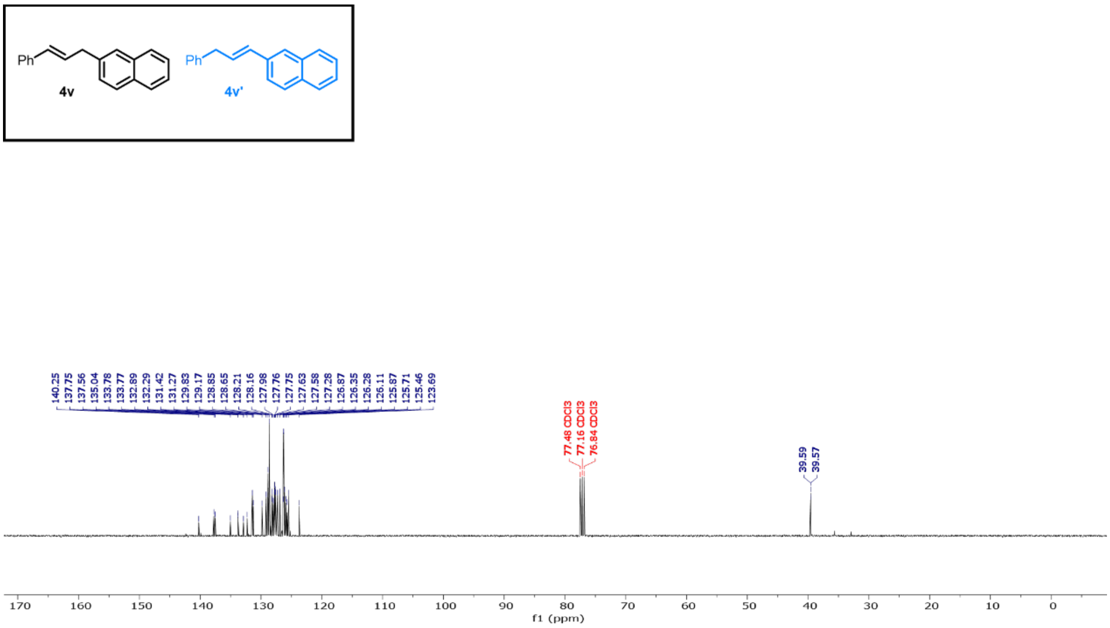

Figure S210 – ¹³C NMR (101 MHz, CDCl₃, 298 K) spectrum of **4v+4v'**.

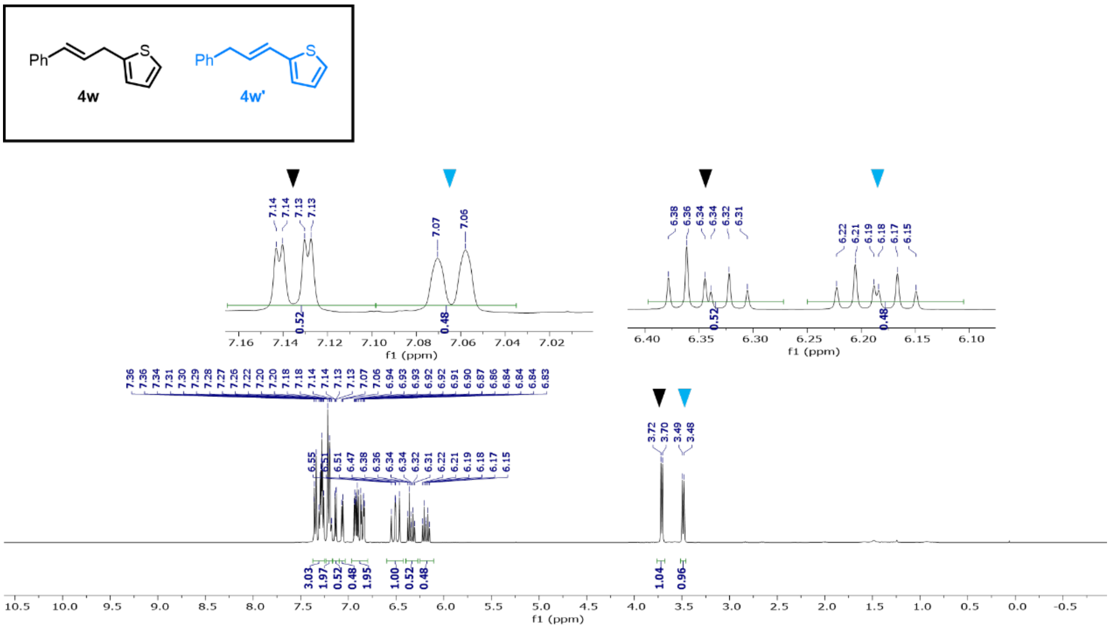

Figure S211 – ^{1}H NMR (400 MHz, CDCl$_3$, 298 K) spectrum of **4w+w'**.

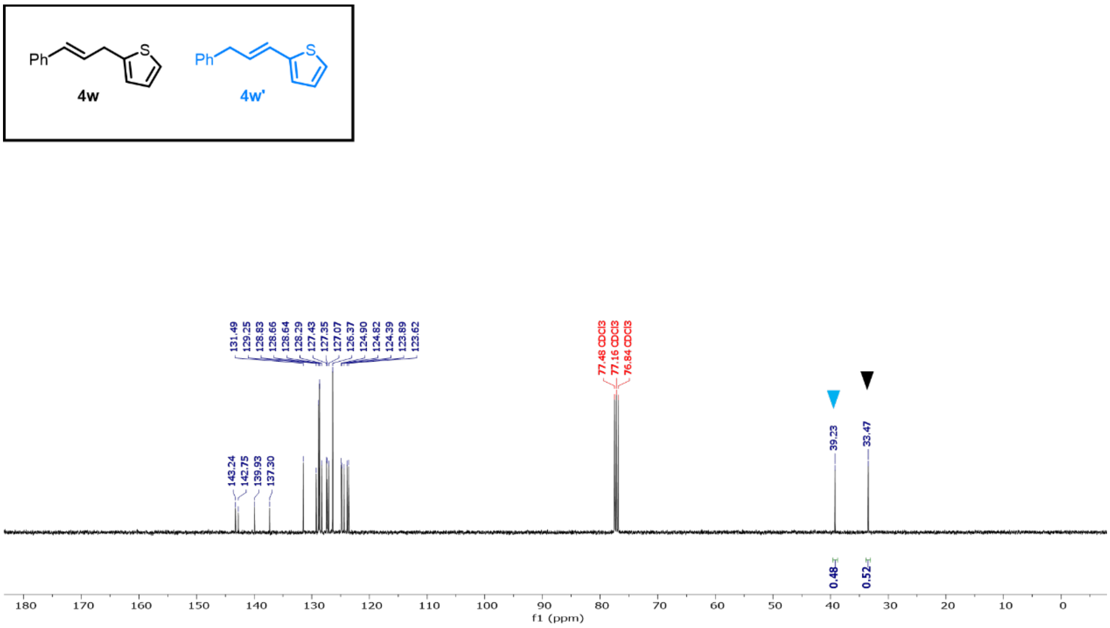

Figure S212 – ^{13}C NMR (101 MHz, CDCl$_3$, 298 K) spectrum of **4w+4w'**.

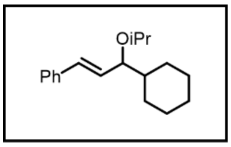

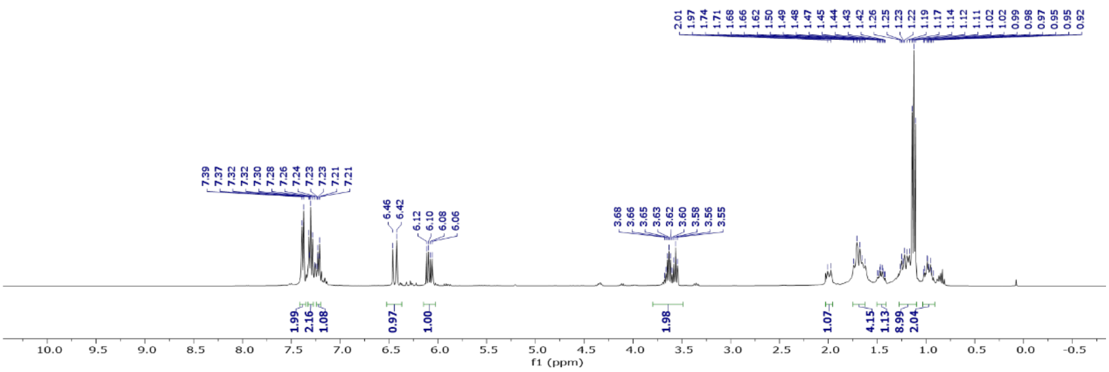

Figure S213 – ^{1}H NMR (400 MHz, CDCl$_3$, 298 K) spectrum of **4x**.

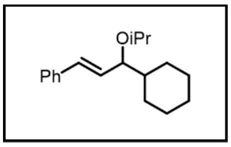

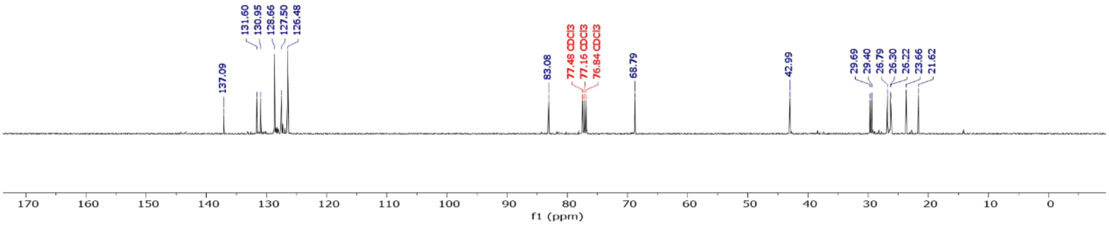

Figure S214 – ^{13}C NMR (101 MHz, CDCl$_3$, 298 K) spectrum of **4x**.

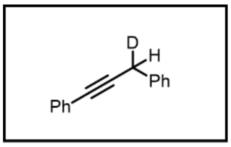

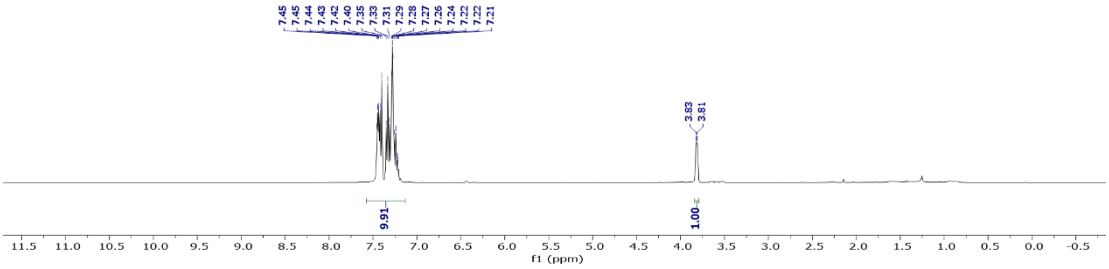

Figure S215 – ^{1}H NMR (400 MHz, CDCl$_3$, 298 K) spectrum of ***d$_1$*-2a**.

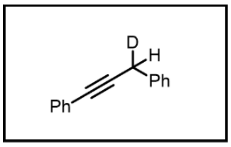

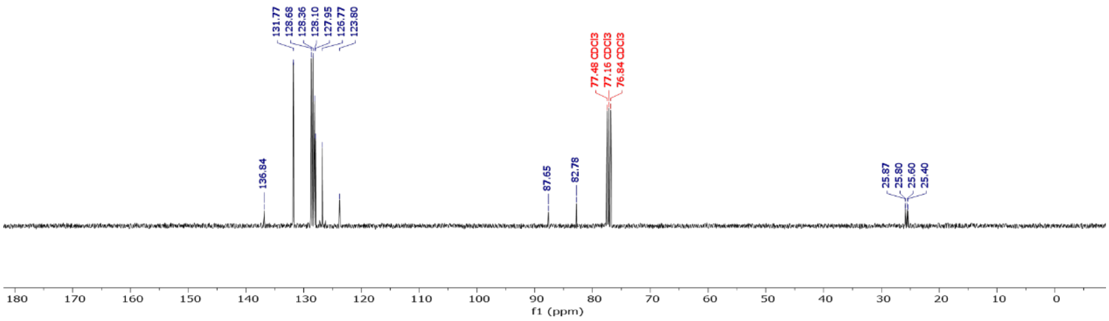

Figure S216 – ^{13}C NMR (101 MHz, CDCl$_3$, 298 K) spectrum of ***d$_1$*-2a**.

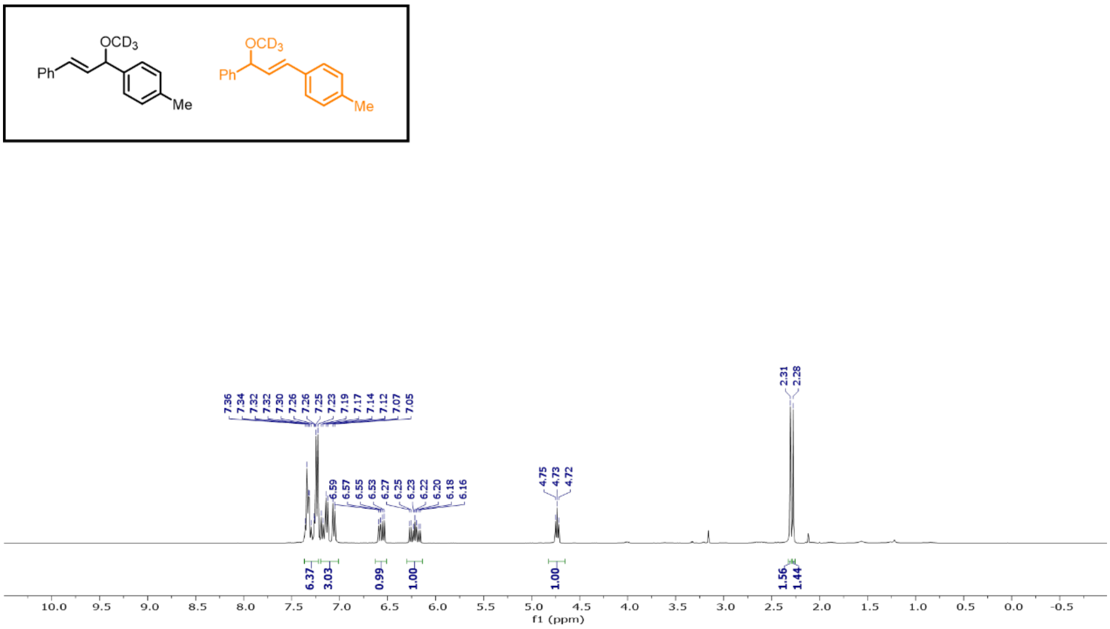

Figure S217 – ^{1}H NMR (400 MHz, CDCl$_3$, 298 K) spectrum of ***d_3*-3m+ *d_3*-3m’**.

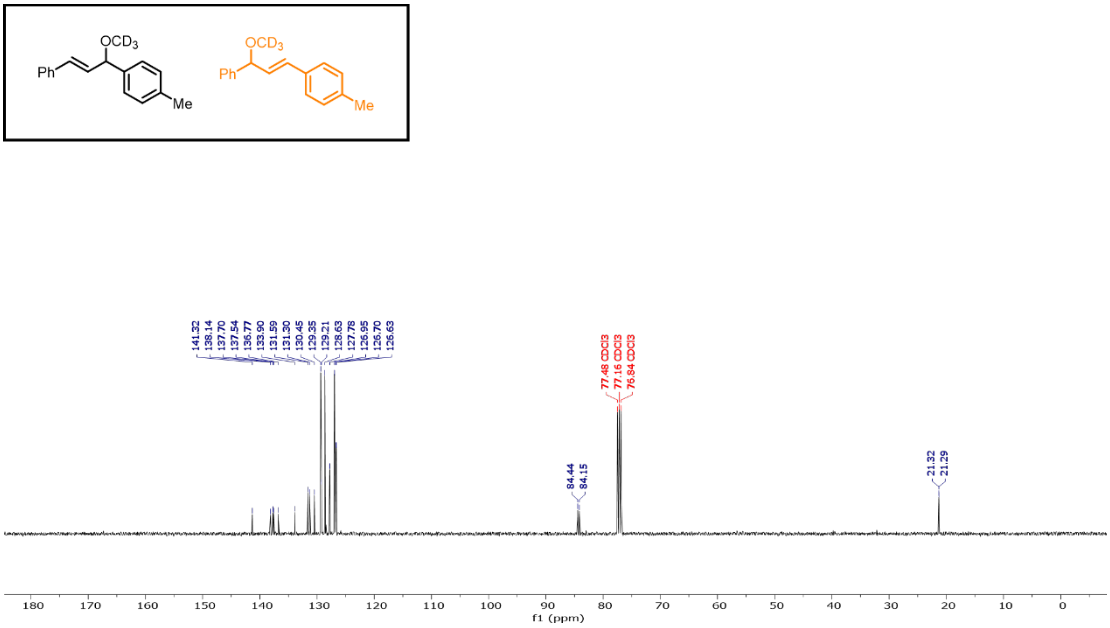

Figure S218 – ^{13}C NMR (101 MHz, CDCl$_3$, 298 K) spectrum of ***d_3*-3m+ *d_3*-3m’**.

13. References

[1] X. Yao, C. J. Li, *Org. Lett.* **2005**, *7*, 4395–4398.

[2] C. Liu, B. Wang, Z. Guo, J. Zhang, M. Xie, *Org. Chem. Front.* **2019**, *6*, 2796–2800.

[3] M. Yoshida, M. Higuchi, K. Shishido, *Org. Lett.* **2009**, *11*, 4752–4755.

[4] C. W. Downey, B. D. Mahoney, V. R. Lipari, *J. Org. Chem.* **2009**, *74*, 2904–2906.

[5] J. F. Bai, K. Yasumoto, T. Kano, K. Maruoka, *Angew. Chem., Int. Ed.* **2019**, *58*, 8898–8901.

[6] Y. Jeong, B. I. Kim, J. K. Lee, J. S. Ryu, *J. Org. Chem.* **2014**, *79*, 6444–6455.

[7] E. E. Wilson, A. G. Oliver, R. P. Hughes, B. L. Ashfeld, *Organometallics* **2011**, *30*, 5214–5221.

[8] A.-L. Zhang, L.-W. Yang, N.-F. Yang, J. Zhang, *J. Organomet. Chem.* **2015**, *775*, 88–93.

[9] Z. Mi, J. Tang, Z. Guan, W. Shi, H. Chen, *Eur. J. Org. Chem.* **2018**, *2018*, 4479–4482.

[10] J. B. Han, H. H. San, A. Guo, L. Wang, X. Y. Tang, *Adv. Synth. Catal.* **2020**, *363*, 2366–2370.

[11] K. Liu, D. Ding, W. Xing, L. Liu, S. Zhang, Q. Meng, T. Chen, *Org. Biomol. Chem.* **2023**, *21*, 1384–1388.

[12] S. Xu, Z. Zhang, C. Han, W. Hu, T. Xiao, Y. Yuan, J. Zhao, *J. Org. Chem.* **2019**, *84*, 12192–12197.

[13] J. Tunge, R. Torregrosa, S. Mendis, A. Davies, *Synthesis* **2018**, *50*, 3205–3216.

[14] X. Liu, R. Liu, J. Dai, X. Cheng, G. Li, *Org. Lett.* **2018**, *20*, 6906–6909.

[15] V. Balakrishnan, V. Murugesan, B. Chindan, R. Rasappan, *Org. Lett.* **2021**, *23*, 1333–1338.

[16] S. Li, J. Zhang, H. Li, L. Feng, P. Jiao, *J. Org. Chem.* **2019**, *84*, 9460–9473.

[17] M. Sai, *Adv. Synth. Catal.* **2018**, *360*, 3482–3487.

[18] S. Kong, L. Zhang, X. Dai, L. Tao, C. Xie, L. Shi, M. Wang, *Adv. Synth. Catal.* **2015**, *357*, 2453–2456.

[19] Y. Wang, Z. Shao, K. Zhang, Q. Liu, *Angew. Chem., Int. Ed.* **2018**, *57*, 15143–15147.

[20] D. N. Tran, C. Battilocchio, S. B. Lou, J. M. Hawkins, S. V. Ley, *Chem. Sci.* **2015**, *6*, 1120–1125.

[21] M. B. Li, Y. Wang, S. K. Tian, *Angew. Chem., Int. Ed.* **2012**, *51*, 2968–2971.

[22] G. Hamasaka, F. Sakurai, Y. Uozumi, *Chem. Commun.* **2015**, *51*, 3886–3888.

[23] G. Ortar, *Tetrahedron Lett.* **2003**, *44*, 4311–4314.

[24] T. Pols, H. R. Sikkema, B. F. Gaastra, J. Frallicciardi, W. M. Smigiel, S. Singh, B. Poolman, *Nat. Commun.* **2019**, *10*, 4239.

[25] B. Yang, Z. X. Wang, *J. Org. Chem.* **2017**, *82*, 4542–4549.

[26] V. Chugh, B. Chatterjee, W. C. Chang, H. H. Cramer, C. Hindemith, H. Randel, T. Weyhermuller, C. Fares, C. Werlé, *Angew. Chem., Int. Ed.* **2022**, *61*, e202205515.
